# Photocyclization by a triplet–triplet annihilation upconversion pair in water – avoiding UV-light and oxygen removal†

**DOI:** 10.1039/d3sc03242f

**Published:** 2023-08-31

**Authors:** R. Jeyaseelan, M. Utikal, C. G. Daniliuc, L. Næsborg

**Affiliations:** a Westfälische Wilhelms-Universität Münster, Organisch-Chemisches Institut Corrensstraße 40 48149 Münster Germany lnaesbor@uni-muenster.de

## Abstract

We present a formal [2 + 2]-cycloaddition of unsaturated ketones enabled by a green-to-ultraviolet triplet–triplet annihilation upconversion (TTA-UC) pair, using commercially available Ru(bpy)_3_^2+^ and pyrene as sensitizer and annihilator, respectively. In the developed protocol, visible light irradiation at *λ*_max_ = 520 nm allows for the reaction to proceed without the need for UV-light and the aqueous medium eliminates the need for oxygen removing protocols. Through this study, the application of the readily available upconversion pair is broadened to include cyclization reactions. We showcase the utility of the system by generating bicyclo[2.1.1]hexanes that are valuable bioisosteres of *ortho*-substituted benzenes, a promising motif for pharmaceuticals.

## Abstract 2

A commercially available upconversion pair is applied to promote a photocyclization for the synthesis of bioisosteres using green light irradiation. The reaction concept is enabled to run in water without oxygen removal by a micellar medium.

## Graphical abstract

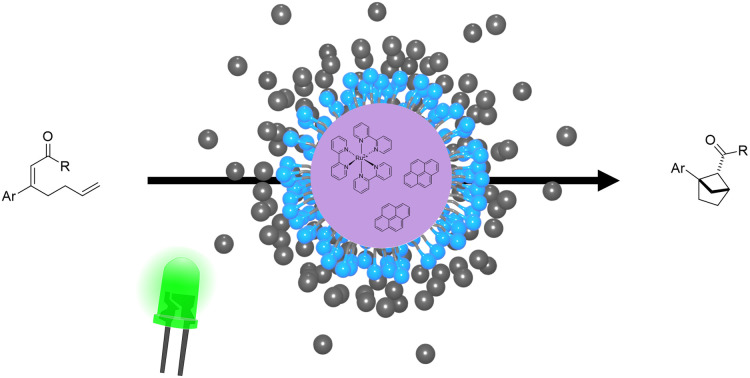

## Introduction

In the past decades visible light photochemistry has received increasing attention, especially following the commercialization of blue LEDs in the early 90s.^1^ Despite the progress of visible light photochemistry, some photochemical transformations require UV-light for sufficient reactivity. Compared to visible light sources, UV-light sources are energy inefficient and environmentally unfriendly, and present harsh reaction conditions.^2,3^ In this context, upconversion processes based on for example triplet–triplet annihilation have the potential to be a part of the solution by enabling milder reaction conditions. They exploit the energy of two photons of a lower energy wavelength to access higher energy excited states whereby visible light can provide “UV-reactivity”. The bimolecular process of TTA-UC requires a sensitizer that absorbs the light, enters the triplet state, and undergoes energy transfer to an annihilator. From two triplet state annihilators, a ground state annihilator and a higher energy singlet state annihilator can be formed potentially fluorescing in the UV region.^4–7^

**Scheme 1:**
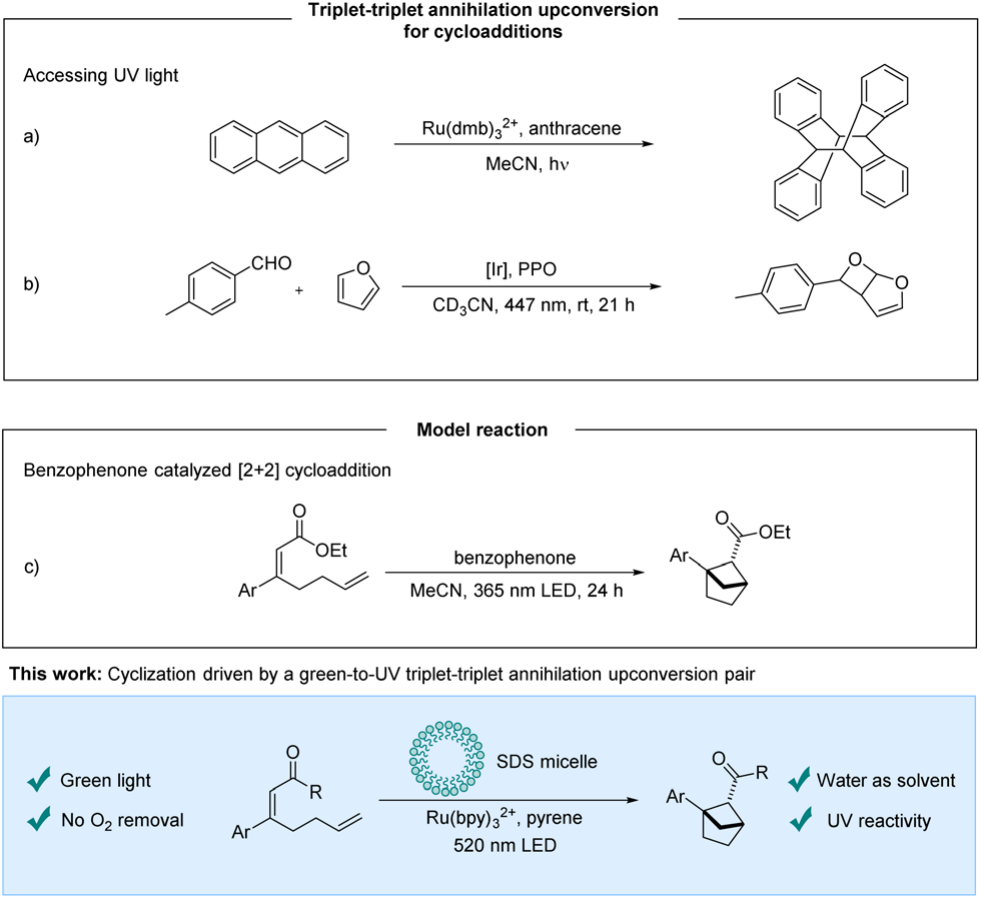
Previous work and this work. PPO = 2,5-diphenyloxazole.

TTA-UC has gained increasing attention from the photochemistry community and many applications such as solar energy applications,^8,9^ bioimaging^9–11^ and phototherapeutics^9,10^ are being explored. Although efforts towards synthetic applications have been made, the opportunity to use visible light to access energetically higher excited states corresponding to UV-reactivity holds an unfulfilled promise. The underdevelopment of TTA-UC mediated synthetic protocols may be due to the complex photocatalytic system that has to work in an interplay of mechanisms that are challenging to optimize and predict. In addition, upconversion strategies rely on multiple subsequent steps that all must function well to obtain satisfactory productivity. A single nonfunctioning step could lead to false negatives in screenings for reactivity. Recently, protocols for protodehalogenation^5,6,12–16^ and reductive C–C couplings of aryl halides with heteroaromatics or arenes^5,6,12,14,17^*via* TTA-UC have been disclosed, whereas the application of TTA-UC for cyclizations is limited.^16,18–20^ Cyclizations in which TTA-UC protocols have been utilized for red-to-blue and red-to-orange systems have been previously disclosed.^16,18^ Cyclizations using TTA-UC systems that access UV light are rare, and have been explored to perform a [4 + 4]-cycloaddition of the annihilator itself (Scheme 1a).^19^ Very recently, during the preparation of this manuscript, Wenger *et al.* demonstrated an elegant example of visible-to-UV TTA-UC for a Paterno–Büchi reaction (Scheme 1b).^20^

We sought to identify a TTA-UC system that can generate UV-light to promote cyclization reactions. Various sensitizer/annihilator pairs have been reported, providing a range of available upconverted emission wavelengths.^3,4,21^ One such pair, which has been reported,^22,23^ is [Ru]-based photosensitizers and pyrene derivatives providing upconverted emission at around 390 nm.^3^ We set out to expand the application of the upconversion system to include a cyclization. As a model reaction for our investigations, the intramolecular [2 + 2]-cycloaddition of α,β-unsaturated esters forming bicyclo[2.1.1]hexanes was selected (Scheme 1c). These target compounds are bioisosteres of *ortho*-substituted benzenes and are a valuable motif for potential pharmaceutical applications. Mykhailiuk *et al.* activated the esters with benzophenone as the photosensitizer using UV-light irradiation.^24^ We hypothesized that the singlet excited state of pyrene accessed *via* triplet–triplet annihilation could excite the benzophenone photosensitizer by an emission–absorption process. Preliminary experiments showed that benzophenone could not promote the desired photocycloaddition under our reaction conditions. As an alternative strategy, we designed a ketone substrate that could also be subject to an electron transfer pathway. We tested the α,β-unsaturated ketone substrate 1a, and were delighted to obtain the desired bioisostere 2a.

## Results and discussion

With Ru(bpy)_3_(PF_6_)_2_ and pyrene as a suitable sensitizer/annihilator pair we continued our investigations into the cyclization of α,β-unsaturated ketone 1a to form the desired bicyclo[2.1.1]hexanes 2a. Using an aqueous sodium dodecyl sulfate (SDS) solution under green-light irradiation led to the desired ketone 2a in 62% NMR yield (Table 1, entry 1). Previous work from our group^25^ showed that an oxygen sensitive photocycloaddition promoted by [Ru(bpy)_3_]^2+^, which has been used as an oxygen sensor,^26^ could be performed in micellar media without freeze–pump–thaw protocols. We hoped to observe similar effects in our protocol for a highly oxygen sensitive TTA-UC process,^27^ and did not make any efforts to remove oxygen. This combination of micellar photocatalysis and upconversion allowed for a simple protocol that is accessible to synthetic chemists outside the field of photochemistry.

**Table 1:** Optimization of the reaction condition

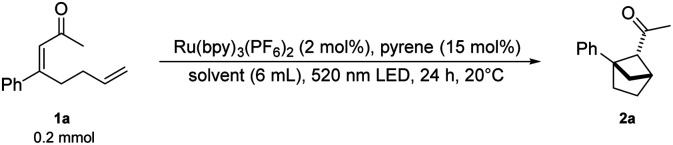				
Entry^a^	Sensitizer	Annihilator	Solvent	NMR yield
1^b^	[Ru]^2+^	Pyrene	SDS (4 wt%)	62
2^b^	[Ru]^2+^	Pyrene	Triton-X-100 (4 wt%)	48
3^b^	[Ru]^2+^	Pyrene	CTAC (4 wt%)	12
4^c^	[Ru]^2+^	Pyrene	SDS (4 wt%)	76 (80^d^)
5^c^	[Ru]^2+^	Pyrene	Acetonitrile, no O_2_	52
6^c^	[Ru]^2+^	Pyrene	DCM, no O_2_	45
7^c^	[Ru]^2+^	Pyrene	MeOH, no O_2_	84 (79^d^)
8^c^	[Ru]^2+^	Pyrene	MeOH	64
9^c^	[Ru]^2+^	Pyrene	SDS (4 wt%), no O_2_	79
10^c^	[Ru]^2+^	Pyrene	H_2_O, no O_2_	22
11^c^	[Ru]^2+^	—	SDS (4 wt%)	28
12^c^	—	Pyrene	SDS (4 wt%)	0
13^c^	—	—	SDS (4 wt%)	0
14^e^	[Ru]^2+^	Pyrene	SDS (4 wt%)	0

a For the determination of the NMR yield, toluene (5.3 μL, 0.05 mmol) was used as internal standard.

b 3 W 520 nm LED.

c 10 W 520 nm LED.

d Yields of isolated product.

e No light. SDS = sodium dodecyl sulfate, CTAC = cetyltrimethylammonium chloride.

As head-group charges are expected to influence the reaction, a neutral and a positively charged amphiphile were tested. Using the neutral amphiphile Triton-X-100 resulted in a lower NMR yield of 48% (Table 1, entry 2) and the positively charged amphiphile cetyltrimethylammonium chloride (CTAC) decreased the NMR yield to a mere 12% (Table 1, entry 3). The drastic decrease of reactivity using a positively charged amphiphile may be caused by repulsion of the positively charged photosensitizer. Changing from 3 W to 10 W green LEDs increased the product formation and the bicyclo[2.1.1]hexane 2a was obtained in 78% isolated yield (Table 1, entry 4). To verify the benefit of using micellar solutions, organic solvents were tested. Acetonitrile and dichloromethane degassed by a freeze–pump–thaw protocol provided the product 2a in moderate yields (52% and 45% NMR yield respectively, Table 1, entries 5 and 6). Degassed methanol provided the desired product in 79% isolated yield (Table 1, entry 7) whereas leaving out a freeze–pump–thaw procedure decreased the NMR yield by 20% (Table 1, entry 8). We were delighted to see that the aqueous sodium dodecyl sulfate (SDS) solution provides the desired product in similar NMR yields with or without oxygen removal (Table 1, entries 4 and 9). Control experiments demonstrated that light, sensitizer, annihilator and surfactant are all necessary for an efficient formation of the desired product (Table 1, entries 10–14). To our surprise, 28% NMR yield could be observed without pyrene although the triplet energy of the substrate is expected to be too high for energy transfer to take place. This observation could be explained by the micelles enabling energy transfer from the sensitizer to the substrate. In this context we have previously proposed micellar substrate activation for energy transfer catalysis.^21^ Owing to the simpler reaction setup we continued our investigations using SDS-micelles as the reaction medium.

**Scheme 2:**
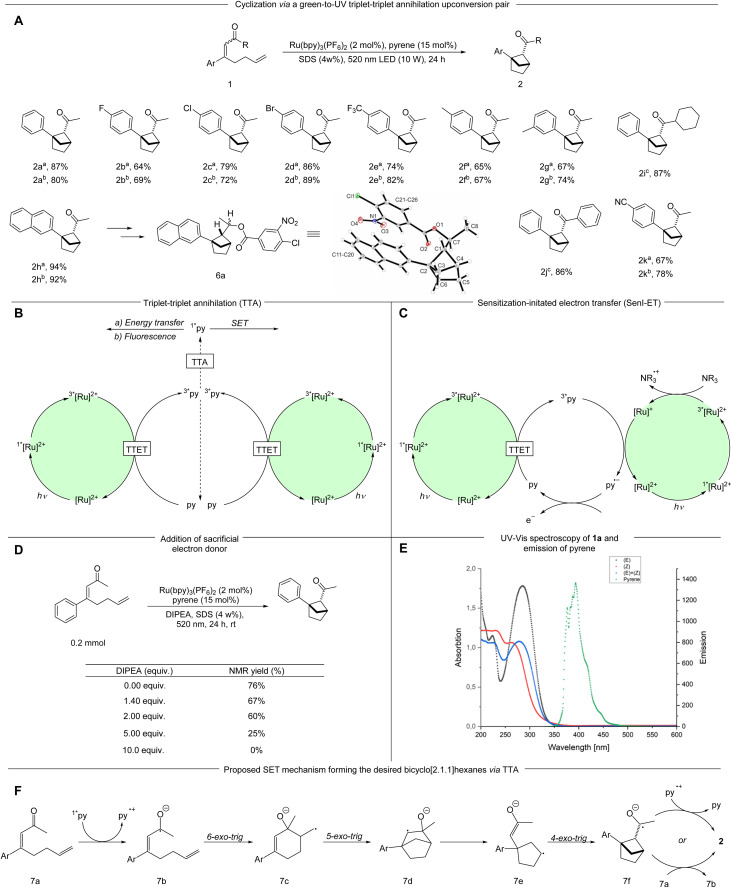
An array of substrates undergoing the cyclization mediated by a TTA-UC pair and mechanistic proposal of the formation of 2. (A) ^*a*^(*Z*)-Isomer of the substrate 1; ^*b*^(*E*)-isomer of the substrate 1. ^*c*^Mixture of (*E*)- and (*Z*) isomer of the substrate.

With the optimized conditions in hand, various α,β-unsaturated ketones were examined (Scheme 2A). The synthesis of starting materials led to the formation of both possible diastereomers of the substrate, both of which were applied in the reaction protocol separately when possible. Seemingly, the alkene geometry of the starting material has no influence on the diastereomeric outcome of the reaction. Different electron-withdrawing substituents in the *para* position such as halogens (1b–1d) a cyano- (1k) and a trifluoromethyl group (1e) are tolerated leading to the desired bicyclo[2.1.1]hexanes-derivatives in good to very good yields (2b–2e; 2k 64–89%). Introduction of an electron donating methyl group in the *meta* and *para* positions respectively resulted in the product being formed in good yields (2f, 2g 65–77%). Notably, a β-napthyl- substituted ketone underwent the targeted transformation in an excellent yield (2h 92–94%). The relative configuration and structure were verified by X-ray analysis of derivative 6a. A cyclohexyl- and a phenyl-ketone were tested (1i, 1j), each leading to very high yields of the desired bicyclic product (2i, 2j 86–87%).

As the reaction takes place in a complex reaction medium with a multicomponent system the determination of the underlying mechanism is not simple. Moore *et al.* demonstrated that pyrene can undergo TTA to form the annihilator singlet state (^1*^py) in the presence of [Ru(bpy)_3_](PF_6_)_2_ as the sensitizer (Scheme 2B).^23^ When an additional sacrificial electron donor *i.e. N*,*N*-diisopropylethylamine (DIPEA) is present the excited ^3*^[Ru(bpy)_3_]^2+^ is likely reduced *via* SET (single electron transfer) to [Ru(bpy)_3_]^+^, which in turn can reduce the ^3*^py to the pyrene radical anion, thereby severely diminishing the TTA pathway (Scheme 2C).^23^ The formed radical anion has a high reduction potential (2.1 V *vs.* SCE), which has been applied for reductive C–C couplings between aryl halides and heteroaromatics or arene derivatives.^28^ In this context, we investigated the effect of adding varying amounts of DIPEA to the reaction. Upon addition of 1.4 eq. of DIPEA, which is previously reported to be important for a sensitization-initiated electron transfer (SenI-ET) pathway,^28^ the NMR yield decreased to 67%. Increasing amounts of the electron donor led to further reductions in the NMR yields (Scheme 2D). These experiments support formation of the ^1*^py *via* TTA as a likely pathway in our reaction as the presence of sacrificial electron donor is known to form the pyrene radical anion *via* SenI-ET.^23,28^ Furthermore, we applied another upconversion system with *fac*-[Ir(ppy)_3_] and pyrene for this transformation. In comparison to Ru(bpy)_3_^2+^, *fac*-[Ir(ppy)_3_] is more difficult to reduce by a sacrificial electron donor and thereby favours the TTA over the SenI-ET pathway.^15^ Using this system we obtained 64% NMR yield of the desired product. Control experiments without pyrene resulted in 28% NMR yield (see ESI†). Additionally, the alternate system only shows a minor decrease in NMR yield with DIPEA present, further strengthening TTA as a likely activation mode (see ESI†).

From the ^1*^py accessed by TTA we consider three possibilities: (1) the relaxation of ^1*^py with emission of upconverted, more energetic photons that can be absorbed by the substrate, (2) Foerster resonance energy transfer (FRET) or (3) direct SET reduction of the substrate by ^1*^py. The first two pathways require spectral overlap, and were investigated *via* spectroscopic analyses: UV-vis absorption spectra of both diastereomers of 4-phenylocta-3,7-dien-2-one (1a) were measured in SDS solution and compared to the fluorescence emission of pyrene (in SDS solution). No productive overlap could be identified, making these pathways unlikely (Scheme 2E and Fig. S9 and S10†).

**Fig. 1:**
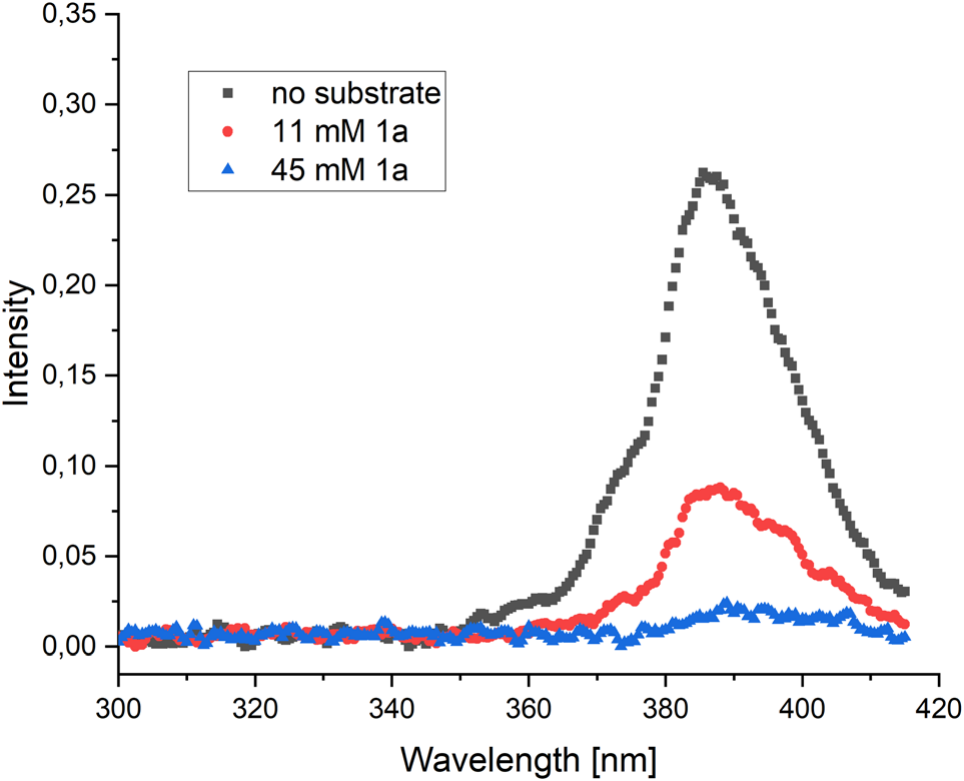
Pyrene fluorescence, produced *via* Ru(bpy)_3_^2+^ and pyrene in aqueous SDS solution, without the presence of substrate (black) and with varying amounts of substrate 1a added as a quencher (red, blue).

The third pathway is enabled by direct SET from ^1*^py which could lead to the ketyl radical 7b. The ketyl radical 7b can undergo a 6-*exo-trig* cyclization forming the primary radical 7c, which, after a subsequent cyclization and fragmentation-cyclization sequence could lead to the product through SET oxidation, possibly by a pyrene radical cation or another substrate molecule (Scheme 2F). To examine the feasibility of the SET reduction, the SET reduction potential of both diastereomers of the substrate 1 were measured in acetonitrile. Given the estimated reduction potential of singlet state pyrene (−2.1 V *vs.* SCE)^29^ and the measured reduction potential of the substrates (−1.87 V *vs.* SCE for the (*E*)-isomer and −1.82 V *vs.* SCE for the (*Z*)-isomer, see ESI†), the SET process should be viable. To further support our mechanistic hypothesis, we set out to perform pyrene fluorescence quenching experiments with substrate 1a as the quencher. As the substrate and pyrene absorb in the same region, we produced pyrene fluorescence using an SDS-micelle solution of Ru(bpy)_3_^2+^ and pyrene, irradiating at 450 nm (see ESI† for further details). A weak pyrene fluorescence signal could be produced (Fig. 1, black) and addition of substrate 1a in varying amounts could quench the signal (Fig. 1, red and blue). These results support a mechanism where pyrene is quenched by the substrate as outlined in our proposed mechanism (Scheme 2F).

## Conclusion

In conclusion, we have expanded the current synthetic possibilities using green light for “UV-reactivity” in water without the need for oxygen removing protocols. A commercially available green-to-UV TTA-UC pair was used in a formal [2 + 2]-cycloaddition to generate bioisosteres. We hope that by broadening the application of triplet–triplet annihilation upconversion, we can start to perform “UV-light photochemistry” under milder and more benign conditions.

## Data availability

The data supporting the findings in this article are presented in the manuscript or available in the ESI.†

## Author contributions

Experiments were performed by R. J. and M. U. The project was guided by L. N. All authors contributed to writing the manuscript. C. G. D. performed the crystallographic analysis.

## Conflicts of interest

There are no conflicts to declare.

## Supplementary Material

SC-014-D3SC03242F-s001

SC-014-D3SC03242F-s002
