# Supplementary material for: Photocyclization by a triplet–triplet annihilation upconversion pair in water – avoiding UV-light and oxygen removal
Source: Chem Sci. 2023 Aug 31;14(40):11040–4. doi: 10.1039/d3sc03242f (PMC10583691; doi:10.1039/d3sc03242f)
Supplement: SC-014-D3SC03242F-s001 [file SC-014-D3SC03242F-s001.pdf]

Electronic Supplementary Material (ESI) for Chemical Science.
This journal is © The Royal Society of Chemistry 2023

Supporting Information

Photocyclization by a Triplet-Triplet Annihilation Upconversion Pair in Water – Avoiding UV-light and Oxygen Removal

Rubaishan Jeyaseelan[+], Martin Utikal[+] and Dr. Line Næsborg

Organisch-Chemisches Institut, Westfälische Wilhelms-Universität, Corrensstrasse 40, 48149 Münster

Email: lnaesbor@uni-muenster.de

Table of Contents

1. General information 2

2. Synthesis of α,β-unsaturated ketones and characterization data 4

3. Optimization of the intramolecular [2+2] cycloaddition 24

4. Synthesis and characterization data of bicyclo[2.1.1]hexanes and derivatives 25

5. Determination of *E*/*Z* configuration of the α,β-unsaturated ketones 32

6. Determination of relative configuration of bicyclo[2.1.1]hexanes 34

7. Mechanistic experiments 36

 7.1 UV-vis absorption spectra and emission spectra 36
 7.2 Luminescence quenching experiments 38
 7.3 Cyclic voltammetry 40
 7.4 Mechanistic investigation 42

8. X-Ray Diffraction Analysis 43

9. NMR Spectra 45

10. Supplementary References 106

1. General information

Oxygen sensitive reactions were performed in oven-dried glassware under argon atmosphere using standard *Schlenk* technique. Anhydrous solvents used in the reactions were either distilled freshly or purchased commercially as extra-dry solvents from *Acros Organics*. All commercially available reagents were obtained from *Sigma Aldrich, Alfa Aesar, Acros, ABCR, TCI, Fluorochem* or *BLDPharm* and were used without further purifications. Flash column chromatography (FC) was performed on *Merck Geduran Si 60* (40-63 µm) or *VWR* silica gel 60 (40-63 µm) with compressed air pressure. Solvents for the purification were distilled prior to use. Diethylether was distilled over ferrous sulfate heptahydrate. The reaction monitoring was performed on thin layer chromatography (TLC) purchased from *Merck silica gel 60 F254*-plates and detected using UV light or Anisaldehyde stain (0.5% anisaldehyde, 10% glacial acetic acid, 85% MeOH, 5% conc. H_2SO_4).

^{1}H-NMR (300 MHz, 500 MHz and 600 MHz), ^{13}C-NMR (75 MHz, 126 MHz and 151 MHz) and ^{19}F-NMR (282 MHz) were recorded on *Bruker Avance II 300, Agilent DD2 500* and *Agilent DD2 600* spectrometers at 299 K. The chemical shifts are stated in ppm and referenced to the solvent residual peak of $CDCl_3$ (δ = 7.26 ppm). The multiplets are given as singlet (s), doublet (d), triplet (t), quartet (q) and multiplet (m). The mass spectra were measured on the *Thermo Fischer Scientific LTQ Orbitrap XL* and the peaks were displayed in *m/z*.

Photoreaction setup

The photoreactions were performed in a custom-made photoreactor equipped with 3 W and 10 W green LEDs (emission maxima: 520 nm) supplied by *Avonec*. The water cooling of the reaction has been performed using a chiller purchased by *Huber* (*Huber minichiller 280*) and the temperature was set to 20°C. The photoreactions were performed in 10 mL headspace vials supplied by *Omnilab* (Headspacevial ND20, 10 ml, 46 x 22.5 mm, rounded bottom). The vials were placed in designated spots of the photoreactor and each reaction was irradiated by one LED from below, maintaining an equal distance to the light source for each reaction. To ensure comparable light intensity for each sample, illuminance in cd·sr·m^{-2} was measured three times for each LED 143 mm from the LEDs, using a *PeakTech 5030*. For the 3 W and 10 W

LEDs illuminances of 8383 cd·sr·m^{-2}(spot A), 7850 cd·sr·m^{-2} (spot B), 9240 cd·sr·m^{-2} (spot C) and 52567 cd·sr·m^{-2}(spot D), 53367 cd·sr·m^{-2} (spot E), 52634 cd·sr·m^{-2} (spot F) were measured respectively.

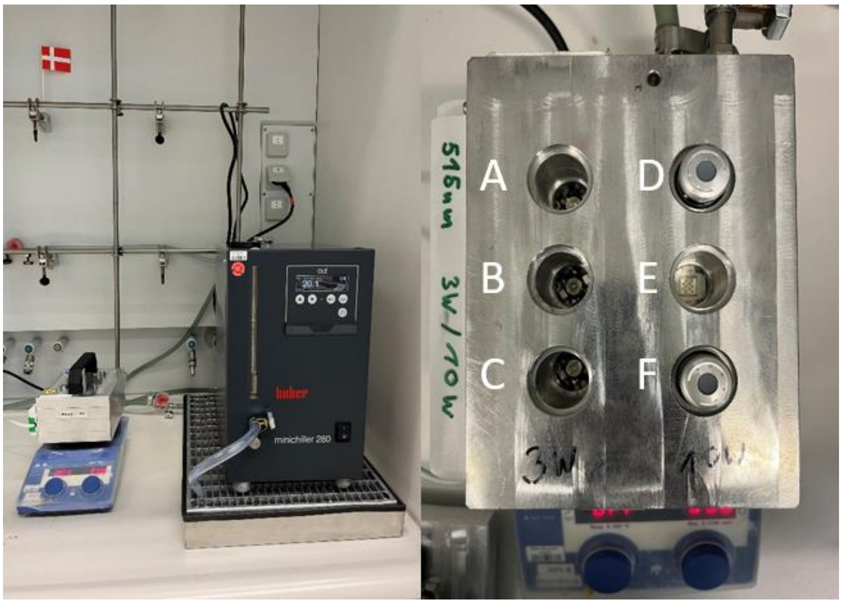

Figure S1: Photoreaction setup

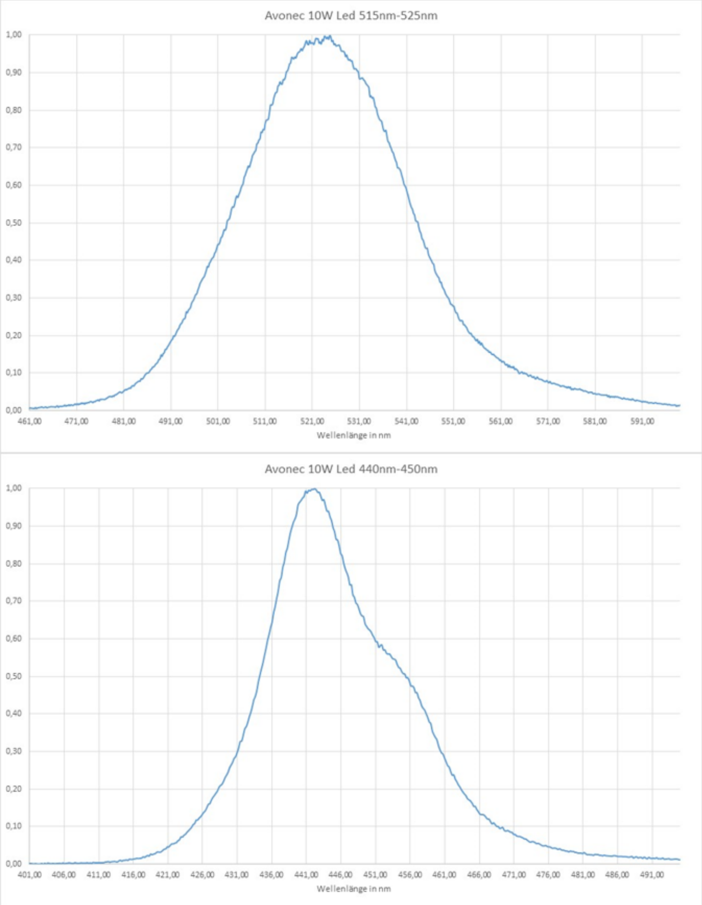

Figure S2: Emissionspectrum of the irradiation source.

2. Synthesis of α,β-unsaturated ketones and characterization data

General procedure for the synthesis of the starting materials

Synthesis of 1-Aryl-pent-4-en-1-one derivatives (GP1)

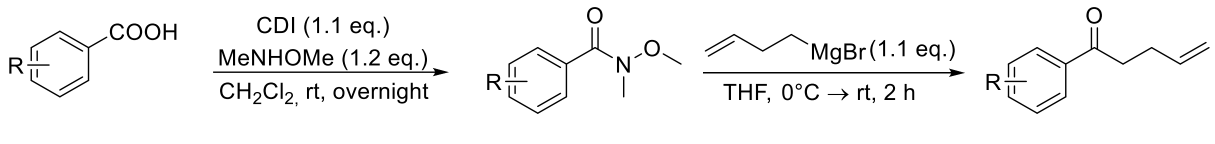

In accordance to a procedure of Mykhailiuk *et al.,* the benzoic acid derivative (12.0 mmol, 1.0 eq.) was dissolved in dry dichloromethane (1.0 M) and carbonyldiimidazole (CDI) (2.14 g, 13.2 mmol, 1.1 eq) was added portion wise under argon. After stirring the reaction mixture for 2 h at room temperature, N,O-dimethylhydroxylamine hydrochloride (1.40 g, 14.4 mmol, 1.2 eq.) was added portion wise and the reaction stirred overnight at room temperature. Afterwards dichloromethane and saturated, aqueous NaCl solution were added and the phases were separated. The aqueous phase was extracted with dichloromethane twice. The combined organic phases were dried over $MgSO_4$, filtrated and the solvent was removed under reduced pressure. The resulting Weinreb amide was used without further purification.[1]

In the second step, the Grignard-reagent was prepared according to a procedure of Waser *et al.* To a suspension of magnesium (321 mg, 13.2 mmol, 1.10 eq.) and iodine (small crystal) in dry tetrahydrofuran (20.0 mL) a solution of 4-bromo-but-1-ene (1.33 mL, 13.2 mmol, 1.10 eq.) in dry tetrahydrofuran (14 mL) was added slowly. After stirring for 2.5 h a solution of the corresponding Weinreb amide in dry tetrahydrofuran (3.0 mL) was added slowly at 0°C. The reaction mixture was stirred at 0°C for 1 h and followed by 1 h at room temperature. Afterwards the reaction was quenched with a saturated aqueous NH_4Cl solution. Water was added and the mixture extracted using ethyl acetate. The combined organic phases were dried over $MgSO_4$, filtrated and the solvent removed under reduced pressure. The product was received after column chromatography. [2]

Horner-Wadsworth-Emmons olefination of the 1-Aryl-pent-4-en-1-one (GP2)

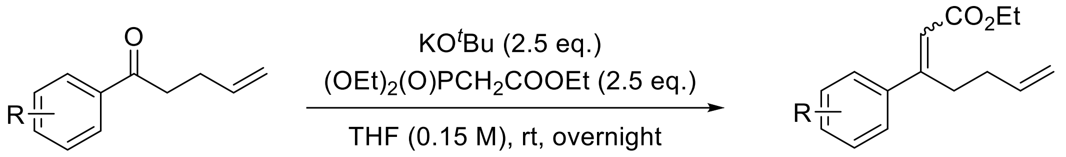

In accordance to a procedure by Mykhailiuk *et al.*, potassium *tert*-butoxide (2.5 eq.) was dissolved in tetrahydrofuran (0.15 M) and ethyl(diethoxyphosphoryl) acetate was added slowly. The reaction mixture was stirred for 30 min at room temperature and afterwards the unsaturated ketone (1.0 eq.) was added. After stirring at room temperature overnight, the solvent was removed. Afterwards ethyl acetate and water were added and the phases separated. The organic phase was washed with water and saturated aqueous NaCl solution, dried over $MgSO_4$, filtrated and the solvent removed under reduced pressure. The diastereomeric products of the reaction were isolated after column chromatography. [1]

Synthesis of a,ß unsaturated ketones (GP3)

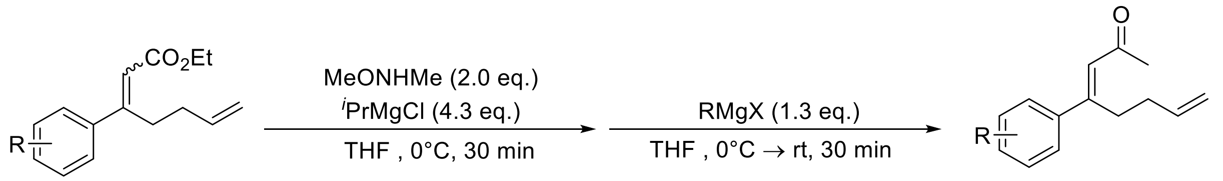

In an oven dried Schlenk tube the unsaturated ester derivative (1.0 eq.) was dissolved in dry tetrahydrofuran (0.33 M). Afterwards N,O-dimethylhydroxylamine hydrochloride (2.0 eq.) was added and the reaction mixture was cooled to 0°C. Subsequently *iso*-propylmagnesium chloride (2.0 M in diethylether, 4.3 eq.) was added slowly and the reaction stirred at 0°C for 30 min. Afterwards the reaction was quenched with saturated, aqueous NH_4Cl solution and transferred to a separatory funnel. The phases were separated and the aqueous phase was extracted once with ethyl acetate. The combined organic phases were dried over $MgSO_4$ and the solvent was removed under reduced pressure. The resulting Weinreb amide (1.0 eq.) was dissolved in dry tetrahydrofuran (0.29 M) and the Grignard-reagent (1.3 eq.) was added at 0°C and stirred for 30 min at room temperature. The reaction mixture was quenched by addition of saturated, aqueous NH_4Cl solution. Afterwards the phases were separated, and the aqueous phase extracted with ethyl acetate. The combined organic phases were dried over $MgSO_4$ and the solvent removed under reduced pressure. The crude product was isolated via FC[3]

1-Phenylpent-4-en-1-one (**3a**)

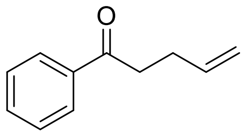

1-Phenylpent-4-en-1-one (**3a**) was prepared according to general procedure GP1 with benzoic acid (1.83 g, 15.0 mmol, 1.00 eq.), carbonyldiimidazole (2.68 g, 16.5 mmol, 1.09 eq.), N,O-dimethylhydroxylamine hydrochloride (1.76 g, 18.0 mmol, 1.20 eq.), magnesium (397 mg, 16.3 mmol, 1.10 eq.), 4-bromo-but-1-ene (1.60 mL, 16.0 mmol, 1.10 eq.), dichloromethane (15 mL) and tetrahydrofuran (20 mL). The product was isolated as colourless liquid (1.84 g, 11.5 mmol, 77%) after flash column chromatography (pentane : ethyl acetate = 95:5).

^{1}H-NMR (300 MHz, $CDCl_3$, 299 K): δ (ppm) = 8.01 – 7.92 (m, 2H), 7.62 – 7.51 (m, 1H), 7.51 – 7.41 (m, 2H), 5.91 (ddt, J = 16.8, 10.2, 6.5 Hz, 1H), 5.16 – 4.94 (m, 2H), 3.08 (dd, J = 7.9, 6.9 Hz, 2H), 2.59 – 2.42 (m, 2H).

^{13}C-NMR (75 MHz, $CDCl_3$, 299 K): δ (ppm) = 199.5, 137.4, 137.0, 133.1, 128.7, 128.1, 115.4, 37.8, 28.2.

HRMS (ESI) *m/z*: $[M+Na]^+$ calculated for $C_{11}H_{12}ONa^+$: 183.0781; found: 183.0779.

1-(4-Fluorophenyl)pent-4-en-1-one (**3b**)

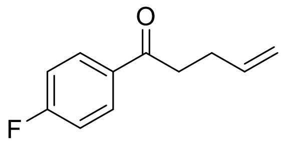

1-(4-Fluorophenyl)pent-4-en-1-one (**3b**) was prepared according to general procedure GP1 using 4-fluorobenzoic acid (1.68 g, 12.0 mmol, 1.00 eq.) carbonyldiimidazole (2.14 g, 13.2 mmol, 1.10 eq.), N,O-dimethylhydroxylamine hydrochloride (1.40 g, 14.4 mmol, 1.20 eq.), magnesium (321 mg, 13.2 mmol, 1.10 eq), 4-bromo-but-1-ene (1.33 mL, 13.2 mmol, 1.10 eq.), dichloromethane (12 mL) and tetrahydrofuran (22 mL). The product was isolated as a colourless liquid (1.50 g, 8.43 mmol, 70%) after flash column chromatography (pentane : diethyl ether = 98:2).

^{1}H-NMR (300 MHz, $CDCl_3$, 299 K): δ (ppm) = 8.12 – 7.87 (m, 2H), 7.19 – 7.01 (m, 2H), 5.90 (ddt, J = 16.9, 10.1, 6.5 Hz, 1H), 5.29 – 4.90 (m, 2H), 3.05 (t, J = 7.4 Hz, 2H), 2.67 – 2.33 (m, 2H).

^{13}C-NMR (75 MHz, $CDCl_3$, 299 K): δ (ppm) = 197.9, 165.8 (d, J = 254.5 Hz), 137.3, 133.5, 130.8 (d, J=9.3 Hz), 115.9, 115.6 (d, J =10.0 Hz), 37.8, 28.2.

^{19}F-NMR (282 MHz, CDCl3, 299 K): δ(ppm) = -105.5.

HRMS (ESI) *m/z*: $[M+Na]^+$ calculated for $C_{11}H_{11}FONa^+$: 201.0687; found: 201.0683.

1-(4-Chlorophenyl)pent-4-en-1-one (**3c**)

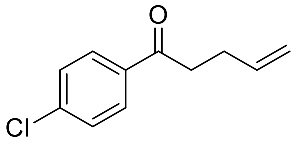

1-(4-Chlorophenyl)pent-4-en-1-one (**3c**) was prepared according to general procedure GP1 using 4-chlorobenzoic acid (2.35 g, 15.0 mmol, 1.00 eq.), carbonyldiimidazole (2.68 g, 16.5 mmol, 1.10 eq.), N,O-dimethylhydroxylamine hydrochloride (1.76 g, 18.0 mmol, 1.20 eq.), magnesium (397 mg, 16.3 mmol, 1.10 eq.), 4-bromo-but-1-ene (1.60 mL, 16.0 mmol, 1.10 eq.), dichloromethane (15 mL) and tetrahydrofuran (20 mL). The product was isolated as yellow liquid (1.92 g, 9.84 mmol, 66%) after flash column chromatography (pentane : diethyl ether = 95:5).

^{1}H-NMR (300 MHz, $CDCl_3$, 299 K): δ (ppm) = 7.93 – 7.85 (m, 2H), 7.46 – 7.38 (m, 2H), 5.88 (ddt, J = 16.8, 10.2, 6.5 Hz, 1H), 5.14 – 4.96 (m, 2H), 3.04 (t, J = 7.4 Hz, 2H), 2.55 – 2.41 (m, 2H).

^{13}C-NMR (75 MHz, $CDCl_3$, 299 K): δ (ppm) = 198.3, 139.6, 137.2, 135.4, 129.6, 129.0, 115.6, 37.8, 28.2.

HRMS (ESI) *m/z*: $[M+Na]^+$ calculated for $C_{11}H_{11}OClNa^+$: 217.0391; found 217.0391.

1-(4-Bromophenyl)pent-4-en-1-one (**3d**)

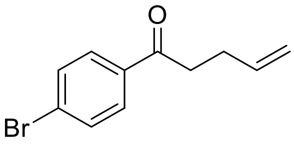

1-(4-Bromophenyl)pent-4-en-1-one (**3d**) was prepared according to the general procedure GP1 using 4-bromobenzoic acid (2.41 g, 12.0 mmol, 1.00 eq.), carbonyldiimidazole (2.14 g, 13.2 mmol, 1.10 eq.), N,O-dimethylhydroxylamine hydrochloride (1.40 g, 14.4 mmol, 1.20 eq.), magnesium (321 mg, 13.2 mmol, 1.10 eq), 4-bromo-but-1-ene (1.33 mL, 13.2 mmol, 1.10 eq.), dichloromethane (12 mL) and tetrahydrofuran (19 mL). The product was isolated as a colourless solid (1.51 g, 6.30 mmol, 53%) after flash column chromatography (pentane : diethyl ether = 98:2).

^{1}H-NMR (300 MHz, $CDCl_3$, 299 K): δ (ppm) = 7.88 – 7.77 (m, 2H), 7.69 – 7.54 (m, 2H), 5.88 (ddt, J = 16.8, 10.2, 6.5 Hz, 1H), 5.18 – 4.94 (m, 2H), 3.04 (t, J = 7.4 Hz, 2H), 2.60 – 2.40 (m, 2H).

^{13}C-NMR (75 MHz, $CDCl_3$, 299 K): δ (ppm) = 198.5, 137.2, 135.7, 132.0, 129.7, 128.3, 115.6, 37.8, 28.1.

HRMS (ESI) *m/z*: $[M+Na]^+$ calculated for $C_{11}H_{11}BrONa^+$: 260.9886; found: 260.9887.

1-(4-(Trifluoromethyl)phenyl)pent-4-en-1-one (**3e**)

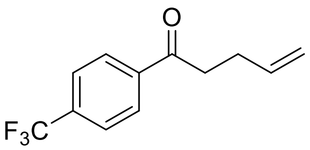

1-(4-(Trifluoromethyl)phenyl)pent-4-en-1-one (**3e**) was prepared according to general procedure GP1 using 4-(trifluoromethyl)benzoic acid (6.84 g, 36.0 mmol, 1.00 eq.) carbonyldiimidazole (6.42 g, 39.6 mmol, 1.10 eq.), N,O-dimethylhydroxylamine hydrochloride (3.80 g, 43.2 mmol, 1.20 eq.), magnesium (969 mg, 39.6 mmol, 1.10 eq), 4-bromo-but-1-ene (4.00 mL, 39.6 mmol, 1.10 eq.), dichloromethane (36 mL) and tetrahydrofuran (56 mL). The product was isolated as a yellowish liquid (3.01 g, 13.2 mmol, 37%) after flash column chromatography (pentane 100%).

^{1}H-NMR (300 MHz, $CDCl_3$, 299 K): δ (ppm) = 8.05 (d, J = 8.1 Hz, 2H), 7.72 (d, J = 8.2 Hz, 2H), 5.89 (ddt, J = 16.8, 10.2, 6.5 Hz, 1H), 5.16 – 4.97 (m, 2H), 3.10 (t, J = 7.3 Hz, 2H), 2.57 – 2.44 (m, 2H).

^{13}C-NMR (75 MHz, $CDCl_3$, 299 K): δ (ppm) = 198.5, 139.7, 137.0, 134.5 (d, J = 32.8 Hz) 128.5, 125.8 (q, J = 3.8 Hz), 123.7 (d, J = 272.6 Hz), 115.7, 38.2, 28.0.

^{19}F-NMR (282 MHz, CDCl3, 299 K): δ(ppm) = -63.1.

HRMS (ESI) *m/z*: $[M+Na]^+$ calculated for $C_{12}H_{11}F_3ONa^+$: 251.0655; found: 251.0651.

1-(*p*-Tolyl)pent-4-en-1-one (**3f**)

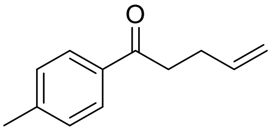

1-(*p*-Tolyl)pent-4-en-1-one (**3f**) was prepared according to general procedure GP1 using 4-methylbenzoic acid (1.63 g, 12.0 mmol, 1.00 eq.), carbonyldiimidazole (2.14 g, 13.2 mmol, 1.10 eq.), N,O-dimethylhydroxylamine hydrochloride (1.40 g, 14.4 mmol, 1.20 eq.), magnesium (321 mg, 13.2 mmol, 1.10 eq), 4-bromo-but-1-ene (1.33 mL, 13.2 mmol, 1.10 eq.), dichloromethane (12 mL) and tetrahydrofuran (22 mL). The product was isolated as a colorless liquid (1.62 g, 9.33 mmol, 78%) after flash column chromatography (pentane : diethyl ether = 95:5).

^{1}H-NMR (300 MHz, $CDCl_3$, 299 K): δ (ppm) = 7.84 (d, J = 8.2 Hz, 2H), 7.31 – 7.11 (m, 2H), 5.88 (ddt, J = 16.8, 10.2, 6.5 Hz, 1H), 5.11 – 4.90 (m, 2H), 3.02 (dd, J = 8.0, 6.9 Hz, 2H), 2.47 (dtt, J = 8.4, 6.8, 1.5 Hz, 2H), 2.39 (s, 3H).

^{13}C-NMR (75 MHz, $CDCl_3$, 299 K): δ (ppm) = 199.3, 143.9, 137.5, 134.6, 129.4, 128.3, 115.3, 37.7, 28.4, 21.8.

HRMS (ESI) *m/z*: $[M+Na]^+$ calculated for $C_{12}H_{14}ONa^+$: 197.0937; found: 197.0932.

1-(*m*-Tolyl)pent-4-en-1-one (**3g**)

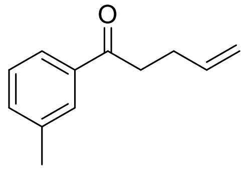

1-(*m*-Tolyl)pent-4-en-1-one (**3g**) was prepared according to general procedure GP1 using 3-methylbenzoic acid (1.64 g, 12.0 mmol, 1.00 eq.) carbonyldiimidazole (2.14 g, 13.2 mmol, 1.10 eq.), N,O-dimethylhydroxylamine hydrochloride (1.40 g, 14.4 mmol, 1.20 eq.), magnesium (321 mg, 13.2 mmol, 1.10 eq), 4-bromo-but-1-ene (1.33 mL, 13.2 mmol, 1.10 eq.), dichloromethane (12 mL) and tetrahydrofuran (22 mL). The product was isolated as a colorless liquid (1.15 g, 6.62 mmol, 55%) after flash column chromatography (pentane : diethyl ether = 95:5).

^{1}H-NMR (300 MHz, $CDCl_3$, 299 K): δ (ppm) = 7.81 – 7.70 (m, 2H), 7.42 – 7.30 (m, 2H), 5.91 (ddt, J = 16.8, 10.1, 6.5 Hz, 1H), 5.15 – 4.95 (m, 2H), 3.06 (dd, J = 8.0, 6.9 Hz, 2H), 2.49 (dtt, J = 8.4, 7.0, 1.4 Hz, 2H), 2.41 (s, 3H).

^{13}C-NMR (75 MHz, $CDCl_3$, 299 K): δ (ppm) = 199.8, 138.5, 137.5, 137.1, 133.9, 128.7, 128.6, 125.4, 115.4, 37.9, 28.3, 21.5.

HRMS (ESI) *m/z*: $[M+Na]^+$ calculated for $C_{12}H_{14}ONa^+$: 197.0937; found: 197.0936.

1-(Naphthalen-2-yl)pent-4-en-1-one (**3h**)

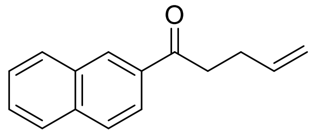

1-(Naphthalen-2-yl)pent-4-en-1-one (**3h**) was prepared according to general procedure GP1 using 2-naphthoic acid (2.58 g, 15 mmol, 1.00 eq.), carbonyldiimidazole (2.68 g, 16.5 mmol, 1.10 eq.), N,O-dimethylhydroxylamine hydrochloride (1.76 g, 18.0 mmol, 1.20 eq.), magnesium (401 mg, 16.5 mmol, 1.10 eq.), 4-bromo-but-1-ene (2.15 mL, 21.5 mmol, 1.43 eq.), dichloromethane (30 mL) and tetrahydrofuran (20 mL). The product was was isolated as a colorless liquid (2.09 g, 9.94 mmol, 66%) after flash column chromatography (pentane : diethyl ether = 97:3).

^{1}H-NMR (300 MHz, $CDCl_3$, 299 K): δ = 8.51 – 8.45 (m, 1H), 8.11 – 7.81 (m, 4H), 7.68 – 7.42 (m, 2H), 5.96 (ddt, J=16.8, 10.2, 6.5, 1H), 5.22 – 4.95 (m, 2H), 3.22 (dd, J=8.0, 6.9, 2H), 2.57 (dtt, J=8.3, 6.7, 1.4, 2H).

^{13}C-NMR (75 MHz, $CDCl_3$, 299 K): δ (ppm) = 199.5, 137.5, 135.7, 134.4, 132.7, 129.8, 129.7, 128.6, 128.5, 127.9, 126.9, 124.0, 115.5, 38.0, 28.5.

HRMS (ESI) *m/z*: $[M+Na]^+$ calculated for $C_{15}H_{14}ONa^+$: 233.0937; found: 233.0931

4-(Pent-4-enoyl)benzonitrile (**3i**)

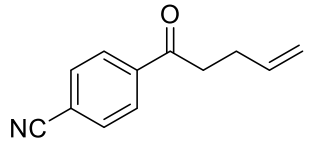

4-(Pent-4-enoyl)benzonitrile (**3i**) was prepared according to general procedure GP1 using 4-cyanobenzoic acid (2.94 g, 20.0 mmol, 1.00 eq.), carbonyldiimidazole (3.57 g, 22.0 mmol, 1.10 eq.), N,O-dimethylhydroxylamine hydrochloride (2.34 g, 24.0 mmol, 1.20 eq.),

magnesium (535 mg, 22.0 mmol, 1.10 eq.), 4-bromo-but-1-ene (2.3mL, 22.0 mmol, 1.10 eq.), dichloromethane (20 mL) and tetrahydrofuran (60 mL). The product was was isolated as a pale yellow solid (1.87 g, 10.0 mmol, 50%) after flash column chromatography (pentane : diethyl ether = 85:15).

^{1}H-NMR (300 MHz, $CDCl_3$, 299 K): δ (ppm) =8.10 – 7.98 (m, 2H), 7.81 – 7.71 (m, 2H), 5.87 (ddt, J = 16.8, 10.2, 6.5 Hz, 1H), 5.13 – 4.95 (m, 2H), 3.08 (t, J = 7.3 Hz, 2H), 2.56 – 2.42 (m, 2H).

^{13}C-NMR (75 MHz, $CDCl_3$, 299 K): δ (ppm) = 198.1, 139.9, 136.8, 132.6, 128.5, 118.1, 116.4, 115.9, 38.2, 27.9.

HRMS (ESI) *m/z*: $[M-H]^-$ calculated for $C_{12}H_{10}NO^-$: 184.0768; found 184.0767.

Ethyl 3-phenylhepta-2,6-dienoate (**4a**)

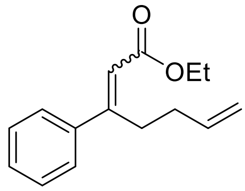

Ethyl-3-phenylhepta-2,6-dienoate (**4a**) was prepared according to general procedure GP2 using 1-phenylpent-4-en-1-one (**3a**) (1.44 g, 9.00 mmol, 1.00 eq.), ethyl(diethoxyphosphoryl) acetate (4.50 mL, 22.5 mmol, 2.50 eq.), KO^tBu (2.51 g, 22.5 mmol, 2.50 eq.) and tetrahydrofuran (22 mL). The diastereomers were separated after flash column chromatography (pentane : ethyl acetate = 98:2) as colorless liquid (1.82 g, 7.90 mmol, 88%, d.r = 5:4).

Major diastereomer (1.01 g, 4.38 mmol, 49%):

^{1}H-NMR (300 MHz, $CDCl_3$, 299 K): δ (ppm) = 7.40 – 7.28 (m, 3H), 7.20 – 7.07 (m, 2H), 5.89 (t, J = 1.3 Hz, 1H), 5.78 (ddt, J = 16.9, 10.3, 6.6 Hz, 1H), 5.07 – 4.94 (m, 2H), 3.98 (q, J = 7.1 Hz, 2H), 2.54 (ddd, J = 7.6, 6.7, 1.3 Hz, 2H), 2.15 (tdt, J = 7.8, 6.5, 1.4 Hz, 2H), 1.06 (t, J = 7.1 Hz, 3H).

^{13}C-NMR (75 MHz, $CDCl_3$, 299 K): δ (ppm) = 166.1, 158.8, 140.1, 137.2, 128.0, 127.8, 127.3, 117.8, 115.6, 60.0, 39.8, 31.5, 14.1.

HRMS (ESI) *m/z*: $[M+Na]^+$ calculated for $C_{15}H_{18}O_2Na^+$: 253.1199; found: 253.1198.

Minor diastereomer (0.81 g, 3.52 mmol, 39%):

^{1}H-NMR (300 MHz, $CDCl_3$, 299 K): δ (ppm) = 7.49 – 7.32 (m, 5H), 6.05 (s, 1H), 5.83 (ddt, J = 16.9, 10.2, 6.6 Hz, 1H), 5.05 – 4.90 (m, 2H), 4.21 (q, J = 7.1 Hz, 2H), 3.28 – 3.13 (m, 2H), 2.27 – 2.08 (m, 2H), 1.31 (t, J = 7.1 Hz, 3H).

^{13}C-NMR (75 MHz, $CDCl_3$, 299 K): δ (ppm) = 166.6, 159.9, 141.3, 137.9, 129.0, 128.7, 126.9, 118.0, 115.0, 60.0, 33.1, 30.4, 14.5.

HRMS (ESI) *m/z*: $[M+Na]^+$ calculated for $C_{15}H_{18}O_2Na^+$: 253.1199; found: 253.1198.

Ethyl 3-(4-fluorophenyl)hepta-2,6-dienoate (**4b**)

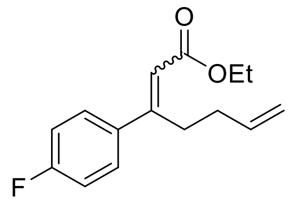

Ethyl 3-(4-fluorophenyl)hepta-2,6-dienoate (**4b**) was prepared according to general procedure. GP2 using 1-(4-fluorophenyl)pent-4-en-1-one (**3b**) (1.50 g, 8.42 mmol, 1.00 eq.), ethyl(diethoxyphosphoryl) acetate (4.21 mL, 21.0 mmol, 2.50 eq.), KO^{t}Bu (2.35 g, 21.0 mmol, 2.50 eq.) and tetrahydrofuran (25 mL). Two diastereomers of the product were obtained as a yellowish liquid (1.730 g, 6.96 mmol, 83%) after flash column chromatography (pentane : diethyl ether = 98:2).

Major isomer: (1.044 g, 4.20 mmol, 50%)

^{1}H-NMR (300 MHz, $CDCl_3$, 299 K): δ (ppm) = 7.20 – 7.10 (m, 2H), 7.08 – 6.99 (m, 2H), 5.89 (t, J = 1.2, 1H), 5.76 (ddt, J = 17.6, 9.7, 6.6, 1H), 5.07 – 4.93 (m, 2H), 4.00 (q, J = 7.1, 2H), 2.52 (td, J = 7.6, 1.3, 2H), 2.18 – 2.07 (m, 2H), 1.10 (t, J = 7.1, 3H).

^{13}C-NMR (75 MHz, $CDCl_3$, 299 K): δ (ppm) = 166.0, 162.5 (d, J = 246.5 Hz), 157.7, 137.0, 135.7, 129.1 (d, J = 8.3 Hz), 118.2, 115.8, 115.2, 114.9, 60.0, 39.8, 14.1.

^{19}F NMR (282 MHz, $CDCl_3$, 299 K) δ (ppm) = -114.4.

HRMS (ESI) *m/z*: $[M+Na]^+$ calculated for $C_{15}H_{17}FO_2Na^+$: 271.1105 found: 271.1101.

Minor isomer: (686 mg, 2.76 mmol, 33%)

^{1}H-NMR (300 MHz, $CDCl_3$, 299 K): δ (ppm) = 7.47 – 7.33 (m, 2H), 7.13 – 6.99 (m, 2H), 6.00 (s, 1H), 5.81 (ddt, J = 16.9, 10.2, 6.6, 1H), 5.04 – 4.89 (m, 2H), 4.21 (q, J = 7.1, 2H), 3.23 – 3.12 (m, 2H), 2.24 – 2.10 (m, 2H), 1.31 (t, J = 7.1, 3H).

^{13}C-NMR (75 MHz, $CDCl_3$, 299 K): δ (ppm) = 166.4, 163.3 (d, J = 248.8 Hz), 158.6, 137.7, 137.3 (d, J = 3.4 Hz), 128.7 (d, J = 8.0 Hz), 118.0, 115.8, 115.3 (d, J = 27.9 Hz), 60.1, 33.0, 30.5, 14.5.

^{19}F NMR (282 MHz, $CDCl_3$, 299 K) δ (ppm) = -112.7.

HRMS (ESI) *m/z*: $[M+Na]^+$ calculated for $C_{15}H_{17}FO_2Na^+$: 271.1105 found: 271.1104

Ethyl 3-(4-chlorophenyl)hepta-2,6-dienoate (**4c**)

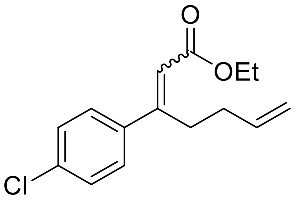

Ethyl 3-(4-chlorophenyl)hepta-2,6-dienoate (**4c**) was prepared according to general procedure GP2 using 1-(4-chlorophenyl)pent-4-en-1-one (**3c**) (1.56 g, 8.00 mmol, 1.00 eq.), ethyl(diethoxyphosphoryl) acetate (4.0 mL, 20 mmol, 2.5 eq.), KO^{t}Bu (2.24 g, 20.0 mmol, 2.50 eq.) and tetrahydrofuran (54 mL). The product was isolated after flash column chromatography (pentane : diethyl ether = 98:3) as a yellowish liquid (1.93 g, 7.89 mmol, 99%, d.r = 3:2). The diastereomers could not be separated.

^{1}H-NMR (300 MHz, $CDCl_3$, 299 K): δ (ppm) = 7.39 – 7.27 (m, 6H), 7.13 – 7.06 (m, 2H), 6.02 (s, 1H), 5.90 (s, 1H), 5.88 – 5.65 (m, 2H), 5.05 – 4.89 (m, 4H), 4.21 (q, J = 7.1 Hz, 2H), 4.00 (q, J =

7.1 Hz, 2H), 3.17 (t, 2H), 2.51 (ddd, J = 8.9, 6.3, 1.3 Hz, 2H), 2.23 – 2.03 (m, 4H), 1.31 (t, J = 7.1 Hz, 3H), 1.11 (t, J = 7.1 Hz, 3H).

^{13}C-NMR (75 MHz, $CDCl_3$, 299 K): δ (ppm) = 166.3, 165.8, 158.4, 157.5, 139.7, 138.4, 137.6, 136.9, 135.0, 133.7, 128.9, 128.8, 128.3, 128.2, 118.4, 118.4, 115.8, 115.2, 60.1, 60.1, 39.6, 33.0, 31.4, 30.3, 14.4, 14.1.

HRMS (ESI) *m/z*: $[M+Na]^+$ calculated for $C_{15}H_{17}O_2ClNa^+$ 287.0809; found 287.0805.

Ethyl 3-(4-bromophenyl)hepta-2,6-dienoate (**4d**)

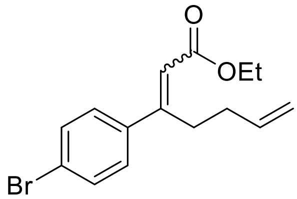

Ethyl 3-(4-bromophenyl)hepta-2,6-dienoate (**4d**) was prepared according to general procedure GP2 using 1-(4-bromophenyl)pent-4-en-1-one (**3d**) (2.39 g, 10.0 mmol, 1.00 eq.), ethyl(diethoxyphosphoryl) acetate (5.0 mL, 25 mmol, 2.5 eq.), KO^{t}Bu (2.81 g, 25.0 mmol, 2.50 eq.) and tetrahydrofuran (66 mL). The product was isolated *via* flash column chromatography (pentane : diethyl ether = 97:3) as a yellowish liquid (2.43 g, 7.87 mmol, 79%, d.r = 5:4). The diastereomers could not be separated.

^{1}H-NMR (300 MHz, $CDCl_3$, 299 K): δ (ppm) = 7.53 – 7.43 (m, 4H), 7.33 – 7.23 (m, 2H), 7.08 – 6.99 (m, 2H), 6.02 (s, 1H), 5.89 (t, J = 1.3 Hz, 1H), 5.87 – 5.67 (m, 2H), 5.06 – 4.88 (m, 4H), 4.21 (q, J = 7.2 Hz, 2H), 4.00 (q, J = 7.1 Hz, 2H), 3.23 – 3.09 (m, 2H), 2.57 – 2.42 (m, 2H) 2.23 – 2.05 (m, 4H), 1.31 (t, J = 7.1 Hz, 3H), 1.10 (t, J = 7.2 Hz, 3H).

^{13}C-NMR (75 MHz, $CDCl_3$, 299 K): δ (ppm) = 166.3, 165.8, 158.5, 157.5, 140.2, 138.9, 137.6, 136.9, 131.9, 131.2, 129.1, 128.5, 123.2, 121.9, 118.4, 118.4, 115.8, 115.2, 60.1, 60.1, 39.6, 33.0, 31.4, 30.2, 14.4, 14.1.

HRMS (ESI) *m/z*: $[M+Na]^+$ calculated for $C_{15}H_{17}O_2BrNa^+$: 331.0304; found: 331.0303.

Ethyl 3-(4-(trifluoromethyl)phenyl)hepta-2,6-dienoate (**4e**)

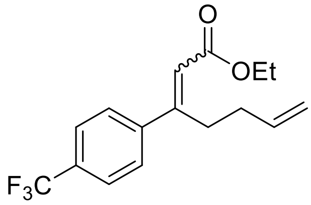

Ethyl 3-(4-(trifluoromethyl)phenyl)hepta-2,6-dienoate (**4e**) was prepared according to general procedure GP2 using 1-(4-trifluorophenyl)pent-4-en-1-one (**3e**) (1.60 g, 7.00 mmol, 1.00 eq.), ethyl(diethoxyphosphoryl) acetate (3.50 mL, 17.5 mmol, 2.50 eq.), KO^{t}Bu (1.96 g, 17.5 mmol, 2.50 eq.) and tetrahydrofuran (47 mL). The product was isolated after flash column chromatography (pentane : diethyl ether = 95:5) as a yellowish liquid (1.89 g, 6.32 mmol, 90%, d.r = 3:2).The diastereomers could not be separated.

^{1}H-NMR (300 MHz, $CDCl_3$, 299 K): δ (ppm) = 7.67 – 7.58 (m, 3H), 7.56 – 7.48 (m, 2H), 7.31 – 7.22 (m, 3H), 6.05 (s, 1H), 5.94 (t, J = 1.3 Hz, 1H), 5.88 – 5.68 (m, 2H), 5.08 – 4.91 (m, 4H), 4.23 (q, J = 7.1 Hz, 2H), 3.99 (q, J = 7.1 Hz, 2H), 3.20 (dd, J = 8.6, 6.9 Hz, 2H), 2.54 (ddd, J = 8.9, 6.3,

1.3 Hz, 2H), 2.16 (dddd, J = 12.3, 10.7, 5.1, 3.7 Hz, 4H), 1.32 (t, J = 7.1 Hz, 3H), 1.07 (t, J = 7.1 Hz, 3H).

^{13}C-NMR (75 MHz, $CDCl_3$, 299 K): δ (ppm) = 166.0, 165.5, 158.1, 157.3, 144.9, 143.9, 137.3, 136.7, 130.9 (d, J = 32.5 Hz), 129.8 (d, J = 32.5 Hz), 127.6, 127.1, 126.1 (d, J = 271.4 Hz), 125.5 (q, J = 3.8 Hz), 124.9 (q, J = 3.8 Hz), 122.30 (d, J = 271.4 Hz), 119.6, 118.7, 115.9, 115.3, 60.2, 60.0, 39.5, 32.7, 31.2, 30.3, 14.3, 13.9.

^{19}F-NMR (282 MHz, CDCl3, 299 K): δ(ppm) = -62.5, -62.7.

HRMS (ESI) *m/z*: $[M+Na]^+$ calculated for $C_{16}H_{17}O_2F_3Na^+$; found 283.1305.

Ethyl 3-(*p*-tolyl)hepta-2,6-dienoate (4f)

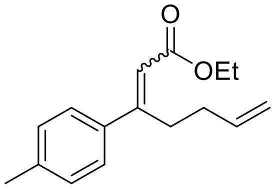

Ethyl 3-(*p*-tolyl)hepta-2,6-dienoate (**4f**) was prepared according to general procedure GP2 1-(*p*-tolyl)pent-4-en-1-one (**3f**) (800 mg, 4.59 mmol, 1.00 eq.), ethyl(diethoxyphosphoryl) acetate (2.29 mL, 11.5 mmol, 2.50 eq.), KO^tBu (1.29 g, 11.5 mmol, 2.50 eq.) and tetrahydrofuran (20 mL). Two diastereomers of the product were obtained as yellowish liquids (966 mg, 3.95 mmol, 86%) after flash column chromatography (pentane : diethyl ether = 98:2 to 90:10).

Major isomer (556 mg, 2.28 mmol, 50%):

^{1}H-NMR (300 MHz, $CDCl_3$, 299 K): δ = 7.16 (d, J = 7.9, 2H), 7.06 (d, J = 8.1, 2H), 5.87 (d, J = 1.3, 1H), 5.77 (ddt, J = 16.9, 10.2, 6.6, 1H), 5.07 – 4.93 (m, 2H), 4.00 (q, J = 7.1, 2H), 2.58 – 2.47 (m, 2H), 2.36 (s, 3H), 2.19 – 2.09 (m, 2H), 1.10 (t, J = 7.1, 3H).

^{13}C-NMR (75 MHz, $CDCl_3$, 299 K): δ (ppm) = 166.2, 159.0, 137.6, 137.3, 136.9, 128.7, 127.3, 117.5, 115.5, 59.9, 39.8, 31.6, 21.4, 14.1.

HRMS (ESI) *m/z*: $[M+Na]^+$ calculated for $C_{16}H_{20}O_2Na^+$: 267.1356, found: 267.1353

Minor isomer (410 mg, 1.68 mmol, 37%):

^{1}H-NMR (300 MHz, $CDCl_3$, 299 K): δ (ppm) = 7.34 (d, J = 8.2, 2H), 7.18 (d, J = 8.0, 2H), 6.04 (s, 1H), 5.84 (ddt, J = 16.8, 10.0, 6.6, 1H), 5.04 – 4.91 (m, 2H), 4.21 (q, J = 7.2, 2H), 3.23 – 3.15 (m, 2H), 2.37 (s, 3H), 2.19 (q, J = 7.3, 2H), 1.31 (t, J = 7.1, 3H).

^{13}C-NMR (75 MHz, $CDCl_3$, 299 K): δ (ppm) = 166.7, 159.8, 139.2, 138.3, 138.0, 129.4, 126.8, 117.1, 114.9, 59.9, 33.2, 30.3, 21.3, 14.5.

HRMS (ESI) *m/z*: $[M+Na]^+$ calculated for $C_{16}H_{20}O_2Na^+$: 267.1356, found: 267.1353

Ethyl 3-(*m*-tolyl)hepta-2,6-dienoate (4g)

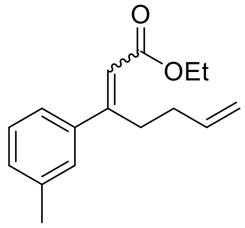

Ethyl 3-(*m*-tolyl)hepta-2,6-dienoate (**4g**) was prepared according to general procedure GP2 using 1-(*m*-tolyl)pent-4-en-1-one (**3g**) (1.05 g, 6.00 mmol, 1.00 eq.), ethyl(diethoxyphosphoryl) acetate (3.0 mL, 15 mmol, 2.5 eq.), KO^{t}Bu (1.68 g, 15.0 mmol, 2.50 eq.) and tetrahydrofuran (40 mL). The diastereomers were separated after flash column chromatography (pentane : diethyl ether = 98:2) as colorless liquid (1.31 g, 5.36 mmol, 89%, d.r = 6:5).

Major isomer (713 mg, 2.92 mmol, 49%):

^{1}H-NMR (300 MHz, $CDCl_3$, 299 K): δ (ppm) = 7.22 (d, J = 8.0 Hz, 1H), 7.12 (d, J = 7.6 Hz, 1H), 7.00 – 6.92 (m, 2H), 5.87 (s, 1H), 5.84 – 5.70 (m, 1H), 5.06 – 4.95 (m, 2H), 3.99 (q, J = 7.1 Hz, 2H), 2.58 – 2.48 (m, 2H), 2.36 (s, 3H), 2.22 – 2.09 (m, 2H), 1.07 (t, J = 7.1 Hz, 3H).

^{13}C-NMR (75 MHz, $CDCl_3$, 299 K): δ (ppm) = 166.2, 158.8, 140.1, 137.5, 137.3, 128.5, 127.9, 127.8, 124.5, 117.7, 115.5, 59.9, 39.8, 31.6, 21.6, 14.1.

HRMS (ESI): *m/z*: $[M+Na]^+$ calculated for $C_{16}H_{20}O_2Na^+$ 267.1356; found 267.1356.

Minor isomer (597 mg, 2.44 mmol, 41%):

^{1}H-NMR (300 MHz, $CDCl_3$, 299 K): δ (ppm) = 7.32 – 7.14 (m, 4H), 6.04 (s, 1H), 5.84 (ddt, J = 16.9, 10.0, 6.7 Hz, 1H), 5.07 – 4.89 (m, 2H), 4.22 (q, 2H), 3.19 (t, J = 7.9 Hz, 2H), 2.38 (s, 3H), 2.19 (q, 2H), 1.32 (t, J = 1.1 Hz, 3H).

^{13}C-NMR (75 MHz, $CDCl_3$, 299 K): δ (ppm) = 166.6, 160.1, 141.4, 138.3, 138.0, 129.8, 128.6, 127.6, 124.0, 117.8, 115.0, 60.0, 33.1, 30.5, 21.6, 14.5.

HRMS (ESI): $[M+Na]^+$ calculated for $C_{16}H_{20}O_2Na^+$ 267.1356; found 267.1354.

Ethyl 3-(naphthalen-2-yl)hepta-2,6-dienoate (4h)

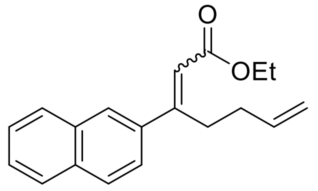

Ethyl 3-(naphthalen-2-yl)hepta-2,6-dienoate (**4h**) was prepared according to general procedure GP2 1-(naphthalen-2-yl)pent-4-en-1-one (**3h**) (1.89 g, 9.00 mmol, 1.00 eq.), ethyl(diethoxyphosphoryl) acetate (4.50 mL, 22.5 mmol, 2.50 eq.), KO^{t}Bu (2.52 g, 22.5 mmol, 2.50 eq.) and tetrahydrofuran (50 mL). Two diastereomers of the product were obtained as yellowish liquids (2.17 g, 7.74 mmol, 86%) after flash column chromatography (pentane : diethyl ether = 97:3).

Major isomer (1.208 g, 4.30 mmol, 48%):

^{1}H-NMR (300 MHz, $CDCl_3$, 299 K): δ (ppm) = 7.88 – 7.77 (m, 3H), 7.63 (t, J = 1.2, 1H), 7.52 – 7.43 (m, 2H), 7.34 – 7.24 (m, 1H), 5.98 (t, J = 1.2, 1H), 5.80 (ddt, J = 17.1, 10.5, 6.6, 1H), 5.06 – 4.96 (m, 2H), 3.97 (q, J = 7.1, 2H), 2.69 – 2.59 (m, 2H), 2.18 (tdt, J = 7.9, 6.6, 1.4, 2H), 1.01 (t, J = 7.1, 3H).

^{13}C-NMR (75 MHz, $CDCl_3$, 299 K): δ (ppm) = 166.1, 158.6, 137.6, 137.2, 133.1, 133.0, 128.2, 127.9, 127.5, 126.2, 126.1, 126.1, 125.9, 118.2, 115.7, 60.0, 39.9, 31.7, 14.1.

HRMS (ESI) *m/z*: $[M+Na]^+$ calculated for $C_{19}H_{20}O_2Na^+$: 303.1356, found: 303.1355.

Minor isomer (961 mg, 3.43 mmol, 38%):

^{1}H-NMR (300 MHz, $CDCl_3$, 299 K): δ (ppm) = 7.93 – 7.80 (m, 4H), 7.61 – 7.45 (m, 3H), 6.19 (s, 1H), 5.87 (ddt, *J*=16.8, 10.1, 6.6, 1H), 5.06 – 4.91 (m, 2H), 4.25 (q, *J*=7.1, 2H), 3.38 – 3.27 (m, 2H), 2.31 – 2.17 (m, 2H), 1.34 (t, *J*=7.1, 3H).

^{13}C-NMR (75 MHz, $CDCl_3$, 299 K): δ (ppm) = (76 MHz, $CDCl_3$) δ = 166.6, 159.7, 138.6, 137.9, 133.6, 133.3, 128.6, 128.4, 127.7, 126.8, 126.6, 126.3, 124.6, 118.4, 115.1, 60.1, 33.2, 30.4, 14.5.

HRMS (ESI) *m/z*: $[M+Na]^+$ calculated for $C_{19}H_{20}O_2Na^+$: 303.1356, found: 303.1357.

Ethyl 3-(4-cyanophenyl)hepta-2,6-dienoate (**4i**)

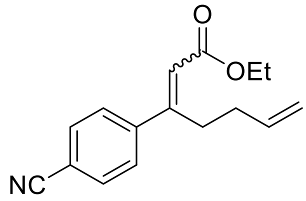

Ethyl 3-(4-cyanophenyl)hepta-2,6-dienoate (**4i**) was prepared according to general procedure GP2 4-(Pent-4-enoyl)benzonitrile (**3i**) (1.48 g, 8.00 mmol, 1.00 eq.), ethyl(diethoxyphosphoryl) acetate (4.00 mL, 20.0 mmol, 2.50 eq.), KO^{t}Bu (2.24 g, 20.0 mmol, 2.50 eq.) and tetrahydrofuran (54 mL). The minor diastereomer was isolated as a colorless oil (644 mg, 2.50 mmol, 32%) *via* flash column chromatography (pentane : diethyl ether = 80:20). The major diastereomer could not be purified and was used with impurity in further transformations.

Minor isomer (644 mg, 2.50 mmol, 32%)

^{1}H-NMR (300 MHz, $CDCl_3$, 299 K): δ (ppm) = 7.72 – 7.61 (m, 2H), 7.56 – 7.45 (m, 2H), 6.04 (s, 1H), 5.77 (ddt, J = 17.0, 10.4, 6.6 Hz, 1H), 5.02 – 4.89 (m, 2H), 4.22 (q, J = 7.1 Hz, 2H), 3.23 – 3.12 (m, 2H), 2.22 – 2.08 (m, 2H), 1.31 (t, J = 7.1 Hz, 3H).

^{13}C-NMR (75 MHz, $CDCl_3$, 299 K): δ (ppm) = 165.9, 157.5, 146.0, 137.2, 132.51, 127.6, 120.4, 118.6, 115.5, 112.6, 60.4, 32.8, 30.2, 14.4.

HRMS (ESI) *m/z*: $[M+Na]^+$ calculated for $C_{16}H_{17}O_2Na^+$: 278.1152; found: 278.1151.

***(E)*-4-Phenylocta-3,7-dien-2-one** (**1a**)

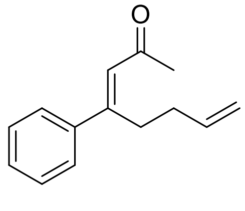

(E)-4-Phenylocta-3,7-dien-2-one (**1a**) was prepared according to GP3 using the major diastereomer of ethyl-3-phenylhepta-2,6-dienoate (**4a**) (2.30 g, 10.0 mmol, 1.00 eq.), N,O-dimethylhydroxylamine hydrochloride (1.95 g, 20.0 mmol, 2.00 eq.), isopropylmagnesium chloride (2.0 M in diethyl ether, 22 mL, 43 mmol, 4.3 eq.), methylmagnesium bromide (3.0 M in diethyl ether, 4.5 mL, 13 mmol, 1.3 eq.) and tetrahydrofuran (52 mL). The product was

isolated as a yellowish liquid (902 mg, 4.50 mmol, 45%) after flash column chromatography (pentane : diethyl ether = 90:10).

^{1}H-NMR (300 MHz, $CDCl_3$, 299 K): δ (ppm) = 7.48 – 7.33 (m, 5H), 6.42 (s, 1H), 5.82 (ddt, J = 16.9, 10.1, 6.6 Hz, 1H), 5.03 – 4.88 (m, 2H), 3.21 – 3.07 (m, 2H), 2.28 (s, 3H), 2.17 (dtt, J = 7.9, 6.6, 1.4 Hz, 2H).

^{13}C-NMR (75 MHz, $CDCl_3$, 299 K): δ (ppm) = 198.5, 158.2, 141.6, 137.9, 129.1, 128.7, 127.0, 125.2, 115.0, 33.1, 32.4, 30.6.

HRMS (ESI) *m/z*: $[M+Na]^+$ calculated for $C_{14}H_{16}ONa^+$ 223.1093; found 223.1093.

(Z)-4-Phenylocta-3,7-dien-2-one (1a)

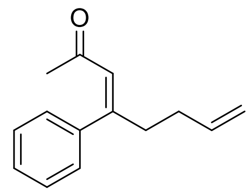

(Z)-4-Phenylocta-3,7-dien-2-one (**1a**) was prepared according to GP3 using the minor diasteromer of ethyl 3-phenylhepta-2,6-dienoate (**4a**) (1.15 g, 5.00 mmol, 1.00 eq.), N,O-dimethylhydroxylamine hydrochloride (975 mg, 10.0 mmol, 2.00 eq.), isopropylmagnesium chloride (2.0 M in diethyl ether, 11.0 mL, 21.5 mmol, 4.30 eq.), methylmagnesium bromide (3.0 M in diethyl ether, 2.50 mL, 7.50 mmol, 1.30 eq.) and tetrahydrofuran (33 mL). The product was isolated as a yellowish liquid (610 mg, 3.05 mmol, 61%) after flash column chromatography (pentane : diethylether = 90:10).

^{1}H-NMR (300 MHz, $CDCl_3$, 299 K): δ (ppm) = 7.42 – 7.31 (m, 3H), 7.20 – 7.13 (m, 2H), 6.09 (t, J = 1.2 Hz, 1H), 5.77 (ddt, J = 17.0, 10.4, 6.5 Hz, 1H), 5.05 – 4.95 (m, 2H), 2.60 – 2.48 (m, 2H), 2.14 (tdt, J = 7.9, 6.6, 1.5 Hz, 2H), 1.77 (s, 3H).

^{13}C-NMR (151 MHz, $CDCl_3$, 299 K): δ (ppm) = 200.6, 156.1, 140.1, 137.2, 128.6, 128.6, 128.4, 127.7, 115.7, 39.9, 31.7, 30.4.

HRMS (ESI) *m/z*: $[M+Na]^+$ calculated for $C_{14}H_{16}ONa^+$: 223.1093; found: 223.1091.

4-(4-Fluorophenyl)octa-3,7-dien-2-one (1b)

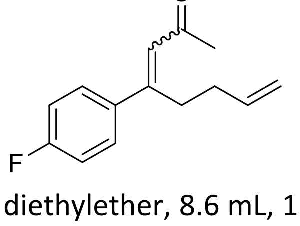

(*E*)-4-(4-Fluorophenyl)octa-3,7-dien-2-one and (*Z*)-4-(4-fluorophenyl)octa-3,7-dien-2-one (**1b**) were prepared according to GP3 using ethyl 3-(4-fluorophenyl)hepta-2,6-dienoate (**4b**) (993 mg, 4.00 mmol, 1.00 eq.), N,O-dimethylhydroxylamine hydrochloride (780 mg, 8.0 mmol, 2.00 eq.), isopropylmagnesium chloride (2.0 M in diethylether, 8.6 mL, 17 mmol, 4.30 eq.), methylmagnesium bromide (3.0 M in diethyl ether, 1.7 mL, 5.2 mmol, 1.3 eq.) and tetrahydrofuran (30 mL). The isomers were separated by FC (pentane : diethyl ether = 96:4 to 90:10). (*E*)-4-(4-fluorophenyl)octa-3,7-dien-2-one (236 mg, 1.08 mmol, 27%) and (*Z*)-4-(4-fluorophenyl)octa-3,7-dien-2-one (**1b**) (444 mg, 2.03 mmol, 51%) were each isolated as a yellow liquid.

(*E*)-4-(4-fluorophenyl)octa-3,7-dien-2-one (236 mg, 1.08 mmol, 27%):

^{1}H-NMR (300 MHz, $CDCl_3$, 299 K): δ (ppm) = 7.48 – 7.34 (m, 2H), 7.11 – 7.02 (m, 2H), 6.38 (s, 1H), 5.80 (ddt, J = 16.9, 10.2, 6.6, 1H), 5.02 – 4.88 (m, 2H), 3.18 – 3.03 (m, 2H), 2.28 (s, 3H), 2.15 (dtt, J = 9.2 , 6.6, 1.4 Hz, 2H).

^{13}C-NMR (75 MHz, $CDCl_3$, 299 K): δ (ppm) = 198.4, 163.4 (d, *J* = 249.1 Hz), 157.0, 137.8, 137.5 (d, *J* = 3.5 Hz), 128.8 (d, *J* = 8.3 Hz), 125.1 (d, *J*=0.9), 115.8, 115.3 (d, *J* = 34.3 Hz), 33.1, 32.4, 30.5.

^{19}F-NMR (282 MHz, CDCl3, 299 K): δ(ppm) = -112.4.

HRMS (ESI) *m/z*: $[M+Na]^+$ calculated for $C_{14}H_{15}OFNa^+$: 241.1000; found: 241.0998

(*Z*)-4-(4-fluorophenyl)octa-3,7-dien-2-one (444 mg, 2.03 mmol, 51%):

^{1}H NMR (300 MHz, $CDCl_3$) δ = 7.21 – 7.00 (m, 4H), 6.12 (t, J = 1.2 Hz, 1H), 5.76 (ddt, J=17.6, 9.7, 6.5, 1H), 5.06 – 4.93 (m, 2H), 2.58 – 2.46 (m, 2H), 2.13 (dtt, J = 8.0, 6.6, 1.4 Hz, 2H), 1.84 (s, 3H).

^{13}C-NMR (75 MHz, $CDCl_3$, 299 K): δ (ppm) = 200.1, 162.8 (d, *J* = 247.8 Hz) 154.9, 137.0, 135.8 (d, *J* = 3.5 Hz), 129.5 (d, *J* = 8.0 Hz), 128.5, 115.8, 115.6 (d, *J* = 21.5 Hz), 39.8, 31.7, 30.7.

^{19}F-NMR (282 MHz, CDCl3, 299 K): δ(ppm) = -113.3.

HRMS (ESI) *m/z*: $[M+Na]^+$ calculated for $C_{14}H_{15}OFNa^+$: 241.1000; found: 241.0999

4-(4-Chlorophenyl)octa-3,7-dien-2-one (**1c**)

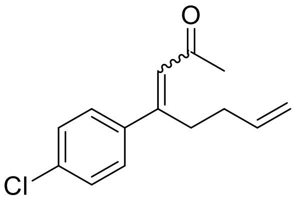

(E)-4-(4-Chlorophenyl)octa-3,7-dien-2-one and *(Z)*-4-(4-chlorophenyl) octa-3,7-dien-2-one (**1c**) were prepared according to GP3 using ethyl 3-(4-chlorophenyl)hepta-2,6-dienoate (**4c**) (1.58 g, 6.00 mmol, 1.00 eq.) N,O-dimethylhydroxylamine hydrochloride (1.12 g, 12.0 mmol, 2.00 eq.), isopropylmagnesium chloride (2.0 M in diethyl ether, 13.0 mL, 25.8 mmol, 4.30 eq.), methylmagnesium bromide (3.0 M in diethyl ether, 2.6 mL, 7.5 mmol, 1.30 eq.) and tetrahydrofuran (32 mL). Both isomers were separated *via* flash column chromatography (pentane : diethyl ether = 95:5). *(E)*-4-(4-chlorophenyl)octa-3,7-dien-2-one 291 mg, 1.24 mmol, 21%) and *(Z)*-4-(4-chlorophenyl)octa-3,7-dien-2-one (342 mg, 1.45 mmol, 24%) (**1c**) were each isolated as a yellow liquid.

(E)-4-(4-chlorophenyl)octa-3,7-dien-2-one (291 mg, 1.24 mmol, 21%)

^{1}H-NMR (300 MHz, $CDCl_3$, 299 K): δ (ppm) = 7.35 (t, J = 1.0 Hz, 4H), 6.39 (s, 1H), 5.79 (ddtd, J = 16.9, 10.2, 6.6, 0.9 Hz, 1H), 5.03 – 4.88 (m, 2H), 3.15 – 3.05 (m, 2H), 2.28 (d, J = 1.0 Hz, 3H), 2.14 (dtt, J = 9.1, 6.6, 1.3 Hz, 2H).

^{13}C-NMR (75 MHz, $CDCl_3$, 299 K): δ (ppm) = 198.4, 156.7, 139.9, 137.7, 135.1, 128.9, 128.3, 125.4, 115.2, 33.0, 32.4, 30.4.

HRMS (ESI) *m/z*: $[M+Na]^+$ calculated for $C_{14}H_{15}OClNa^+$ 257.0704; found 257.0705.

(Z)-4-(4-chlorophenyl)octa-3,7-dien-2-one (342 mg, 1.45 mmol, 24%)

¹H-NMR (300 MHz, $CDCl_3$, 299 K): δ (ppm) = 7.38 – 7.31 (m, 2H), 7.15 – 7.04 (m, 2H), 6.12 (s, 1H), 5.83 – 5.66 (m, 1H), 5.11 – 4.82 (m, 2H), 2.56 – 2.45 (m, 2H), 2.17 – 2.05 (m, 2H), 1.87 (s, 3H).

¹³C-NMR (75 MHz, $CDCl_3$, 299 K): δ (ppm) = 199.7, 154.6, 138.4, 136.9, 134.3, 129.1, 128.8, 128.3, 115.9, 39.7, 31.6, 30.8.

HRMS (ESI) *m/z*: $[M+Na]^+$ calculated for $C_{14}H_{15}OClNa^+$ 257.0704; found 257.0704.

4-(4-Bromophenyl)octa-3,7-dien-2-one (**1d**)

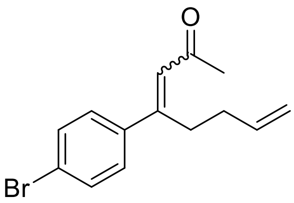

(E)-4-(4-Bromophenyl)octa-3,7-dien-2-one and *(Z)*-4-(4-bromophenyl)octa-3,7-dien-2-one (**1d**) were prepared according to GP3 using Ethyl 3-(4-bromophenyl)hepta-2,6-dienoate (**4d**) (1.55 g, 5.00 mmol, 1.00 eq.), N,O-dimethylhydroxylamine hydrochloride (975 mg, 10.0 mmol, 2.00 eq.), isopropylmagnesium chloride (2.00 M in diethyl ether, 10.8 mL, 21.5 mmol, 4.30 eq.), methylmagnesium bromide (3.0 M in diethyl ether, 2.5 mL, 7.5 mmol, 1.3 eq.) and tetrahydrofuran (32 mL). Both isomers were separated *via* flash column chromatography (pentane : diethyl ether = 93:7). *(E)*-4-(4-bromophenyl)octa-3,7-dien-2-one (413 mg, 1.48 mmol, 30%) and *(Z)*-4-(4-bromophenyl)octa-3,7-dien-2-one (527 mg, 1.89 mmol, 38%) (**1d**) were each isolated as a yellow liquid.

(E)-4-(4-bromophenyl)octa-3,7-dien-2-one (413 mg, 1.48 mmol, 30%):

¹H-NMR (300 MHz, $CDCl_3$, 299 K): δ (ppm) = 7.53 – 7.47 (m, 2H), 7.32 – 7.26 (m, 2H), 6.39 (t,J = 1.2 Hz, 1H), 5.79 (ddt, J = 16.9, 10.2, 6.7 Hz, 1H), 5.01 – 4.89 (m, 2H), 3.10 (dd, J = 8.5, 7.0 Hz, 2H), 2.27 (s, 3H), 2.14 (dtt, J = 8.0, 6.7, 1.4 Hz, 2H).

¹³C-NMR (75 MHz, $CDCl_3$, 299 K): δ (ppm) = 198.4, 156.8, 140.4, 137.7, 131.9, 128.6, 125.5, 123.3, 115.2, 33.0, 32.4, 30.3.

HRMS (ESI) *m/z*: $[M+Na]^+$ calculated for $C_{14}H_{15}OBrNa^+$: 301.0199; found: 301.0199.

(Z)-4-(4-bromophenyl)octa-3,7-dien-2-one (527 mg, 1.89 mmol, 38%):

¹H-NMR (300 MHz, $CDCl_3$, 299 K): δ (ppm) = 7.55 – 7.46 (m, 2H), 7.07 – 7.00 (m, 2H), 6.12 (s, 1H), 5.74 (ddt, J = 17.5, 9.7, 6.6 Hz, 1H), 4.99 (ddq, J = 15.1, 3.4, 1.7 Hz, 2H), 2.50 (ddd, J = 8.8, 6.3, 1.2 Hz, 2H), 2.12 (tdt, J = 8.3, 6.9, 1.5 Hz, 2H), 1.87 (s, 3H).

¹³C-NMR (75 MHz, $CDCl_3$, 299 K): δ (ppm) = 199.6, 154.6, 138.9, 136.9, 131.7, 129.4, 128.3, 122.5, 115.9, 39.6, 31.6, 30.8.

HRMS (ESI) *m/z*: $[M+Na]^+$ calculated for $C_{14}H_{15}OBrNa^+$ 301.0199; found 301.0200.

4-(4-(Trifluoromethyl)phenyl)octa-3,7-dien-2-one (**1e**)

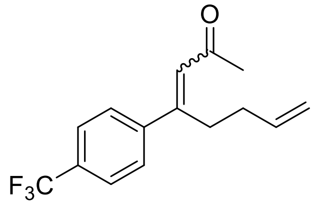

(E)-4-(4-(Trifluoromethyl)phenyl)octa-3,7-dien-2-one and *(Z)*-4-(4-(trifluoromethyl)phenyl)octa-3,7-dien-2-one (**1e**) were prepared according to GP3 using Ethyl 3-(4-(trifluoromethyl)phenyl)hepta-2,6-dienoate (**4e**) (1.49 g, 5.00 mmol, 1.00 eq.), N,O-dimethylhydroxylamine hydrochloride (975 mg, 10.0 mmol, 2.00 eq.), isopropylmagnesium chloride (2.0 M in diethyl ether, 10.8 mL, 21.5 mmol, 4.30 eq.), methylmagnesium bromide (3.0 M in diethyl ether, 2.5 mL, 7.5 mmol, 1.3 eq.) and tetrahydrofuran (32 mL). The isomers were separated by flash column chromatography (Pentane : diethyl ether = 90:10). *(E)*- 4-(4-(trifluoromethyl)phenyl)octa-3,7-dien-2-one was isolated as a yellow liquid (234 mg, 0.87 mmol, 17%) and *(Z)*- 4-(4-(trifluoromethyl)phenyl)octa-3,7-dien-2-one (**1e**) was isolated as a yellow liquid (229.6 mg, 0.86 mmol, 17%).

(E)- 4-(4-(trifluoromethyl)phenyl)octa-3,7-dien-2-one (234 mg, 0.87 mmol, 17%)

^{1}H-NMR (300 MHz, $CDCl_3$, 299 K): δ (ppm) =7.69 – 7.59 (m, 2H), 7.55 – 7.48 (m, 2H), 6.41 (s, 1H), 5.79 (ddt, J = 16.9, 10.2, 6.7 Hz, 1H), 5.03 – 4.89 (m, 2H), 3.13 (dd, J = 8.4, 7.0 Hz, 2H), 2.30 (s, 3H), 2.14 (dddt, J = 9.2, 8.0, 6.6, 1.4 Hz, 2H).

^{13}C-NMR (75 MHz, $CDCl_3$, 299 K): δ (ppm) = 198.5, 156.5, 145.3, 137.5, 130.9 (d, J = 32.6 Hz), 127.4, 126.6, 125.7 (q, J = 3.8 Hz), 124.6 (d, J = 272.0 Hz), 115.3, 32.9, 32.4, 30.5.

^{19}F-NMR (282 MHz, CDCl3, 299 K): δ(ppm) = -62.7.

HRMS (ESI) *m/z*: $[M+Na]^+$ calculated for $C_{15}H_{15}OF_3Na^+$ 291.0967; found 291.0967.

(Z)- 4-(4-(trifluoromethyl)phenyl)octa-3,7-dien-2-one (229.6 mg, 0.86 mmol, 17%)

^{1}H-NMR (300 MHz, $CDCl_3$, 299 K): δ (ppm) = 7.62 (d, J = 8.0 Hz, 2H), 7.31 – 7.23 (m, 2H), 6.19 (t, J = 1.2 Hz, 1H), 5.84 – 5.65 (m, 1H), 5.09 – 4.93 (m, 2H), 2.53 (ddd, J = 8.8, 6.4, 1.2 Hz, 2H), 2.20 – 2.07 (m, 2H), 1.91 (s, 3H).

^{13}C-NMR (75 MHz, $CDCl_3$, 299 K): δ (ppm) = 198.9, 154.4, 144.0, 136.8, 130.2 (d, J = 32.6 Hz), 128.1, 128.0, 125.5 (q, J = 3.8 Hz), 125.1 (d, J = 271.9 Hz), 116.0, 39.7, 31.4, 30.9.

^{19}F-NMR (282 MHz, CDCl3, 299 K): δ(ppm) = -62.6.

HRMS (ESI) *m/z*: $[M+Na]^+$ calculated for $C_{15}H_{15}OF_3Na^+$: 291.0967; found: 291.0966.

4-(*p*-Tolyl)octa-3,7-dien-2-one (**1f**)

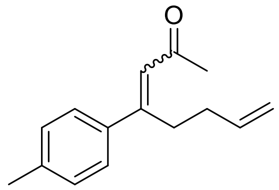

(E)-4-(*p*-Tolyl)octa-3,7-dien-2-one and (Z)-4-(*p*-tolyl)octa-3,7-dien-2-one (**1f**) were prepared according to GP3 using Ethyl 3-(*p*-tolyl)hepta-2,6-dienoate (**4f**) (905 mg, 3.70 mmol, 1.00 eq.), N,O-dimethylhydroxylamine hydrochloride (723 mg, 7.41 mmol, 2.00 eq.), isopropylmagnesium chloride (2.0 M in diethyl ether, 8.0 mL, 16 mmol, 4.30 eq.), methylmagnesium bromide (3.0 M in diethyl ether, 2.0 mL, 5.6 mmol, 1.5 eq.) and

tetrahydrofuran (24 mL). The isomers were separated by flash column chromatography (pentane : diethyl ether = 90:10). (*E*)-4-(*p*-tolyl)octa-3,7-dien-2-one (287 mg, 1.34 mmol, 36%) and (*Z*)-4-(*p*-tolyl)octa-3,7-dien-2-one (282 mg, 1.31 mmol , 36%) (**1f**) were each isolated as a yellow liquid.

(*E*)-4-(*p*-tolyl)octa-3,7-dien-2-one (287 mg, 1.34 mmol, 36%):

^{1}H-NMR (300 MHz, $CDCl_3$, 299 K): δ (ppm) =7.35 (d, J = 7.9, 2H), 7.19 (d, J = 7.9, 2H), 6.42 (s, 1H), 5.92 – 5.73 (m, 1H), 5.05 – 4.83 (m, 2H), 3.13 (t, J = 7.8, 2H), 2.38 (s, 3H), 2.27 (s, 3H), 2.16 (tdt, J = 8.0, 6.5, 1.4 Hz, 2H).

^{13}C-NMR (75 MHz, $CDCl_3$, 299 K): δ (ppm) = 198.6, 158.2, 139.4, 138.5, 138.1, 129.5, 126.9, 124.4, 114.9, 33.2, 32.5, 30.4, 21.4.

HRMS (ESI) *m/z*: $[M+Na]^+$ calculated for $C_{15}H_{18}ONa^+$: 237.1250 found: 237.1245.

(*Z*)-4-(*p*-tolyl)octa-3,7-dien-2-one (282 mg, 1.31 mmol , 36%):

^{1}H-NMR (300 MHz, $CDCl_3$, 299 K): δ (ppm) = 7.18 (d, J = 7.8, 2H), 7.06 (d, J = 7.7, 2H), 6.07 (t, J = 1.2 Hz, 1H), 5.76 (ddt, J = 12.9, 10.0, 6.6, 1H), 5.05 – 4.90 (m, 2H), 2.53 (t, J = 7.6, 2H), 2.37 (s, 3H), 2.19 – 2.06 (m, 2H), 1.78 (s, 3H).

^{13}C-NMR (75 MHz, $CDCl_3$, 299 K): δ (ppm) = 201.0, 156.3, 138.4, 137.3, 137.0, 129.3, 128.5, 127.7, 115.6, 39.9, 31.8, 30.4, 21.4.

HRMS (ESI) *m/z*: $[M+Na]^+$ calculated for $C_{15}H_{18}ONa^+$: 237.1250 found: 237.1245.

4-(*m*-Tolyl)octa-3,7-dien-2-one (**1g**)

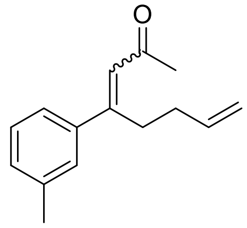

(E)-4-(*m*-Tolyl)octa-3,7-dien-2-one and *(Z)*-4-(*m*-tolyl)octa-3,7-dien-2-one (**1g**) were prepared according to GP3 using ethyl 3-(*m*-tolyl)hepta-2,6-dienoate (**4g**) (1.22 g, 5.00 mmol, 1.00 eq.), N,O-dimethylhydroxylamine hydrochloride (975 mg, 10.0 mmol, 2.00 eq.), isopropylmagnesium chloride (2.0 M in diethyl ether, 10.8 mL, 21.5 mmol, 4.30 eq.), methylmagnesium bromide (3.0 M in diethyl ether, 2.5 mL, 7.5 mmol, 1.3 eq.) and tetrahydrofuran (32 mL). Both isomers were separated *via* flash column chromatography (pentane : diethyl ether = 95:5). *(E)*-4-(*m*-tolyl)octa-3,7-dien-2-one (268 mg, 1.25 mmol, 25%) and *(Z)*-4-(*m*-tolyl)octa-3,7-dien-2-one (292 mg, 1.36 mmol, 27%) (**1g**) were each isolated as a yellow liquid.

(E)-4-(*m*-tolyl)octa-3,7-dien-2-one (268 mg, 1.25 mmol, 25%):

^{1}H-NMR (300 MHz, $CDCl_3$, 299 K): δ (ppm) = 7.31 – 7.15 (m, 4H), 6.43 (s, 1H), 5.85 (ddt, J = 16.9, 10.2, 6.7 Hz, 1H), 5.07 – 4.92 (m, 2H), 3.19 – 3.11 (m, 2H), 2.40 (s, 3H), 2.30 (s, 3H), 2.18 (dtt, J = 9.3, 6.5, 1.4 Hz, 2H).

^{13}C-NMR (75 MHz, $CDCl_3$, 299 K): δ (ppm) = 198.6, 158.5, 141.6, 138.3, 138.0, 129.9, 128.6, 127.6, 125.1, 124.1, 114.9, 33.1, 32.4, 30.6, 21.6.

HRMS (ESI) *m/z*: $[M+Na]^+$ calculated for $C_{15}H_{18}ONa^+$: 237.1250, found:237.1248

(Z)-4-(*m*-tolyl)octa-3,7-dien-2-one (292 mg, 1.36 mmol, 27%):

^{1}H-NMR (300 MHz, $CDCl_3$, 299 K): δ (ppm) = 7.33 – 7.11 (m, 2H), 7.01 – 6.88 (m, 2H), 6.07 (s, 1H), 5.77 (ddt, J = 16.9, 10.3, 6.5 Hz, 1H), 5.08 – 4.93 (m, 2H), 2.57 – 2.49 (m, 2H), 2.36 (s, 3H), 2.20 – 2.07 (m, 2H), 1.77 (s, 3H).

^{13}C-NMR (75 MHz, $CDCl_3$, 299 K): δ (ppm) = 200.9, 156.4, 140.0, 138.2, 137.2, 129.1, 128.5, 128.4, 128.2, 124.8, 115.6, 39.9, 31.7, 30.3, 21.5.

HRMS (ESI) *m/z*: $[M+Na]^+$ calculated for $C_{15}H_{18}ONa^+$: 237.1250, found:237.1248.

4-(Naphthalen-2-yl)octa-3,7-dien-2-one (**1h**)

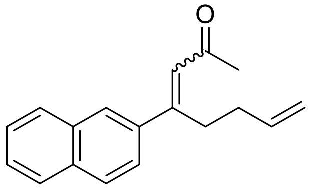

(*E*)-4-(Naphthalen-2-yl)octa-3,7-dien-2-one and (*Z*)-4-(naphthalen-2-yl)octa-3,7-dien-2-one (**1h**) were prepared according to GP3 using ethyl 3-(naphthalen-2-yl)hepta-2,6-dienoate (**4h**) (1.79 g, 6.06 mmol, 1.00 eq.), N,O-dimethylhydroxylamine hydrochloride (1.18 g, 12.13 mmol, 2.00 eq.), isopropylmagnesium chloride (2.0 M in diethyl ether, 12.4 mL, 24.9 mmol, 4.10 eq.), methylmagnesium bromide (3.0 M in diethyl ether, 2.0 mL, 9.1 mmol, 1.5 eq.) and tetrahydrofuran (40 mL). The isomers were separated by flash column chromatography (pentane : diethyl ether = 90:10). (*E*)-4-(naphthalen-2-yl)octa-3,7-dien-2-one (465 mg, 1.86 mmol, 31%) and (*Z*)-4-(naphthalen-2-yl)octa-3,7-dien-2-one (**1h**) (414 mg, 1.65 mmol, 27%) were each isolated as a yellow liquid.

(*E*)-4-(naphthalen-2-yl)octa-3,7-dien-2-one (465 mg, 1.86 mmol, 31%):

^{1}H-NMR (300 MHz, $CDCl_3$, 299 K): δ (ppm) = 7.96 – 7.77 (m, 4H), 7.60 – 7.46 (m, 3H), 6.56 (s, 1H), 5.86 (ddt, J = 16.8, 10.1, 6.6, 1H), 5.06 – 4.89 (m, 2H), 3.32 – 3.20 (m, 2H), 2.32 (s, 3H), 2.22 (tdt, J = 9.3, 6.6, 1.4 Hz, 2H).

^{13}C-NMR (75 MHz, $CDCl_3$, 299 K): δ (ppm) = 198.5, 158.1, 138.8, 138.0, 133.7, 133.3, 128.6, 128.4, 127.8, 126.9, 126.7, 126.5, 125.6, 124.6, 115.0, 33.3, 32.5, 30.5.

HRMS (ESI) *m/z*: $[M+Na]^+$ calculated for $C_{18}H_{18}ONa^+$: 273.1250; found: 273.1246.

(*Z*)-4-(naphthalen-2-yl)octa-3,7-dien-2-one (414 mg, 1.65 mmol, 27%):

^{1}H-NMR (300 MHz, $CDCl_3$, 299 K): δ (ppm) = 7.90 – 7.80 (m, 3H), 7.65 – 7.62 (m, 1H), 7.55 – 7.48 (m, 2H), 7.30 (dd, J = 8.4, 1.8, 1H), 6.19 (s, 1H), 5.79 (ddt, J = 16.1, 10.9, 6.6, 1H), 5.05 – 4.95 (m, 2H), 2.64 (td, J = 7.5, 1.1, 2H), 2.17 (tdt, J = 7.9, 6.6, 1.5 Hz, 2H), 1.78 (s, 3H).

^{13}C-NMR (75 MHz, $CDCl_3$, 299 K): δ (ppm) = 200.7, 156.0, 137.5, 137.2, 133.2, 133.1, 128.9, 128.3, 128.2, 127.9, 126.9, 126.7, 126.6, 125.7, 115.7, 39.9, 31.8, 30.5.

HRMS (ESI) *m/z*: $[M+Na]^+$ calculated for $C_{18}H_{18}ONa^+$: 273.1250; found: 273.1245.

1-Cyclohexyl-3-phenylhepta-2,6-dien-1-one (**1i**)

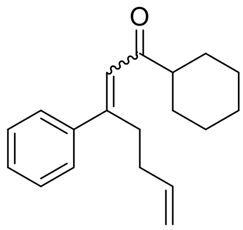

1-Cyclohexyl-3-phenylhepta-2,6-dien-1-one (**1j**) was prepared according to GP3 using ethyl-3-phenylhepta-2,6-dienoate (**4a**) (691 mg, 3.00 mmol, 1.00 eq.), N,O-dimethylhydroxylamine hydrochloride (585 mg, 6.00 mmol, 2.00 eq.), isopropylmagnesium chloride (2.0 M in diethyl ether, 6.50 mL, 13.0 mmol, 4.30 eq.), cyclohexylmagnesium bromide (1.3 M in diethyl ether, 3.0 mL, 3.9 mmol, 1.3 eq.) and tetrahydrofuran (20 mL). The product (164 mg, 0.61 mmol, 20%) was isolated as a yellow liquid by flash column chromatography (pentane : diethyl ether = 95:5).

^{1}H-NMR (300 MHz, $CDCl_3$, 299 K): δ (ppm) = 7.40 – 7.28 (m, 3H), 7.17 – 7.10 (m, 2H), 6.14 (d, J = 1.2 Hz, 1H), 5.78 (ddt, J = 17.0, 10.4, 6.6 Hz, 1H), 5.07 – 4.95 (m, 2H), 2.54 (td, J = 7.5, 1.2 Hz, 2H), 2.24 – 2.03 (m, 3H), 1.76 – 1.52 (m, 5H), 1.31 – 0.95 (m, 5H).

^{13}C-NMR (75 MHz, $CDCl_3$, 299 K): δ (ppm) = 205.3, 155.1, 140.3, 137.3, 128.3, 128.0, 127.5, 126.0, 115.6, 50.5, 39.7, 31.8, 28.8, 26.0, 25.9.

HRMS (ESI) *m/z*: $[M+Na]^+$ calculated for $C_{19}H_{24}ONa^+$: 291.1719; found: 291.1718.

1,3-Diphenylhepta-2,6-dien-1-one (**1j**)

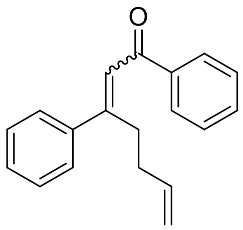

1,3-Diphenylhepta-2,6-dien-1-one (**1i**) was prepared according to GP3 using ethyl-3-phenylhepta-2,6-dienoate (**4a**) (691 mg, 3.00 mmol, 1.00 eq.), N,O-dimethylhydroxylamine hydrochloride (585 mg, 6.00 mmol, 2.00 eq.), isopropylmagnesium chloride (2.0 M in diethyl ether, 6.50 mL, 13.0 mmol, 4.30 eq.), phenylmagnesium bromide (3.0 M in diethylether, 1.3 mL, 3.9 mmol, 1.3 eq.) and tetrahydrofuran (16 mL). The product (464 mg, 1.77 mmol, 59%) was isolated as a yellow oil after flash column chromatography (pentane : diethyl ether = 90:10).

^{1}H-NMR (300 MHz, $CDCl_3$, 299 K): δ (ppm) = 7.87 – 7.79 (m, 2H), 7.45 (ddt, J = 8.3, 6.5, 1.4 Hz, 1H), 7.39 – 7.31 (m, 2H), 7.25 – 7.11 (m, 5H), 6.67 (d, J = 1.2 Hz, 1H), 5.86 (ddt, J = 16.2, 10.9, 6.6 Hz, 1H), 5.11 – 4.98 (m, 2H), 2.69 (ddd, J = 7.5, 6.8, 1.2 Hz, 2H), 2.24 (tdt, J = 7.9, 6.6, 1.4 Hz, 2H).

^{13}C-NMR (75 MHz, $CDCl_3$, 299 K): δ (ppm) = 193.4, 155.8, 139.9, 138.2, 137.4, 132.7, 128.9, 128.4, 128.2, 128.0, 127.8, 124.3, 115.7, 39.2, 32.0.

HRMS (ESI) *m/z*: $[M+Na]^+$ calculated for $C_{19}H_{18}ONa^+$: 285.1250; found: 285.1249.

(E)-4-(2-oxoocta-3,7-dien-4-yl)benzonitrile (**1k**)

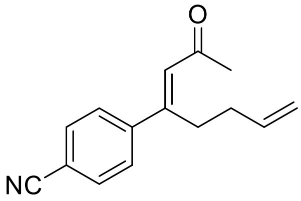

(E)-4-(2-oxoocta-3,7-dien-4-yl)benzonitrile (1k) was prepared according to GP3 using ethyl 3-(4-cyanophenyl)hepta-2,6-dienoate (**4i**) (510 mg, 2.00 mmol, 1.00 eq.), N,O-dimethylhydroxylamine hydrochloride (390 mg, 4.00 mmol, 2.00 eq.), isopropylmagnesium chloride (2.0 M in diethyl ether, 4.30 mL, 8.60 mmol, 4.30 eq.), methylmagnesium bromide (3.0 M in diethyl ether, 0.9 mL, 2.6 mmol, 1.5 eq) and tetrahydrofuran (12 mL). The product (74.4 mg, 0.33 mmol, 17%) was isolated as a pale yellow oil by flash column chromatography (pentane : diethyl ether = 75:25).

^{1}H-NMR (300 MHz, $CDCl_3$, 299 K): δ (ppm) = 7.70 – 7.63 (m, 1H), 7.55 – 7.47 (m, 1H), 6.40 (s, 0H), 5.85 – 5.63 (m, 1H), 5.00 – 4.85 (m, 1H), 3.16 – 3.05 (m, 1H), 2.29 (s, 1H), 2.19 – 2.05 (m, 1H).

^{13}C-NMR (75 MHz, $CDCl_3$, 299 K): δ (ppm) = 198.2, 155.6, 146.2, 137.1, 132.4, 127.6, 127.0, 118.5, 115.4, 112.5, 32.8, 32.4, 30.4.

HRMS (ESI) *m/z*: $[M+Na]^+$ calculated for $C_{15}H_{15}NONa^+$: 248.1046; found: 248.1046.

(Z)-4-(2-oxoocta-3,7-dien-4-yl)benzonitrile (**1k**)

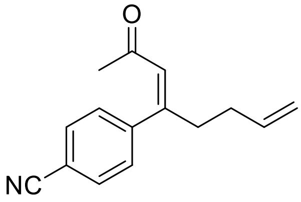

(Z)-4-(2-oxoocta-3,7-dien-4-yl)benzonitrile (1k) was prepared according to GP3 using the major diasteromer of ethyl 3-(4-cyanophenyl)hepta-2,6-dienoate (**4i**) (730 mg, 3.00 mmol, 1.00 eq.), N,O-dimethylhydroxylamine hydrochloride (585 mg, 6.00 mmol, 2.00 eq.), isopropylmagnesium chloride (2.0 M in diethyl ether, 6.50 mL, 13.0 mmol, 4.30 eq.), methylmagnesium bromide (3.0 M in diethyl ether, 1.3 mL, 2.6 mmol, 1.5 eq) and tetrahydrofuran (24 mL). The product (134 mg, 0.60 mmol, 20%) was isolated as a pale yellow oil by flash column chromatography (pentane : diethyl ether = 65:35).

^{1}H-NMR (300 MHz, $CDCl_3$, 299 K): δ (ppm) = 7.71 – 7.63 (m, 2H), 7.28 – 7.21 (m, 2H), 6.22 (s, 1H), 5.84 – 5.65 (m, 1H), 5.10 – 4.92 (m, 2H), 2.52 (td, J = 7.5, 1.3 Hz, 2H), 2.21 – 2.07 (m, 3H), 1.98 (s, 3H).

^{13}C-NMR (75 MHz, $CDCl_3$, 299 K): δ (ppm) = 198.1, 153.9, 145.2, 136.5, 132.1, 128.2, 127.5, 116.1, 111.7, 39.3, 31.3, 31.1.

HRMS (ESI) *m/z*: $[M+Na]^+$ calculated for $C_{15}H_{15}NONa^+$: 248.1046; found: 248.1047.

3. Optimization of the intramolecular [2+2] cycloaddition

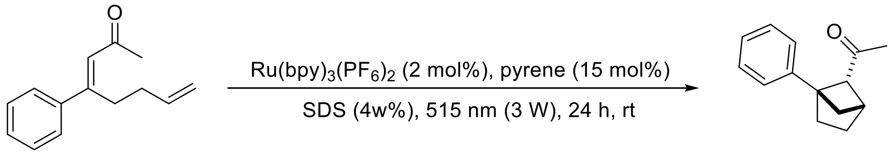

Table 1: Optimization of the reaction.

Entry	Sensitizer	Annihilator	Tenside	Time	NMR yield(isolated yield)
1[a,b]	$Ru(bpy)_3(PF_6)_2$ (2 mol%)	Pyrene (15 mol%)	SDS	24h	19%
2[b]	$Ru(bpy)_3(PF_6)_2$ (2 mol%)	Pyrene (15 mol%)	Triton-X-100	24 h	48%
3[b]	$Ru(bpy)_3(PF_6)_2$ (2 mol%)	Pyrene (15 mol%)	SBDS	24 h	34%
4[b]	$Ru(bpy)_3(PF_6)_2$ (2 mol%)	Pyrene (15 mol%)	CTAC	24 h	12%
5[b,c]	$Ru(bpy)_3(PF_6)_2$ (2 mol%)	Pyrene (15 mol%)	TPGS-750-M	24 h	28%
6[b]	$Ru(bpy)_3(PF_6)_2$ (2 mol%)	Pyrene (15 mol%)	SDS	24 h	62%
7[b]	$Ru(bpy)_3(PF_6)_2$ (2 mol%)	Pyrene (15 mol%)	SDS	30 h	71%
8[b]	$Ru(bpy)_3(PF_6)_2$ (2 mol%)	Pyrene (15 mol%)	SDS	48 h	82%
9[b]	$Ru(bpy)_3(PF_6)_2$ (2 mol%)	Pyrene (15 mol%)	SDS	48 h	69%
10[b]	$Ru(bpy)_3(PF_6)_2$ (2 mol%)	Pyrene (30 mol%)	SDS	24 h	63%
11[b]	$Ru(bpy)_3(PF_6)_2$ (1 mol%)	Pyrene (10 mol%)	SDS	48 h	70%
12[d]	$Ru(bpy)_3(PF_6)_2$ (2 mol%)	Pyrene (15 mol%)	SDS	24 h	76%(78%)
13[d]	$Ru(bpy)_3(PF_6)_2$ (2 mol%)	Pyrene (15 mol%)	SDS	48 h	80%
14[d]	-	-	SDS	24 h	0%
15[d]	$Ru(bpy)_3(PF_6)_2$ (2 mol%)	-	SDS	24 h	28%
16[d]	-	Pyrene (15 mol%)	SDS	24 h	0%
17[e]	$Ru(bpy)_3(PF_6)_2$ (2 mol%)	Pyrene (15 mol%)	SDS	48 h	0%
18[d,f]	$Ru(bpy)_3(PF_6)_2$ (2 mol%)	Pyrene (15 mol%)	ACN	24 h	52%
19[d,f]	$Ru(bpy)_3(PF_6)_2$ (2 mol%)	Pyrene (15 mol%)	CH_2Cl_2	24 h	45%
20[d,f]	$Ru(bpy)_3(PF_6)_2$ (2 mol%)	Pyrene (15 mol%)	MeOH	24h	84% (79%)
21[d,f]	$Ru(bpy)_3(PF_6)_2$ (2 mol%)	Pyrene (15 mol%)	SDS	24 h	79%
22[d,f]	$Ru(bpy)_3(PF_6)_2$ (2 mol%)	Pyrene (15 mol%)	water	24 h	22%
23[d,g]	$Ru(bpy)_3(PF_6)_2$ (2 mol%)	Pyrene (15 mol%)	SDS	24 h	60%
24[d,h]	$Ru(bpy)_3(PF_6)_2$ (2 mol%)	Pyrene (15 mol%)	SDS	24 h	25%
25[d,i]	$Ru(bpy)_3(PF_6)_2$ (2 mol%)	Pyrene (15 mol%)	SDS	24 h	0%

Reactions were performed with (*E*)-4-Phenylocta-3,7-dien-2-one (0.2 mmol), $Ru(bpy)_3(PF_6)_2$ (2 mol%), pyrene (15 mol%) in solvent (6 mL) under irradiation with a 520 nm LED light (10 W) for 24 h at rt. For the determination of NMR yield, toluene (5.3 µl, 0.05 mmol) was used as internal standard. [a] addition of 20 mol% benzophenone [b] 4 w% of micellar solution, [c] 2 w% of micellar solution, [d] 10 W LED lamp, [e] dark reaction, [f] degassed solvent, [g] 2 equiv. of DIPEA, [h] 5 equiv. of DIPEA, [i] 10 equiv. of DIPEA.

4. Synthesis and characterization data of bicyclo[2.1.1]hexanes and derivatives

General procedure for the intramolecular [2+2] cycloaddition (GP4)

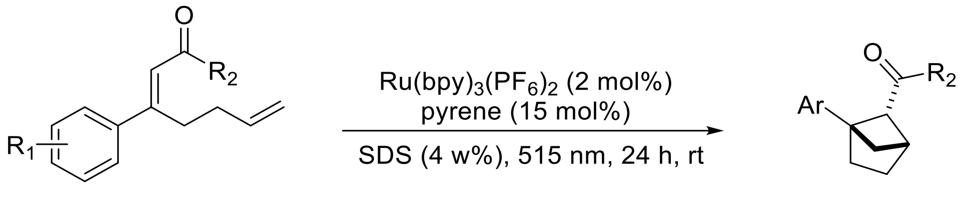

0.2 mmol

In a headspace vial equipped with a magnetic stir bar, the α,β-unsaturated ketone (0.20 mmol, 1.0 eq.), $Ru(bpy)_3(PF_6)_2$ (3.4 mg, 4.0 µmol, 2.0 mol%), pyrene (6.1 mg, 0.03 mmol, 15 mol%) and SDS solution (240 mg in 5.76 mL H_2O) were added and the reaction stirred until a homogeneous solution was observed. Afterwards the solution was irradiated with a 520 nm LED lamp (10 W) for 24 h at 20 °C. The reaction mixture was transferred to a separatory funnel diluted with dichloromethane and saturated, aqueous NaCl solution. The phases were separated and the aqueous phase was extracted six times with dichloromethane. The combined organic phases were dried over $MgSO_4$ and the solvent removed under reduced pressure. The product was isolated *via* flash column chromatography.

Table 2: Yields of the employed substrates.

Product	Substrate	Yield
**2a**	(*Z*)-**1a** (40.4 mg, 0.20 mmol, 1.00 eq)	25.0 mg, 0.17 mmol, 87%
	(*E*)-**1a** (40.4 mg, 0.20 mmol, 1.00 eq)	31.3 mg, 0.16 mmol, 80%
**2b**	*(Z)*-**1b** (43.7 mg; 0.20 mmol, 1.00 eq)	28.0 mg, 0.13 mmol, 64%
	(E)-**1b** (43.6 mg; 0.20 mmol, 1.00 eq)	30.0 mg, 0.14 mmol, 69%
**2c**	(*Z*)-**1c** (46.9 mg, 0.20 mmol, 1.00 eq.)	37. 0mg, 0.16 mmol, 79%
	(*E*)-**1c** (47.0 mg, 0.20 mmol, 1.00 eq.)	34.0 mg, 0.14 mmol, 72%
**2d**	*(Z)*-**1d** (55.8 mg, 0.20 mmol, 1.00 eq.)	47.9 mg, 0.17 mmol, 86%
	(E)-**1d** (55.7 mg, 0.20 mmol, 1.00 eq.)	49.5 mg, 0.18 mmol, 89%
**2e**	*(Z)*-**1e** (53.7 mg; 0.20 mmol, 1.00 eq)	39.5 mg, 0.15 mmol, 74%
	(E)-**1e** (53.6 mg; 0.20 mmol, 1.00 eq)	45.0 mg, 0.17 mmol, 82%
**2f**	*(Z)*-**1f** (42.8 mg, 0.20 mmol, 1.00 eq.)	28.0 mg, 0.13 mmol, 65%

	(E)-**1f** (42.8 mg, 0.20 mmol, 1.00 eq.)	28.5 mg, 0.13 mmol, 67%
**2g**	*(Z)*-**1g** (42.8 mg; 0.20 mmol, 1.00 eq.)	28.5 mg, 0.13 mmol, 67%
	(E)-**1g** (42.9 mg; 0.20 mmol, 1.00 eq.)	33.0 mg, 0.15 mmol, 74%
**2h**	*(Z)*-**1h** (50.1 mg, 0.20 mmol, 1.00 eq.)	47.0 mg, 0.19 mmol, 94%
	(E)-**1h** (50.0 mg, 0.20 mmol, 1.00 eq.)	46.0 mg, 0.18 mmol, 92%
**2i**	**1i** (53.9 mg, 0.20 mmol, 1.00 eq.)	46.7 mg, 0.17 mmol, 87%
**2j**	**1j** (53.6 mg, 0.20 mmol, 1.00 eq.)	45.0 mg, 0.17 mmol, 86%
**2k**	(*Z*)-**1k**	
	(*E*)-**1k** (45.1 mg, 0.20 mmol, 1.00 eq.)	39.6 mg; 0.16 mmol, 78%

1-(1-Phenylbicyclo[2.1.1]hexan-5-yl)ethan-1-one (**2a**)

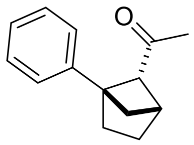

The product **2a** was synthesized according to the general procedure **GP4** and purified as a yellow liquid after flash column chromatography in a diastereomeric ratio of 20:1 (pentane : diethyl ether = 95:5).

^{1}H-NMR (600 MHz, $CDCl_3$, 299 K): δ (ppm) = 7.45 – 7.14 (m, 5H), 2.83 (tt, J = 2.9, 1.4 Hz, 1H), 2.64 (d, J = 2.9 Hz, 1H), 2.09 (ddt, J = 10.3, 7.4, 4.7 Hz, 1H), 1.89 (s, 3H), 1.84 – 1.62 (m, 4H), 1.30 (dt, J = 6.4, 1.1 Hz, 1H).

^{13}C-NMR (151 MHz, $CDCl_3$, 299 K): δ (ppm) = 207.9, 141.9, 128.3, 127.0, 126.5, 61.1, 57.2, 41.7, 39.8, 28.8, 28.1, 26.2.

HRMS (ESI) *m/z*: $[M+Na]^+$ calculated for $C_{14}H_{16}ONa^+$ 223.1093, found 223.1092.

1-(4-Fluorophenyl)bicyclo[2.1.1]hexan-5-yl)ethan-1-one (**2b**)

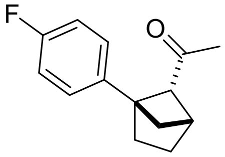

The product **2b** was synthesized according to the general procedure **GP4** and purified as a colorless liquid after flash column chromatography (pentane : diethyl ether = 95:5).

^{1}H-NMR (300 MHz, $CDCl_3$, 299 K): δ (ppm) = 7.39 – 7.30 (m, 2H), 7.05 – 6.94 (m, 2H), 2.84 (tt, J=2.9, 1.3, 1H), 2.59 (d, J=2.8, 1H), 2.16 – 2.01 (m, 1H), 1.92 (s, 3H), 1.84 – 1.63 (m, 4H), 1.30 (dd, J=6.4, 0.8, 1H).

^{13}C-NMR (75 MHz, $CDCl_3$, 299 K): δ (ppm) = 208.0, 161.7 (d, J=244.6), 137.9 (d, J=3.2), 128.7 (d, J=7.7), 115.1 (d, J=21.2), 61.4, 56.5, 41.7, 40.1, 29.2, 28.1, 26.4.

^{19}F-NMR (282 MHz, $CDCl_3$, 299 K) δ (ppm) = -116.5.

HRMS (ESI) *m/z*: $[M+Na]^+$ calculated for $C_{14}H_{15}FONa^+$:241.1000, found: 241.0999.

1-(1-(4-chlorophenyl)bicyclo[2.1.1]hexan-5-yl)ethan-1-one (**2c**)

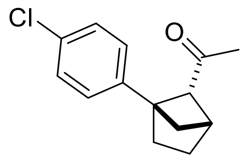

The product **2c** was synthesized according to the general procedure **GP 4** and purified as a colorless liquid after flash column chromatography (pentane : diethyl ether = 95:5).

^{1}H-NMR (300 MHz, $CDCl_3$, 299 K): δ (ppm) = 7.34 – 7.20 (m, 4H), 2.83 (tt, J = 2.8, 1.3, 1H), 2.57 (d, J=2.8, 1H), 2.15 – 2.02 (m, 1H), 1.90 (s, 3H), 1.83 – 1.58 (m, 4H), 1.32 – 1.25 (m, 1H).

^{13}C-NMR (75 MHz, $CDCl_3$, 299 K): δ (ppm) = 207.9, 140.7, 132.3, 128.6, 128.4, 61.2, 56.5, 41.6, 40.2, 29.2, 28.1, 26.4.

HRMS (ESI) *m/z*: $[M+Na]^+$ calculated for $C_{14}H_{15}OClNa^+$: 257.0704, found:257.0702.

1-(1-(4-bromophenyl)bicyclo[2.1.1]hexan-5-yl)ethan-1-one (**2d**)

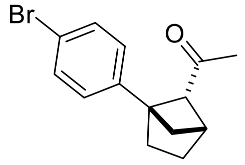

The product **2d** was synthesized according to the general procedure **GP4** and purified as a yellow liquid in a 20:1 diastereomeric ratio after flash column chromatography (pentane : diethyl ether = 93:7).

^{1}H-NMR (600 MHz, $CDCl_3$, 299 K): δ (ppm) = 7.45 – 7.37 (m, 2H), 7.26 (dt, J = 6.4, 2.2 Hz, 2H), 2.84 (tt, J = 2.8, 1.4 Hz, 1H), 2.59 (d, J = 2.8 Hz, 1H), 2.18 – 2.04 (m, 1H), 1.92 (s, 3H), 1.96 – 1.57 (m, 4H), 1.30 (dd, J = 6.4, 0.8 Hz, 1H).

^{13}C-NMR (151 MHz, $CDCl_3$, 299 K): δ (ppm) = 207.8, 141.2, 131.4, 128.9, 120.4, 61.2, 56.3, 41.5, 40.2, 29.1, 28.1 26.4.

HRMS (ESI) *m/z*: $[M+Na]^+$ calculated for $C_{14}H_{15}BrONa^+$ 301.0199, found 301.0198.

1-(1-(4-(Trifluoromethyl)phenyl)bicyclo[2.1.1]hexan-5-yl)ethan-1-one (**2e**)

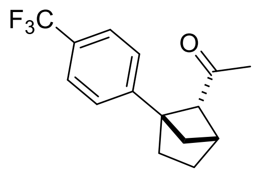

The product **2e** was synthesized according to the general procedure **GP4** and purified as a colorless liquid, with a 3% starting material impurity which has been subtracted from the reported yield, after flash column chromatography (pentane : diethyl ether = 93:7).

^{1}H-NMR (300 MHz, $CDCl_3$, 299 K): δ (ppm) = 7.60 – 7.46 (m, 4H), 2.90 (s, 1H), 2.65 (s, 1H), 2.24 – 2.12 (m, 1H), 1.97 (s, 3H), 1.88 – 1.63 (m, 4H), 1.37 (d, J = 6.4 Hz, 1H).

^{13}C-NMR (75 MHz, $CDCl_3$, 299 K): δ (ppm) = 207.7, 146.3, 128.64 (d, J=32.4 Hz) 127.4, 125.1 (q, J = 3.8 Hz), 124.59 (d, J=271.9 Hz), 61.0, 56.5, 41.4, 40.5, 29.3, 27.9, 26.3.

^{19}F-NMR (282 MHz, $CDCl_3$, 299 K) δ (ppm) = -62.4.

HRMS (ESI) *m/z*: $[M+Na]^+$ calculated for $C_{15}H_{15}F_3ONa^+$: 291.0968, found: 291.0966.

1-(*p*-Tolyl)bicyclo[2.1.1]hexan-5-yl)ethan-1-one (2f)

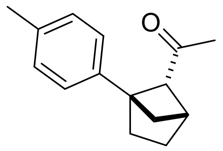

The product **2f** was synthesized according to the general procedure **GP4** and purified as a colorless liquid by flash column chromatography (pentane : diethyl ether = 90:10).

^{1}H-NMR (500 MHz, $CDCl_3$, 299 K): δ (ppm) =7.32 – 7.27 (m, 2H), 7.16 – 7.12 (m, 2H), 2.82 (tt, *J*=2.8, 1.3, 1H), 2.62 (d, *J*=2.7, 1H), 2.34 (s, 3H), 2.11 – 2.03 (m, 1H), 1.89 (s, 3H), 1.85 – 1.75 (m, 3H), 1.71 (dt, *J*=6.4, 2.7, 1H), 1.29 (dd, *J*=6.4, 0.8, 1H).

^{13}C-NMR (125 MHz, CDCl3, 299 K): δ (ppm) = 208.1, 139.0, 136.2, 129.1, 127.1, 61.4, 57.2, 41.9, 39.8, 28.8, 28.3, 26.3, 21.2.

HRMS (ESI) *m/z*: $[M+Na]^+$ calculated for $C_{15}H_{18}ONa^+$:237.1250, found: 237.1248.

1-(1-(*m*-Tolyl)bicyclo[2.1.1]hexan-5-yl)ethan-1-one (2g)

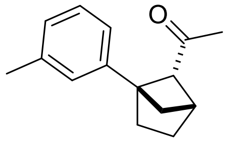

The product **2g** was synthesized according to the general procedure **GP4** and purified as a colorless liquid after flash column chromatography (pentane : diethyl ether = 95:5). A 5% impurity deriving from the starting material (double bond is still intact) was identified and has been subtracted from the yield based on the molecular weight of the starting material.

^{1}H-NMR (600 MHz, $CDCl_3$, 299 K): δ (ppm) = 7.25 – 7.17 (m, 3H), 7.05 (dtd, J = 7.2, 1.6, 0.8 Hz, 1H), 2.83 (tt, J = 2.8, 1.3 Hz, 1H), 2.65 (p, J = 1.4 Hz, 1H), 2.36 (s, 3H), 2.16 – 2.05 (m, 1H), 1.91 (s, 3H), 1.86 – 1.74 (m, 3H), 1.71 (dq, J = 6.2, 2.6 Hz, 1H), 1.30 (dd, J = 6.4, 0.8 Hz, 1H).

^{13}C-NMR (151 MHz, $CDCl_3$, 299 K): 208.1, 142.0, 137.9, 128.3, 127.8, 127.4, 124.2, 61.2, 57.3, 41.9, 39.9, 28.9, 28.3, 26.3, 21.6.

HRMS (ESI) *m/z*: $[M+Na]^+$ calculated for $C_{15}H_{18}ONa^+$: 237.1250, found 237.1247.

1-(1-(Naphthalen-2-yl)bicyclo[2.1.1]hexan-5-yl)ethan-1-one (2h)

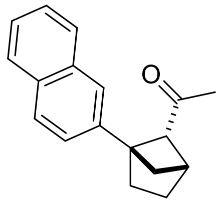

The product **2h** was synthesized according to the general procedure **GP4** and purified as a colorless liquid by flash column chromatography (pentane : diethyl ether = 96:4).

^{1}H-NMR (300 MHz, $CDCl_3$, 299 K): δ (ppm) = 7.87 – 7.75 (m, 4H), 7.60 (dd, J=8.5, 1.8, 1H), 7.46 (ddt, J = 9.5, 7.0, 3.4 Hz, 2H), 2.90 (tt, J=2.8, 1.3, 1H), 2.78 – 2.71 (m, 1H), 2.22 (m, 1H), 1.92 (s, 3H) 1.86 (ddt, J = 12.9, 4.8, 2.3 Hz, 3H), 1.40 (dd, J=6.4, 0.8, 1H), 1.28 (s, 1H).

^{13}C-NMR (75 MHz, $CDCl_3$, 299 K): δ (ppm) = 208.1, 139.6, 133.5, 132.4, 127.9, 127.9, 127.7, 126.1, 125.7, 125.6, 125.5, 61.3, 57.5, 41.9, 40.0, 28.9, 28.3, 26.3.

HRMS (ESI) *m/z*: $[M+Na]^+$ calculated for$C_{18}H_{18}ONa^+$:273.1250, found: 273. 1247

Cyclohexyl-(1-phenylbicyclo[2.1.1]hexan-5-yl)methanone (2i)

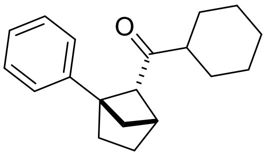

The product **2i** was synthesized according to the general procedure **GP4** and purified as a colorless liquid after flash column chromatography pentane : diethyl ether = 95:5). The product contains 2% impurity of another isomer.

^{1}H-NMR (300 MHz, $CDCl_3$, 299 K): δ (ppm) = 7.42 – 7.27 (m, 4H), 7.25 – 7.17 (m, 1H), 2.85 (tt, J = 2.9, 1.3 Hz, 1H), 2.76 (d, J = 2.8 Hz, 1H), 2.32 – 2.13 (m, 2H), 1.83 – 1.51 (m, 10H), 1.38 – 1.00 (m, 5H).

^{13}C-NMR (75 MHz, $CDCl_3$, 299 K): δ (ppm) = 213.2, 142.4, 128.3, 127.1, 126.5, 59.1, 57.4, 49.0, 41.8, 40.8, 29.3, 28.7, 28.4, 26.7, 25.9, 25.9, 25.7.

HRMS (ESI) *m/z*: $[M+Na]^+$ calculated for $C_{19}H_{24}ONa$ 291.1720, found 291.1716

Phenyl-(1-phenylbicyclo[2.1.1]hexan-5-yl)methanone (2j)

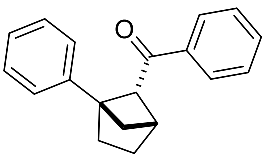

The product **2j** was synthesized according to the general procedure **GP4** and purified as a yellow liquid after flash column chromatography (pentane : diethyl ether = 97:3).

^{1}H-NMR (600 MHz, $CDCl_3$, 299 K): δ (ppm) = 7.90 (ddt, J=8.4, 1.9, 0.9, 2H), 7.56 – 7.50 (m, 1H), 7.45 – 7.39 (m, 4H), 7.34 – 7.29 (m, 2H), 7.24 – 7.19 (m, 1H), 3.31 (t, J=2.0 Hz, 1H), 3.02 (td, J=3.0, 1.4 Hz, 1H), 2.56 – 2.45 (m, 1H), 1.94 (dt, J=6.1, 2.8 Hz, 1H), 1.87 – 1.78 (m, 2H), 1.78 – 1.67 (m, 1H), 1.45 (d, J=6.3 Hz, 1H).

^{13}C-NMR (125 MHz, $CDCl_3$, 299 K): δ (ppm) = 199.0, 142.5, 136.7, 133.0, 128.5, 128.3, 128.2, 127.2, 126.5, 57.7, 57.4, 43.1, 42.0, 29.8, 26.6.

HRMS (ESI) *m/z*: $[M+Na]^+$ calculated for $C_{19}H_{18}ONa^+$: 285.1250, found: 285.1245.

4-(5-acetylbicyclo[2.1.1]hexan-1-yl)benzonitrile (2i)

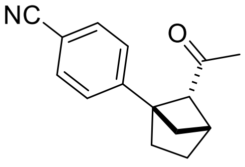

The product **2i** was synthesized according to the general procedure **GP4** and purified as a yellow liquid after flash column chromatography (pentane : diethyl ether = 75:25).

^{1}H-NMR (600 MHz, $CDCl_3$, 299 K): δ (ppm) = 7.61 – 7.54 (m, 2H), 7.52 – 7.44 (m, 2H), 2.91 (tt, J = 2.9, 1.4 Hz, 1H), 2.64 (dd, J = 3.1, 1.8 Hz, 1H), 2.18 (dddd, J = 13.6, 7.7, 3.9, 2.2 Hz, 1H), 1.98 (s, 3H), 1.89 – 1.59 (m, 4H), 1.42 – 1.30 (m, 1H).

^{13}C-NMR (125 MHz, $CDCl_3$, 299 K): δ (ppm) = 207.6, 147.9, 132.1, 127.8, 119.1, 110.1, 61.0, 56.5, 41.2, 40.7, 29.6, 27.8, 26.4.

HRMS (ESI) *m/z*: $[M+Na]^+$ calculated for 248.1046, found: 248.1045.

Unsuccesful substrates

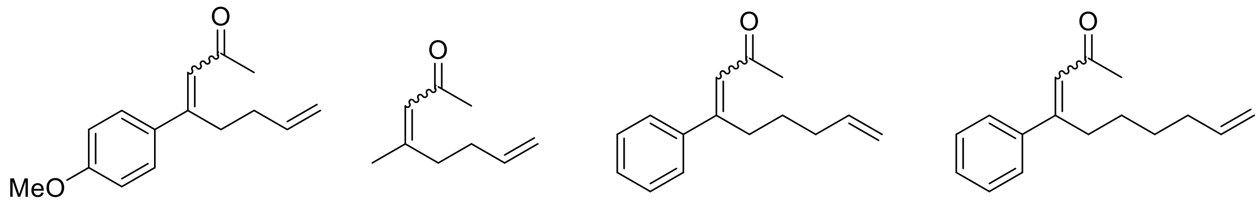

1-(1-(Naphthalen-2-yl)bicyclo[2.1.1]hexan-5-yl)ethan-1-ol (8)

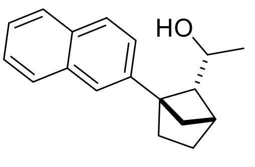

The ketone **2h** was reduced to the corresponding alcohol following a procedure by Bach.[4] The substrate (33.0 mg, 132 µmol, 1.00 eq.) was dissolved in methanol (1.4 mL) and cooled to -20 °C. Sodium borohydride (10.0 mg, 0.26 mmol, 2.00 eq.) was added portionwise and the reaction was stirred at -20 °C until completion (1.5 h, reaction control by TLC). The mixture was diluted with saturated aqueous ammonium chloride solution (20 mL), extracted with dichloromethane (3 x 20 mL), dried over magnesium sulfate and the solvent removed under reduced pressure. The product was obtained after FC (pentane : diethyl ether = 80:20) as a colorless oil (22.5 mg, 89.2 µmol, 68 %).

^{1}H-NMR (300 MHz, $CDCl_3$, 299 K): δ (ppm) = 7.87 – 7.73 (m, 4H), 7.59 (dd, J=8.5, 1.8 Hz, 1H), 7.50 – 7.38 (m, 2H), 3.78 (dq, J=9.4, 6.2 Hz, 1H), 2.47 (tt, J=2.9, 1.4 Hz, 1H), 2.15 (dddd, J=11.2, 8.7, 3.9, 2.7 Hz, 1H), 2.06 – 1.95 (m, 1H), 1.95 – 1.89 (m, 1H), 1.85 – 1.66 (m, 3H), 1.35 (d, J=6.5 Hz, 1H), 1.11 (d, J=6.2 Hz, 4H).

^{13}C-NMR (75 MHz, $CDCl_3$, 299 K): δ (ppm) = 140.4, 133.6, 132.4, 128.0, 127.9, 127.7, 126.1, 125.7, 125.6, 125.4, 65.8, 59.0, 55.5, 42.4, 38.2, 27.9, 25.6, 21.0.

HRMS (ESI) *m/z*: $[M+Na]^+$ calculated for $C_{18}H_{20}ONa^+$: 275.1406, found: 275.1404.

1-(1-(Naphthalen-2-yl)bicyclo[2.1.1]hexan-5-yl)ethyl 4-chloro-3-nitrobenzoate (6a)

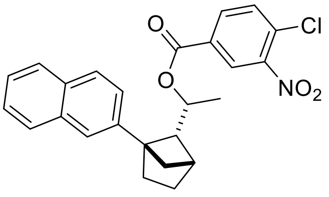

4-Chloro-3-nitrobenzoyl chloride was prepared by dissolving 4-chloro-3-nitrobenzoic acid (1.01 g, 5.00 mmol, 1.00 eq.) in dry dichloromethane (15 mL) with a catalytic amount of *N*,*N*-dimethylformamide at 0 °C, followed by addition of oxalyl chloride (952 mg, 7.50 mmol, 1.50 eq.). After stirring overnight at rt and removal of the solvent, the acid chloride was obtained as a yellow solid () and used in the next step without further purification. Alcohol **6a** (15 mg,60 µmol, 1.0 eq.) was dissolved in dry dichloromethane (0.5 mL) and cooled to 0 °C. Dry triethylamine (17 mL, 0.12 mmol, 2.0 eq.) was added and the resulting solution stirred for 15 min. 4-Chloro-3-nitrobenzoyl chloride (15 mg, 66 µmol, 1.1 eq.) was added to the solution at 0 °C and the reaction allowed to warm to rt. After stirring overnight, the mixture was extracted with ethyl acetate (2 x 20 mL), washed with 1M hydrochloric acid, water, aqueous saturated sodium chloride solution and

dried over magnesium sulfate. After removal of the solvent the product was obtained as an off-white solid (22 mg, 50 µmol, 84 %) after FC (pentane : diethyl ether = 95:5).

^{1}H-NMR (300 MHz, $CDCl_3$, 299 K): δ (ppm) = 7.75 (d, J=2.0 Hz, 1H), 7.64 – 7.54 (m, 2H), 7.48 – 7.41 (m, 3H), 7.37 – 7.27 (m, 2H), 7.12 (dd, J=8.4, 2.0 Hz, 1H), 6.78 (d, J=8.4 Hz, 1H), 5.18 (dq, J=10.3, 6.3 Hz, 1H), 2.62 (tt, J=2.8, 1.4 Hz, 1H), 2.26 (dddd, J=13.1, 10.4, 4.9, 2.7 Hz, 2H), 1.97 (dddd, J=21.7, 9.4, 4.6, 1.9 Hz, 2H), 1.89 – 1.81 (m, 2H), 1.35 (d, J=6.7 Hz, 1H), 1.27 (d, J=6.3 Hz, 3H).

^{13}C-NMR (75 MHz, $CDCl_3$, 299 K): δ (ppm) = 163.3, 146.8, 139.0, 133.1, 133.0, 131.8, 131.1, 130.8, 129.4, 127.6, 127.4, 127.4, 126.0, 125.9, 125.9, 125.7, 125.5, 70.1, 57.1, 55.3, 42.5, 38.6, 26.1, 25.2, 18.4.

HRMS (ESI) *m/z*: $[M+Na]^+$ calculated for $C_{25}H_{22}ClNO_4Na^+$: 458.1130, found: 458.1128.

5. Determination of *E/Z* configuration of the α,β-unsaturated ketones

The determination of the structure and the assignment of E/Z isomers *via* 2D-NMR analysis were performed using both diastereomers of 4-Phenylocta-3,7-dien-2-one as a model substrate. The diastereomer of 4-Phenylocta-3,7-dien-2-one with the following analytic data were analyzed using standard 2D-NMR, which revealed the relative configuration.

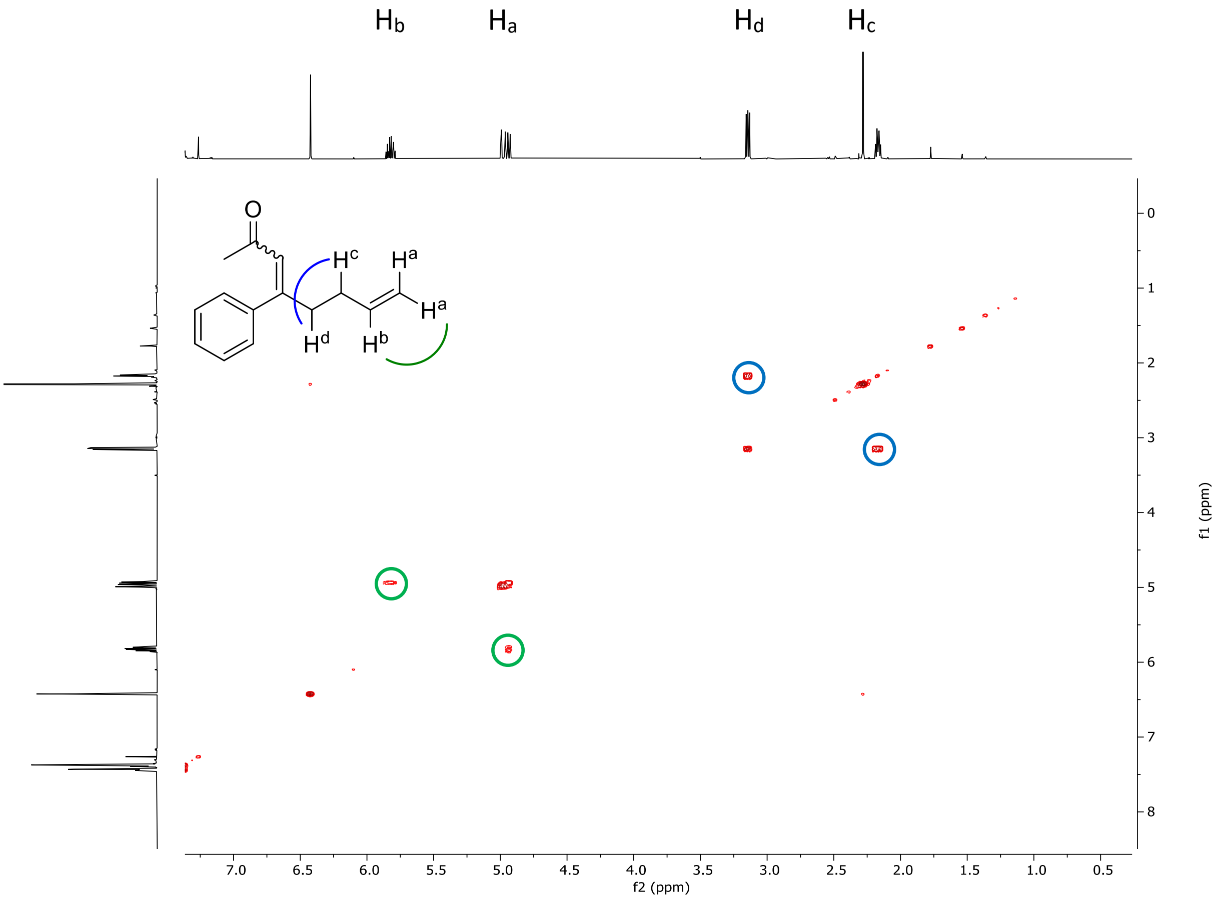

*Figure S1: COSY spectrum of (E)-4-phenylocta-3,7-dien-2-one (**1a**).*

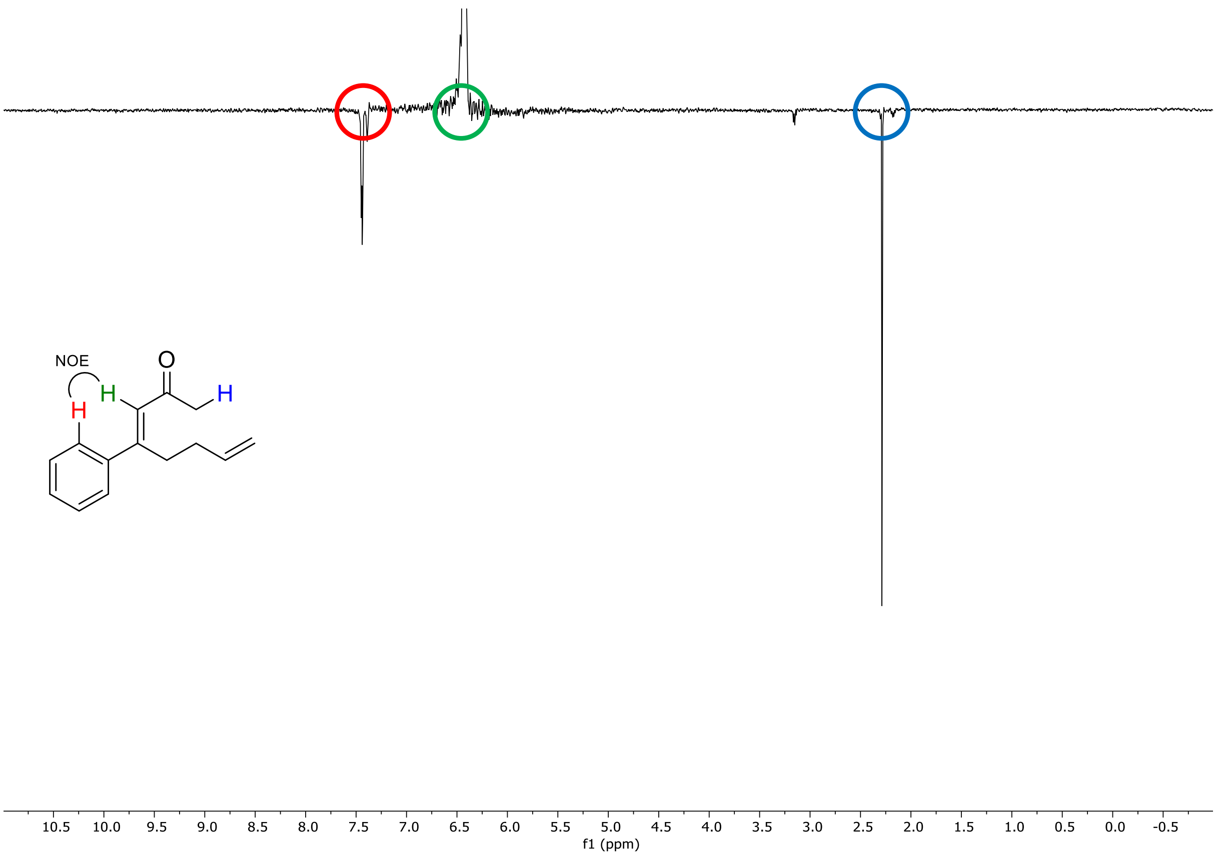

*Figure S4: Selected NOE spectrum for determination of the relative configuration of (E)-4-phenylocta-3,7-dien-2-one (**1a**).*

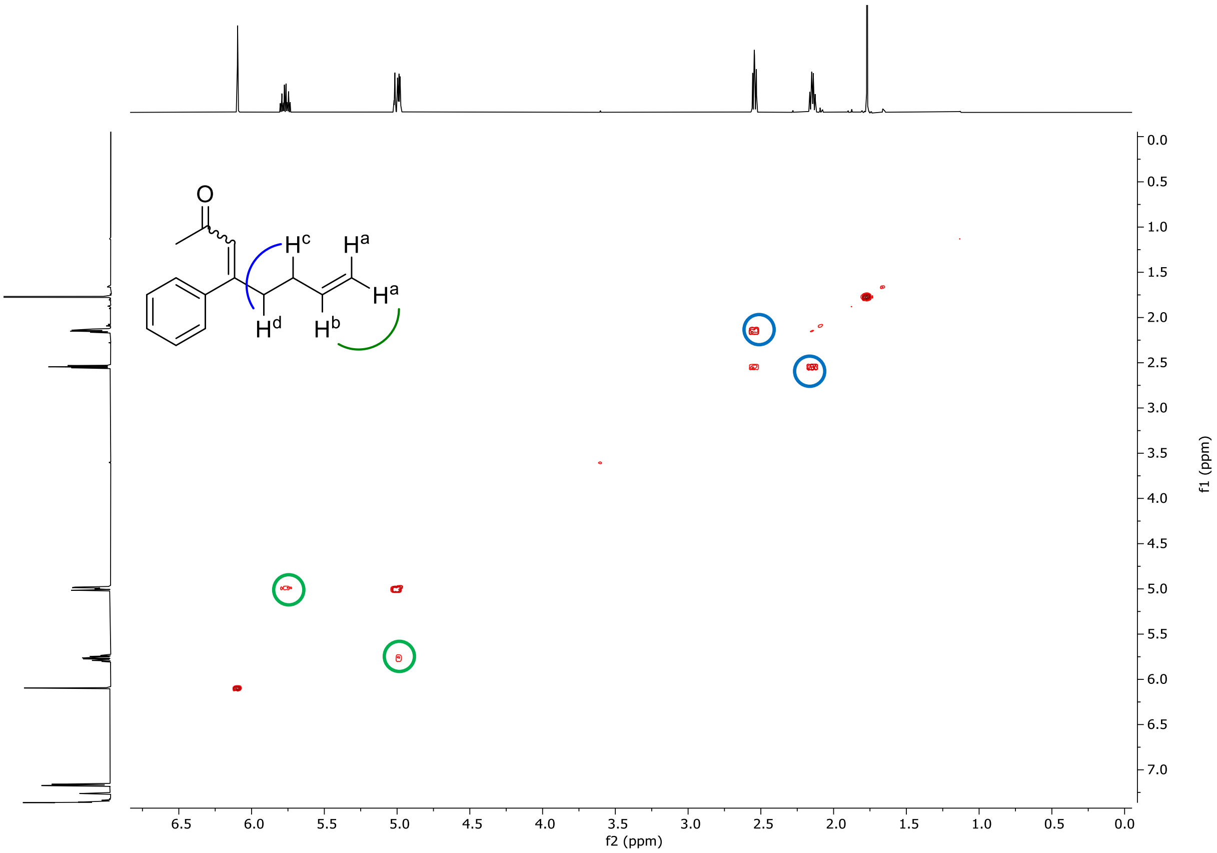

*Figure S5: COSY spectrum of (Z)-4-phenylocta-3,7-dien-2-one (**1a**).*

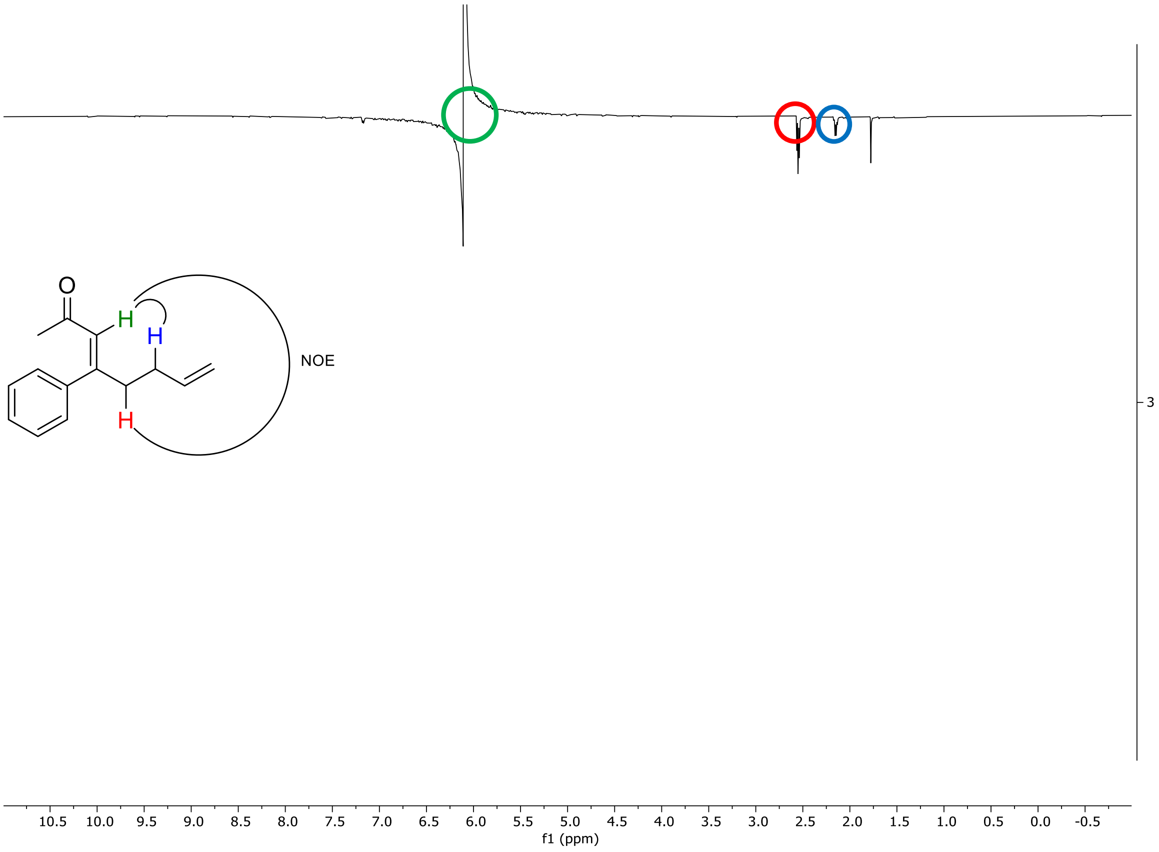

*Figure S6: Selected NOE spectrum for determination of the relative configuration of (Z)-4-phenylocta-3,7-dien-2-one (**1a**).*

6. Determination of relative configuration of bicyclo[2.1.1]hexanes

The determination of the relative configuration and constitution of the product 2a has been analyzed using 2D-NMR. The spectra have been recorded in deuterated chloroform and deuterated benzene. In benzene an improved signal separation was observed and therefore chosen for further investigation.

^{1}H-NMR (600 MHz, C_6D_6, 299 K): δ (ppm) = 7.33 – 7.28 (m, 2H), 7.19 – 7.11 (m, 2H), 7.10 – 7.01 (m, 1H), 2.38 (tt, J = 2.9, 1.4 Hz, 1H), 2.17 (dddd, J = 10.7, 8.9, 3.5, 2.6 Hz, 1H), 2.11 – 2.07 (m, 1H), 1.71 (ddddd, J = 11.4, 9.1, 3.8, 2.5, 1.4 Hz, 1H), 1.55 (tdd, J = 10.8, 3.6, 1.5 Hz, 1H), 1.46 (tdt, J = 10.7, 3.2, 1.4 Hz, 1H), 1.45 (s, 2H), 1.32 (dq, J = 6.3, 2.7 Hz, 1H), 0.95 (dd, J = 6.3, 0.8 Hz, 1H).

^{13}C NMR (151 MHz, C_6D_6, 299 K): δ (ppm) = 205.7, 142.5, 128.5, 127.4, 126.8, 61.1, 57.3, 41.6, 40.1, 29.3, 27.7, 26.5.

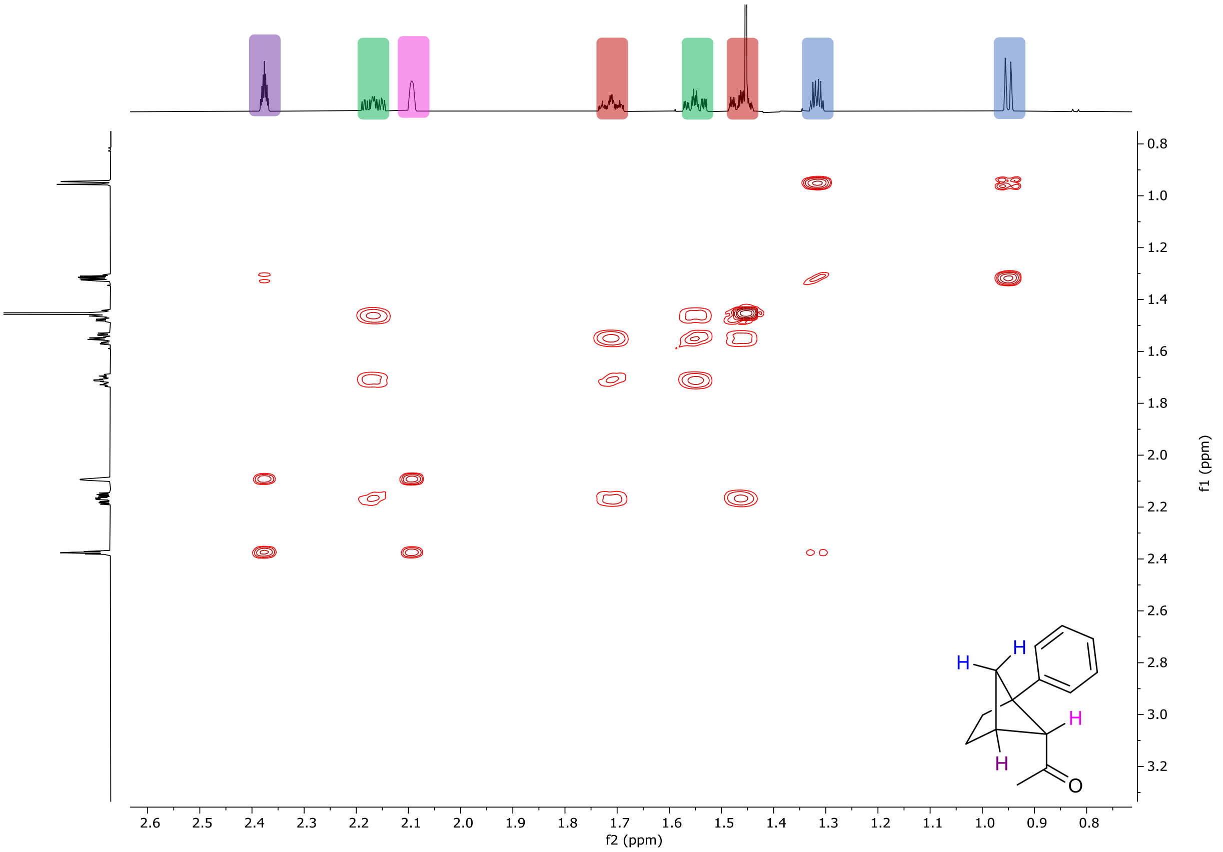

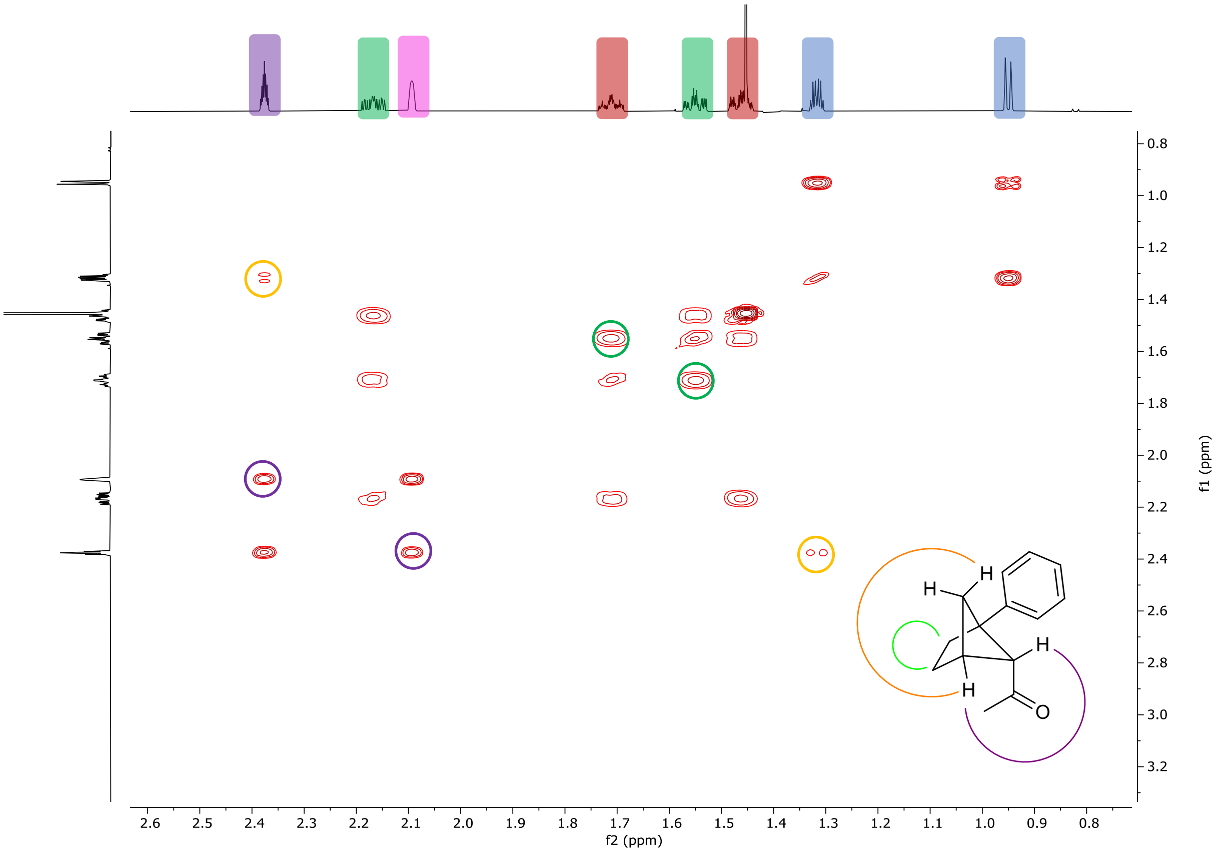

Figure S7: Assignment of proton to each carbon and the COSY interaction for the determination of the molecular structure.

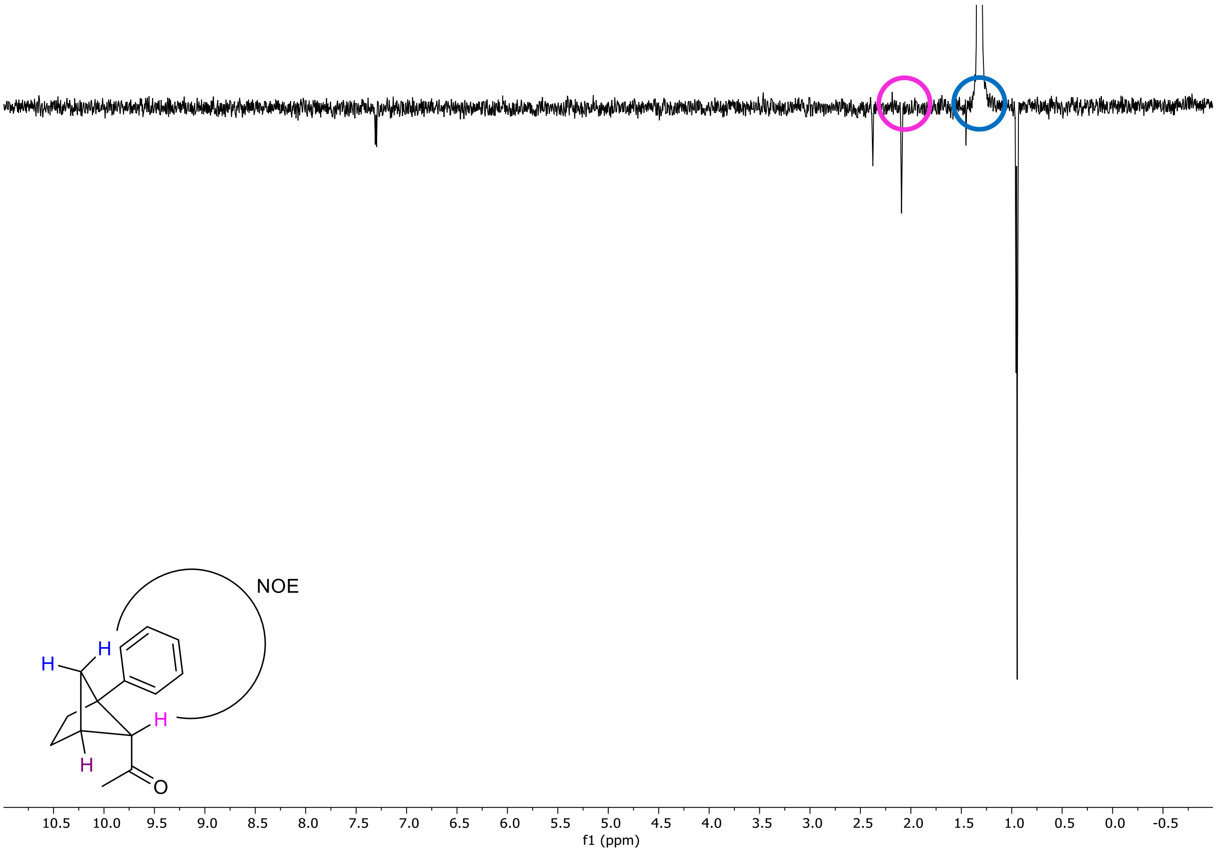

*Figure S8: Selected NOE interaction to determine the relative configuration of **2a***

7. Mechanistic experiments

7.1 UV-vis absorption spectra and emission spectra

The UV-vis absorption spectra were measured using a *Jasco V-730* with a spectral bandwith of 1.0 nm and a scan rate of 1000 nm/min. The spectra were recorded in a range of 200-600m and glass cuvettes with an optical length of 1 mm and 1 cm were used. The fluorescence emission spectra were recorded on a *Jasco Spectrafluorometer FP-8500* with a spectral bandwidth of 5.0 nm and a scan rate of 1000 nm/min. The samples were measured in glass cuvettes with an optical length of 1 cm.

The UV-vis spectra of both diastereomers of 4-phenylocta-3,7-dien-2-on were measured separately and as a 1:1 mixture in 4w% SDS. Each sample were 2 mM and were recorded in a glass cuvette with an optical length of 1 mm. The fluorescence emission spectra of pyrene in 4w% SDS were measured in a concentration of 200 µmol/L in a glass cuvette with an optical length of 1 cm. The sample was excited at 330 nm.

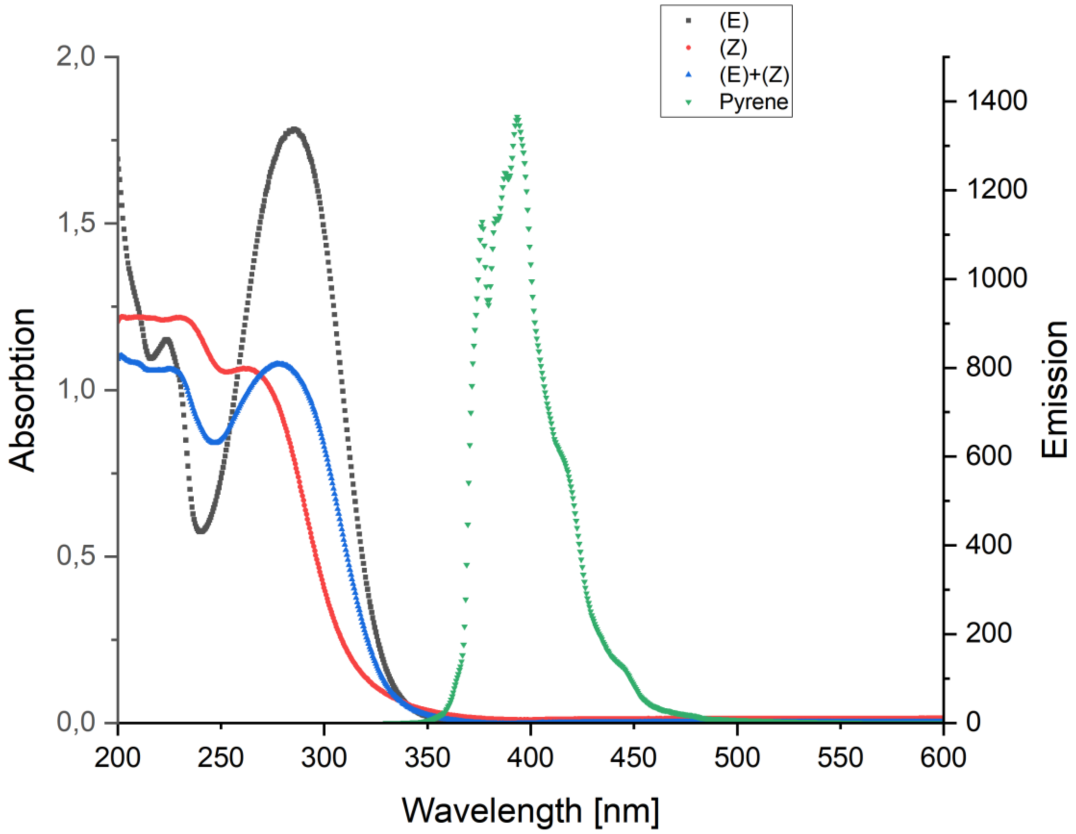

Figure S9: UV-Vis spectra of both diastereomers of ***1a*** *and the fluorescence emission spectrum of pyrene in a range of 200-600 nm.*

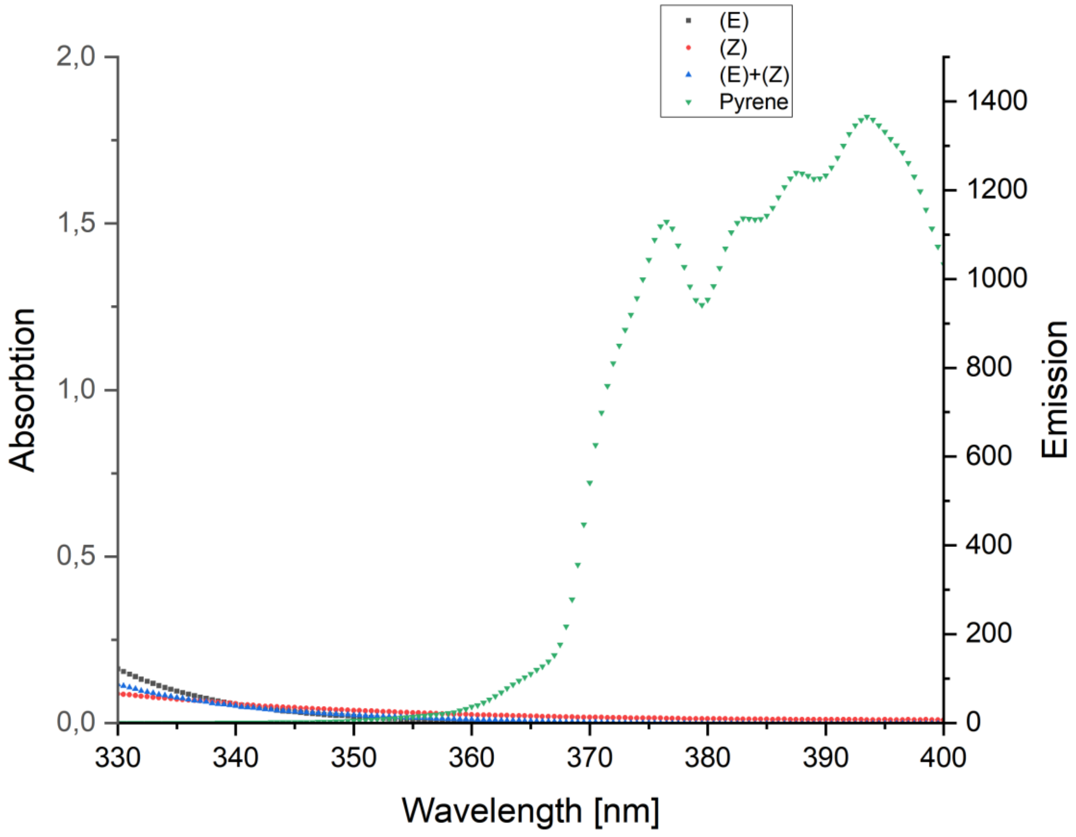

Figure S10: UV-Vis spectra of both diastereomers of ***1a*** *and the fluorescence emission spectrum of pyrene in a range of 330-400 nm.*

7.2 Luminescence quenching experiments

Luminescence quenching experiments were conducted on a *Jasco FP-8550* spectrofluorometer at 20°Cin *Hellma* fluorescence QS quartz cuvettes equipped with a PTFE stopper and a stirring bar. The concentration of $[Ru(bpy)_3](PF_6)_2$ and pyrene were kept at reaction conditions (0.66 mM and 5.0 mM respectively), with an aqueous SDS solution (4 w%) as solvent. The samples were excited at 450 nm and emission was recorded from 300 to 415 nm, and at 385 nm for the Stern-Volmer plot. The depicted spectra show the accumulated emission over ten cycles.

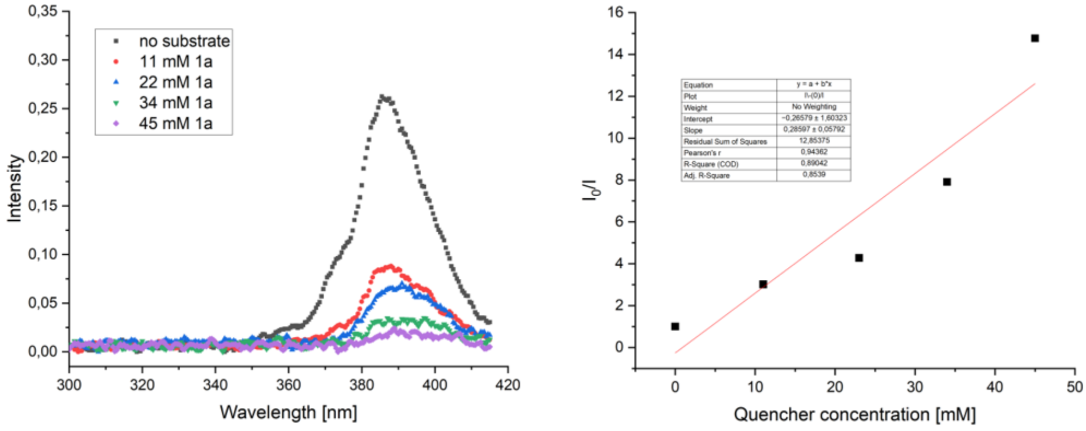

*Figure S11: Left: fluorescence spectra of [Ru(bpy)$_3$](PF$_6$)$_2$ and pyrene with varying amount of substrate **1a** as quencher; right: Stern-Volmer plot of emission at 385 nm.*

A weak fluorescence signal could be produced irradiating at 450 nm. The produced, weak fluorescence signal fits with pyrene monomer emission[5] and could be quenched in the presence of substrate **1a**. As expected, more substrate increases quenching. Using the above illustrated measurements as datapoints for a Stern-Volmer plot resulted in what appears to be a linear correlation with a somewhat low corrected correlation coefficient of 0.85. As the signal is of low intensity, the errors are more significant and the corrected correlation coefficient is lower.

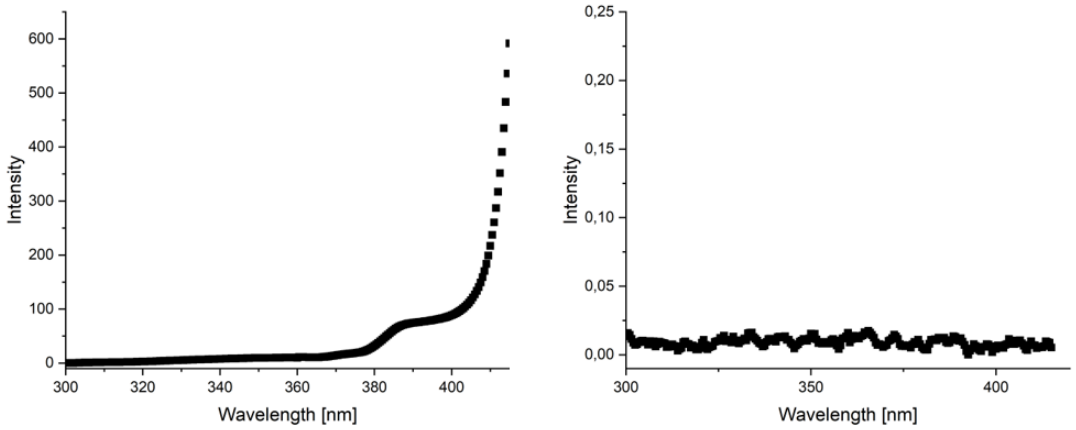

Figure S12: Left: Pyrene emission irradiated at 450 nm; right: [Ru(bpy)$_3$](PF$_6$)$_2$ emission irradiated at 450 nm.

The Ru(bpy)$_3^{2+}$ background spectrum shows no emission, whereas the spectrum of pyrene shows emission in the relevant region. While pyrene is not expected to absorb when irradiated at 450 nm, emission corresponding to pyrene excimer emission (peaking around 470 nm) and a smaller “shoulder” of monomer pyrene emission (around 390 nm) was recorded.[5] In the presence of Ru(bpy)$_3^{2+}$ we observe the monomer emission which could be quenched in the presence of substrate (see Figure S11).

Due to the observed excimer emission formed when irradiating at 450 nm, we performed control experiments to determine if this formation of excimer could promote the reaction. Our control experiments show that pyrene on its own cannot promote the reaction using 520 nm LEDs (0% NMR yield). We tested other wavelengths and observed 18% NMR yield using a 445 nm light source. With these observations in mind, we cannot use our experiments to confirm upconverted emission. However, we can use them to confirm pyrene quenching, which supports our proposed mechanism.

7.3 Cyclic voltammetry

Equipment

Cyclic voltammetry experiments were carried out in an air-tight three-electrode undivided measuring cell (rhd instruments, TSC 1600 closed) with an approximate sample volume of 1.0 mL, with a platinum disc (diameter 0.25 mm) as the working electrode and a platinum crucible as the counter electrode. A silver wire pseudo-reference electrode (rhd instruments, Ag wire MicroPseudo reference) was used. Measurements were performed using a Metrohm Autolab potentiostat (Metrohm, PGSTAT204) and data were collected and analysed using the Autolab Nova 2.1 program. Figures were prepared for publication using the Python libraries matplotlib and pandas.

Materials

Acetonitrile was obtained from Acros Organics in extra-dry grade, then degassed using three freeze-pump-thaw cycles and stored over activated molecular sieves (3 Å) under argon. Tetrabutylammonium hexafluorophosphate (>99.0%, for electrochemical analysis) was purchased from Sigma Aldrich. Ferrocene (high purity, 99+%) was purchased from Alfa Aesar. All chemicals were used without further purification.

Experimental

Experiments were performed on 1 mL of a 2 mM solution of the analyte in acetonitrile, with 0.1 M $[Bu_4N^+][PF_6^-]$ as the supporting electrolyte, at a scan rate of 100 mV s−1. The starting potential was taken as the measured open circuit potential, which in both cases was close to 0 V vs the Ag pseudo reference electrode. For both depicted CVs, the initial scanning direction was towards positive potentials; scanning in the reverse direction did not reveal significantly different behaviour.

Samples were prepared as follows: the solids were weighed and dried in a vial on a Schlenk line before filling the headspace with argon, and bringing into an argon-filled glovebag. The working electrode of the cell was polished before use by making figure-of-eight motions in a water-alumina (0.05 µm) slurry on a microfibre polishing pad, after which alumina residues were removed by sonication and rinsing with deionized water. The solids were dissolved in dry, degassed acetonitrile, and transferred to the cell under argon in the glovebag. After successful measurement the cell was returned to the glovebag and reopened, a small quantity of a stock solution of ferrocene was added, and the sample with the internal reference

remeasured with a fresh working electrode. All potentials were then referenced to the potential of the Fc/Fc^+ redox couple. Potentials are also provided referenced to SCE for convenience, for which a conversion factor of +0.380 V for Fc/Fc^+ vs SCE has been used.[6]

Standard potentials E° of reversible redox events, or irreversible events where both anodic and cathodic peaks were discernible, were estimated as usual using half-wave potentials $E_{½}$. For irreversible peaks, the inflection point of the curve was instead used as the best estimate of the standard potential.[7] Voltages were not compensated for IR drop.

Results and Discussion

Both isomers exhibit very similar redox behaviour. In each case the CV features an irreversible oxidation at around +1.7 V (vs Fc/Fc^+) and an at least partially reversible reduction at around −2.2 V (vs Fc/Fc^+). As the reduction event lies close to the electrochemical window of the solvent, it is difficult to ascertain whether the reduction is truly reversible or not. By inspection, the peak current of the oxidation wave is approximately double that of the reduction wave, suggesting that the oxidation event is likely a two-electron oxidation.

The reduction of (*Z*)-**1a** shows interesting kinetic behaviour, featuring not the typical CV waveform but instead a very late onset and a sudden jump when the estimated E° is reached. This behaviour was consistent across multiple measurements.

For (*Z*)-**1a**, the reduction potential was estimated using the average of the two peak potentials. For (*E*)-**1a**, this was not possible, so the reduction potential was estimated using the average of the inflection points in each direction. In both cases, the reduction peak was used to reference the potentials vs ferrocene (not shown).

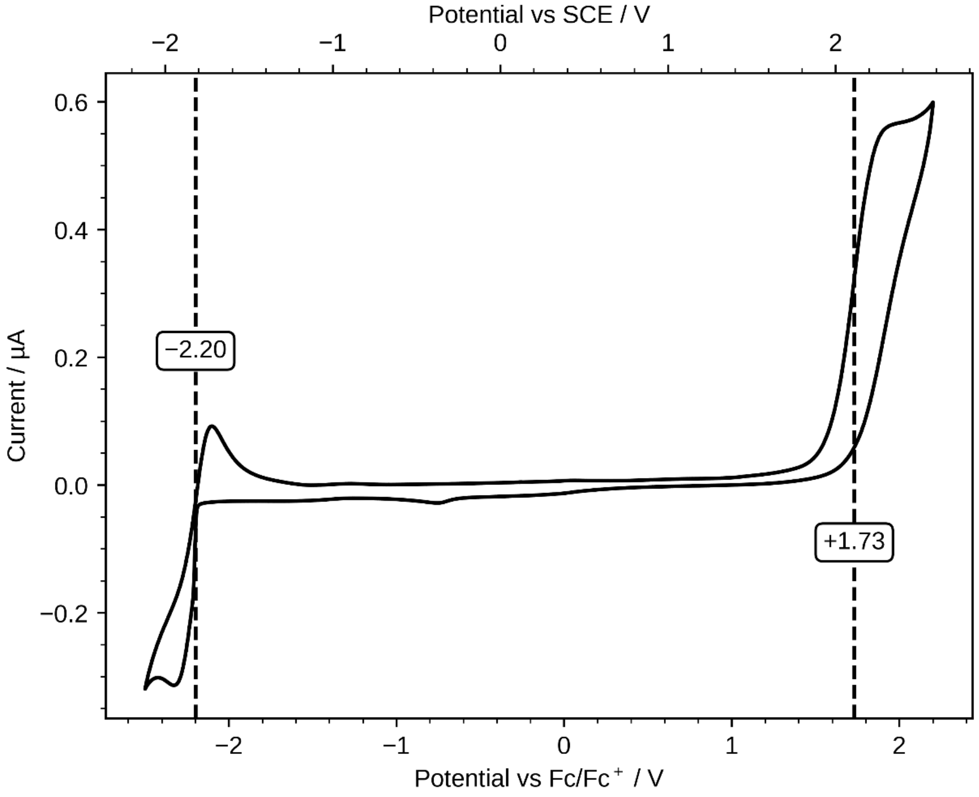

Figure S7: CV of (Z)-1a (2 mM) in acetonitrile with $[Bu_4N^+][PF_6^-]$ (0.1 M) as the supporting electrolyte, at a scan rate of 100 mV s^{-1}, with a 0.25 mm diameter Pt working electrode. Potentials were referenced separately to the Fc/Fc^+ redox couple using ferrocene as an internal standard.

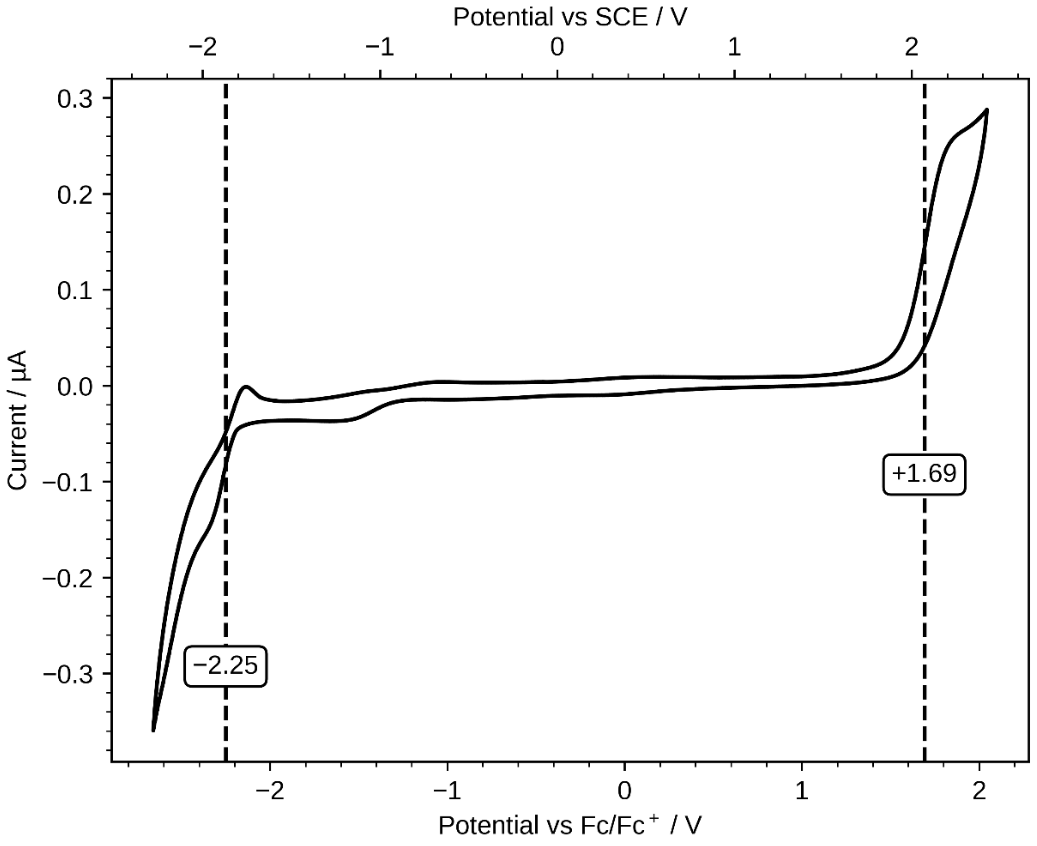

Figure S8: CV of (E)-1a (2 mM) in acetonitrile with $[Bu_4N^+][PF_0^-]$ (0.1 M) as the supporting electrolyte, at a scan rate of 100 mV s−1, with a 0.25 mm diameter Pt working electrode. Potentials were referenced separately to the Fc/Fc^+ redox couple using ferrocene as an internal standard.

7.4 Mechanistic investigations

To indicate whether triplet-triplet annihilation (TTA) or sensitization-initiated electron transfer (SenI-ET) is more likely to take place, experiments with DIPEA as an addititve, as well as *fac*-Ir(ppy)$_3$ as alternative sensitizer were conducted. DIPEA is capable of reducing Ru(bpy)$_3^{2+}$ thereby possibly diminishing the TTA pathway, which should lead to decreased product formation. DIPEA has been shown by Wenger et al[8] to not be able to reduce *fac*-Ir(ppy)$_3$. Therefore the underlying mechanistic pathway should not be affected by DIPEA addition in a major way.

(*Z*)-4-phenylocta-3,7-dien-2-on (40.1 mg, 0.20 mmol, 1.0 eq.), Ru(bpy)$_3$(PF$_6$)$_2$ (3.4 mg, 4.0 µmol, 2.0 mol%) or *fac*-Ir(ppy)$_3$ (2.6 mg, 4.0µmol, 2.0 mol%), pyrene (6.1 mg, 0.03 mmol, 15 mol%) DIPEA and SDS solution (240 mg in 5.76 mL H_2O) were added to a headspace vial equipped with a magnetic stirring bar. The reaction was stirred until a homogeneous solution was observed. Afterwards the reaction mixture was irradiated with a suitable light source (*fac*-Ir(ppy)$_3$: 445 nm for 15 h and Ru(bpy)$_3$(PF$_6$)$_2$: 520 nm for 24 h) at 20 C. The reaction mixture was transferred to a separatory funnel and dichloromethane and saturated aqueous

NaCl solution were added. The phases were separated and the aqueous phase was extracted six times with dichloromethane and the combined organic phases were dried over $MgSO_4$. After removing the solvent under reduced pressure, toluene was added as internal standard and the yield was determined via NMR. In addition, control experiment with only *fac*-Ir(ppy)$_3$ was performed under the above mentioned procedure, which formed the desired bicycle **2a** in 28% NMR yield.

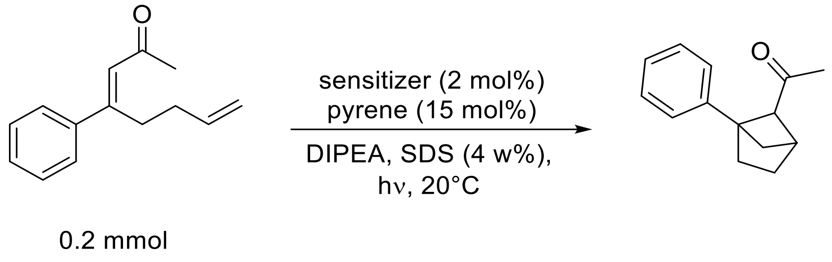

Figure S2: DIPEA as an electron donor additive can turn off the reaction.

Table S1: DIPEA experiments; n.p = not performed.

DIPEA	[Ru] as sens. (NMR yield)	[Ir] as sens. (NMR yield)
0 equiv.	76%	64%
1.4 equiv.	67%	n.p
2.0 equiv.	60%	65%
5.0 equiv.	25%	55%
10.0 equiv.	0%	n.p

8. X-Ray Diffraction Analysis

X-Ray diffraction: Data sets for compounds **6a** was collected with a Bruker D8 Venture Photon III Diffractometer. Programs used: data collection: *APEX4* Version 2021.4-0[9] (Bruker AXS Inc., **2021**); cell refinement: *SAINT* Version 8.40B (Bruker AXS Inc., **2021**); data reduction: *SAINT* Version 8.40B (Bruker AXS Inc., **2021**); absorption correction, *SADABS* Version 2016/2 (Bruker AXS Inc., **2021**); structure solution *SHELXT*-Version 2018-3[10] (Sheldrick, G. M. *Acta Cryst.,* **2015**, *A71*, 3-8); structure refinement *SHELXL*- Version 2018-3[11] (Sheldrick, G. M. *Acta Cryst.,* **2015**, *C71* (1), 3-8) and graphics, *XP* [12] (Version 5.1, Bruker AXS Inc., Madison, Wisconsin, USA, **1998**). *R*-values are given for observed reflections, and wR^2 values are given for all reflections.

X-ray crystal structure analysis of 6a: A colorless, prism-like specimen of $C_{25}H_{22}ClNO_4$, approximate dimensions 0.153 mm x 0.207 mm x 0.257 mm, was used for the X-ray crystallographic analysis. The X-ray intensity data were measured on a single crystal diffractometer Bruker D8 Venture Photon III system equipped with a micro focus tube Mo ImS (MoKα, λ = 0.71073 Å) and a MX mirror monochromator. A total of 1096 frames were collected. The total exposure time was 4.19 hours. The frames were integrated with the Bruker SAINT software package using a narrow-frame algorithm. The integration of the data

using a monoclinic unit cell yielded a total of 54666 reflections to a maximum θ angle of 26.74° (0.79 Å resolution), of which 4409 were independent (average redundancy 12.399, completeness = 99.8%, R_{int} = 4.41%, R_{sig} = 1.99%) and 3915 (88.80%) were greater than 2σ(F^2). The final cell constants of a = 12.4061(4) Å, b = 15.0978(5) Å, c = 11.6059(3) Å, β = 106.8060(10)°, volume = 2080.99(11) $Å^3$, are based upon the refinement of the XYZ-centroids of 9964 reflections above 20 σ(I) with 4.552° < 2θ < 53.47°. Data were corrected for absorption effects using the Multi-Scan method (SADABS). The ratio of minimum to maximum apparent transmission was 0.957. The calculated minimum and maximum transmission coefficients (based on crystal size) are 0.9460 and 0.9680. The structure was solved and refined using the Bruker SHELXTL Software Package, using the space group $P2_1/c$, with Z = 4 for the formula unit, $C_{25}H_{22}ClNO_4$. The final anisotropic full-matrix least-squares refinement on F^2 with 284 variables converged at R1 = 3.08%, for the observed data and wR2 = 8.04% for all data. The goodness-of-fit was 1.065. The largest peak in the final difference electron density synthesis was 0.315 $e^-/Å^3$ and the largest hole was -0.219 $e^-/Å^3$ with an RMS deviation of 0.039 $e^-/Å^3$. On the basis of the final model, the calculated density was 1.391 g/cm^3 and F(000), 912 e^-. CCDC Nr.: 2264151.

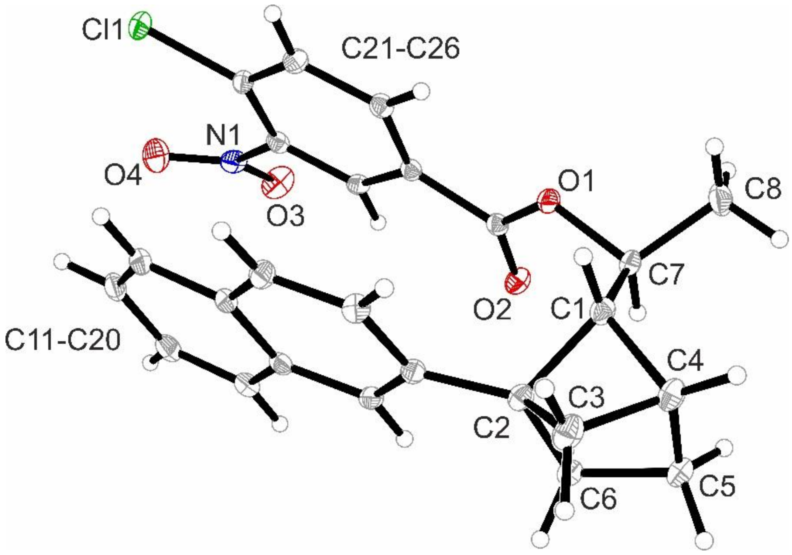

Figure S9: Crystal structure of compound 6a. Thermal ellipsoids are shown at 30% probability.

9. NMR Spectra

^{1}H-NMR (300 MHz, $CDCl_3$) of 1-Phenylpent-4-en-1-one (**3a**)

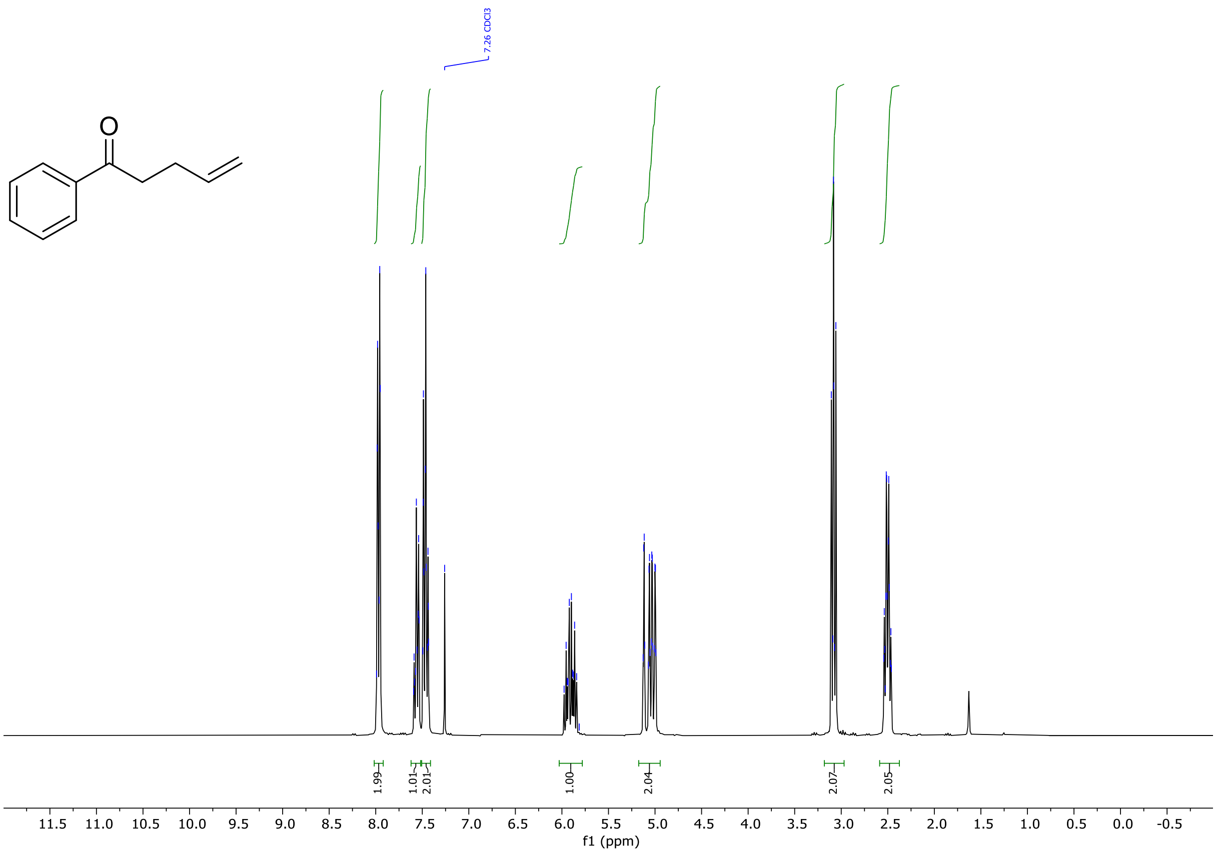

^{13}C-NMR (75 MHz, $CDCl_3$) of 1-Phenylpent-4-en-1-one (**3a**)

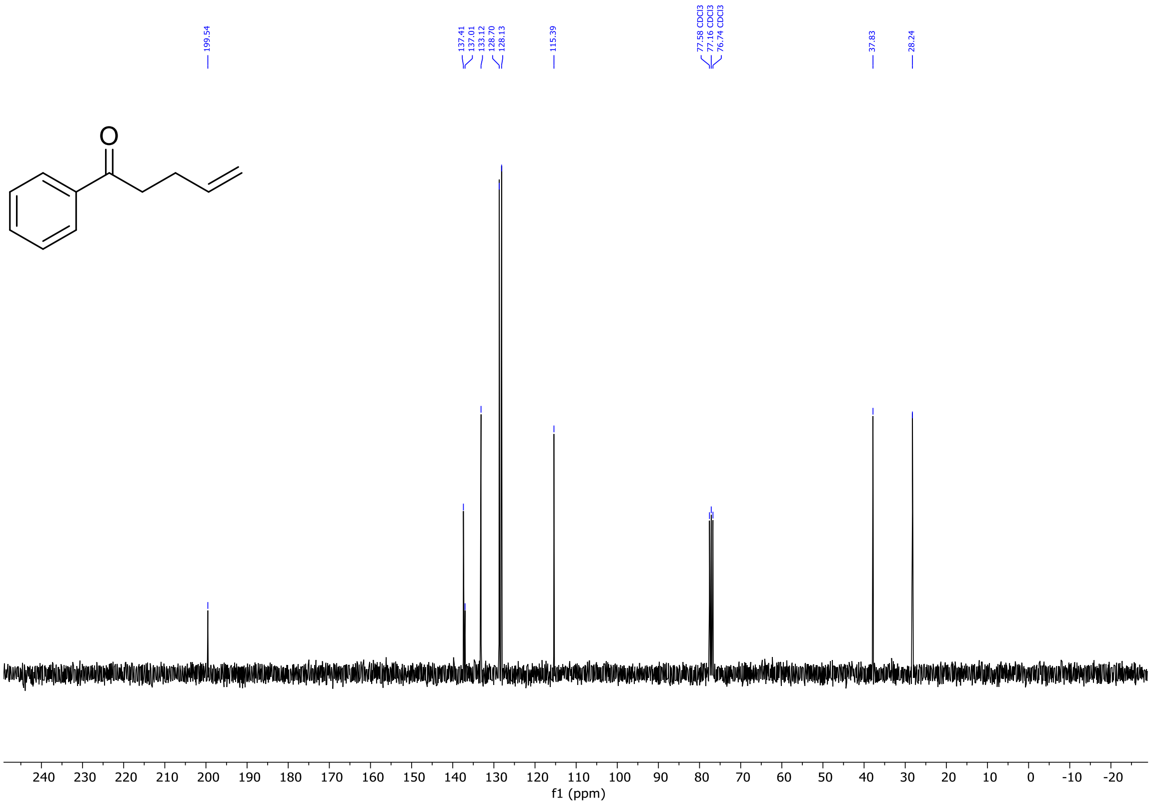

^{1}H-NMR (300 MHz, $CDCl_3$) of 1-(4-Fluorophenyl)pent-4-en-1-one (**3b**)

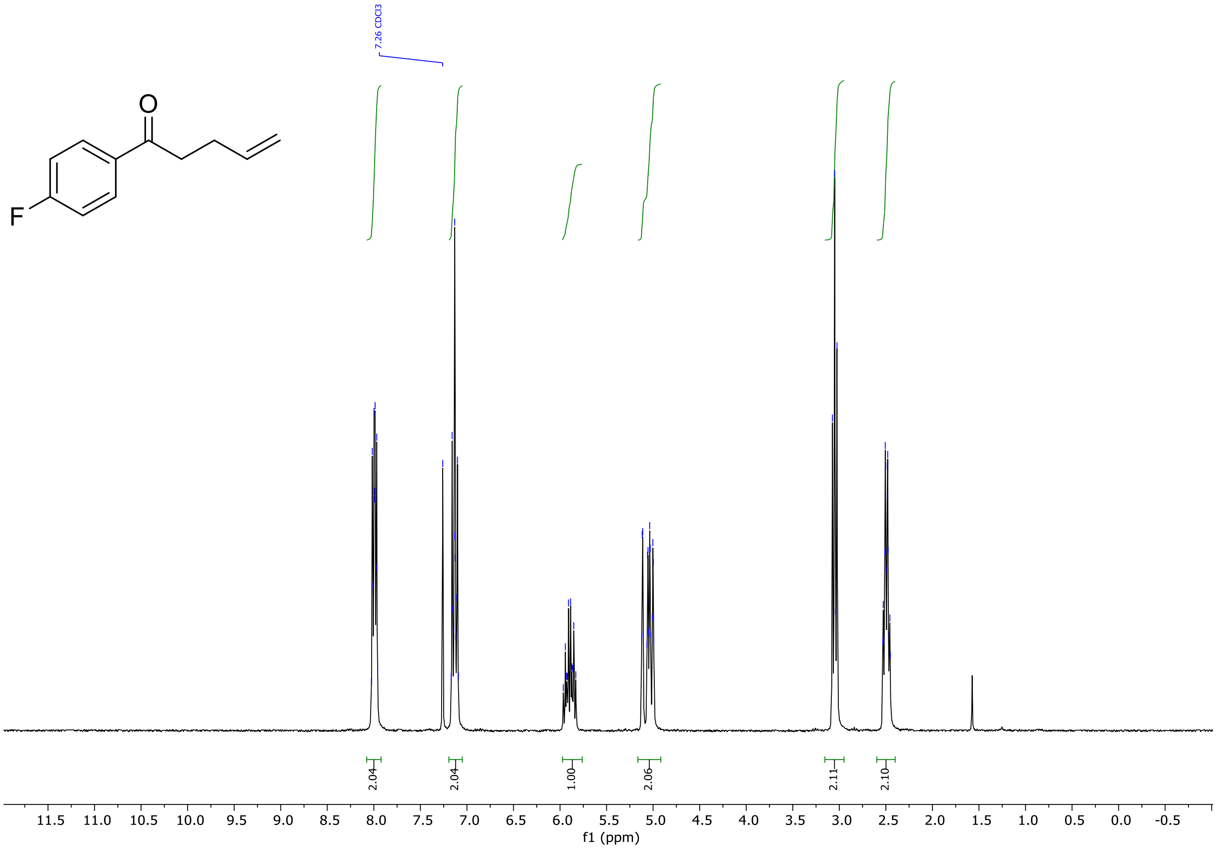

^{13}C-NMR (75 MHz, $CDCl_3$) of 1-(4-Fluorophenyl)pent-4-en-1-one (**3b**)

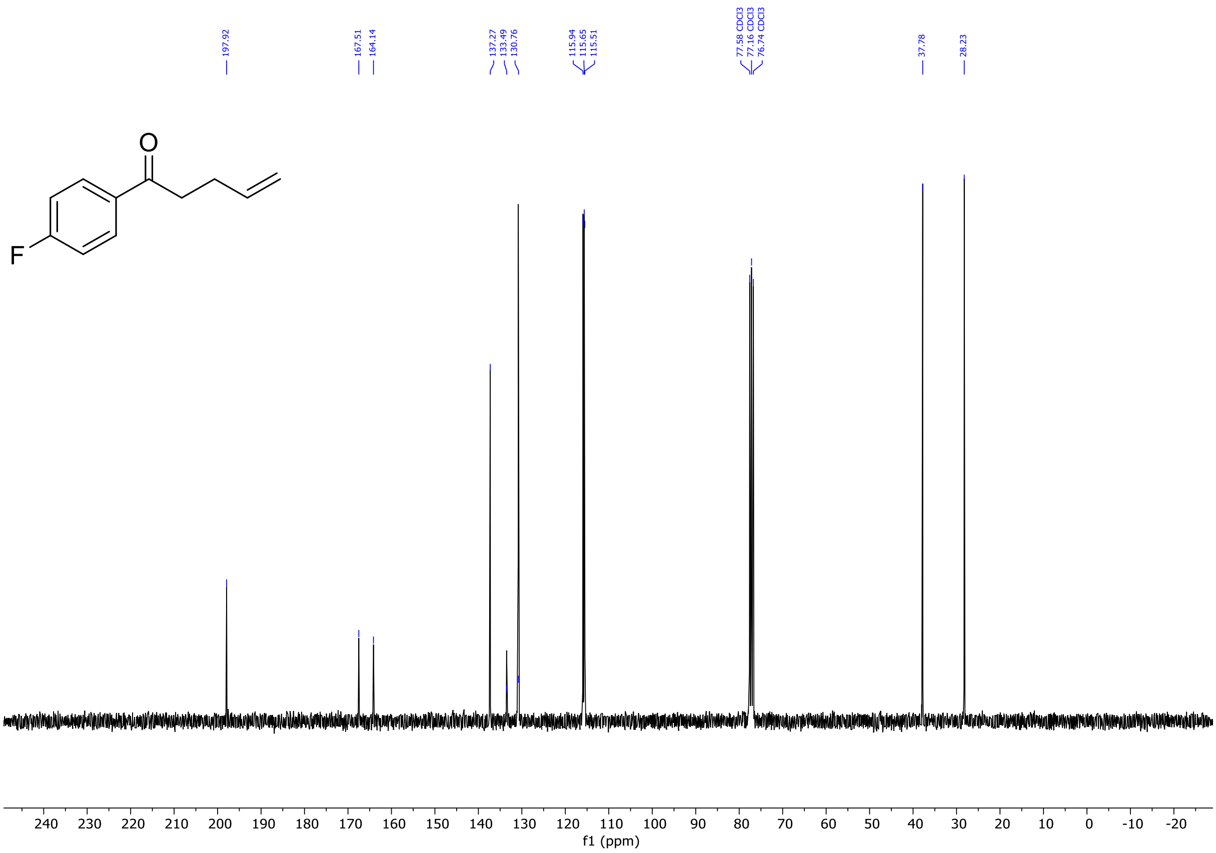

^{19}F-NMR (282 MHz, $CDCl_3$) of 1-(4-Fluorophenyl)pent-4-en-1-one (**3b**)

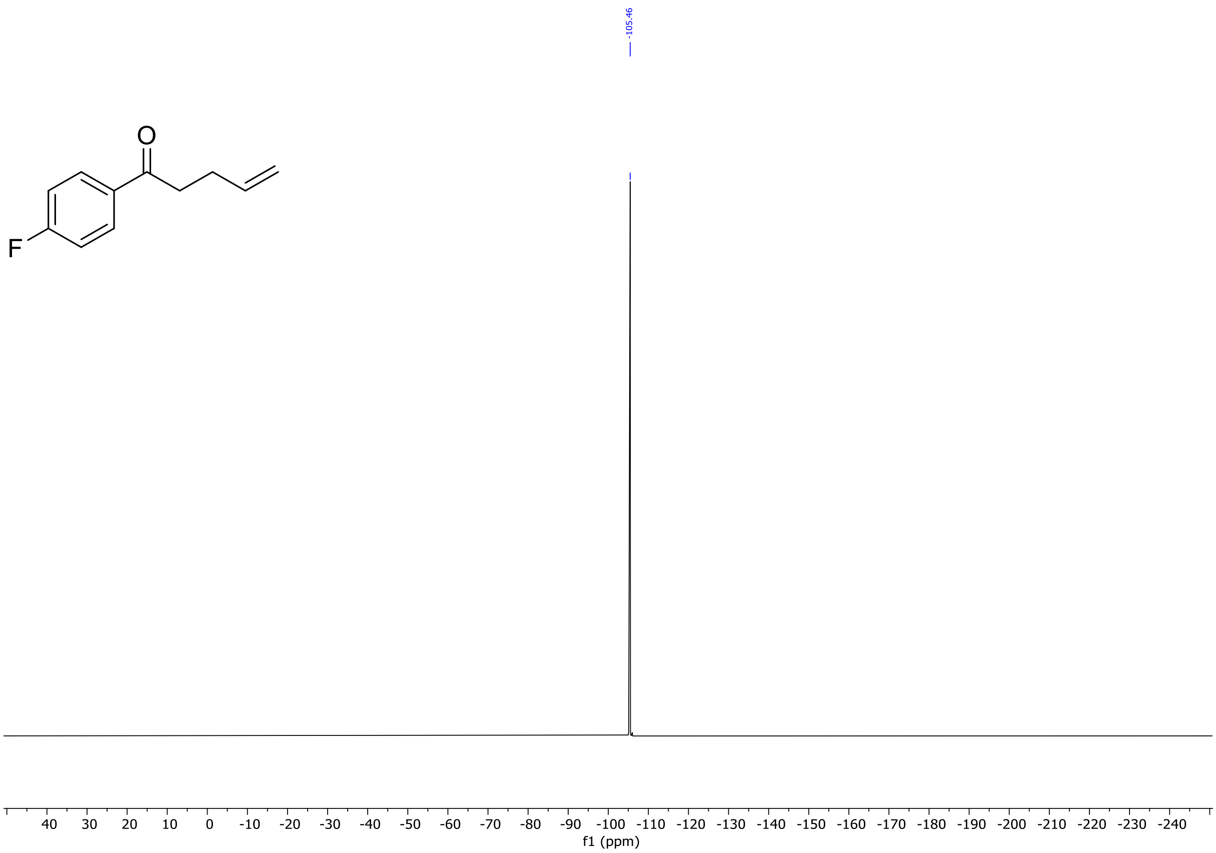

^{1}H-NMR (300 MHz, $CDCl_3$) of 1-(4-Chlorophenyl)pent-4-en-1-one (**3c**)

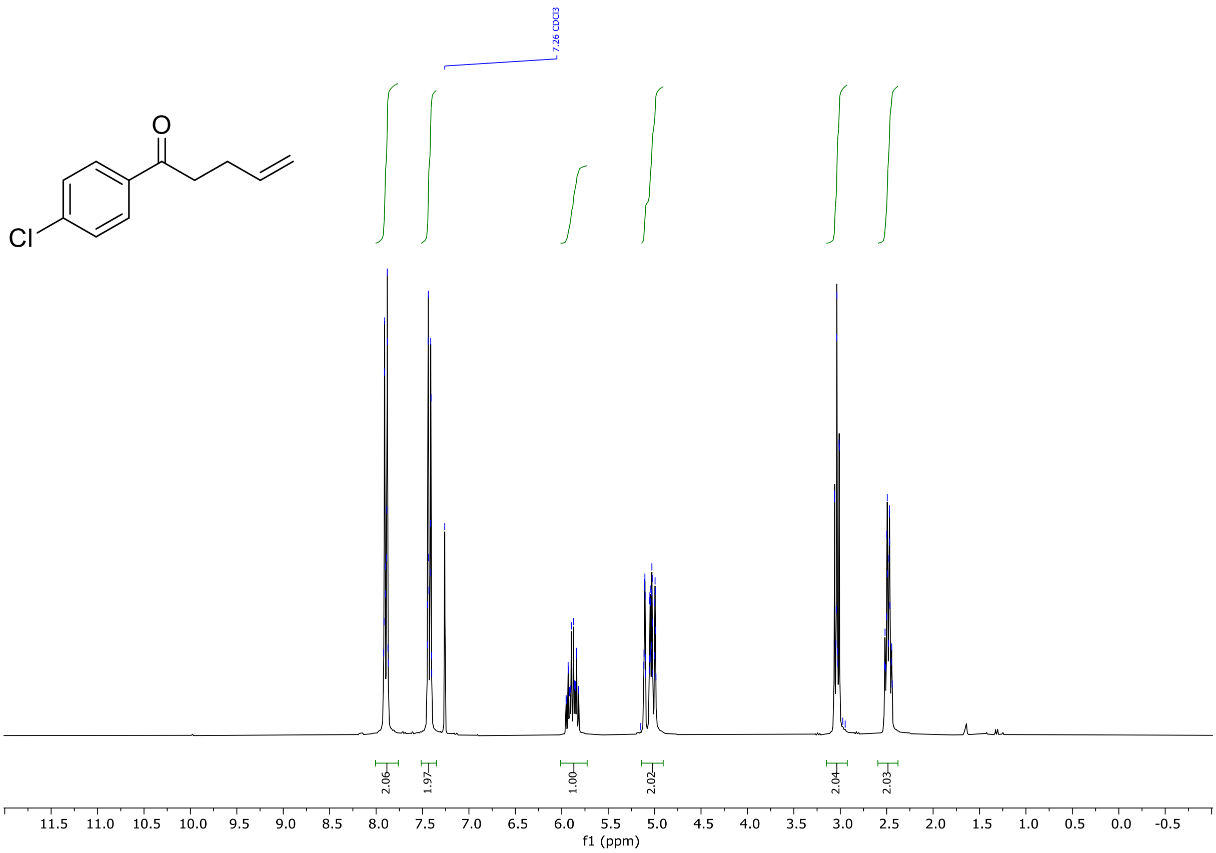

^{13}C-NMR (75 MHz, $CDCl_3$) of 1-(4-Chlorophenyl)pent-4-en-1-one (**3c**)

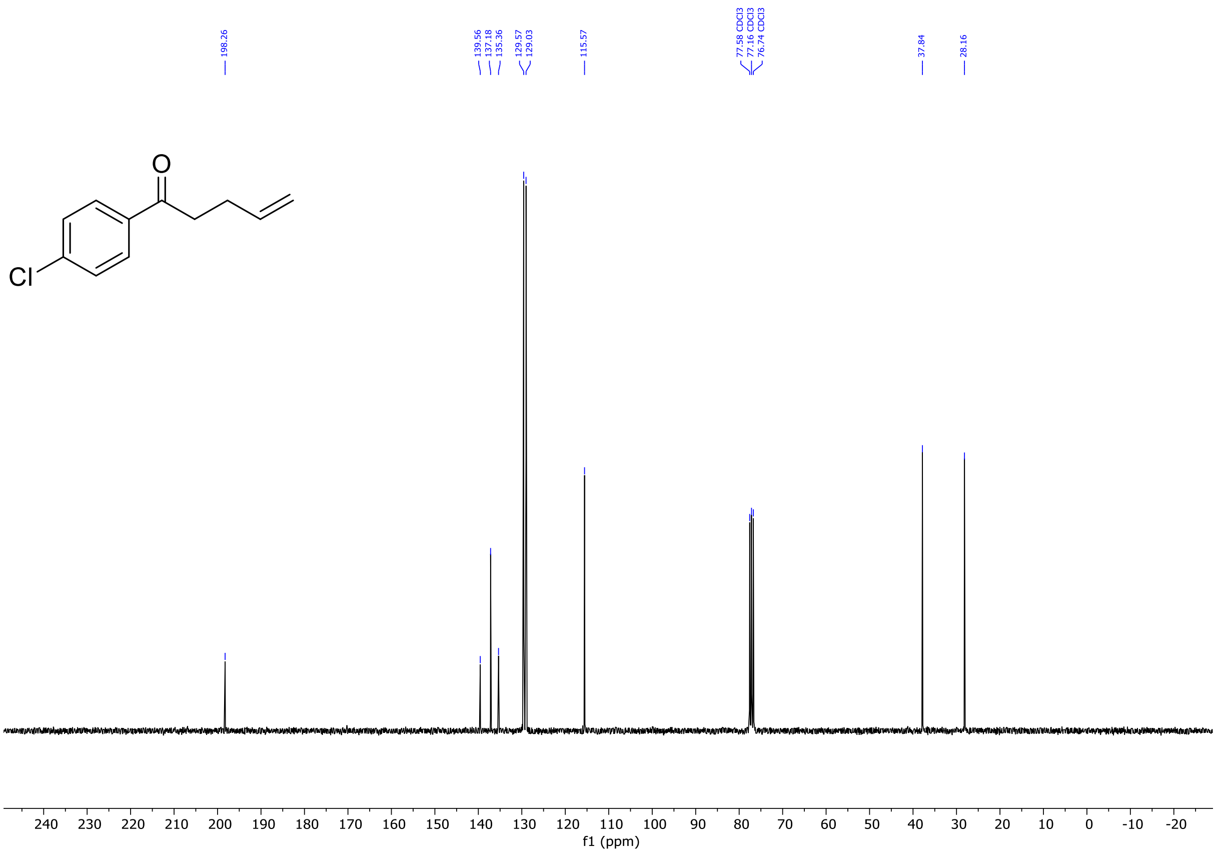

^{1}H-NMR (300 MHz, $CDCl_3$) of 1-(4-Bromophenyl)pent-4-en-1-one (**3d**)

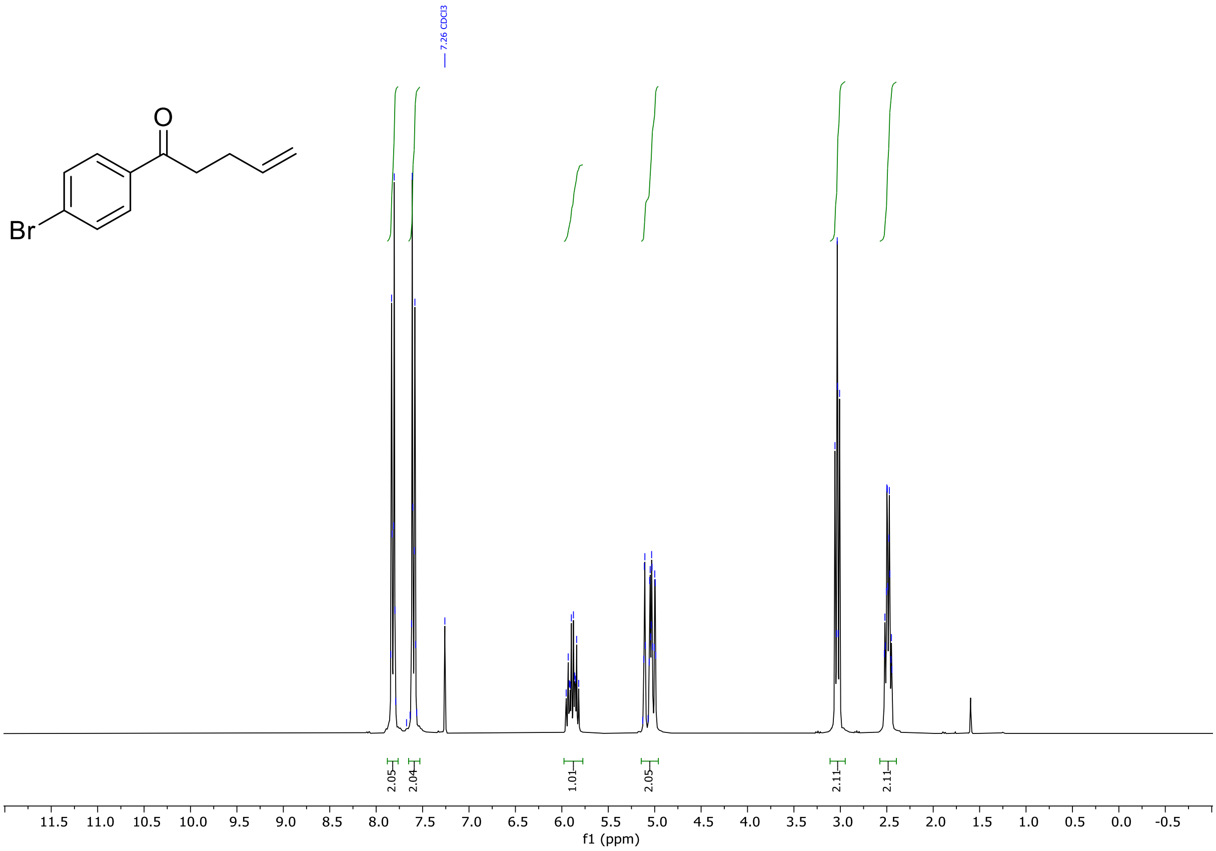

^{13}C-NMR (75 MHz, $CDCl_3$) of 1-(4-Bromophenyl)pent-4-en-1-one (**3d**)

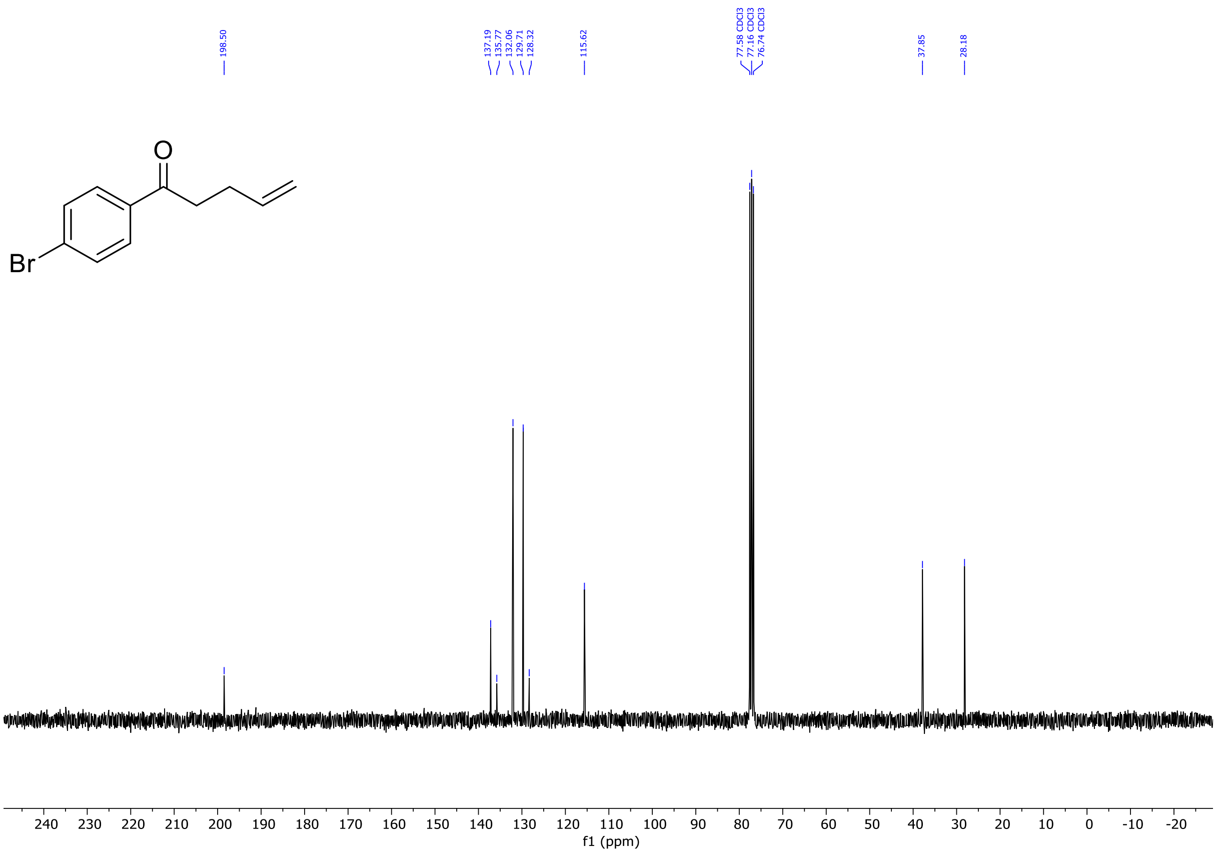

^{1}H-NMR (300 MHz, $CDCl_3$) of 1-(4-(Trifluoromethyl)phenyl)pent-4-en-1-one (**3e**)

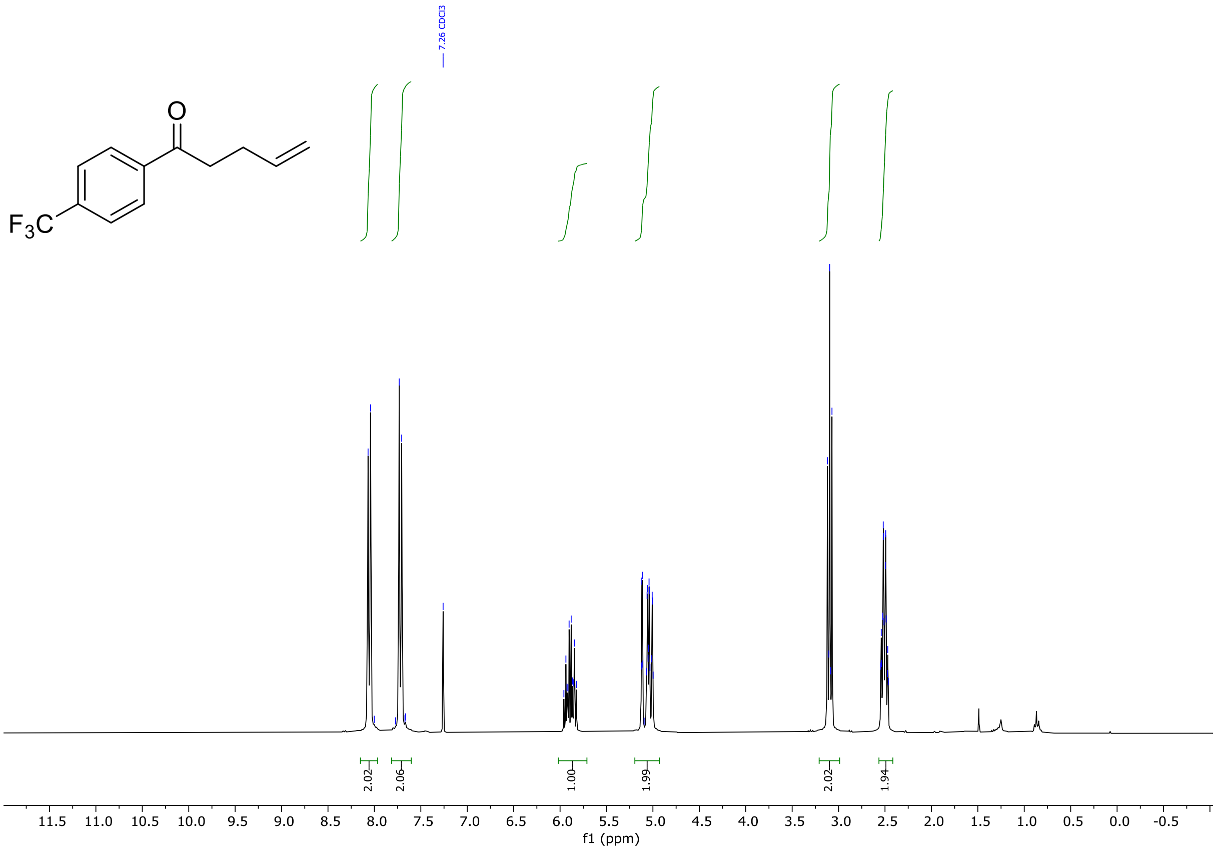

^{13}C-NMR (75 MHz, $CDCl_3$) of 1-(4-(Trifluoromethyl)phenyl)pent-4-en-1-one (**3e**)

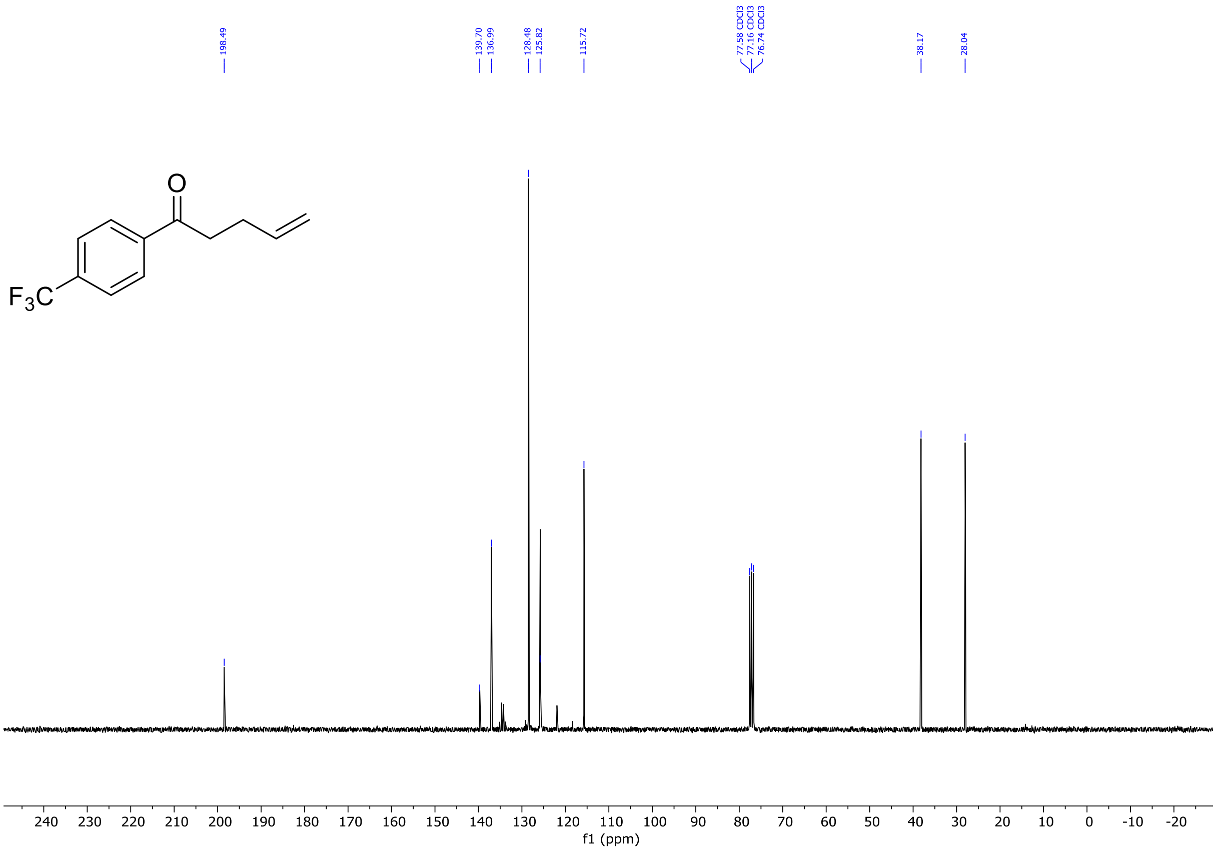

^{19}F-NMR (282 MHz, $CDCl_3$) of 1-(4-(Trifluoromethyl)phenyl)pent-4-en-1-one (**3e**)

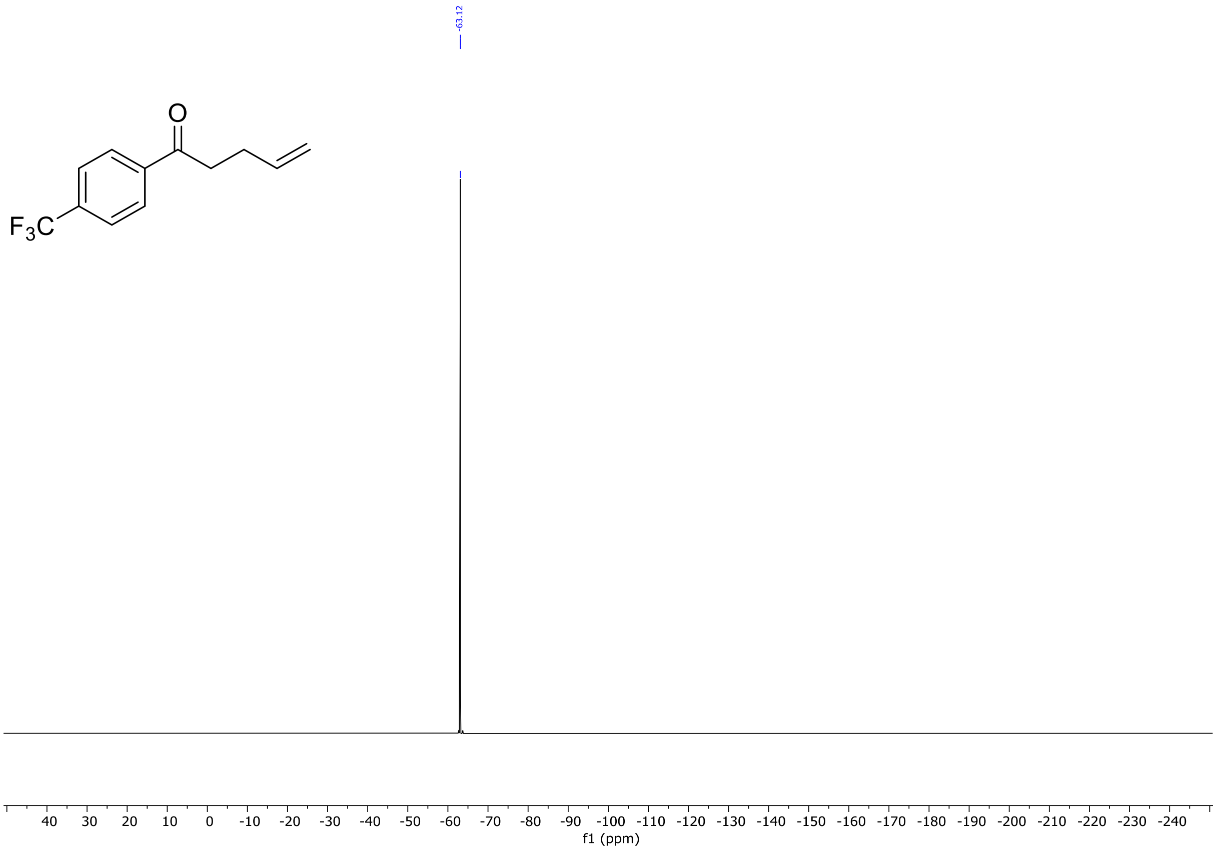

^{1}H-NMR (300 MHz, $CDCl_3$) of 1-(*p*-tolyl)pent-4-en-1-one (**3f**)

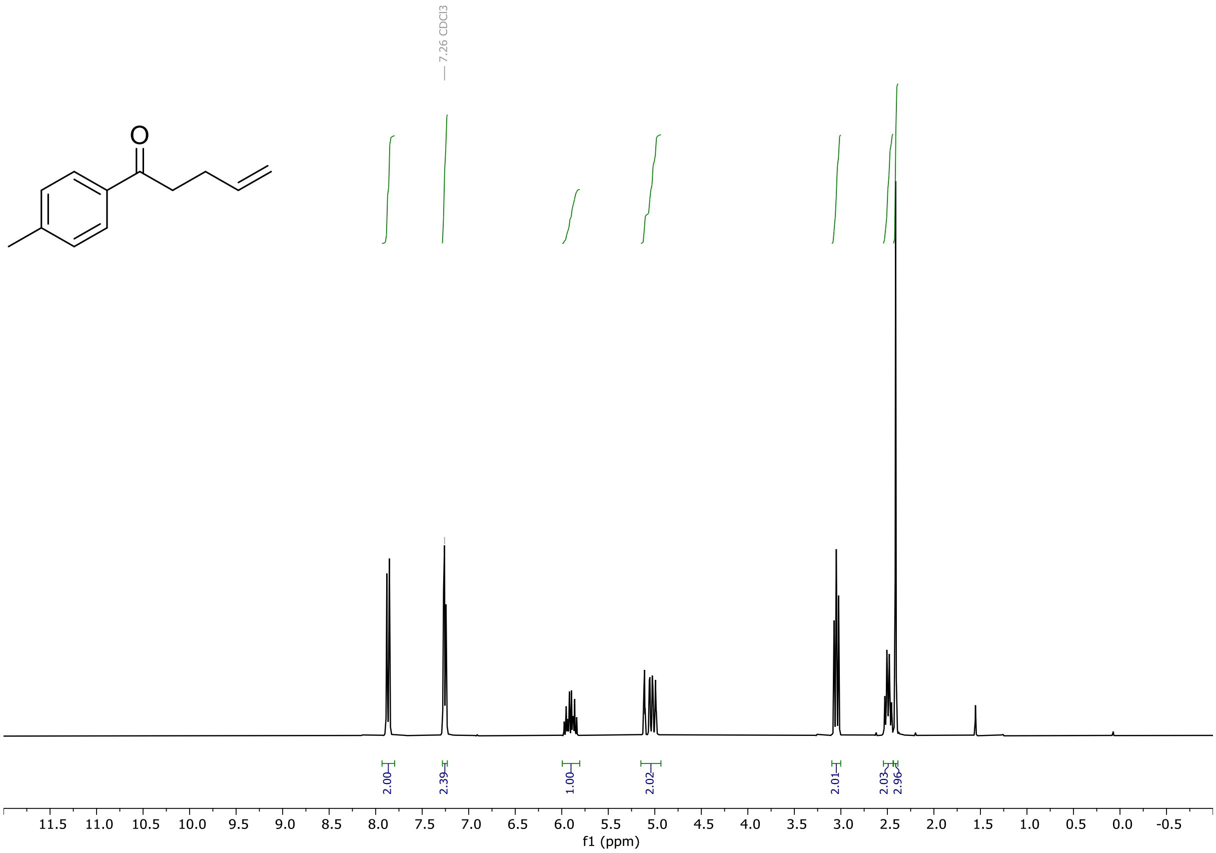

^{13}C-NMR (75 MHz, $CDCl_3$) of 1-(*p*-tolyl)pent-4-en-1-one (**3f**)

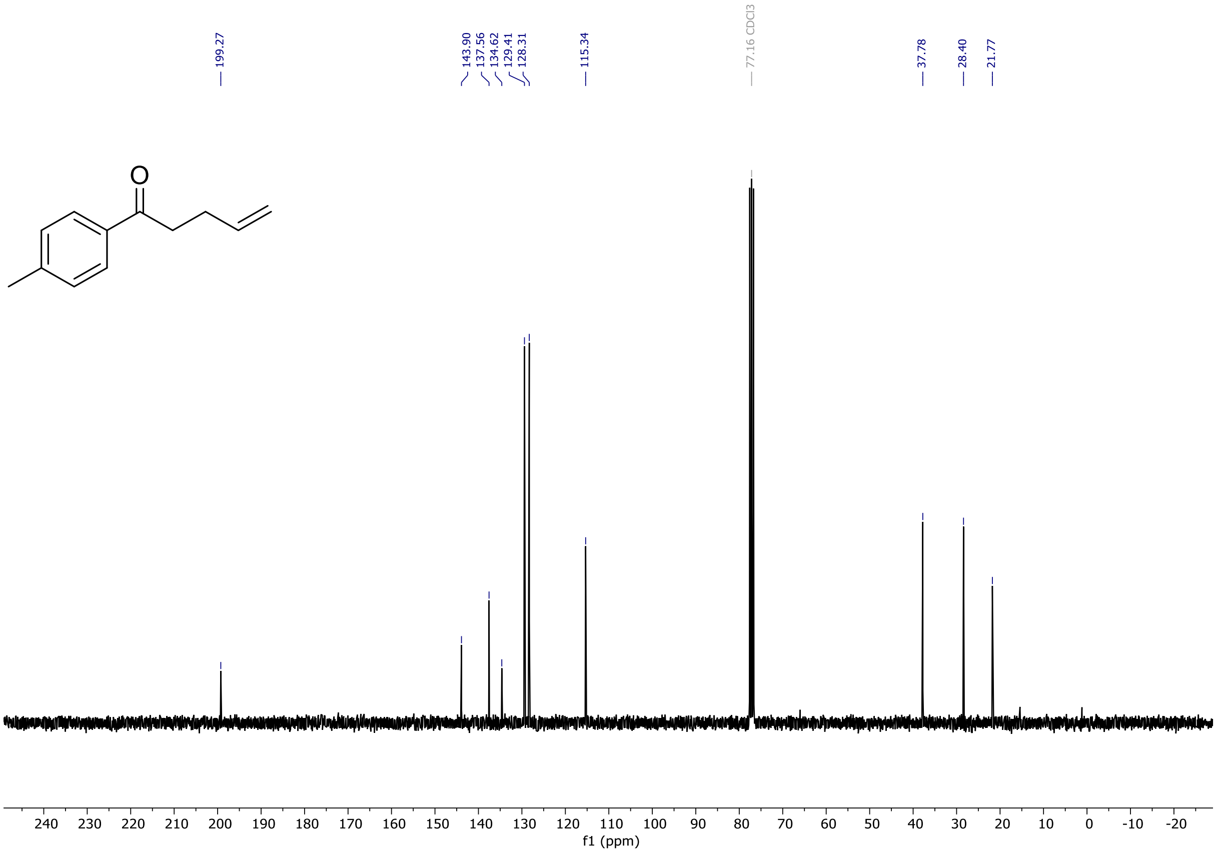

^{1}H-NMR (300 MHz, $CDCl_3$) of 1-(*m*-Tolyl)pent-4-en-1-one (**3g**)

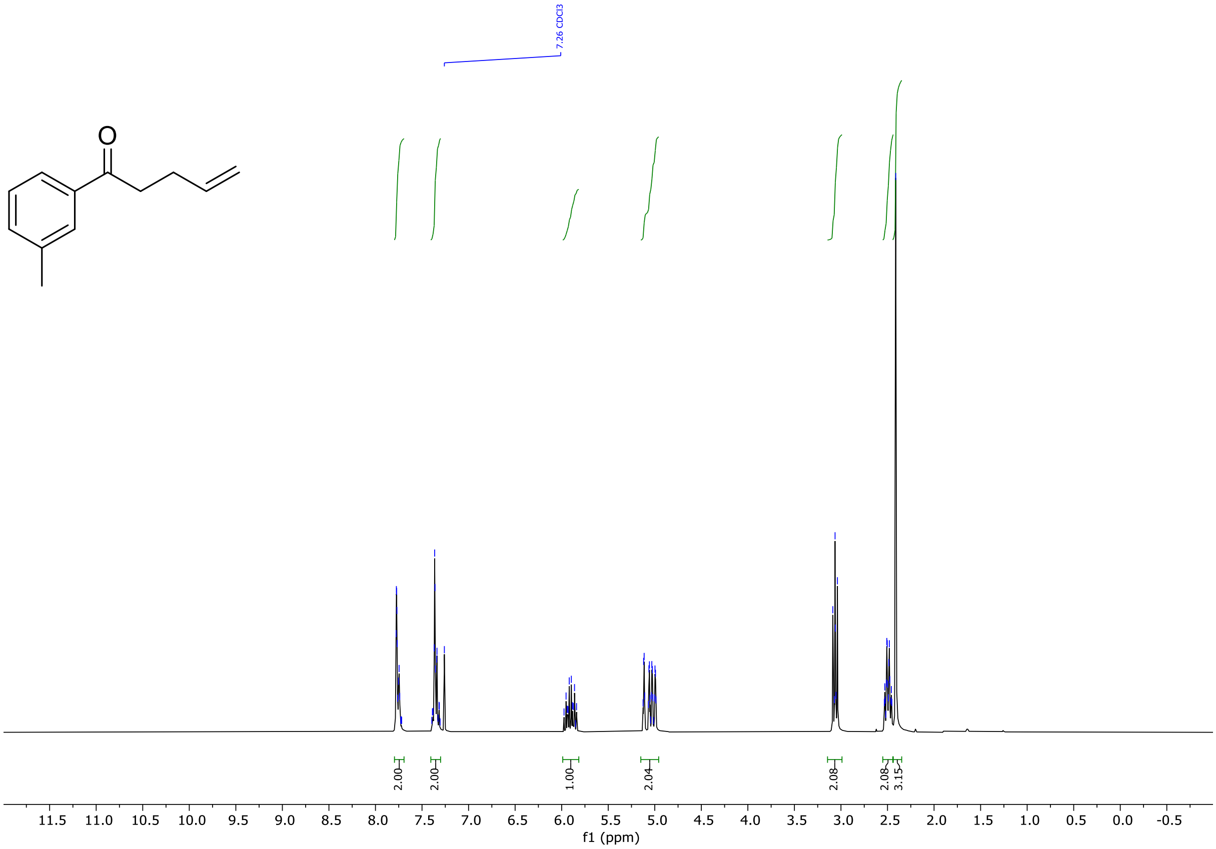

^{13}C-NMR (75 MHz, $CDCl_3$) of 1-(*m*-Tolyl)pent-4-en-1-one (**3g**)

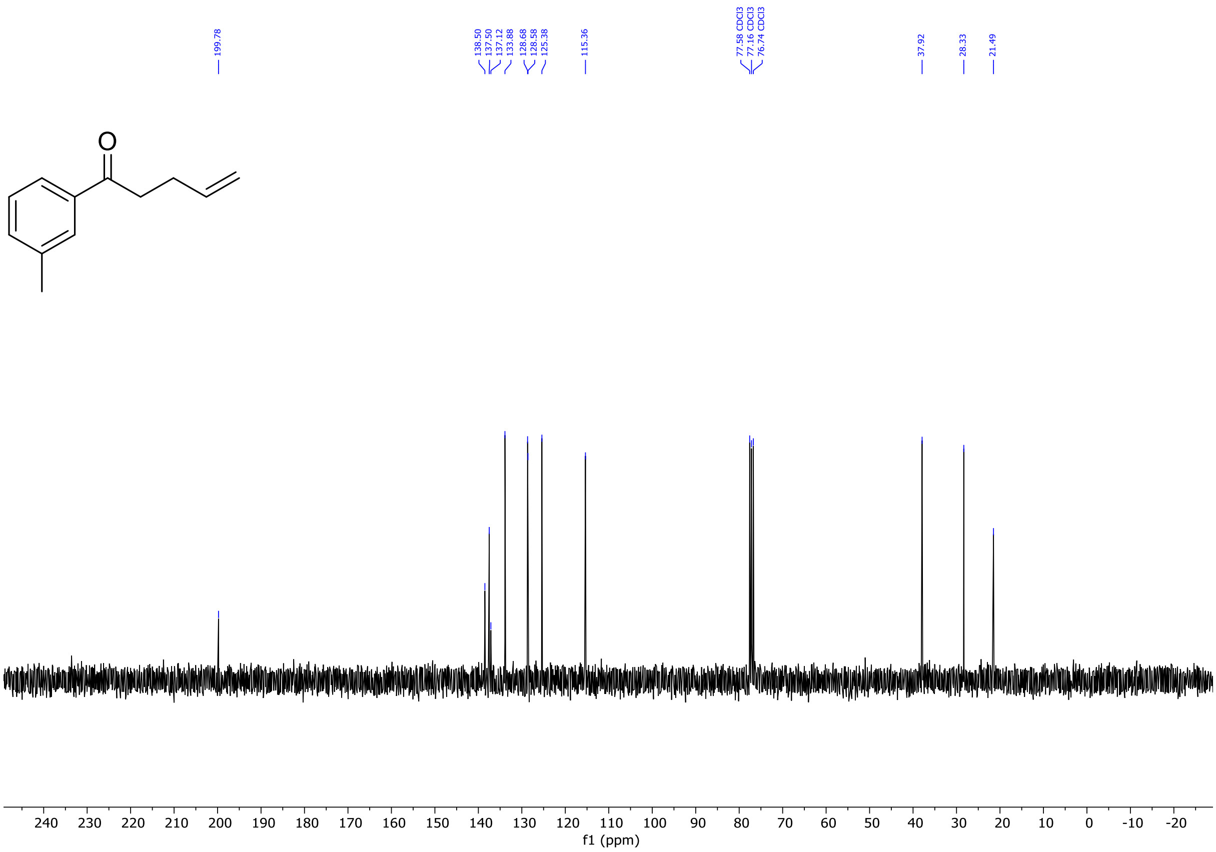

^{1}H-NMR (300 MHz, $CDCl_3$) of 1-(naphthalen-2-yl)pent-4-en-1-one (**3h**)

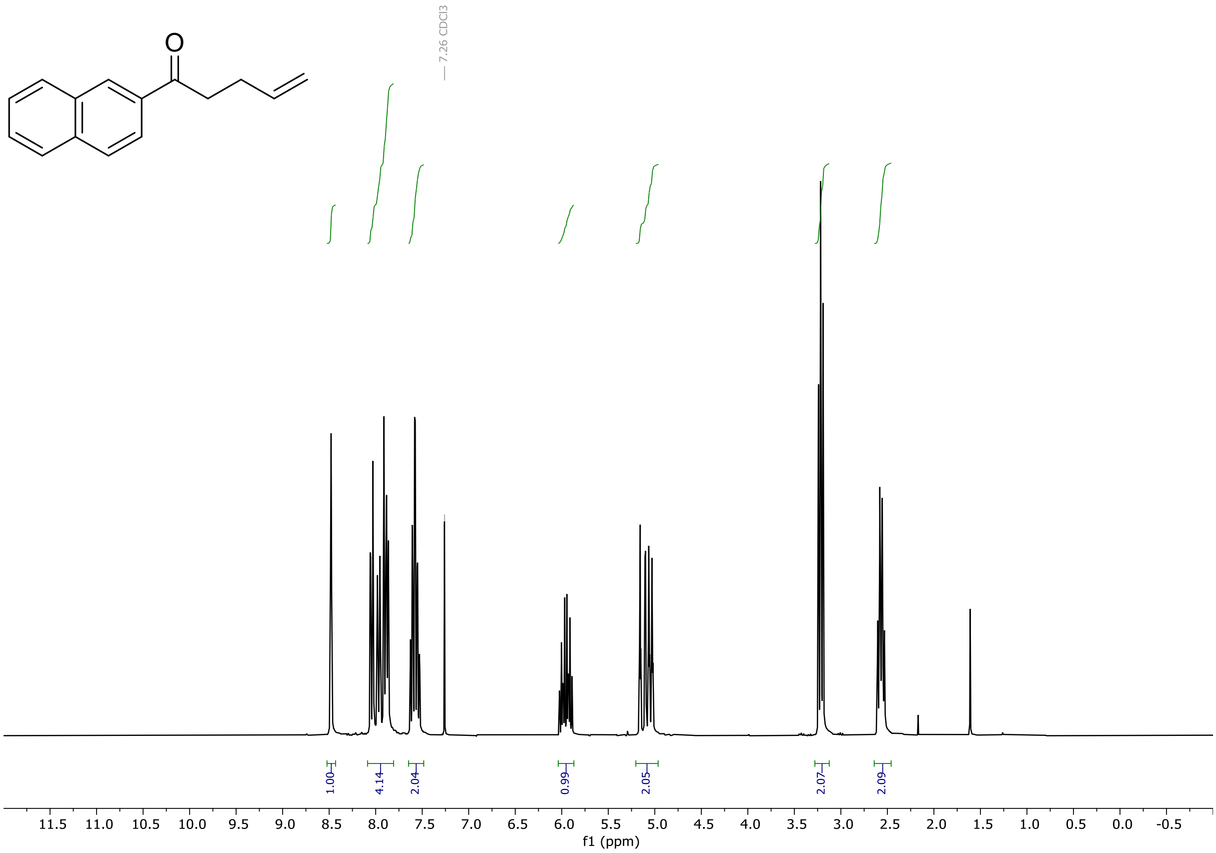

^{13}C-NMR (75 MHz, $CDCl_3$) of 1-(naphthalen-2-yl)pent-4-en-1-one (**3h**)

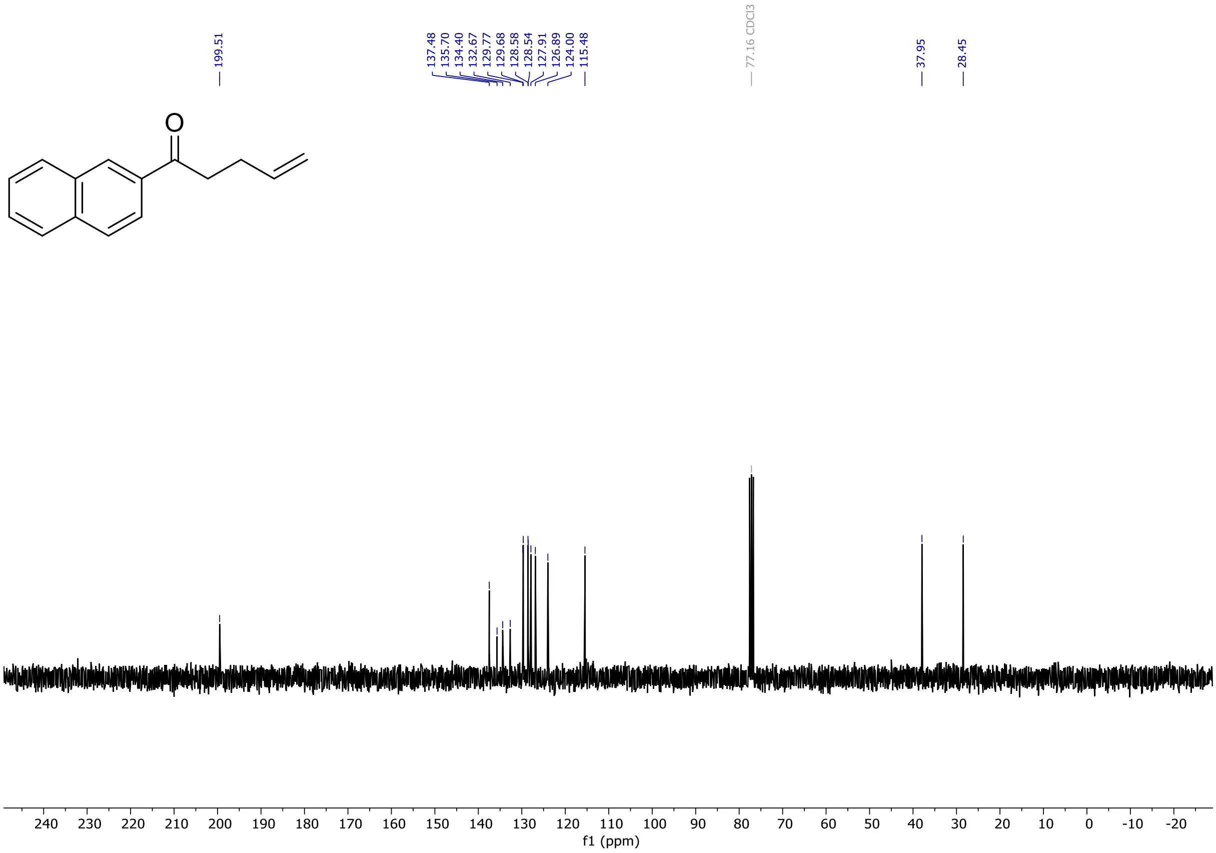

^{1}H-NMR (300 MHz, $CDCl_3$) of 4-(Pent-4-enoyl)benzonitrile (**3i**)

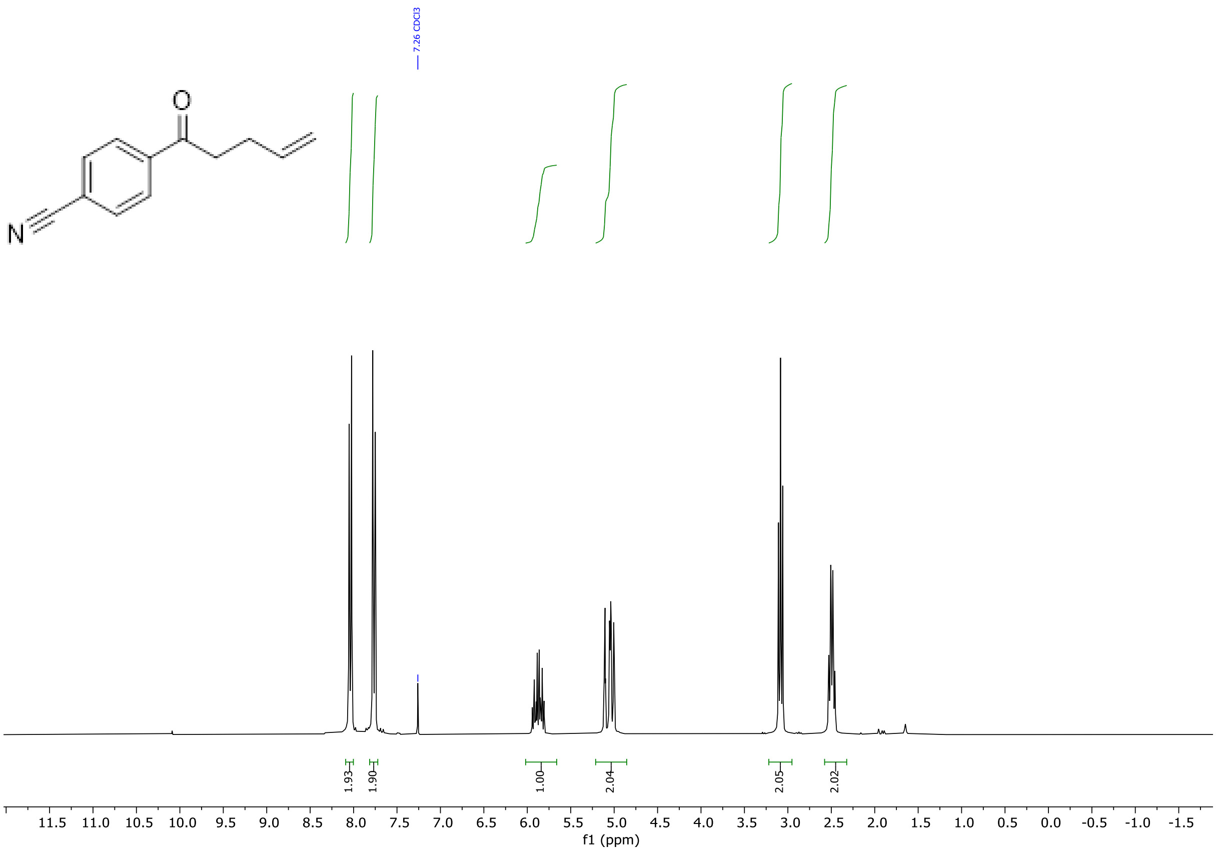

^{13}C-NMR (75 MHz, $CDCl_3$) of 4-(Pent-4-enoyl)benzonitrile (**3i**)

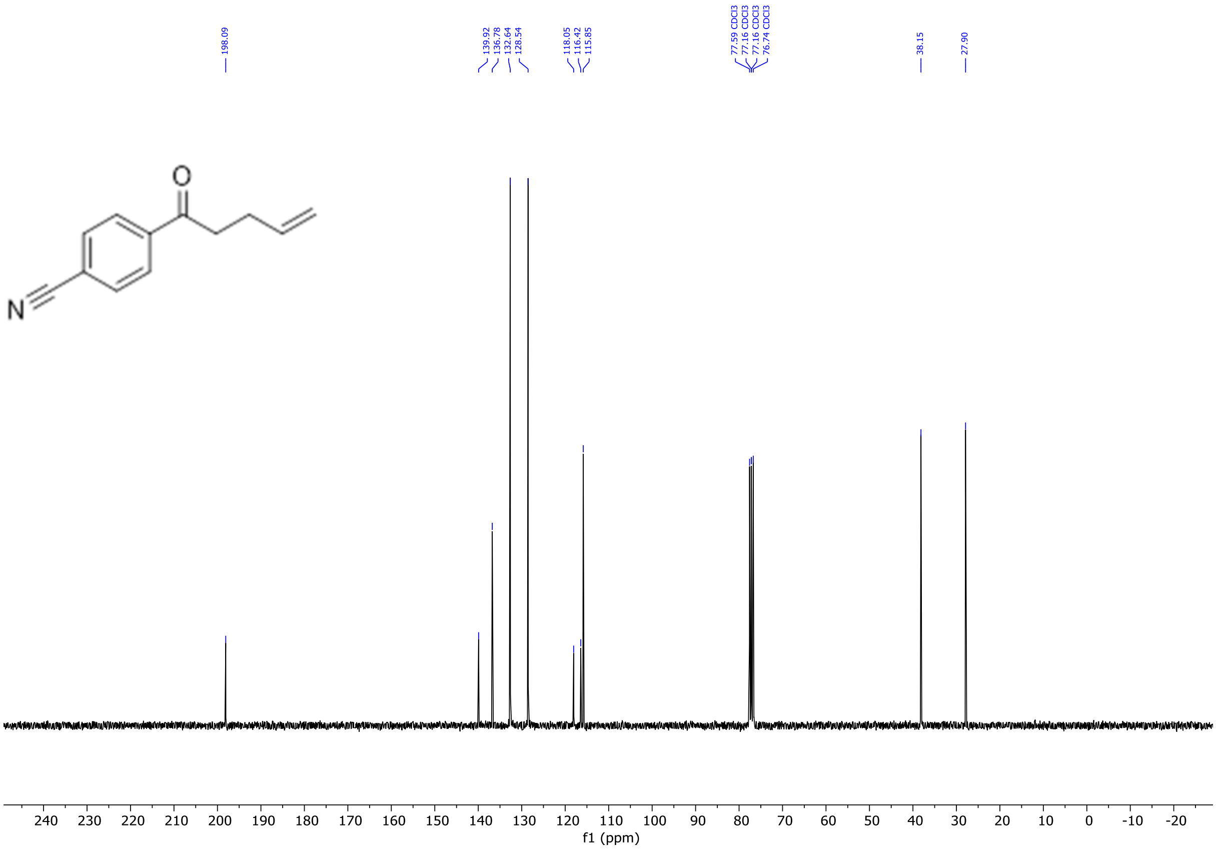

^{1}H-NMR (300 MHz, $CDCl_3$) of major diastereomer of Ethyl 3-phenylhepta-2,6-dienoate (**4a**)

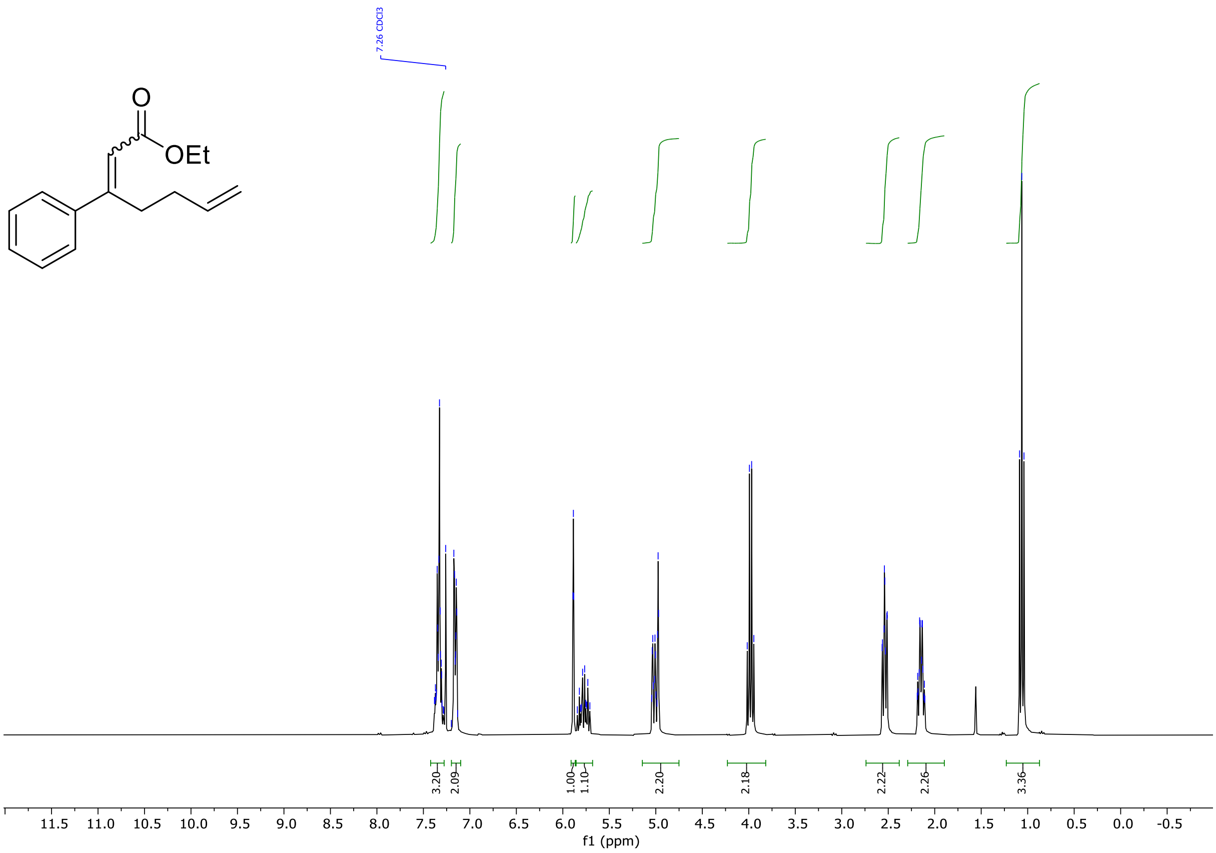

^{13}C-NMR (75 MHz, $CDCl_3$) of major diastereomer of Ethyl 3-phenylhepta-2,6-dienoate (**4a**)

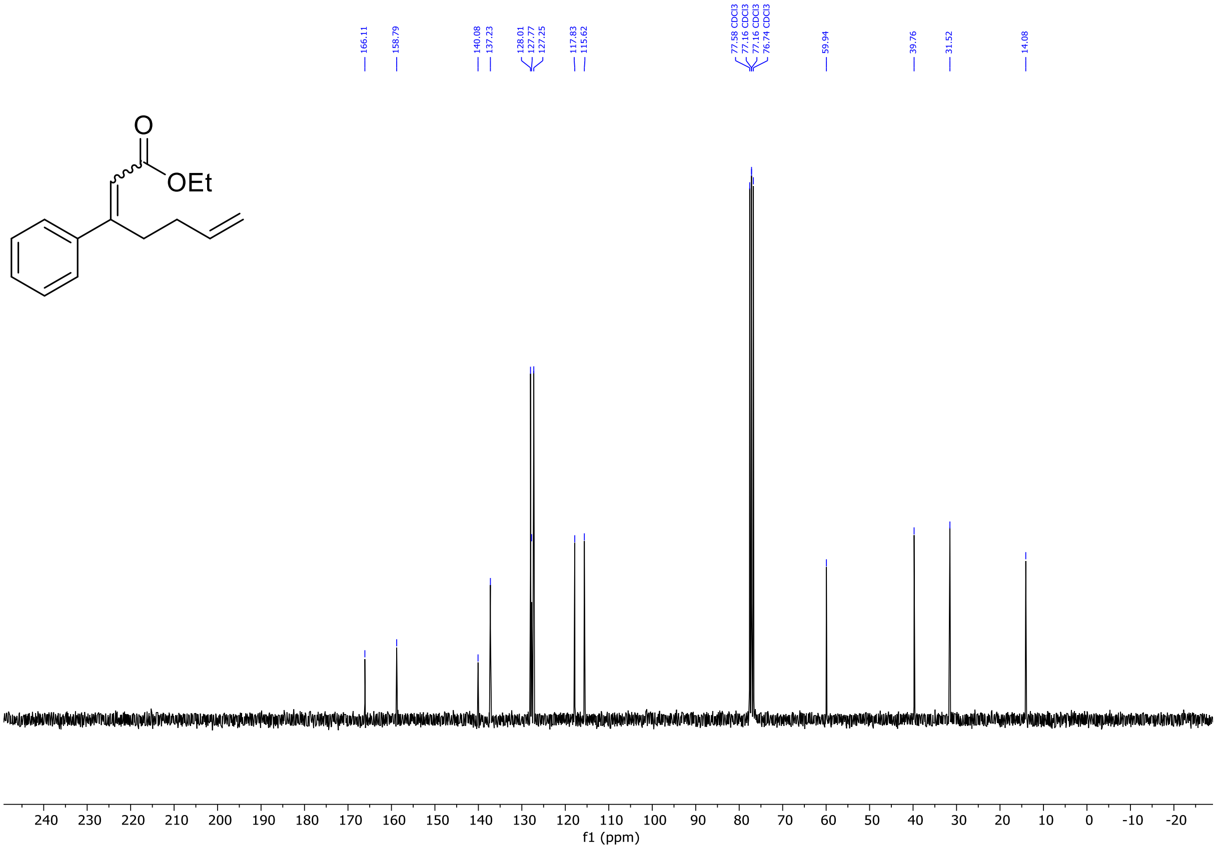

^{1}H-NMR (300 MHz, $CDCl_3$) of minor diastereomer of Ethyl 3-phenylhepta-2,6-dienoate (**4a**)

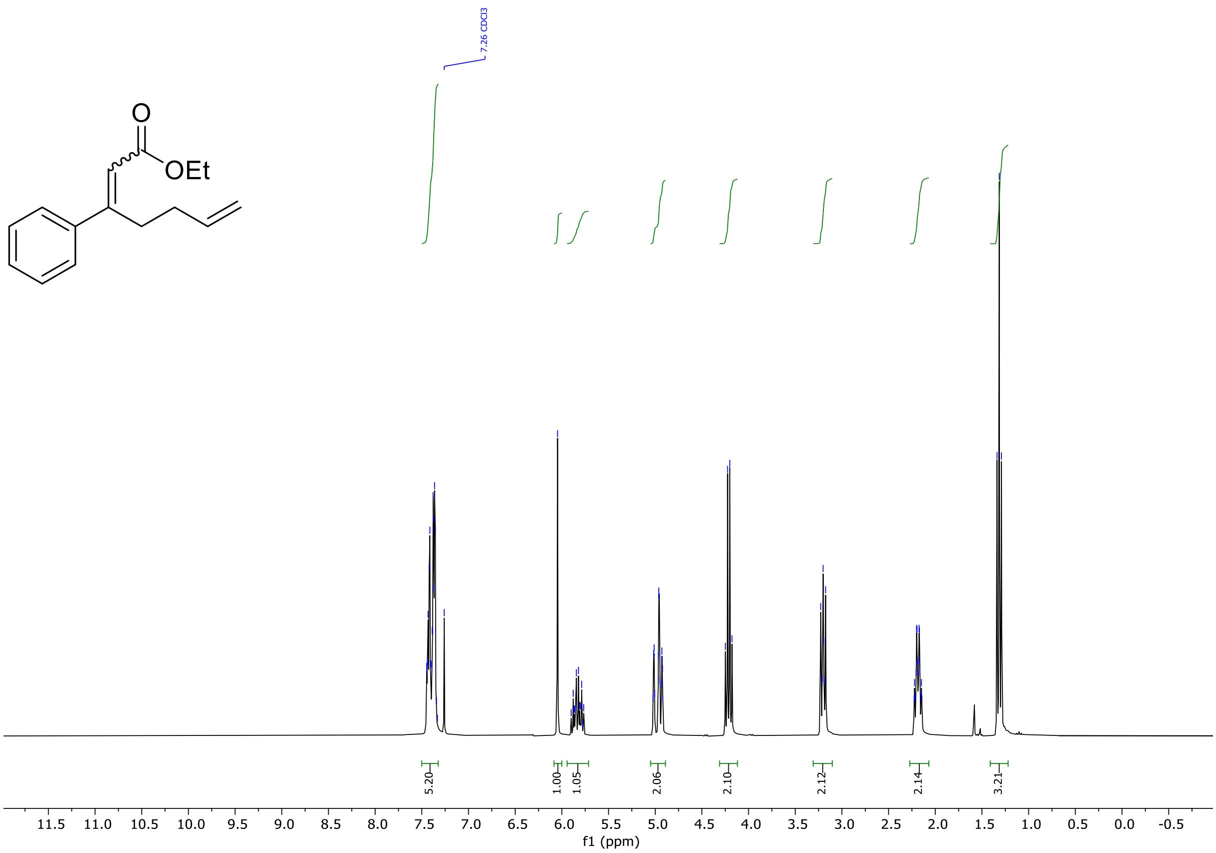

^{13}C-NMR (75 MHz, $CDCl_3$) of minor diastereomer of Ethyl 3-phenylhepta-2,6-dienoate (**4a**)

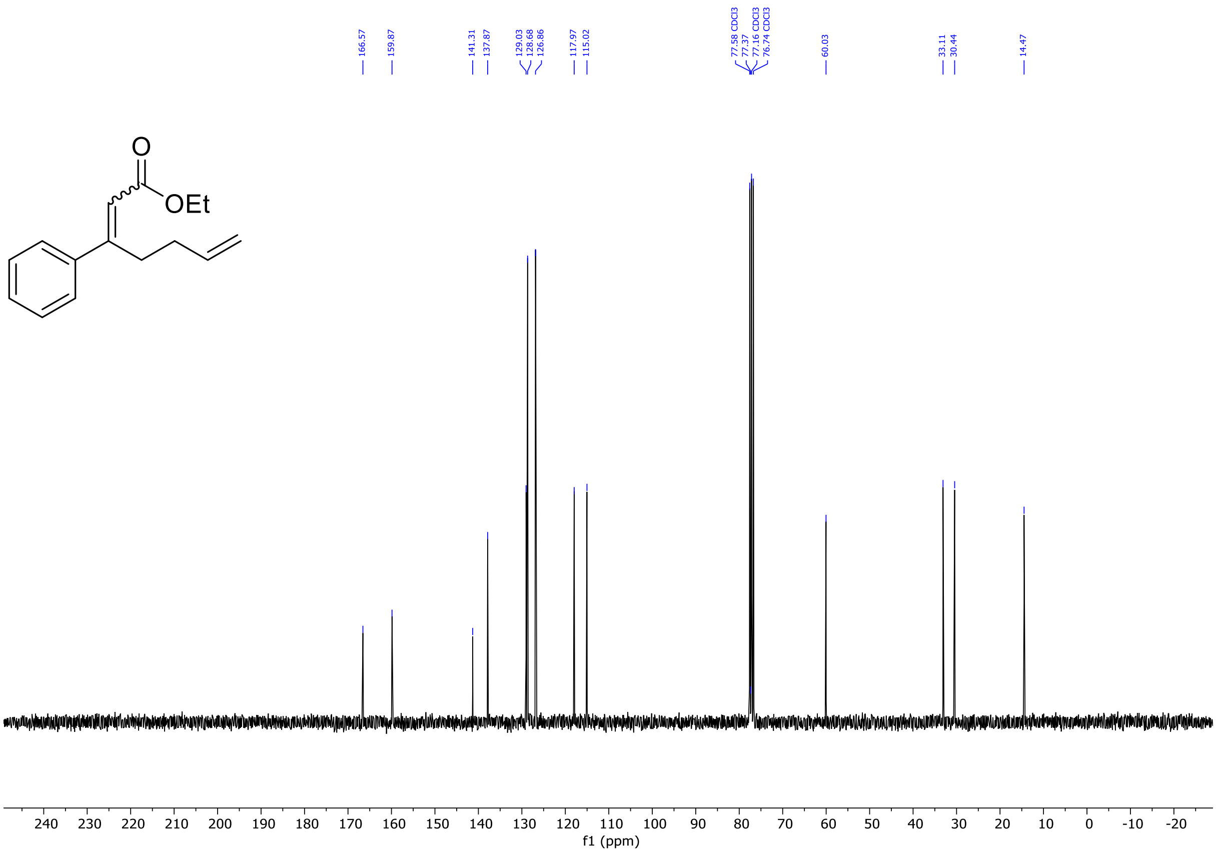

^{1}H-NMR (300 MHz, $CDCl_3$) of major diastereomer of Ethyl 3-(4-fluorophenyl)hepta-2,6-dienoate (**4b**)

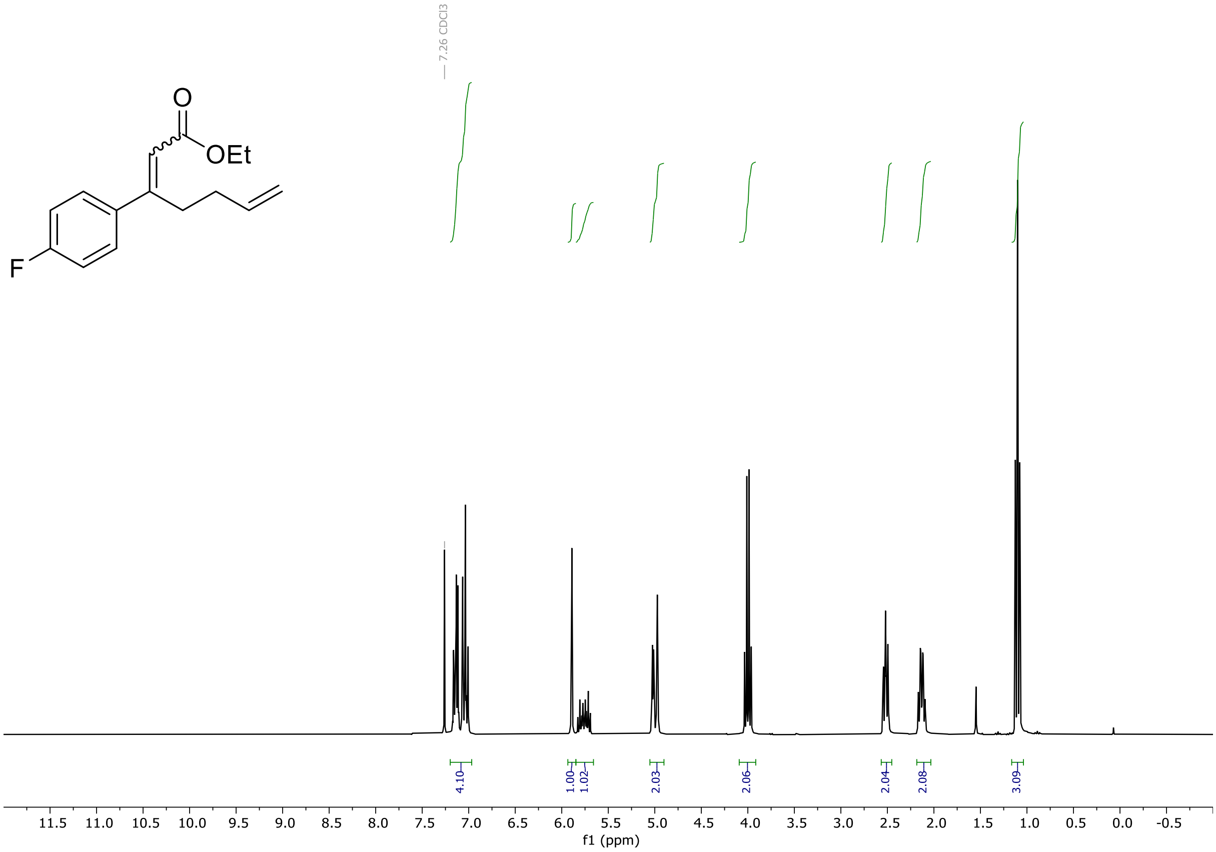

^{13}C-NMR (75 MHz, $CDCl_3$) of major diastereomer of Ethyl 3-(4-fluorophenyl)hepta-2,6-dienoate (**4b**)

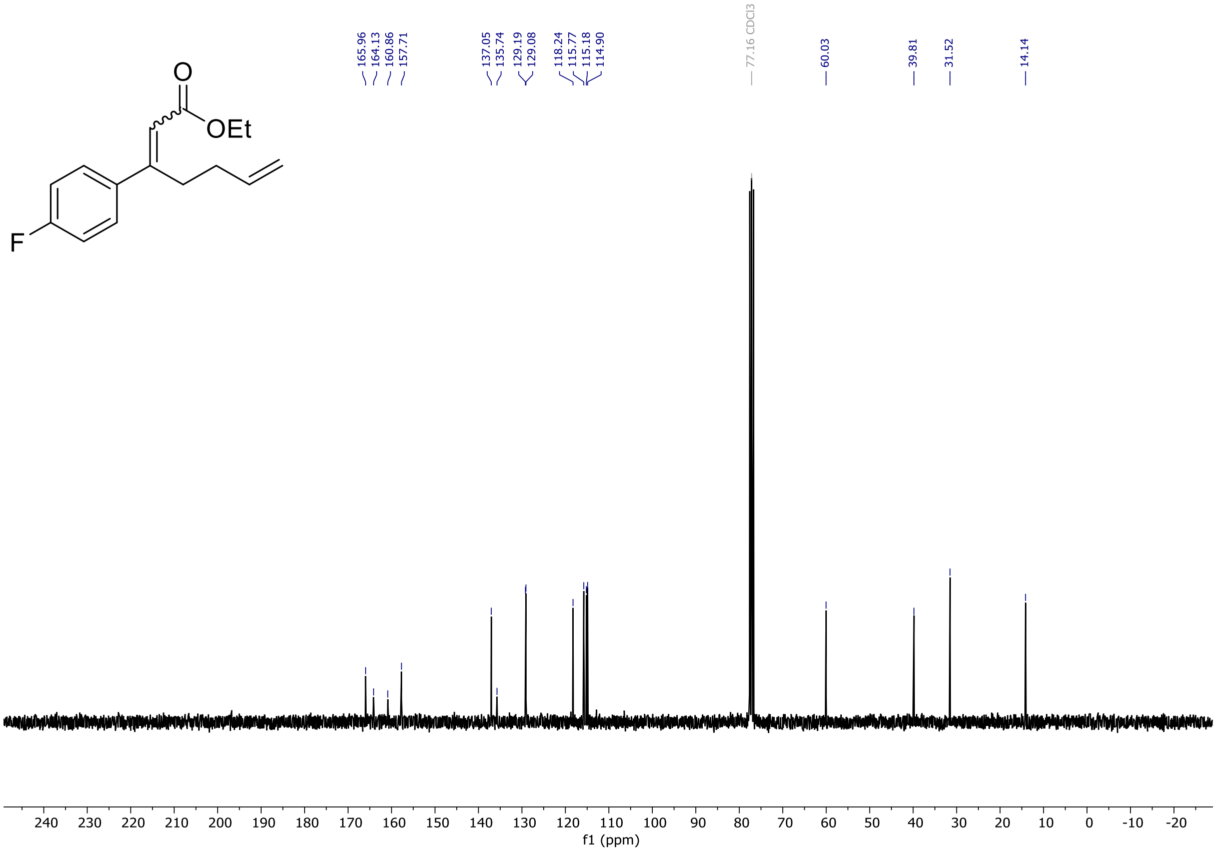

^{19}F-NMR (282 MHz, $CDCl_3$) of major diastereomer of Ethyl 3-(4-fluorophenyl)hepta-2,6-dienoate (**4b**)

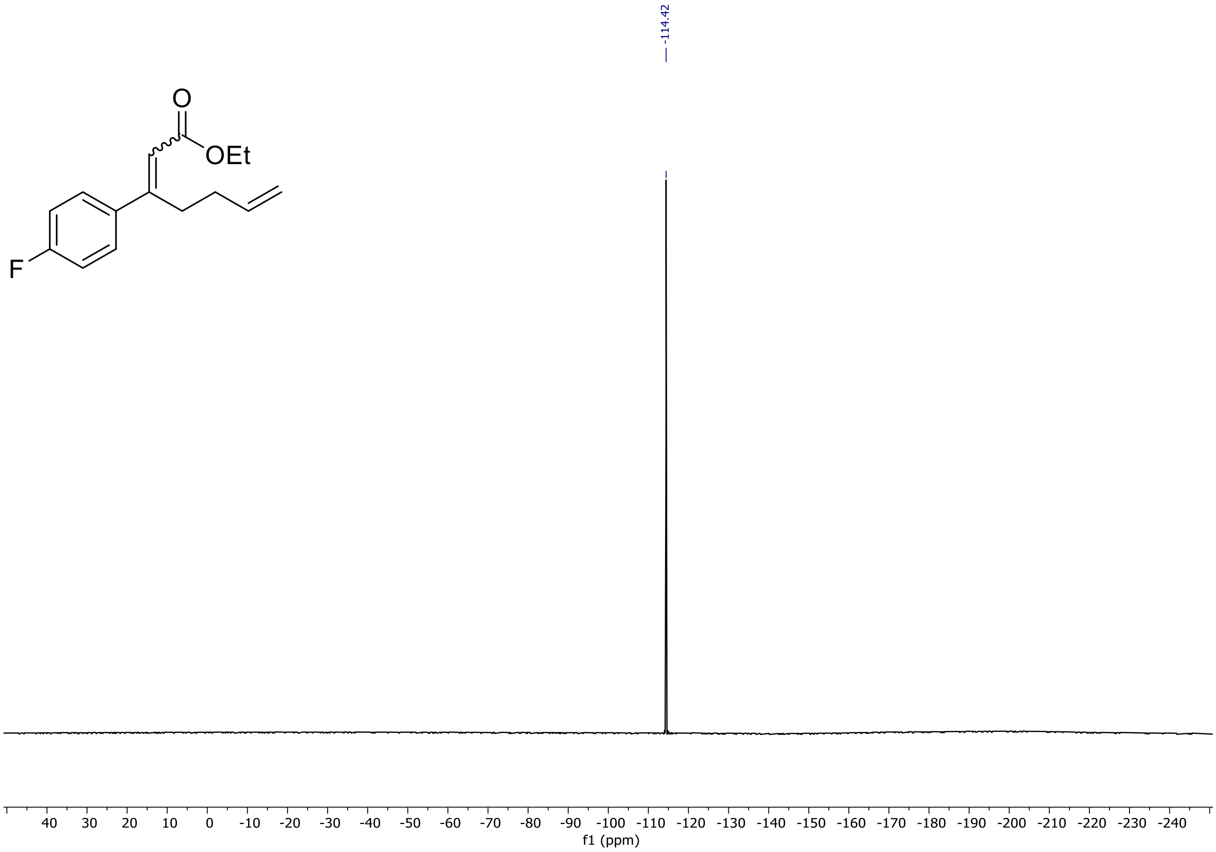

^{1}H-NMR (300 MHz, $CDCl_3$) of minor diastereomer of Ethyl 3-(4-fluorophenyl)hepta-2,6-dienoate (**4b**)

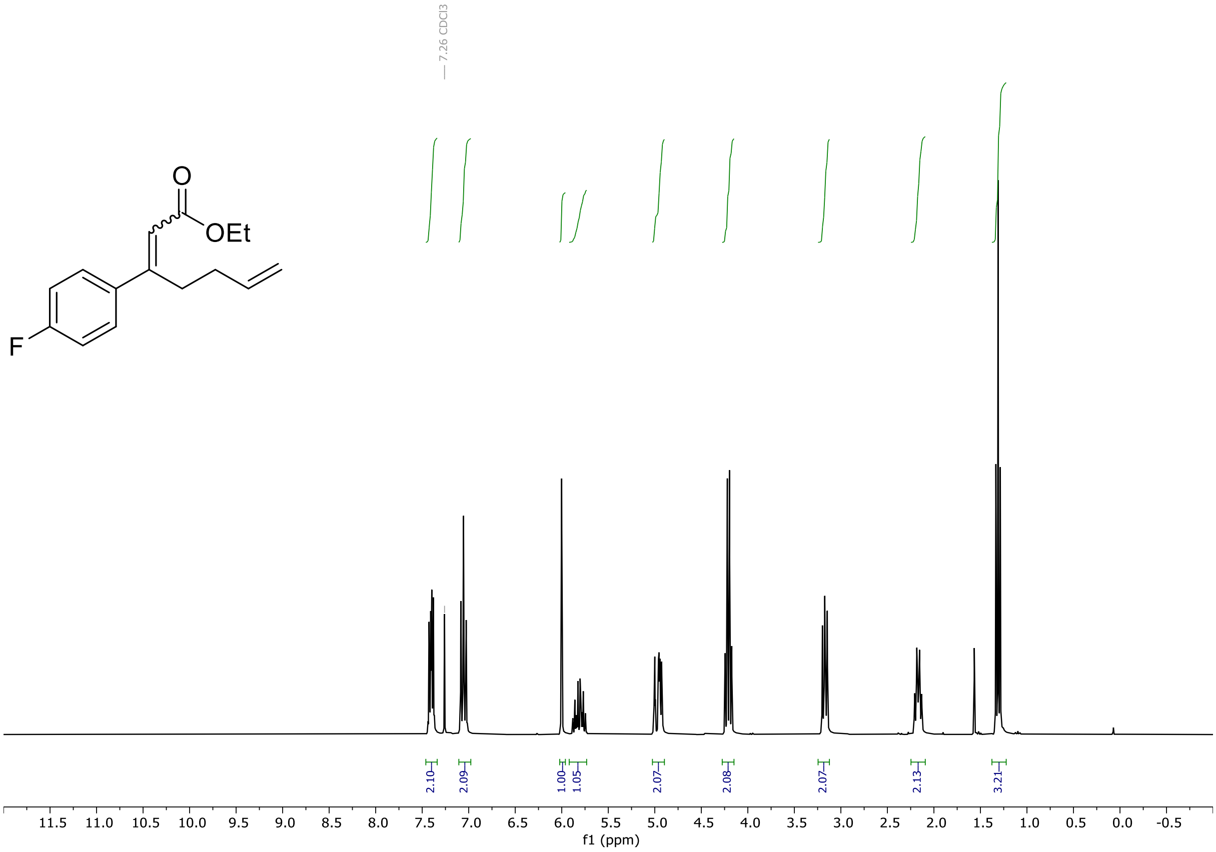

^{13}C-NMR (75 MHz, $CDCl_3$) of minor diastereomer of Ethyl 3-(4-fluorophenyl)hepta-2,6-dienoate (**4b**)

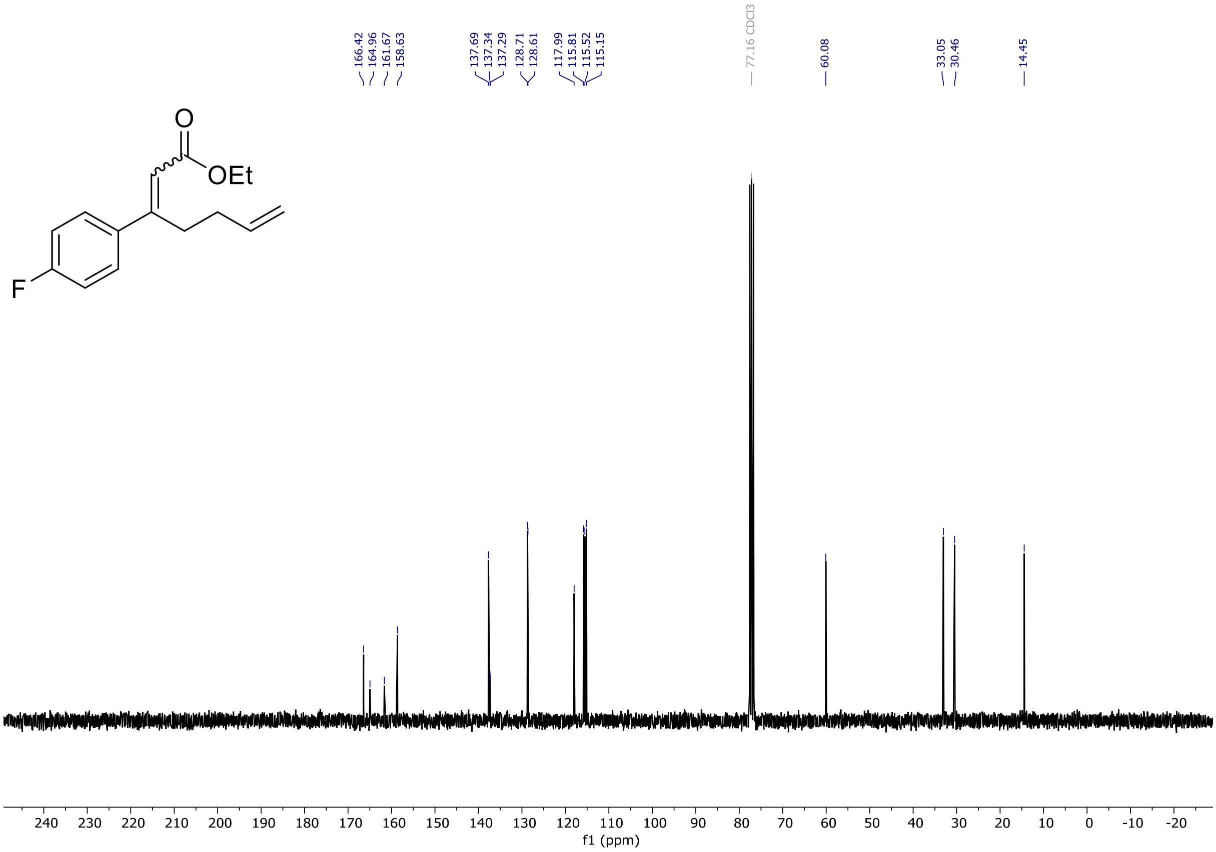

^{19}F-NMR (282 MHz, $CDCl_3$) of minor diastereomer of Ethyl 3-(4-fluorophenyl)hepta-2,6-dienoate (**4b**)

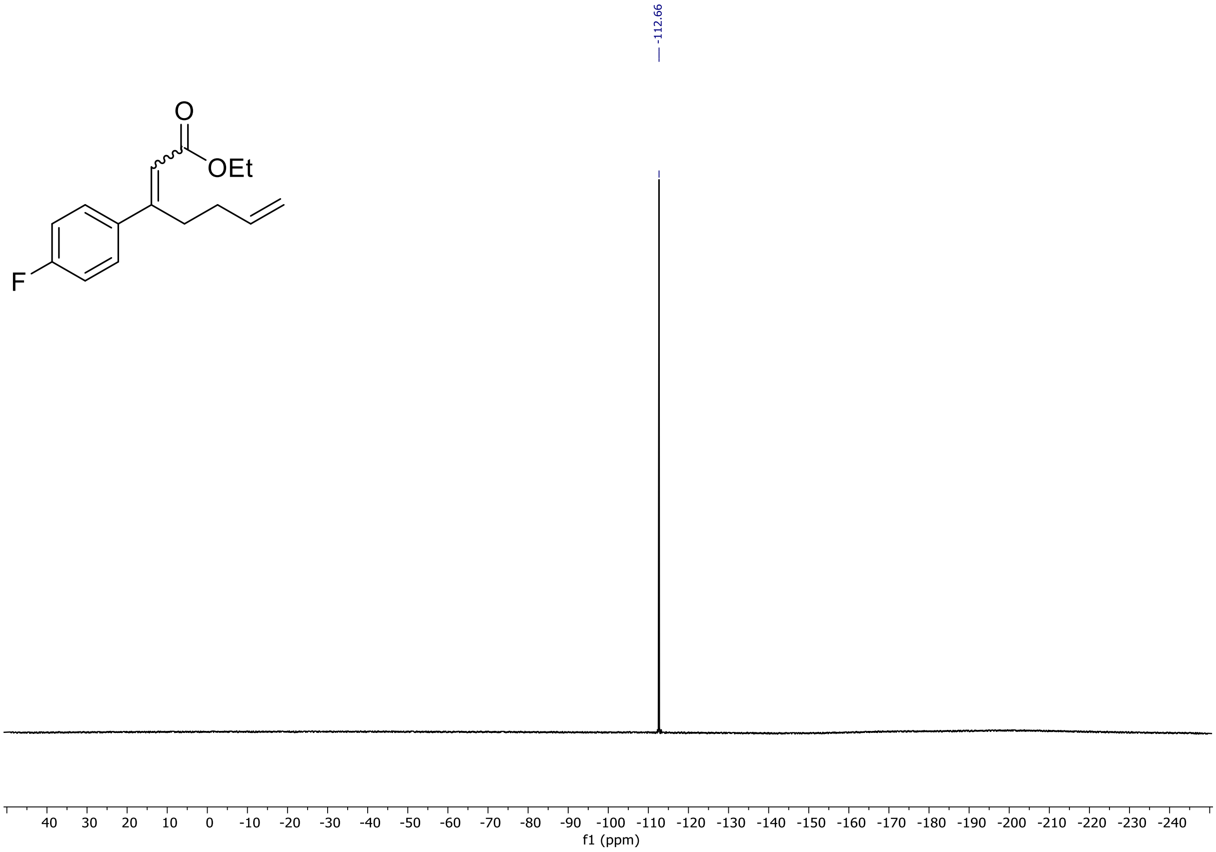

^{1}H-NMR (300 MHz, $CDCl_3$) of Ethyl 3-(4-chlorophenyl)hepta-2,6-dienoate (**4c**)

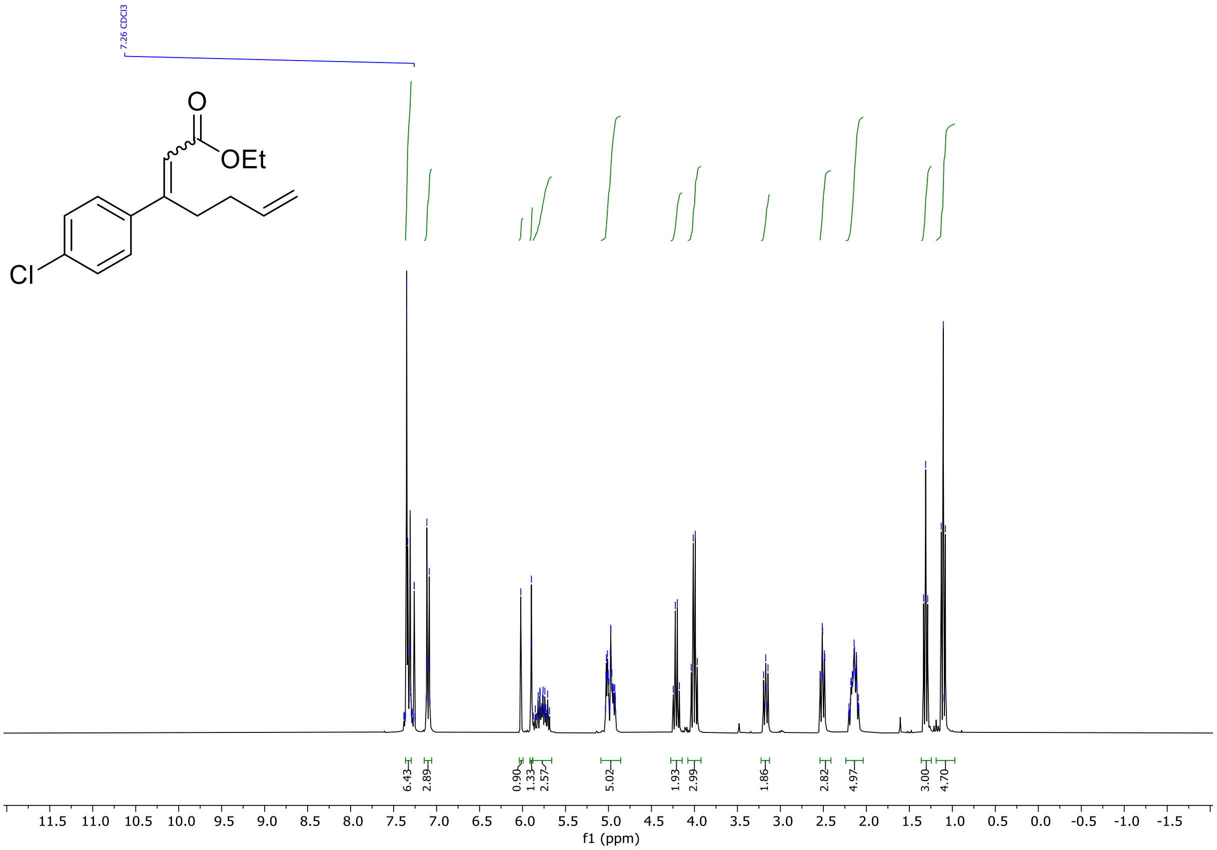

^{13}C-NMR (75 MHz, $CDCl_3$) of Ethyl 3-(4-chlorophenyl)hepta-2,6-dienoate (**4c**)

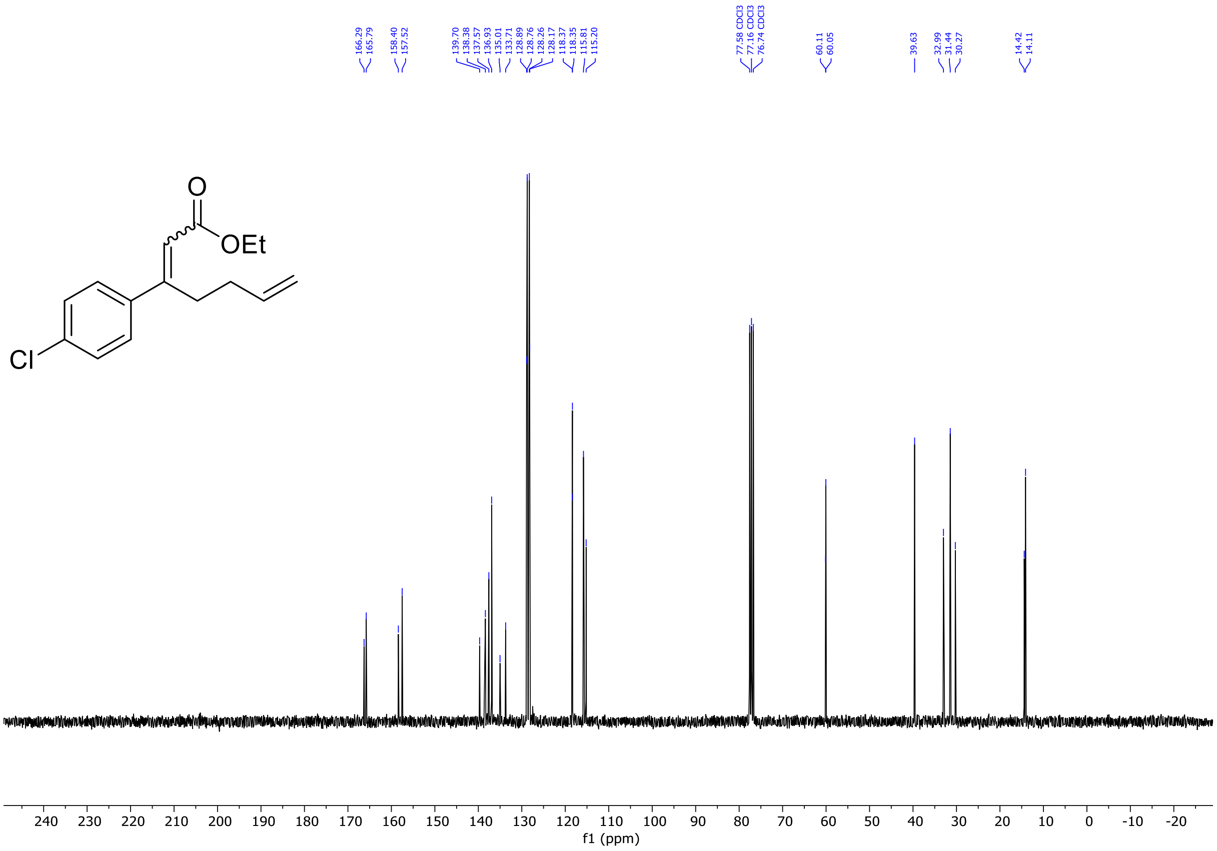

^{1}H-NMR (300 MHz, $CDCl_3$) of Ethyl 3-(4-bromophenyl)hepta-2,6-dienoate (**4d**)

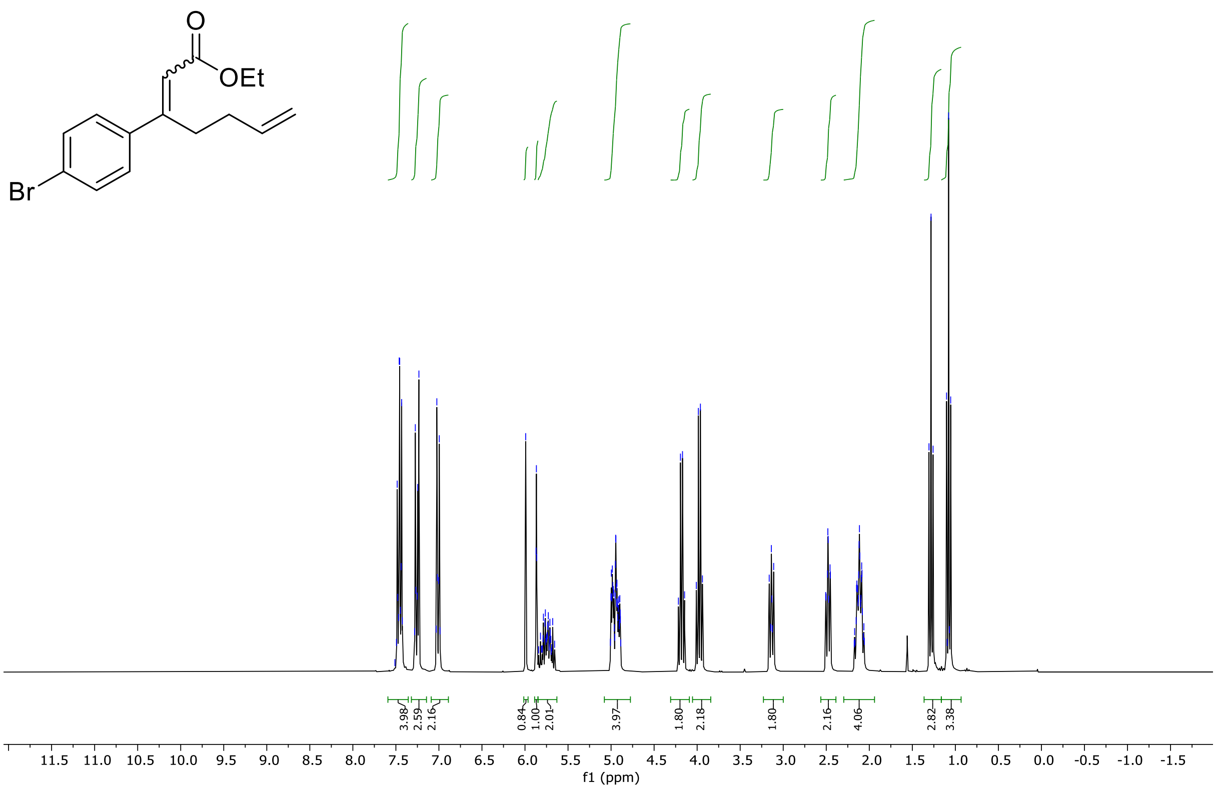

^{13}C-NMR (75 MHz, $CDCl_3$) of Ethyl 3-(4-bromophenyl)hepta-2,6-dienoate (**4d**)

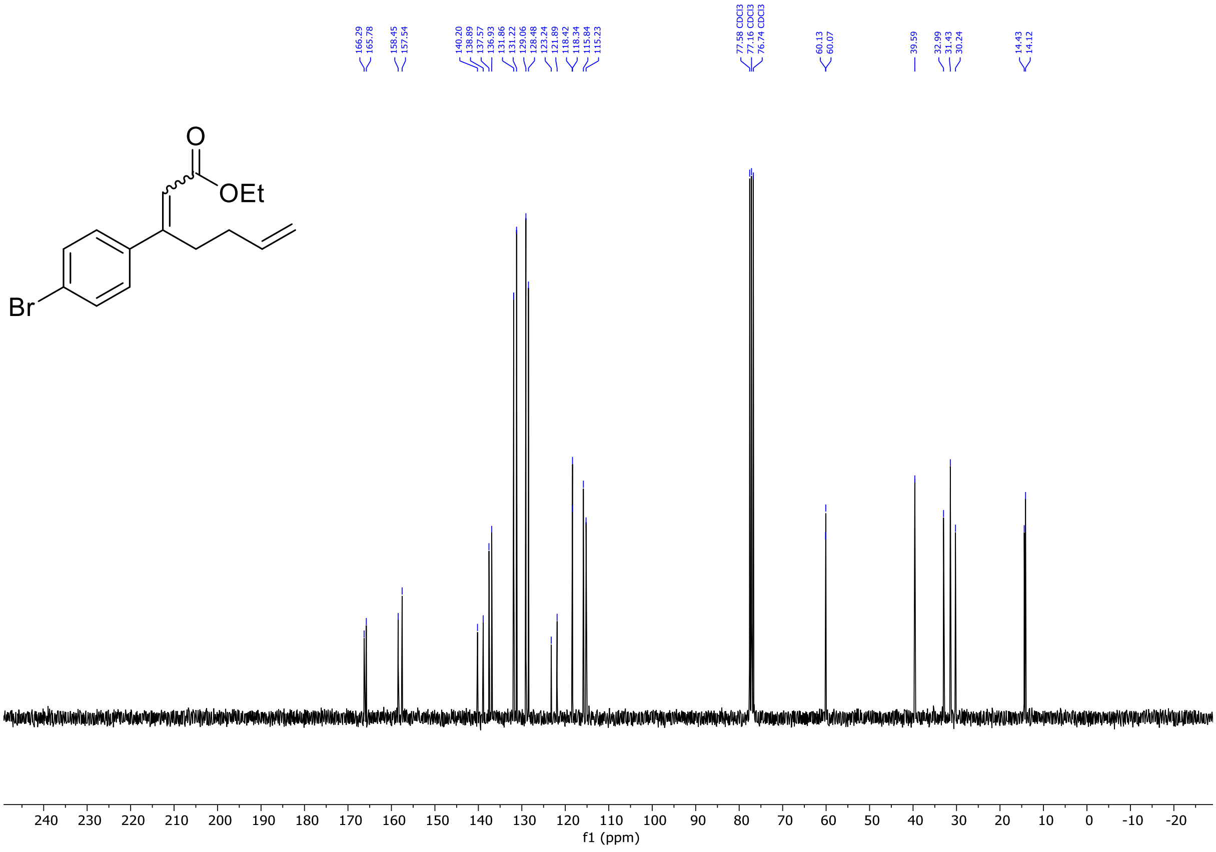

^{1}H-NMR (300 MHz, $CDCl_3$) of Ethyl 3-(4-(trifluoromethyl)phenyl)hepta-2,6-dienoate (**4e**)

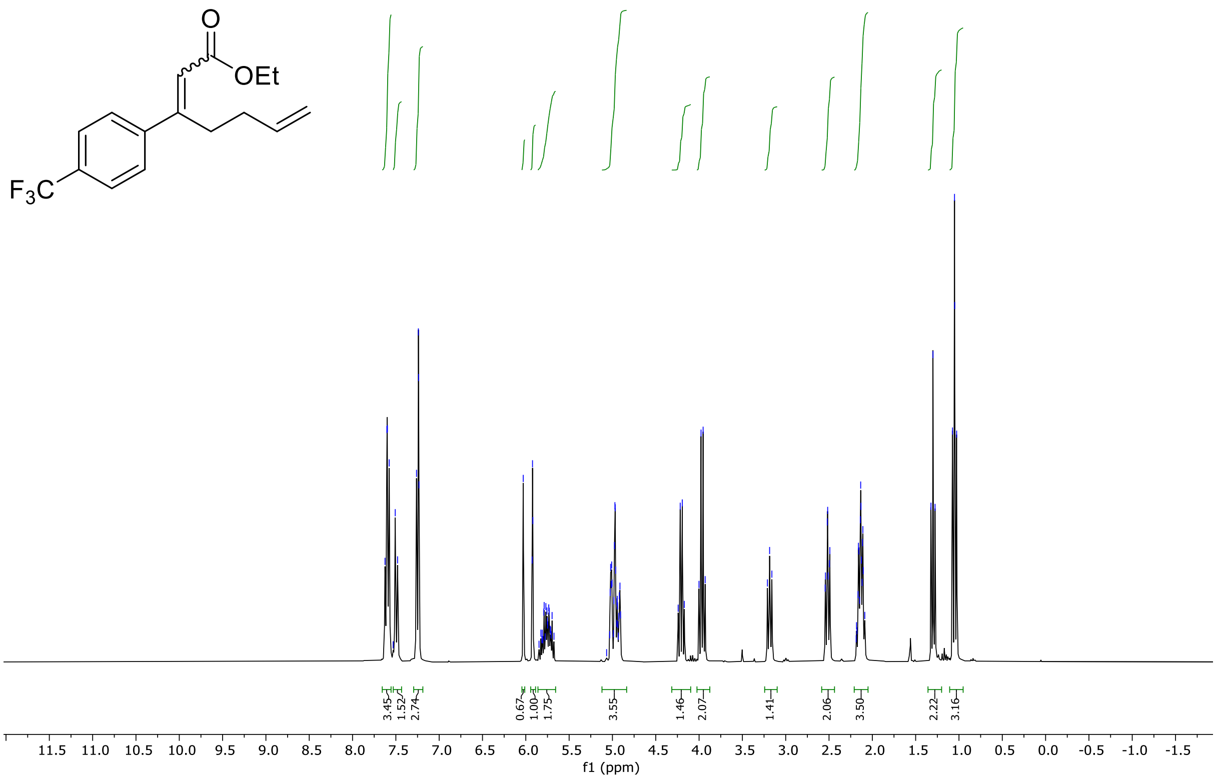

^{13}C-NMR (75 MHz, $CDCl_3$) of Ethyl 3-(4-(trifluoromethyl)phenyl)hepta-2,6-dienoate (**4e**)

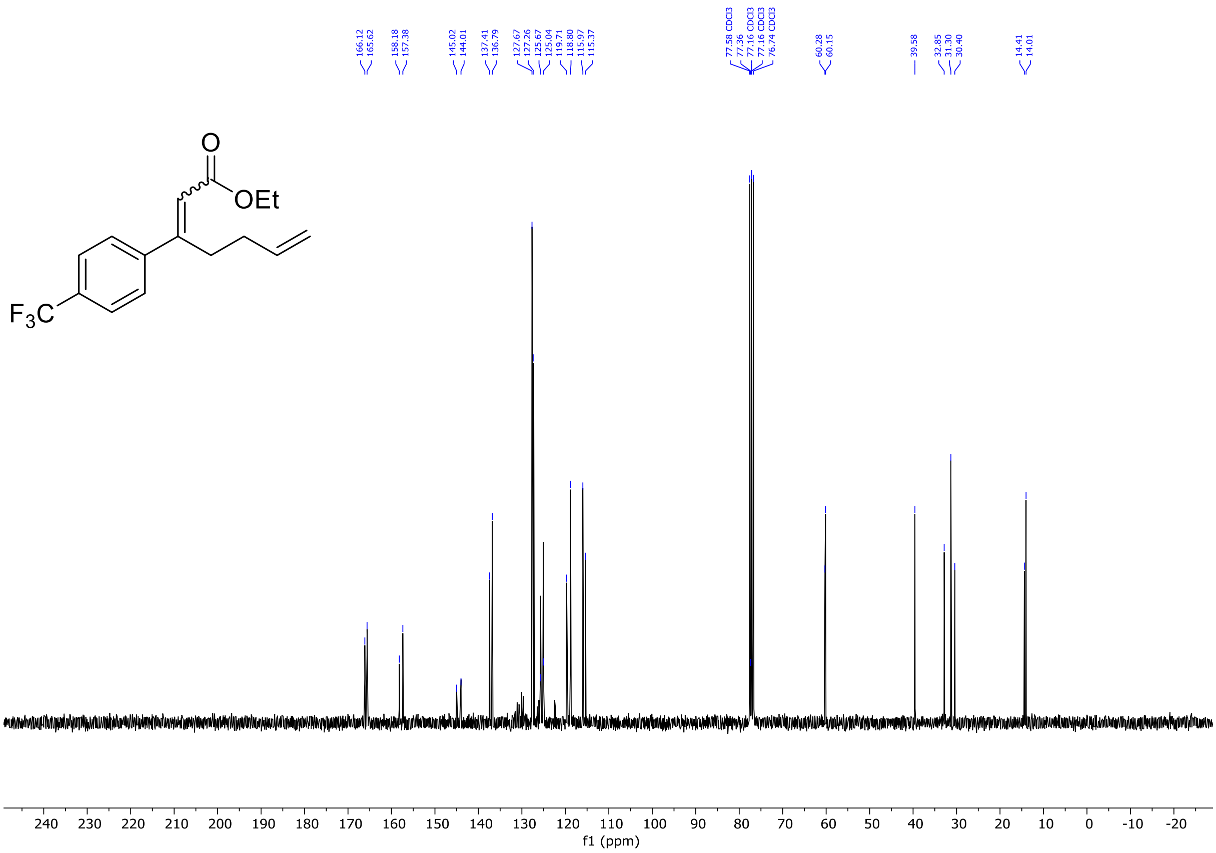

^{19}F-NMR (282 MHz, $CDCl_3$) of Ethyl 3-(4-(trifluoromethyl)phenyl)hepta-2,6-dienoate (**4e**)

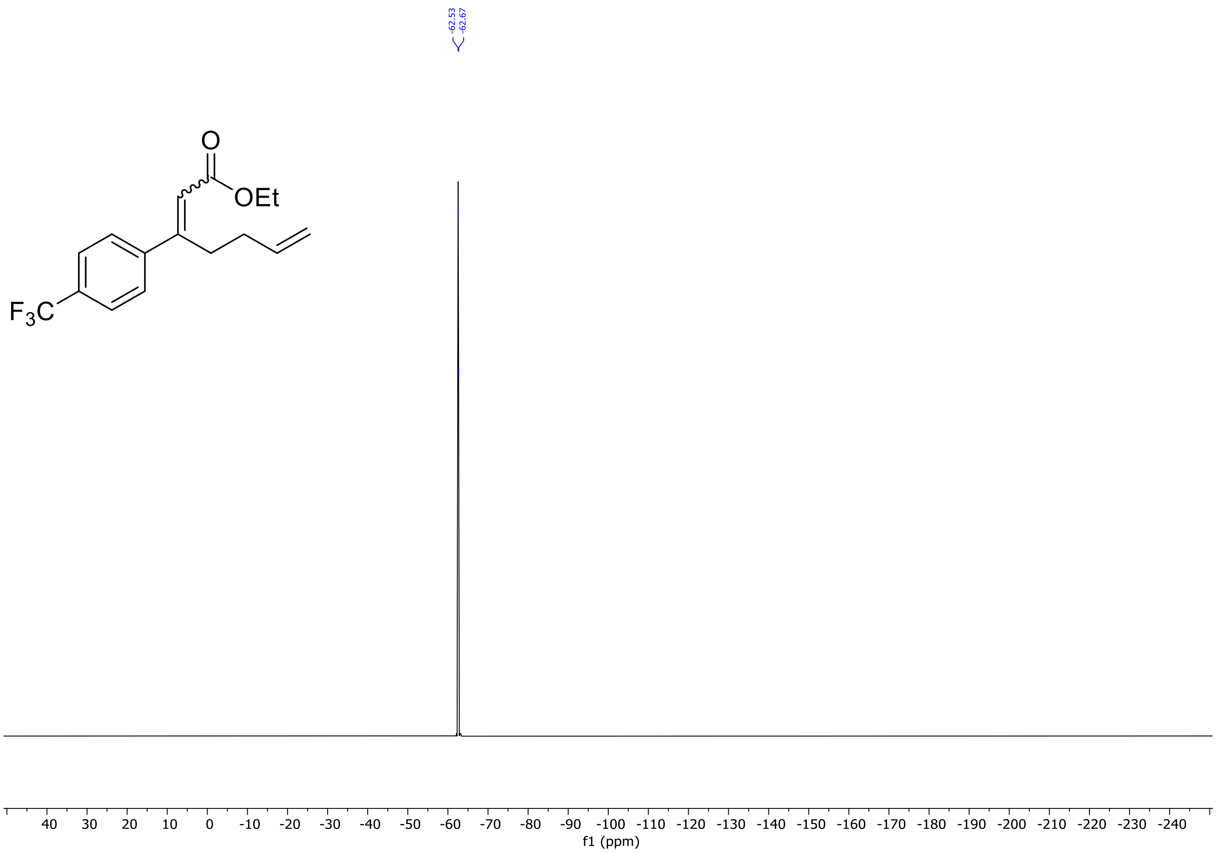

^{1}H-NMR (300 MHz, $CDCl_3$) of major diastereomer of Ethyl 3-(*p*-tolyl)hepta-2,6-dienoate (**4f**)

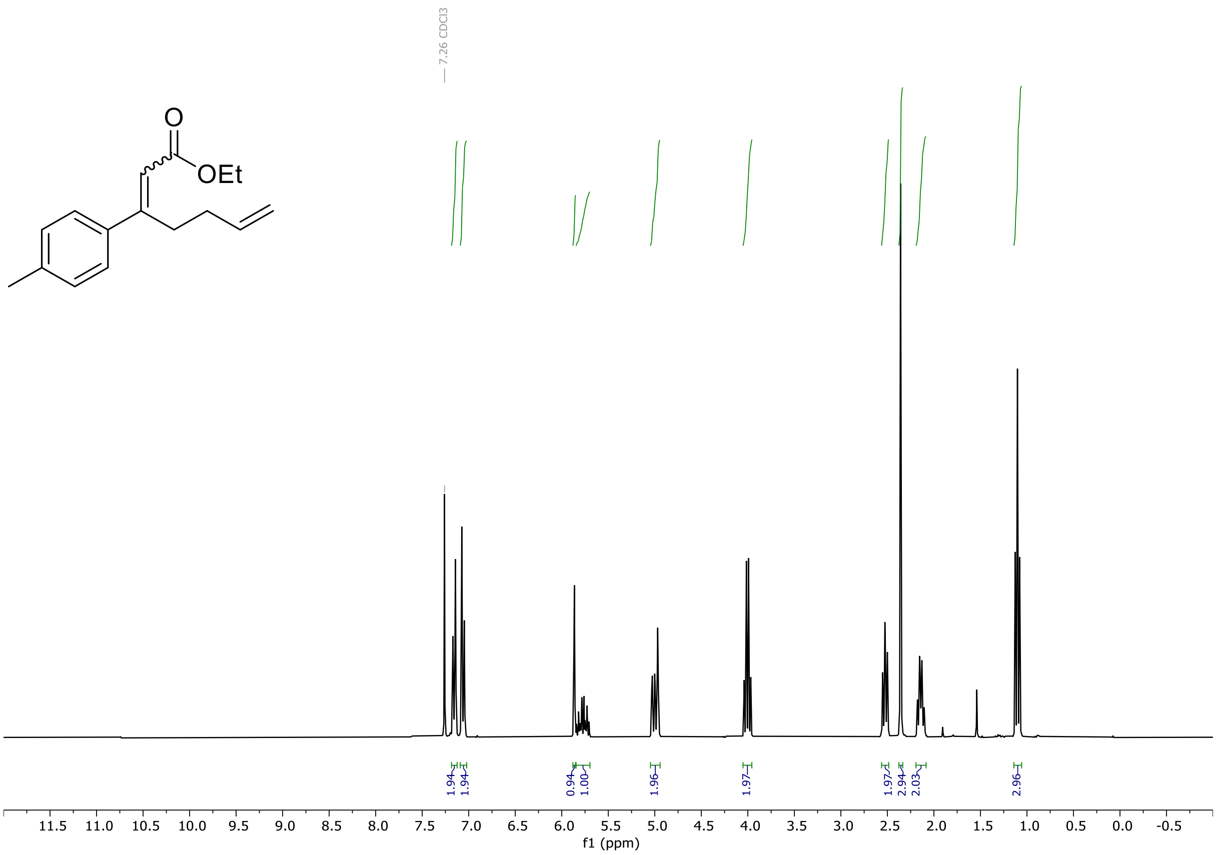

^{13}C-NMR (75 MHz, $CDCl_3$) of major diastereomer of Ethyl 3-(*p*-tolyl)hepta-2,6-dienoate (**4f**)

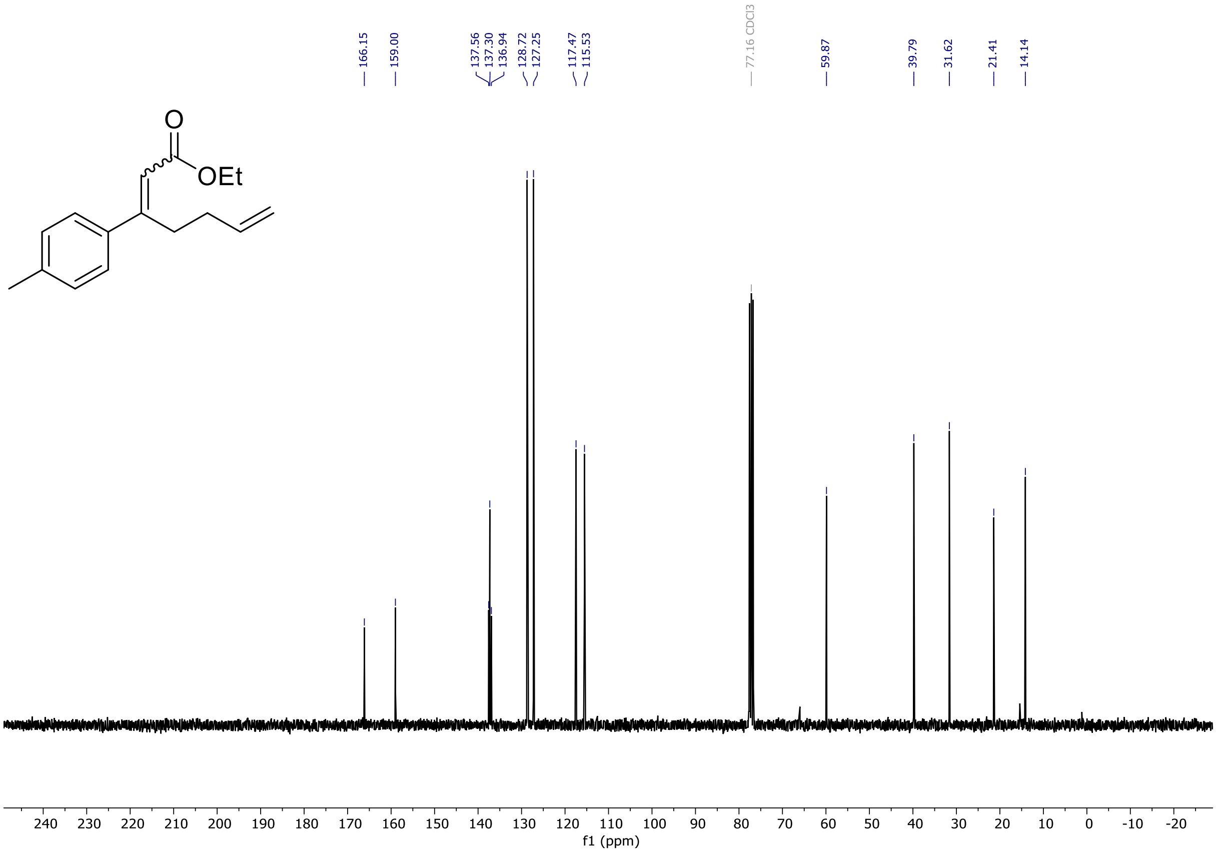

^{1}H-NMR (300 MHz, $CDCl_3$) of minor diastereomer of Ethyl 3-(*p*-tolyl)hepta-2,6-dienoate (**4f**)

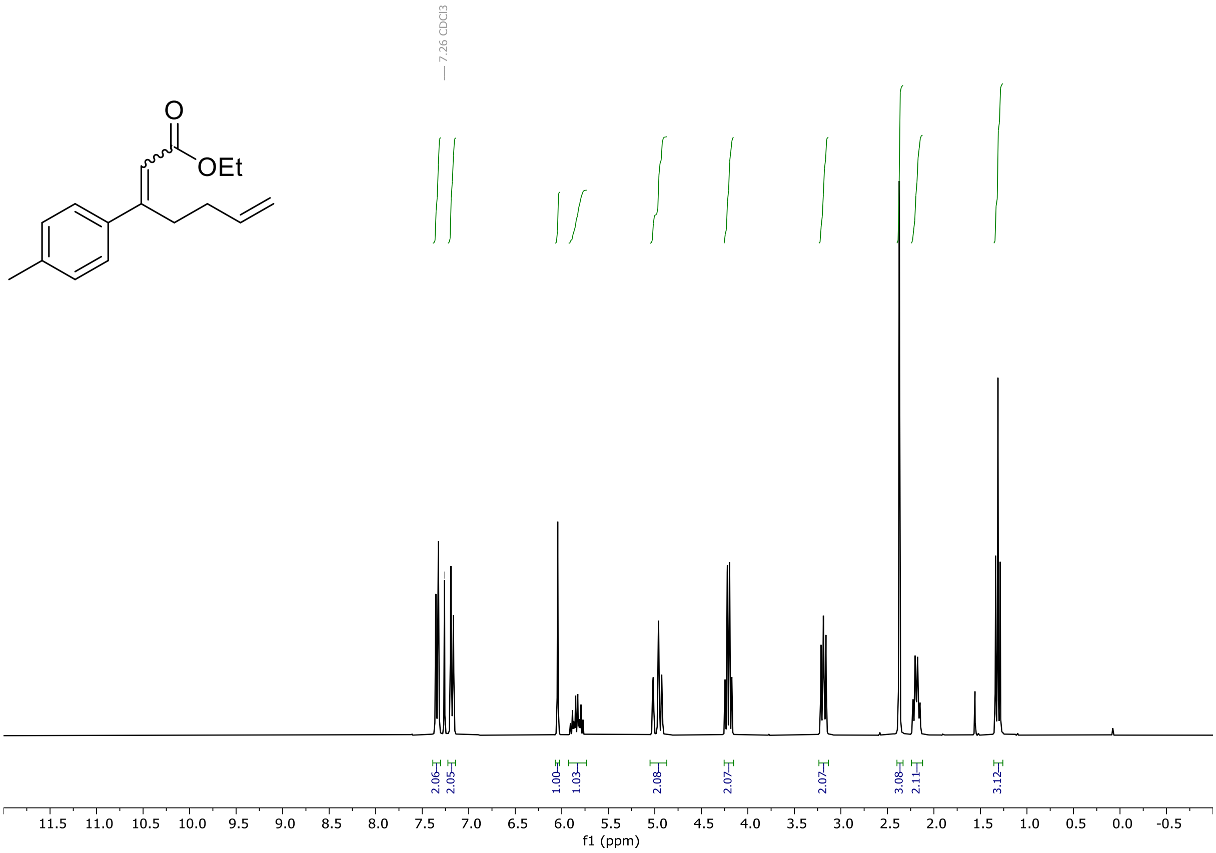

^{13}C-NMR (75 MHz, $CDCl_3$) of minor diastereomer of Ethyl 3-(*p*-tolyl)hepta-2,6-dienoate (**4f**)

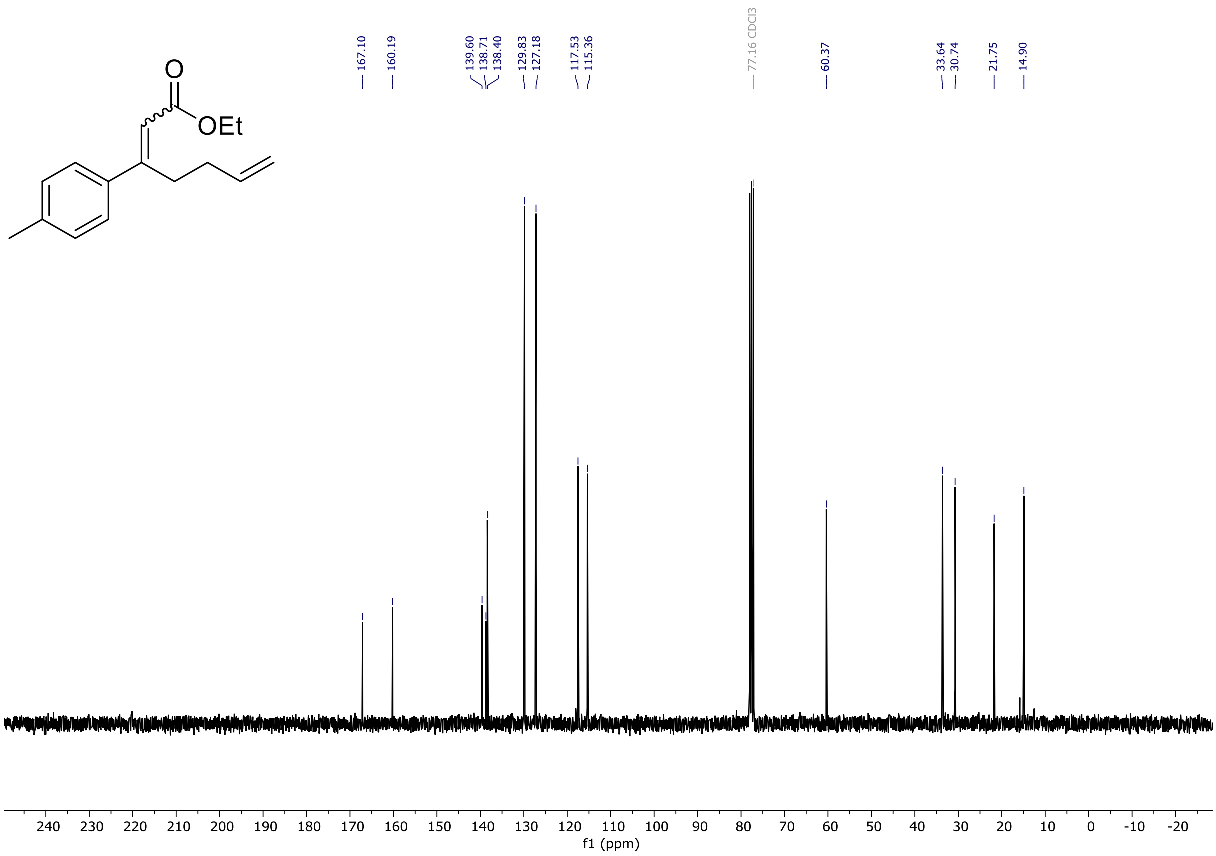

^{1}H-NMR (300 MHz, $CDCl_3$) of major diastereomer of Ethyl 3-(*m*-tolyl)hepta-2,6-dienoate (**4g**)

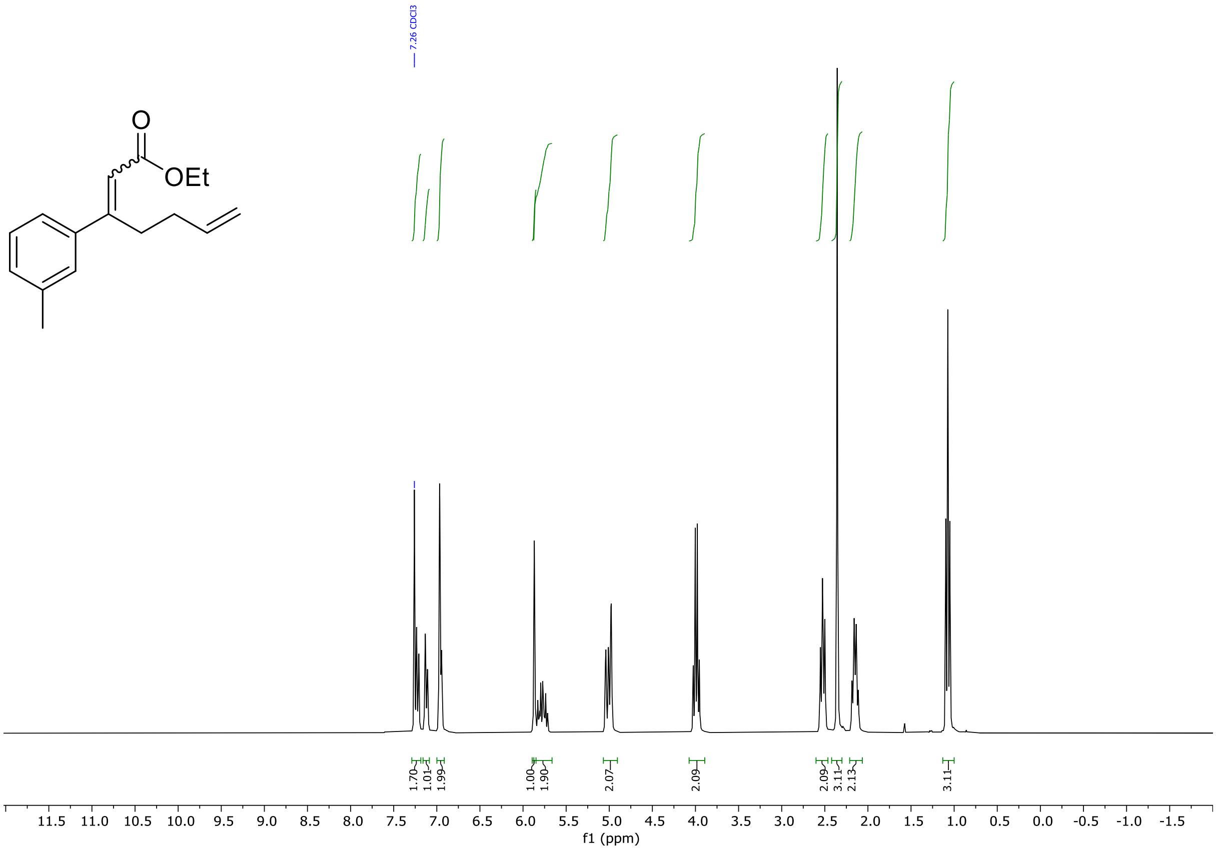

^{13}C-NMR (75 MHz, $CDCl_3$) of major diastereomer of Ethyl 3-(*m*-tolyl)hepta-2,6-dienoate (**4g**)

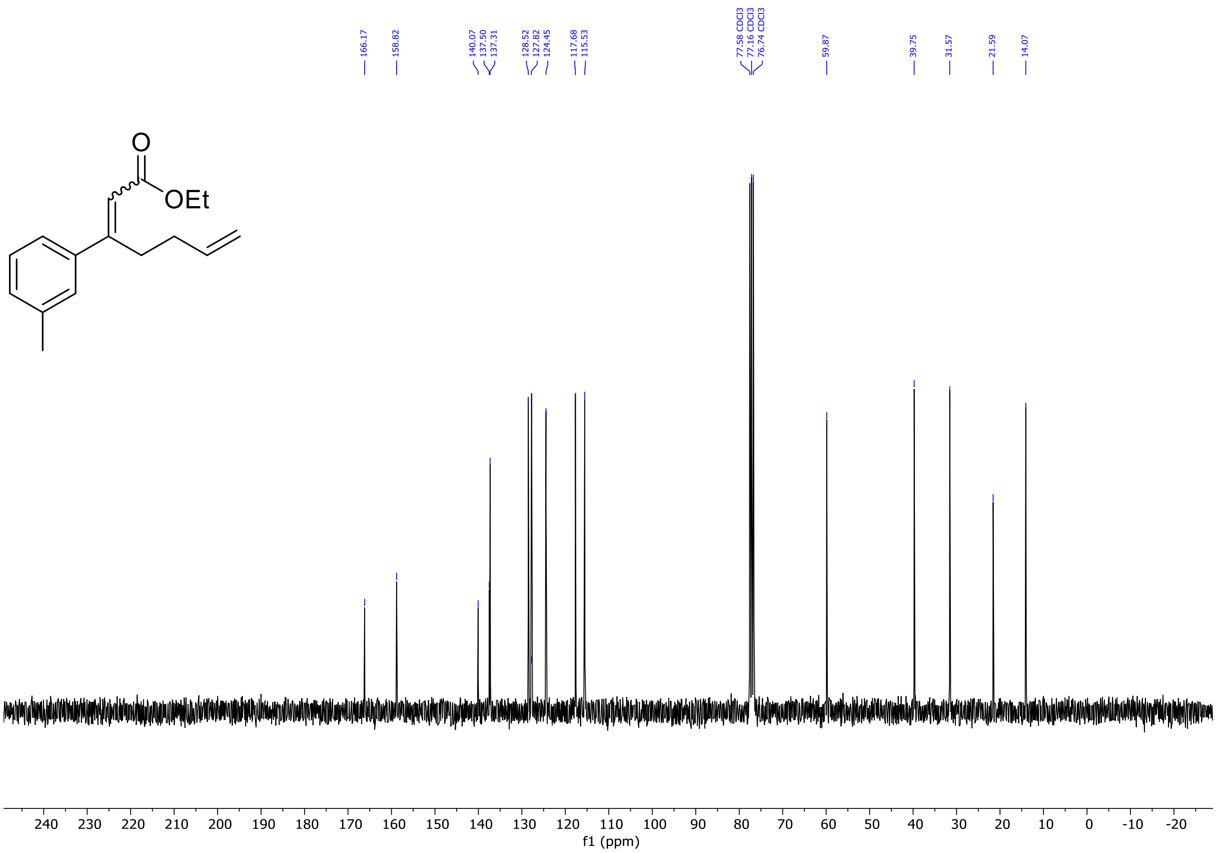

^{1}H-NMR (300 MHz, $CDCl_3$) of minor diastereomer of Ethyl 3-(*m*-tolyl)hepta-2,6-dienoate (**4g**)

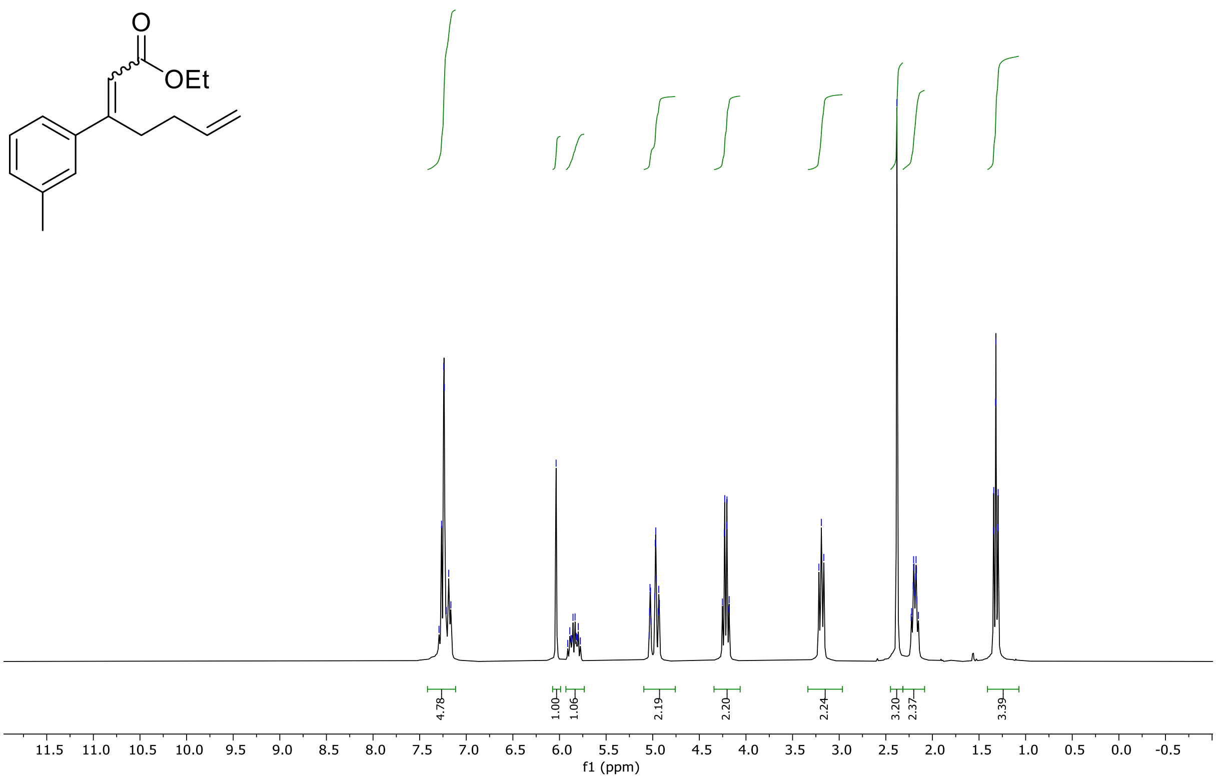

^{13}C-NMR (75 MHz, $CDCl_3$) of minor diastereomer of Ethyl 3-(*m*-tolyl)hepta-2,6-dienoate (**4g**)

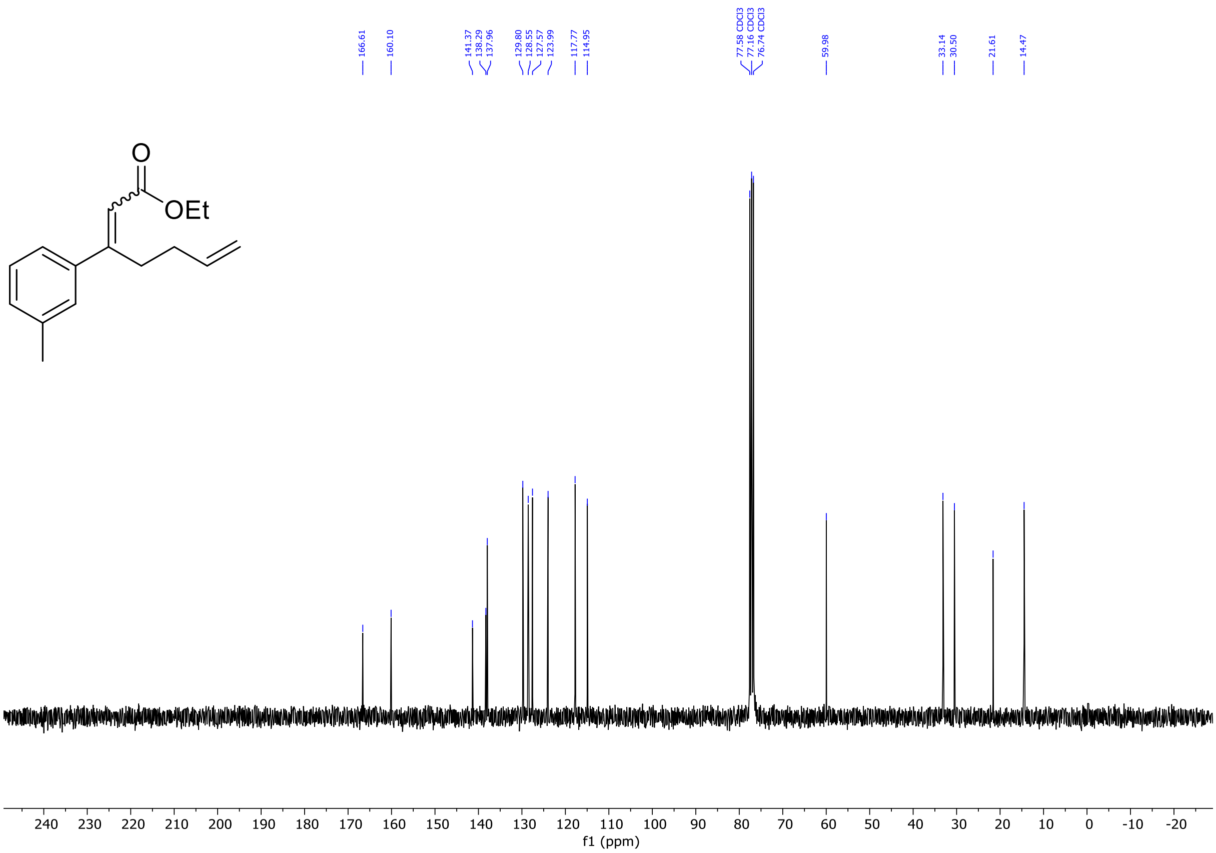

^{1}H-NMR (300 MHz, $CDCl_3$) of major diastereomer of Ethyl 3-(naphthalen-2-yl)hepta-2,6-dienoate (**4h**)

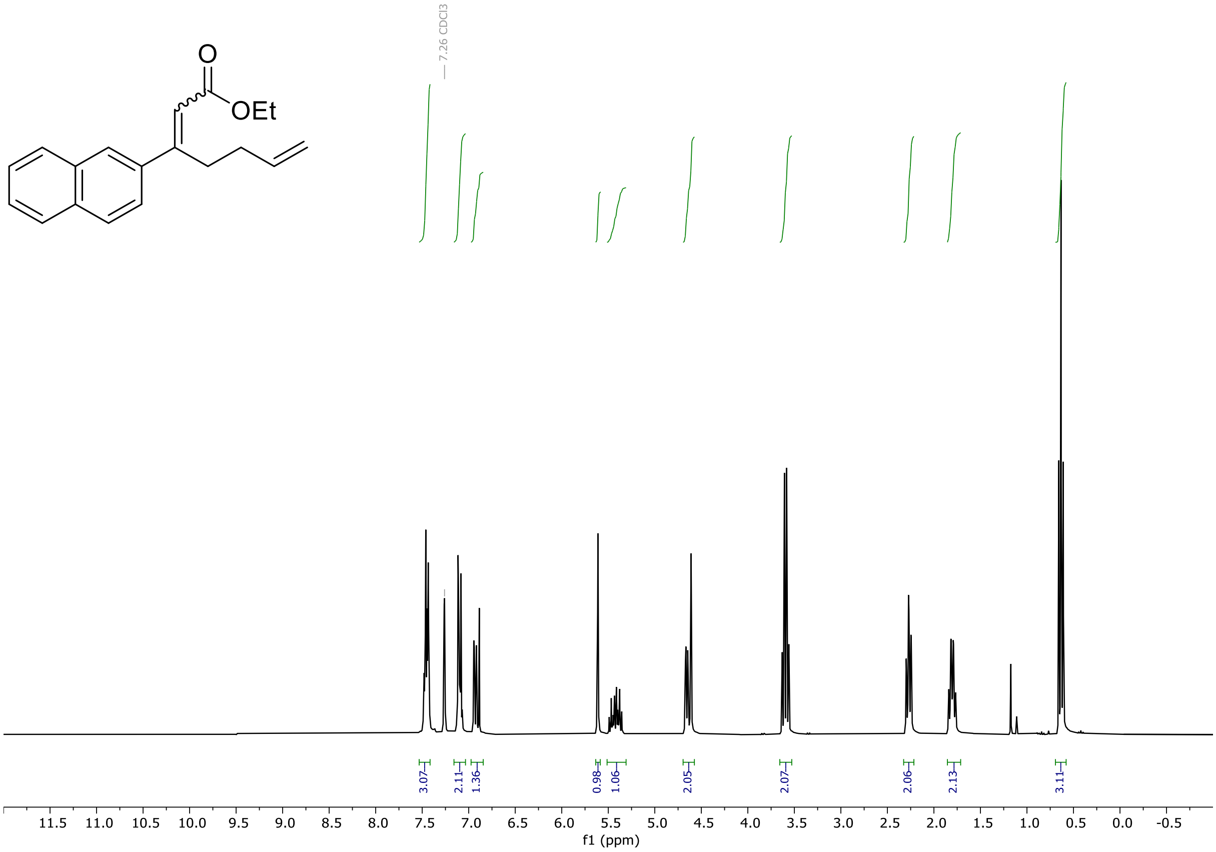

^{13}C-NMR (75 MHz, $CDCl_3$) of major diastereomer of Ethyl 3-(naphthalen-2-yl)hepta-2,6-dienoate (**4h**)

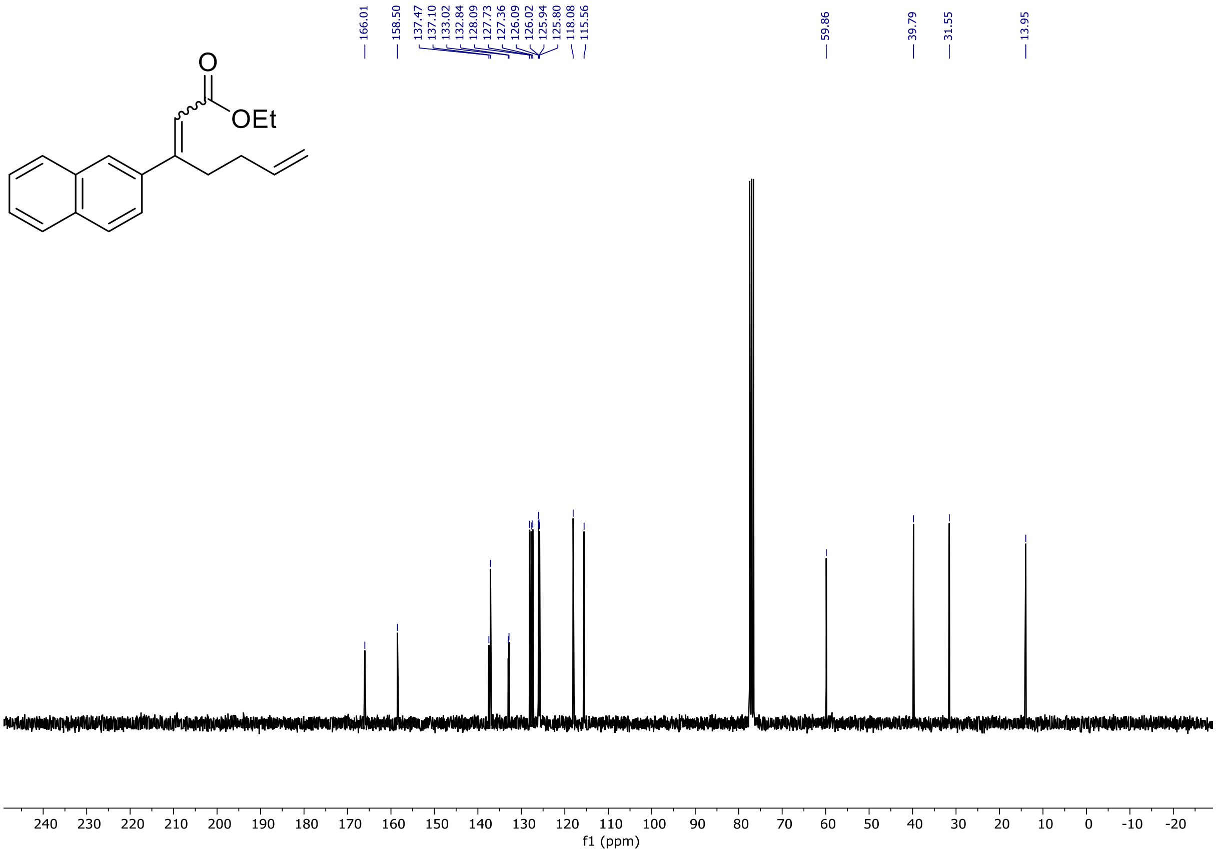

^{1}H-NMR (300 MHz, $CDCl_3$) of minor diastereomer of Ethyl 3-(naphthalen-2-yl)hepta-2,6-dienoate (**4h**)

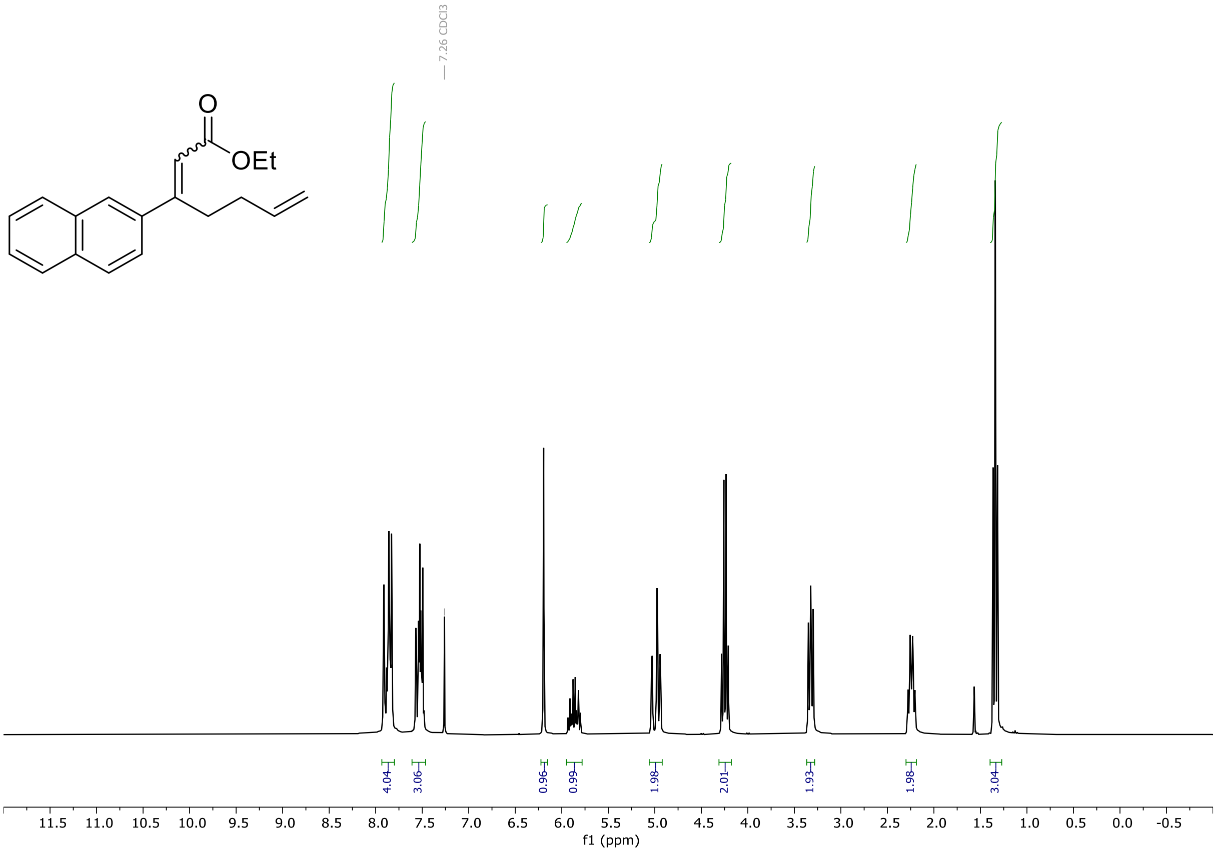

^{13}C-NMR (75 MHz, $CDCl_3$) of minor diastereomer of Ethyl 3-(naphthalen-2-yl)hepta-2,6-dienoate (**4h**)

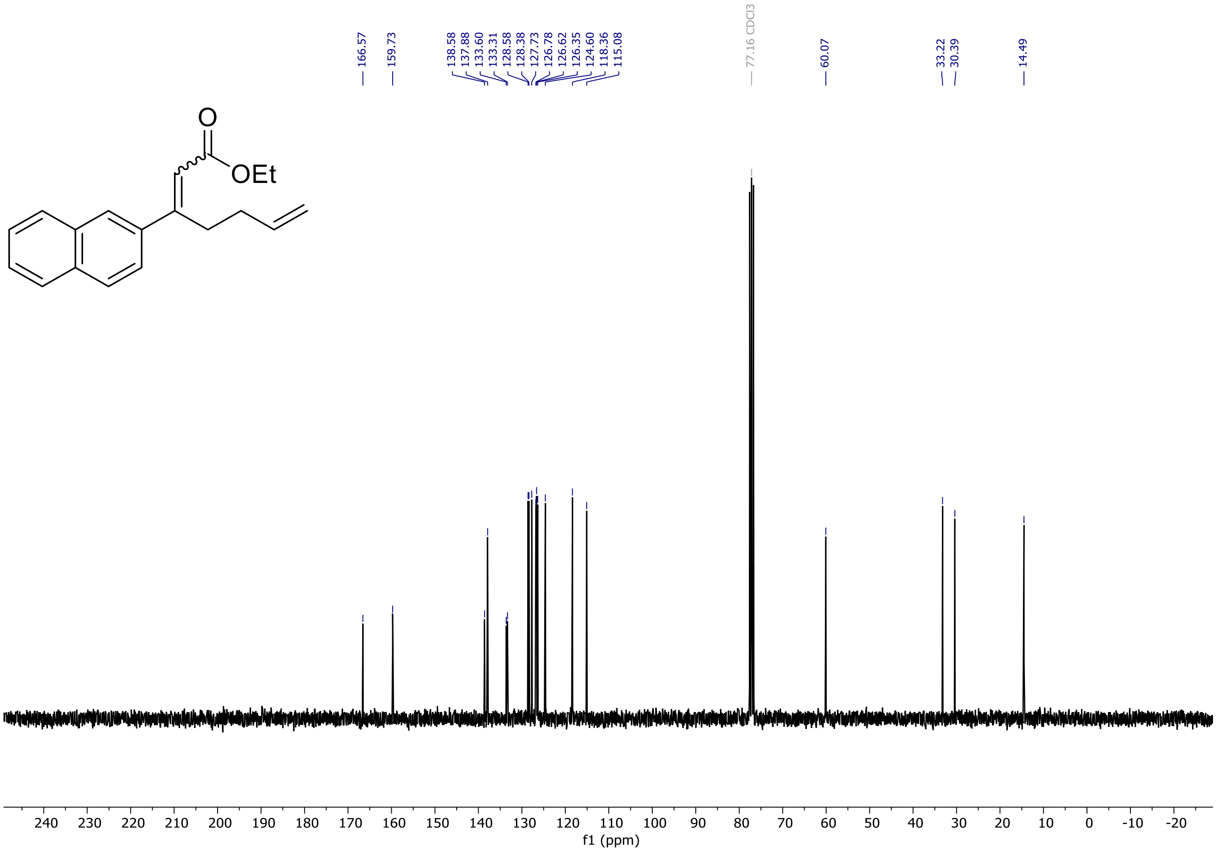

^{1}H-NMR (300 MHz, $CDCl_3$) of minor diastereomer of Ethyl 3-(4-cyanophenyl)hepta-2,6-dienoate (**4i**)

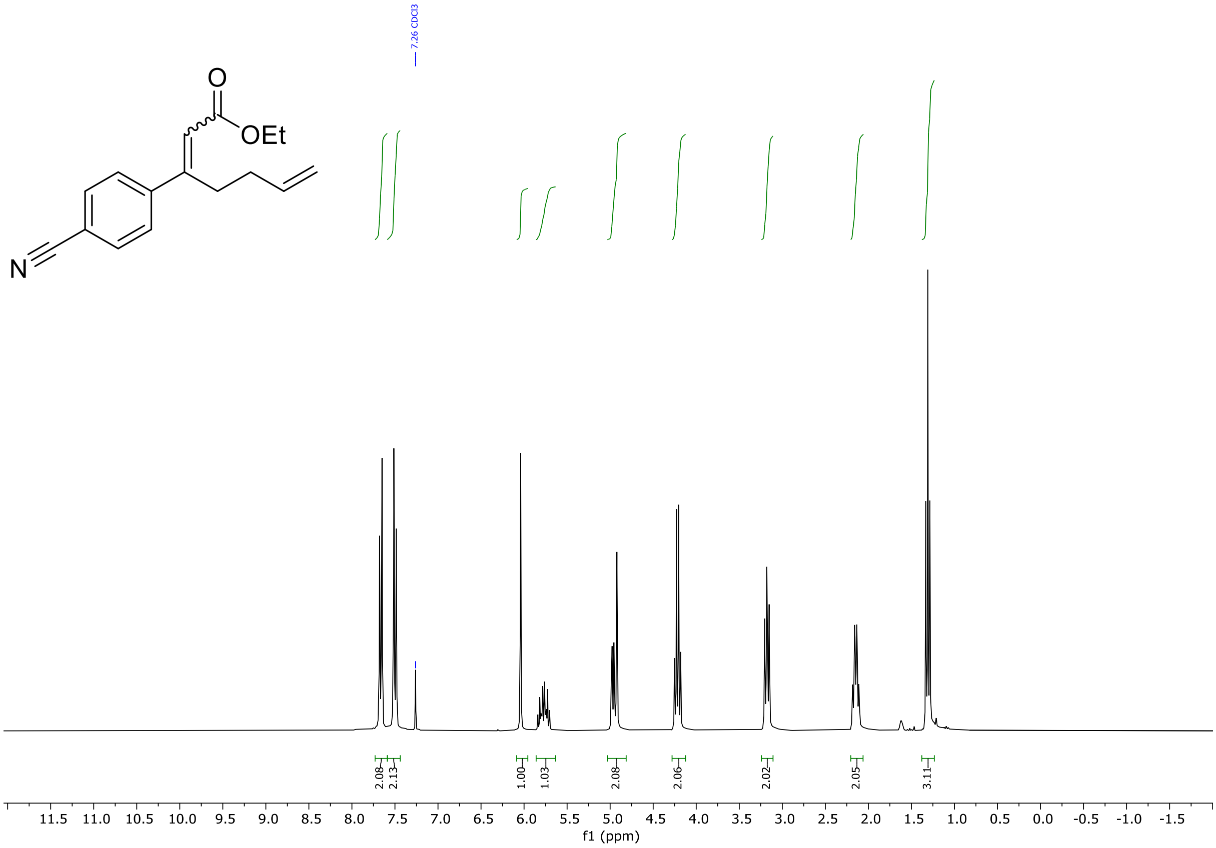

^{13}C-NMR (75 MHz, $CDCl_3$) of minor diastereomer of Ethyl 3-(4-cyanophenyl)hepta-2,6-dienoate (**4i**)

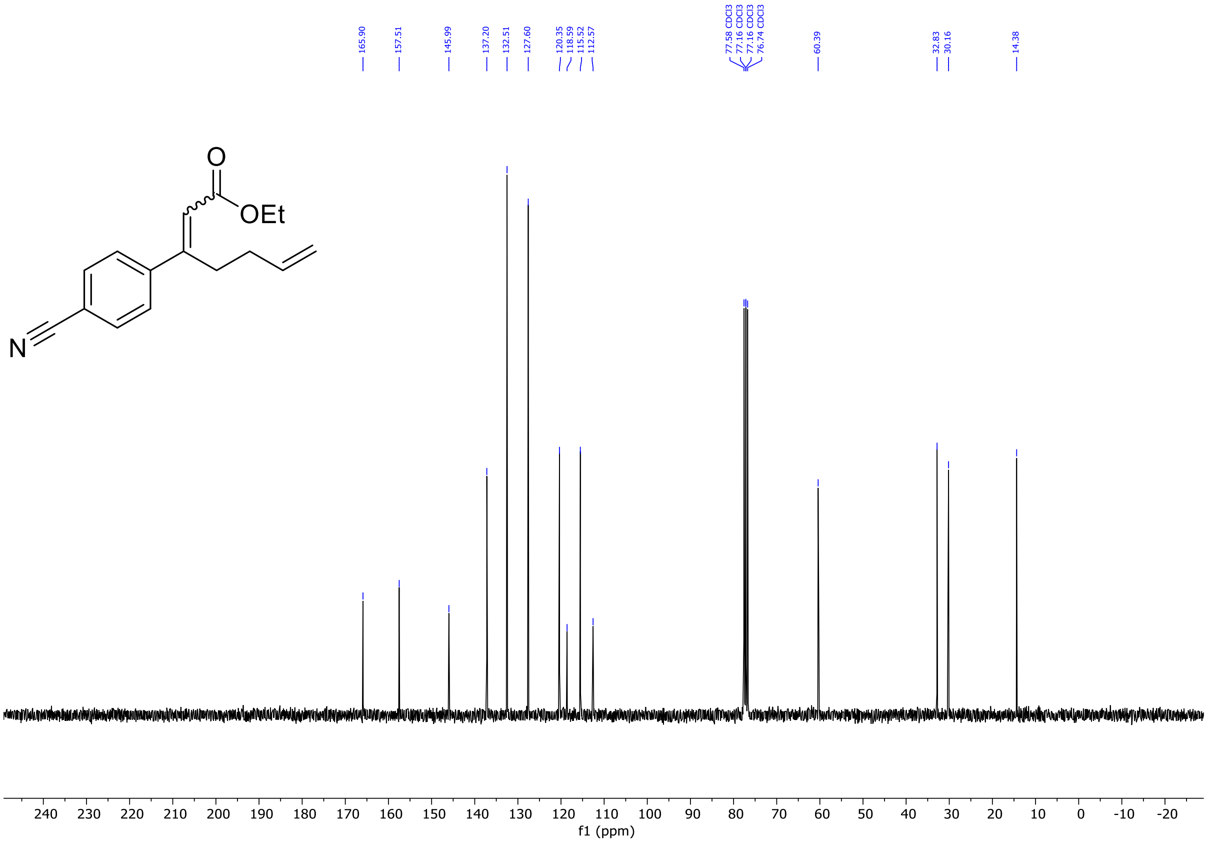

^{1}H-NMR (600 MHz, $CDCl_3$) of *(E)*-4-Phenylocta-3,7-dien-2-one (**1a**)

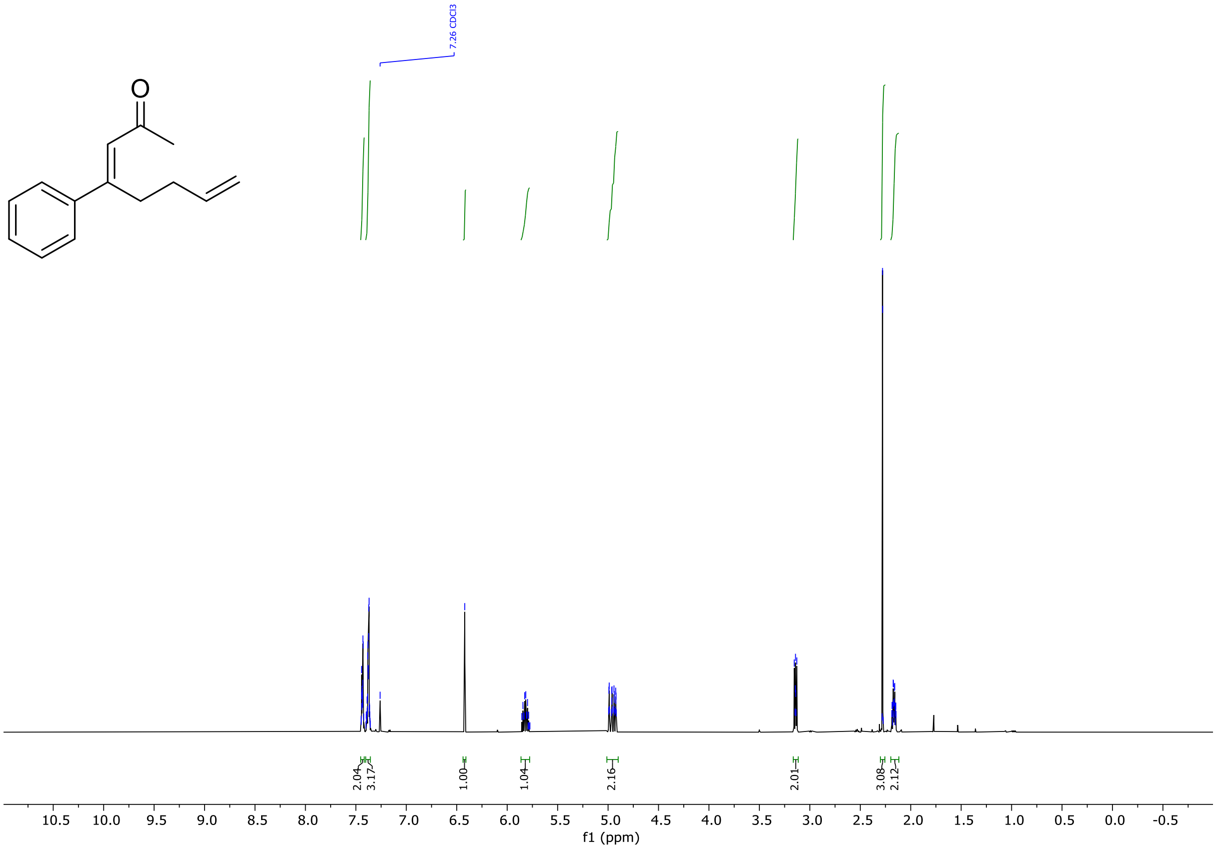

^{13}C-NMR (151 MHz, $CDCl_3$) of *(E)*-4-Phenylocta-3,7-dien-2-one (**1a**)

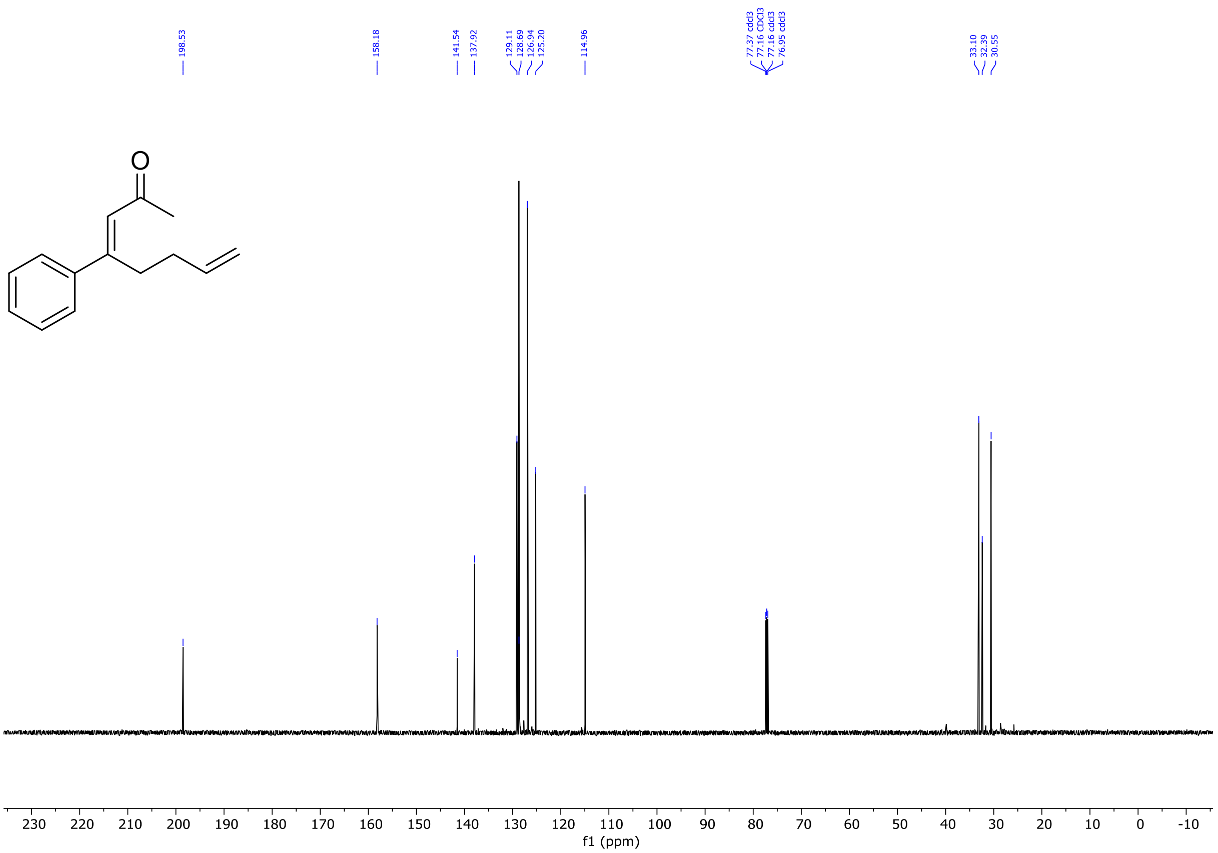

^{1}H-NMR (600 MHz, $CDCl_3$) of *(Z)*-4-Phenylocta-3,7-dien-2-one (**1a**)

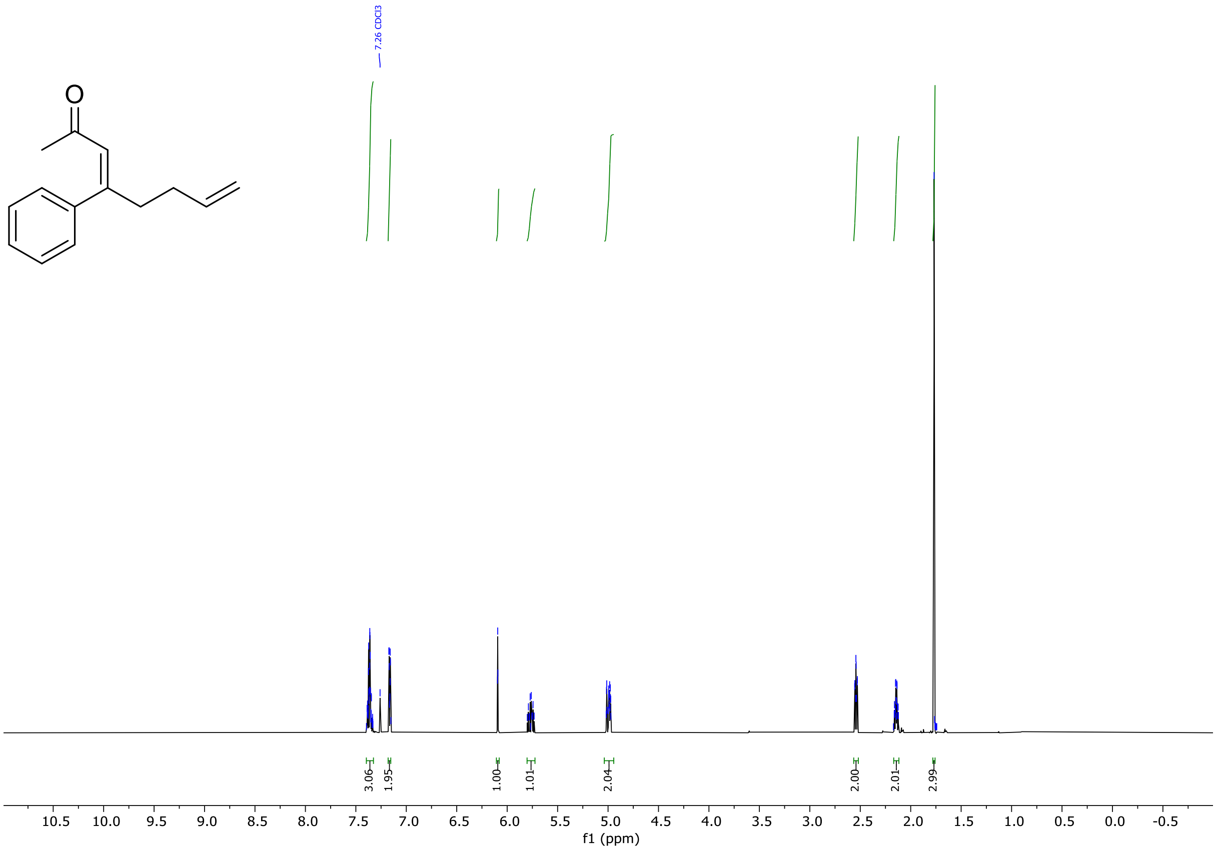

^{13}C-NMR (151 MHz, $CDCl_3$) of *(Z)*-4-Phenylocta-3,7-dien-2-one (**1a**)

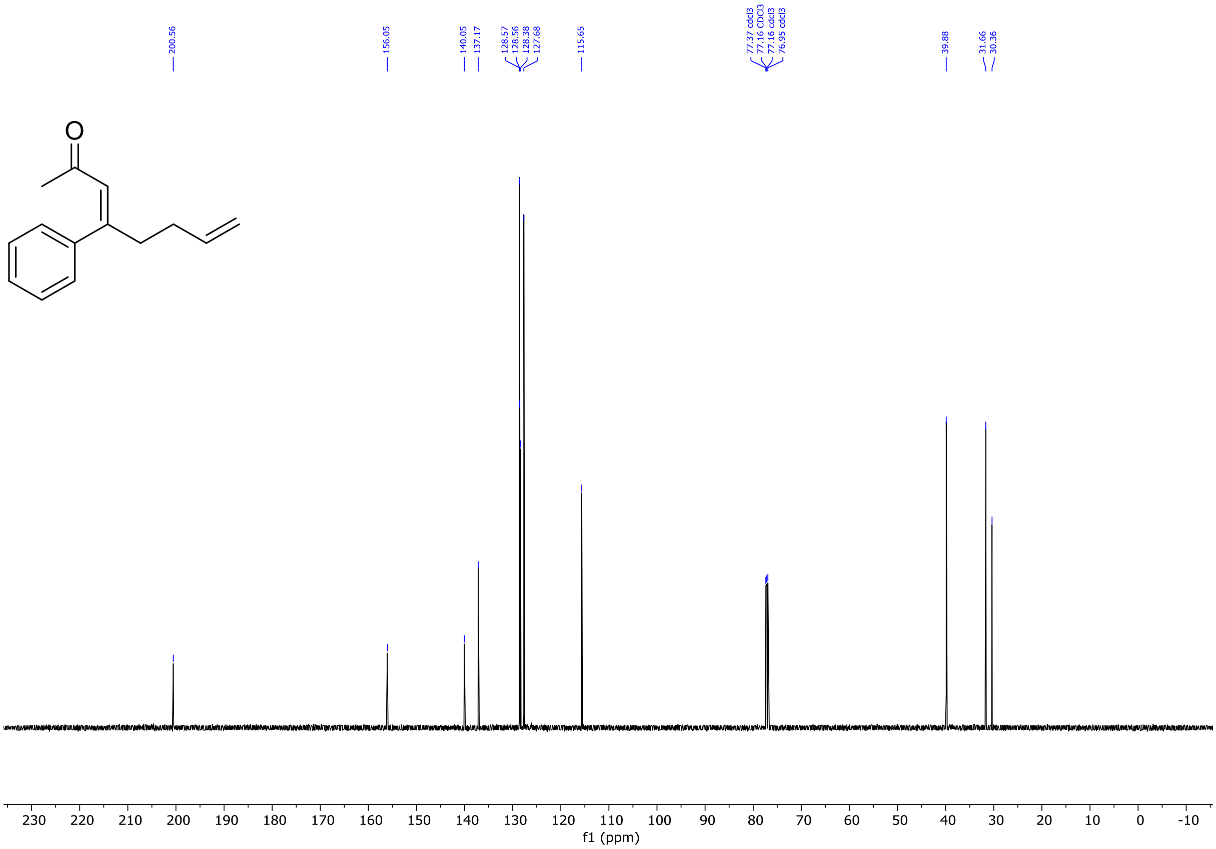

^{1}H-NMR (300 MHz, $CDCl_3$) of (*E*)-4-(4-fluorophenyl)octa-3,7-dien-2-one (**1b**)

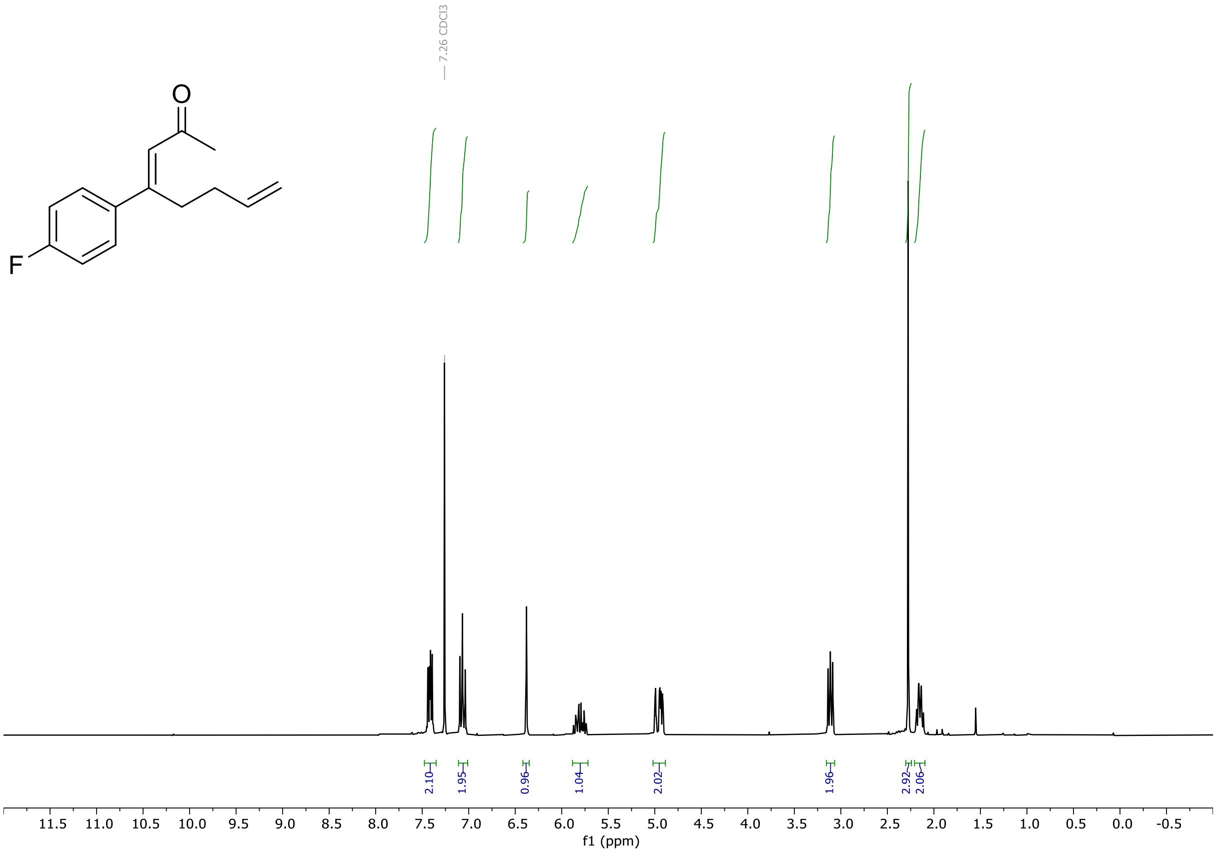

^{13}C-NMR (75 MHz, $CDCl_3$) of (*E*)-4-(4-fluorophenyl)octa-3,7-dien-2-one (**1b**)

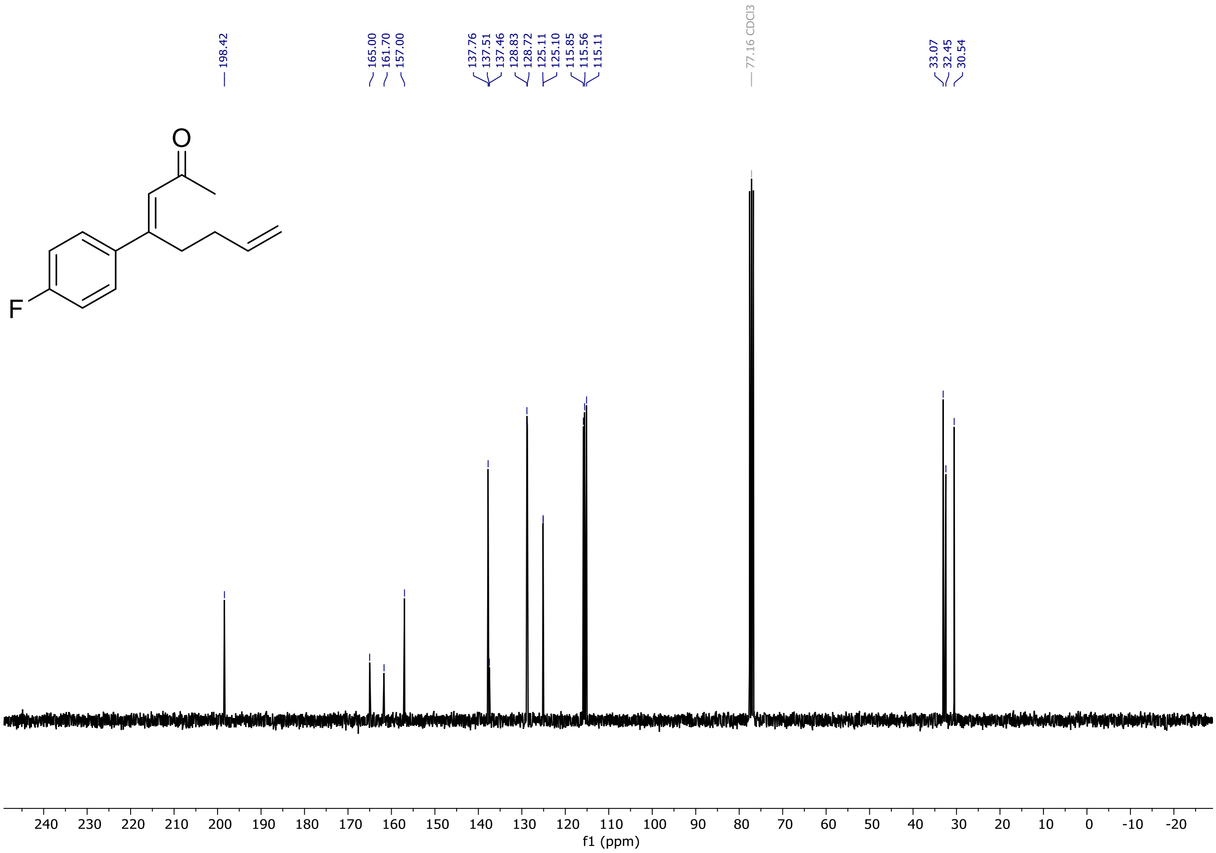

^{19}F-NMR (282 MHz, $CDCl_3$) of (*E*)-4-(4-fluorophenyl)octa-3,7-dien-2-one (**1b**)

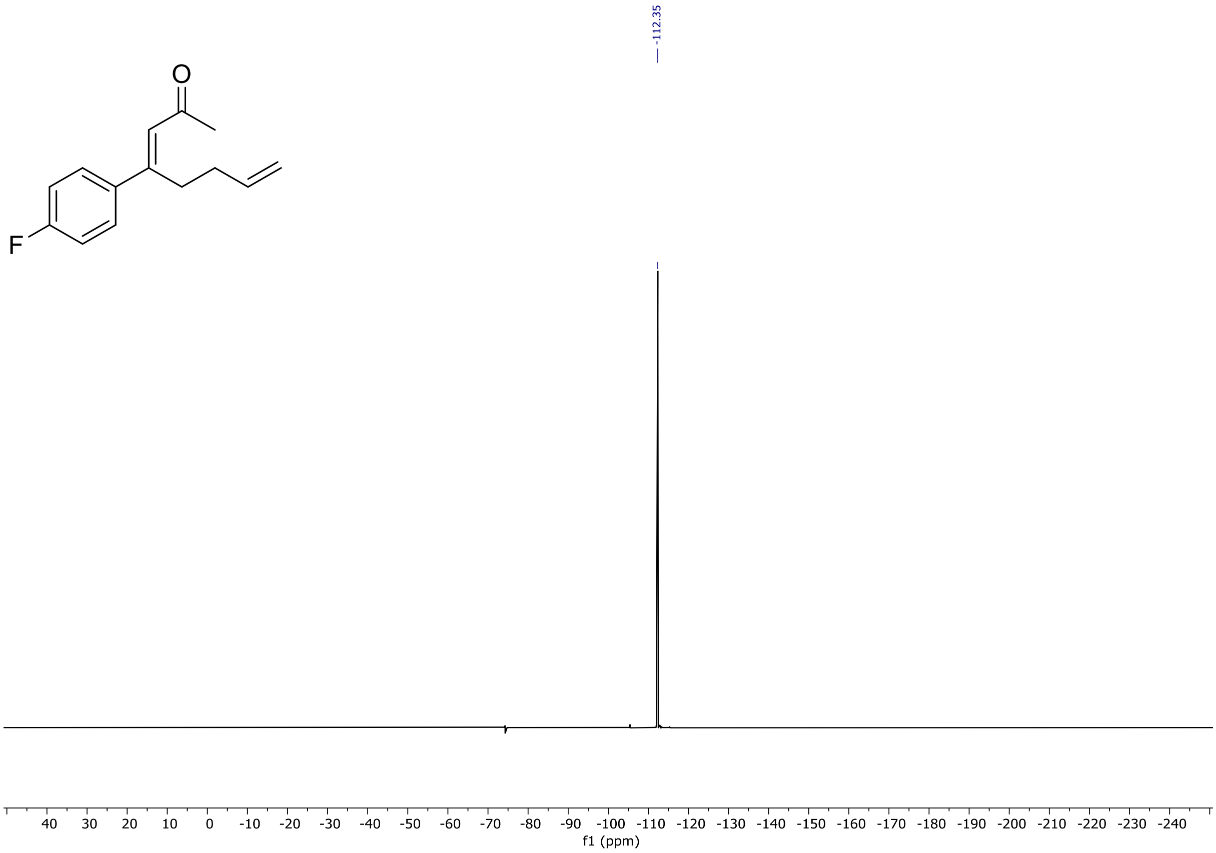

^{1}H-NMR (300 MHz, $CDCl_3$) of (*Z*)-4-(4-fluorophenyl)octa-3,7-dien-2-one (**1b**)

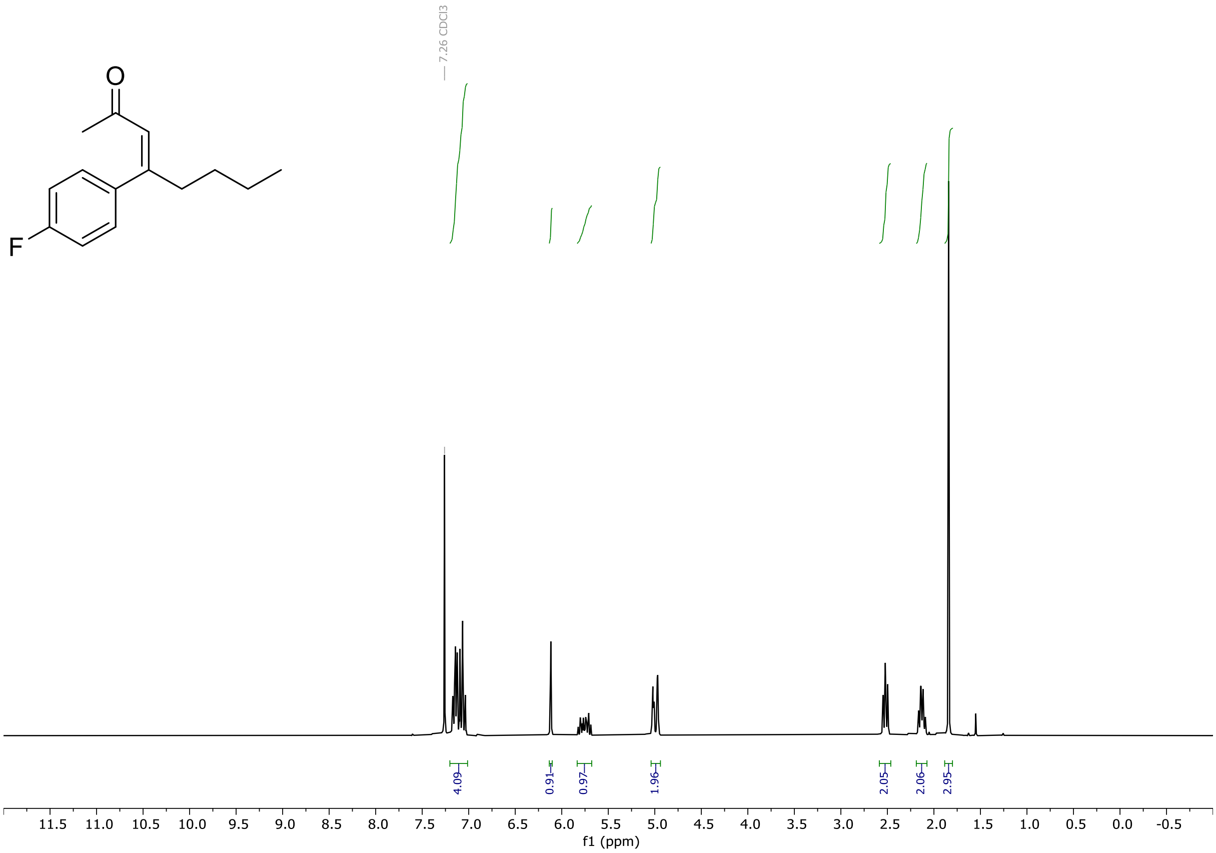

^{13}C-NMR (75 MHz, $CDCl_3$) of (*Z*)-4-(4-fluorophenyl)octa-3,7-dien-2-one (**1b**)

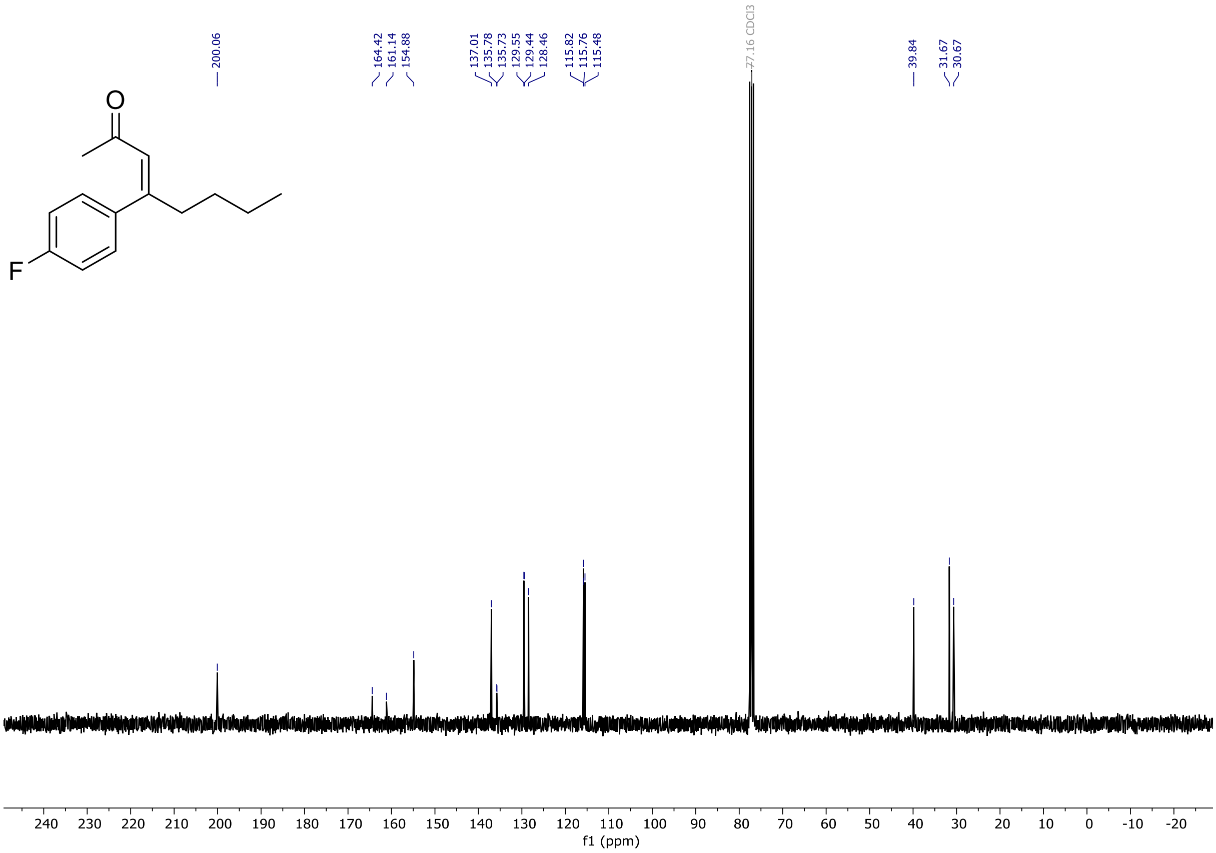

^{19}F-NMR (282 MHz, $CDCl_3$) of (Z)-4-(4-fluorophenyl)octa-3,7-dien-2-one (**1b**)

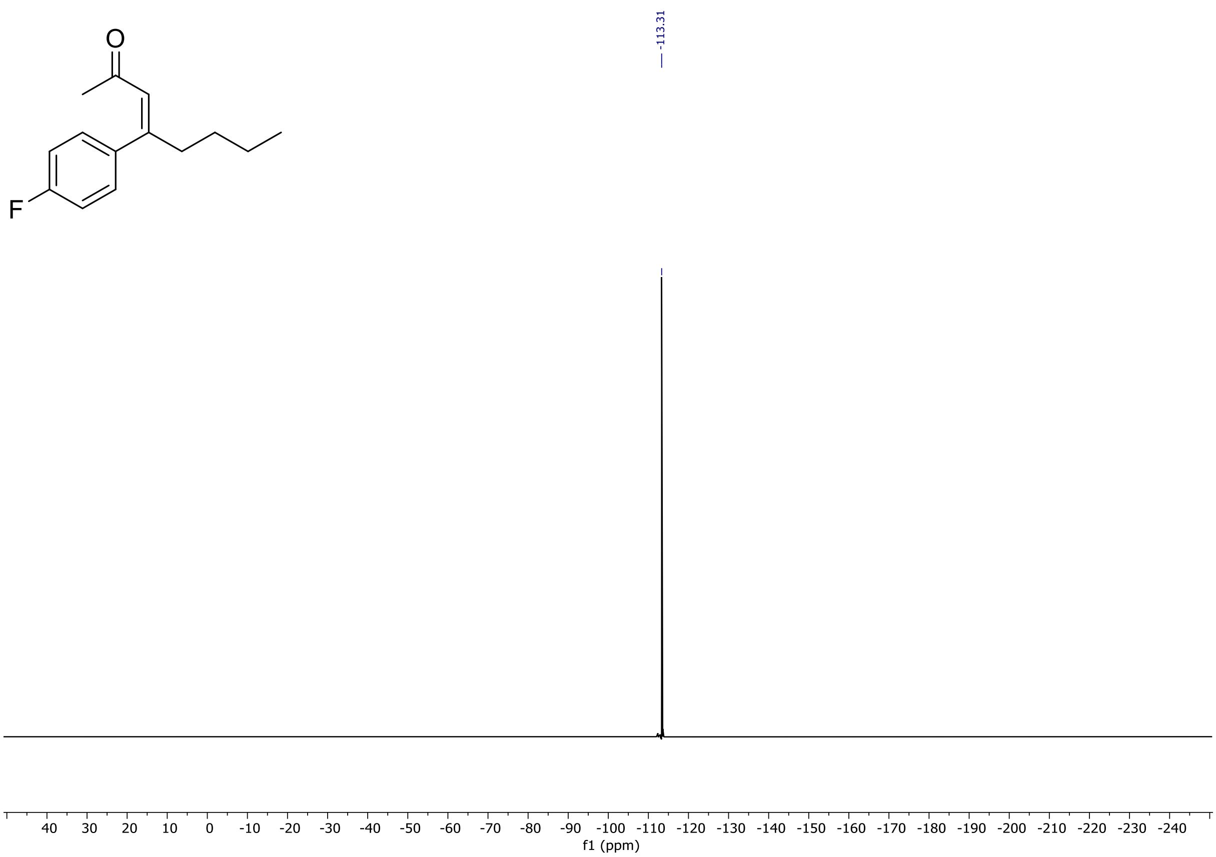

^{1}H-NMR (300 MHz, $CDCl_3$) of *(E)*-4-(4-chlorophenyl)octa-3,7-dien-2-one (**1c**)

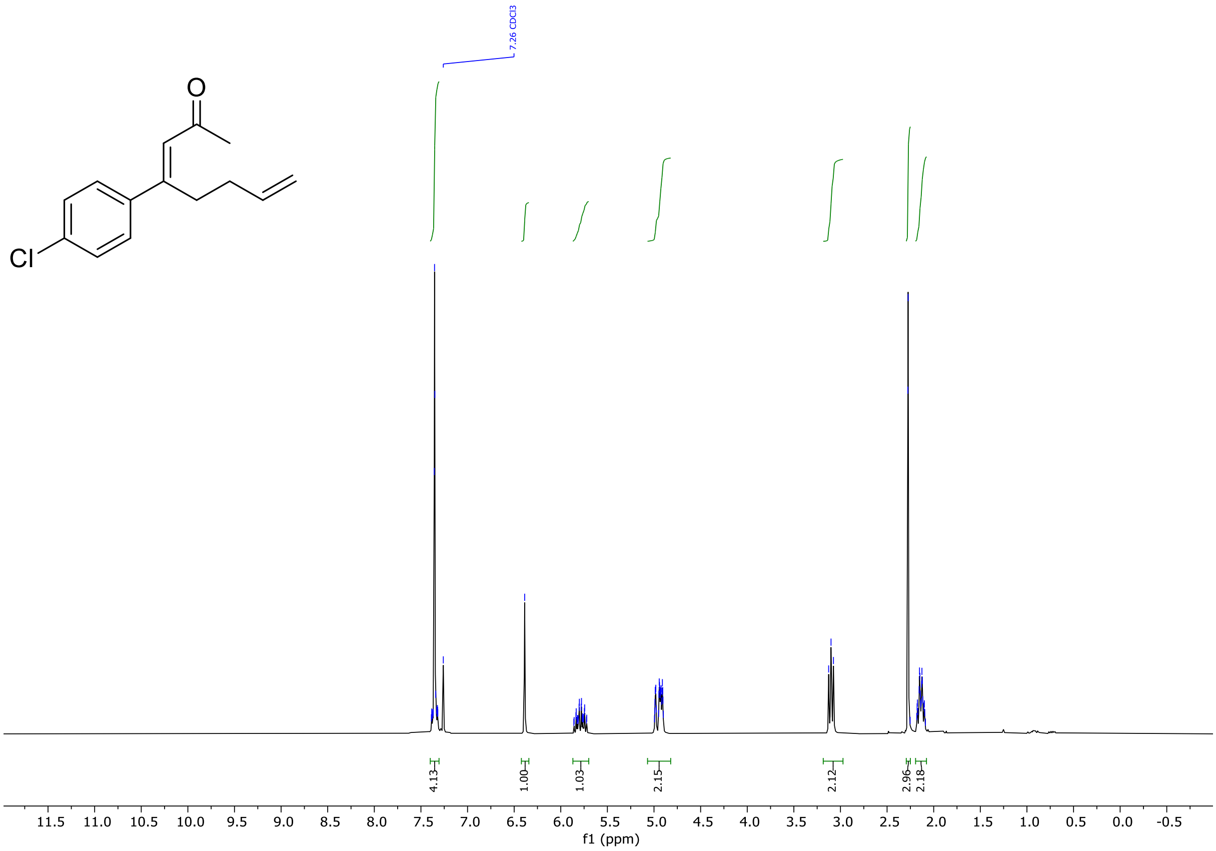

^{13}C-NMR (75 MHz, $CDCl_3$) of *(E)*-4-(4-chlorophenyl)octa-3,7-dien-2-one (**1c**)

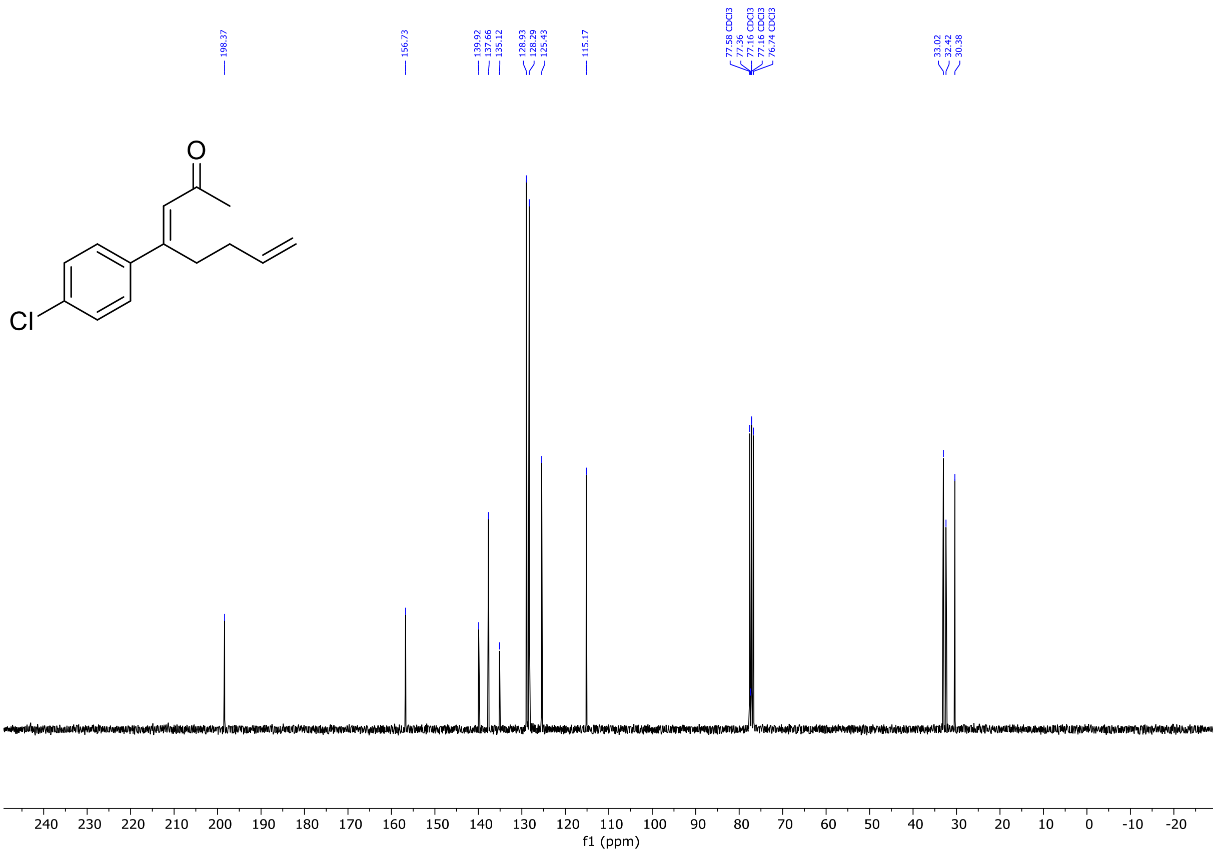

^{1}H-NMR (300 MHz, $CDCl_3$) of *(Z)*-4-(4-chlorophenyl)octa-3,7-dien-2-one (**1c**)

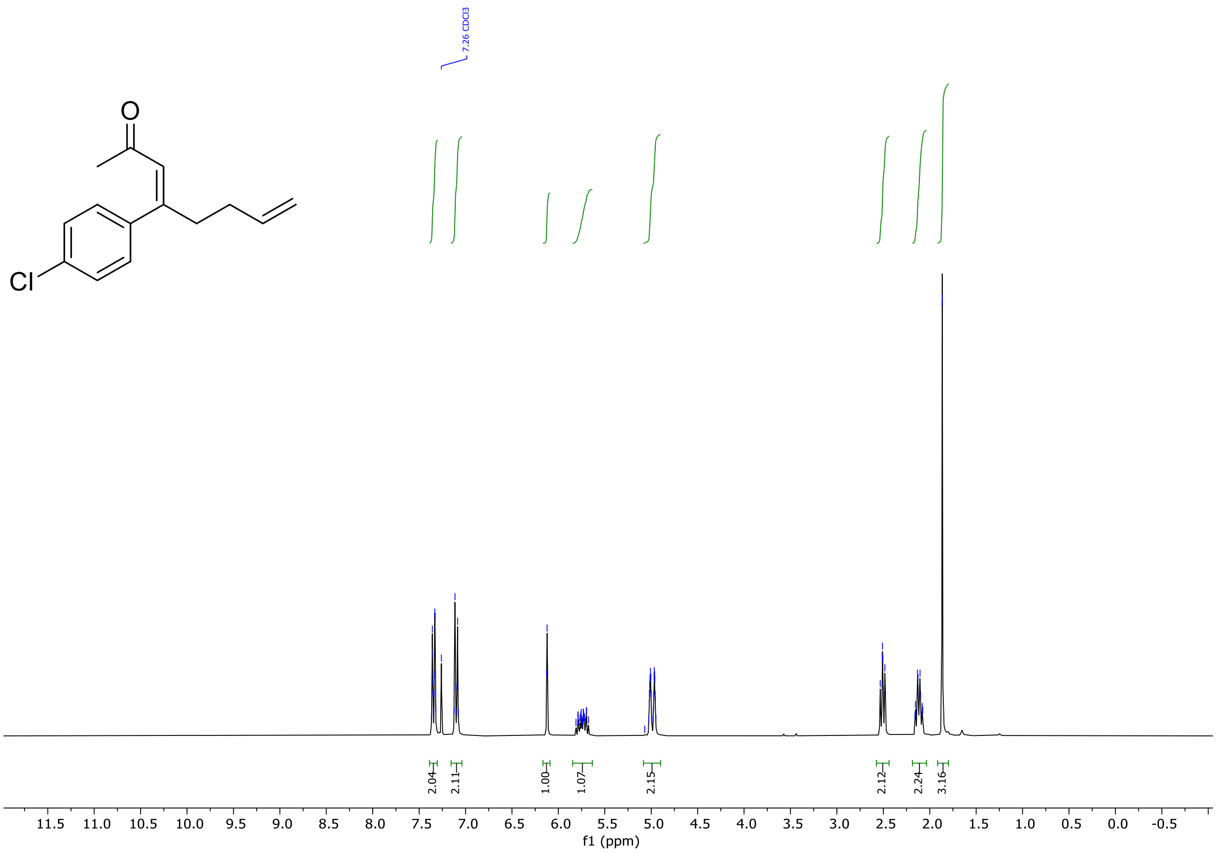

^{13}C-NMR (75 MHz, $CDCl_3$) of *(Z)*-4-(4-chlorophenyl)octa-3,7-dien-2-one (**1c**)

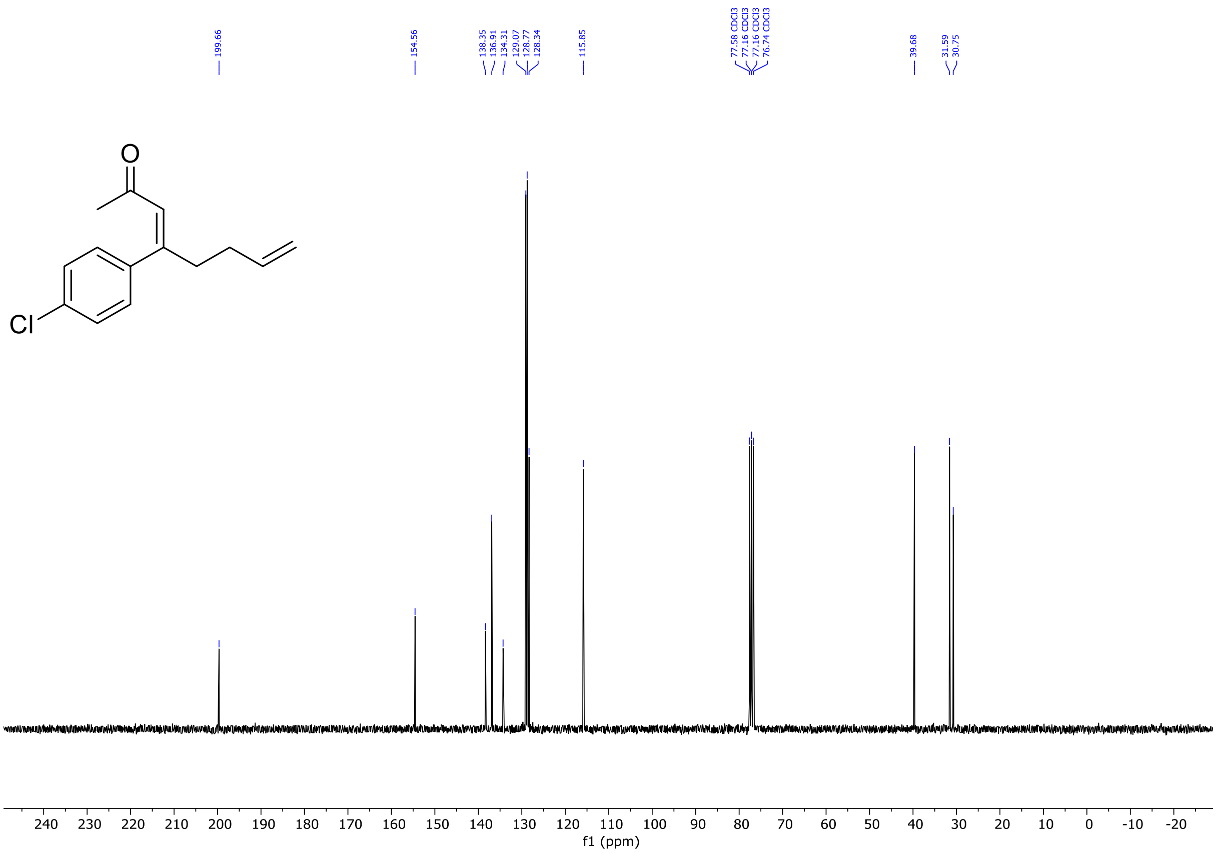

^{1}H-NMR (300 MHz, $CDCl_3$) of (*E*)-4-(4-bromophenyl)octa-3,7-dien-2-one (**1d**)

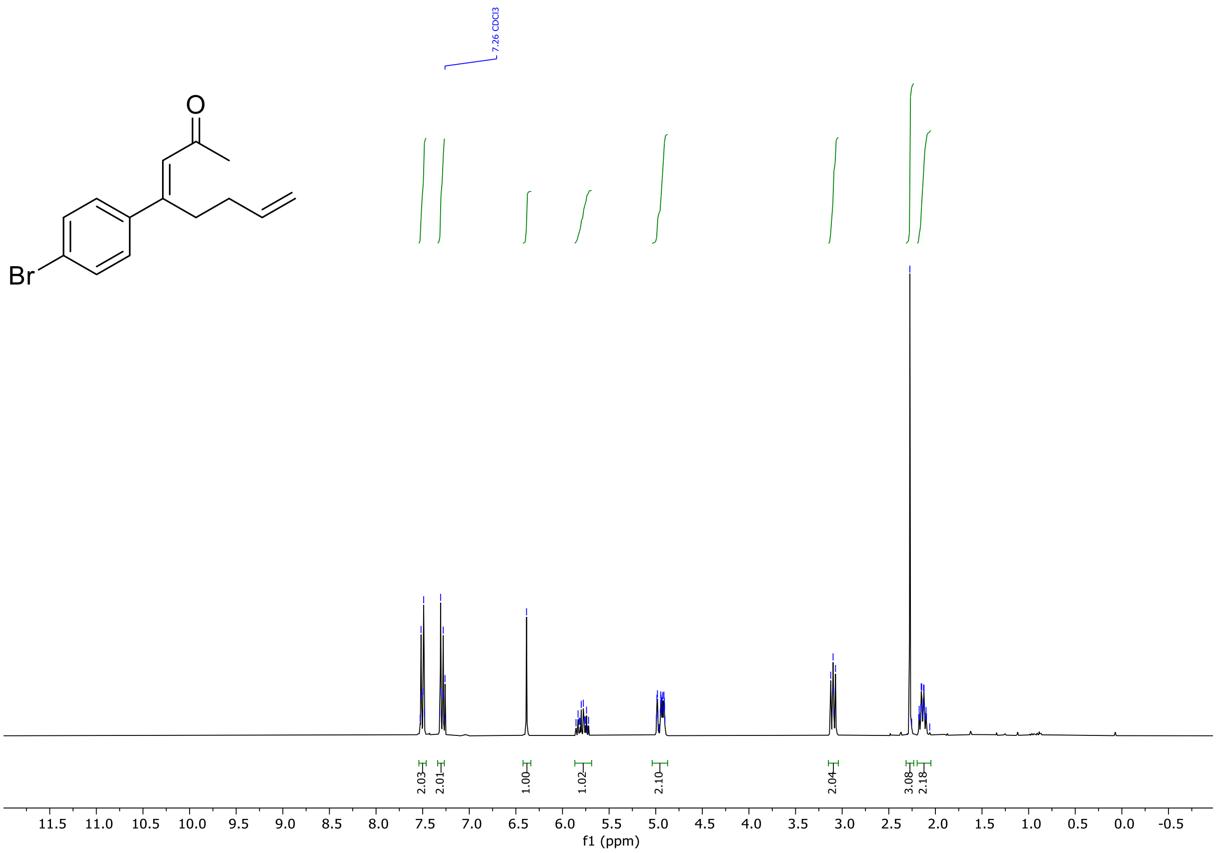

^{13}C-NMR (75 MHz, $CDCl_3$) of (*E*)-4-(4-bromophenyl)octa-3,7-dien-2-one (**1d**)

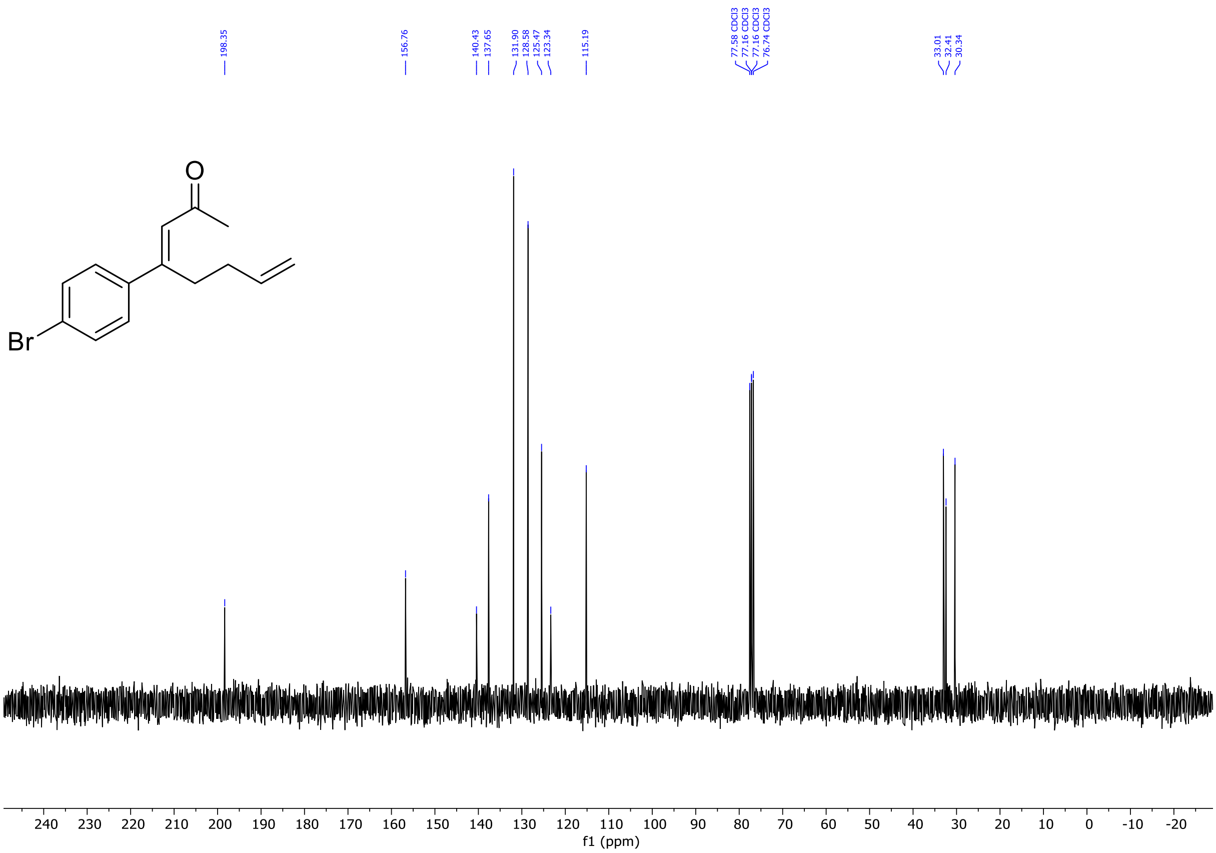

^{1}H-NMR (300 MHz, $CDCl_3$) of (*Z*)-4-(4-bromophenyl)octa-3,7-dien-2-one (**1d**)

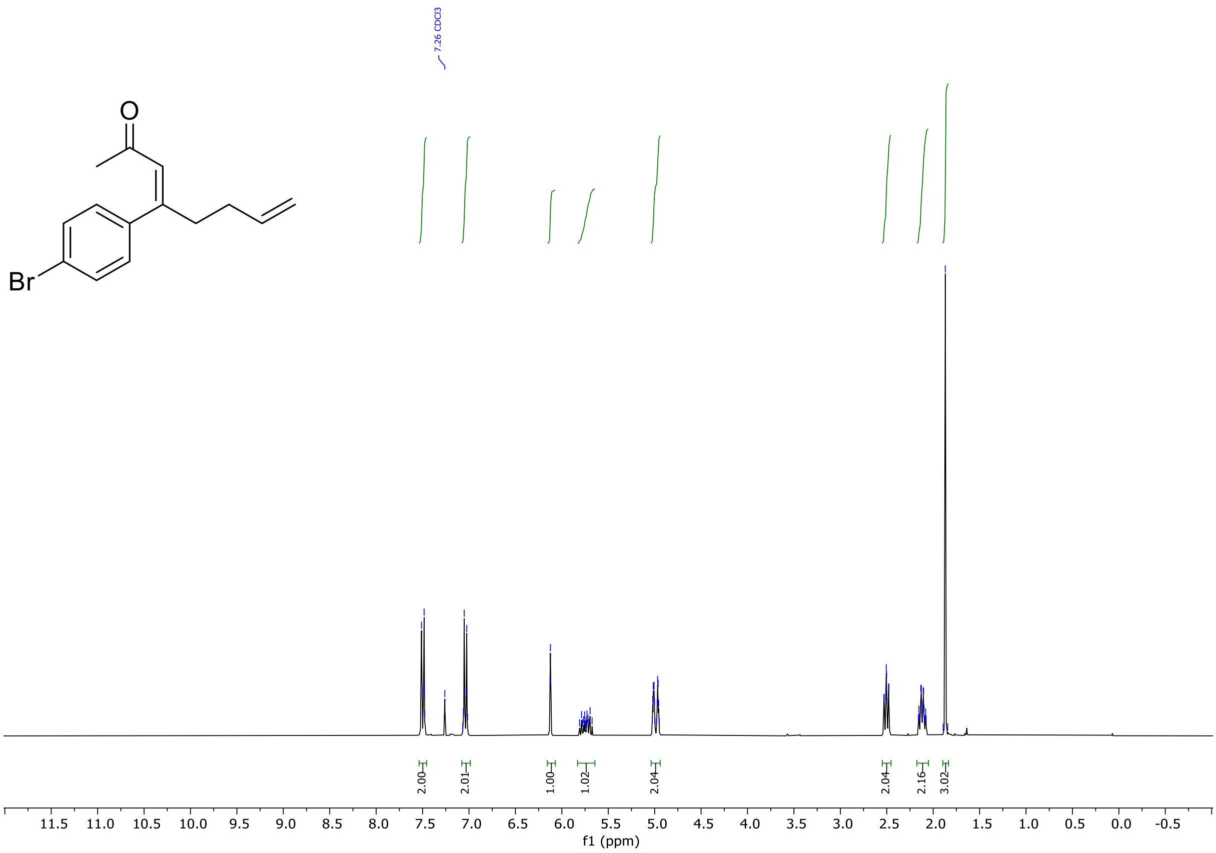

^{13}C-NMR (75 MHz, $CDCl_3$) of (*Z*)-4-(4-bromophenyl)octa-3,7-dien-2-one (**1d**)

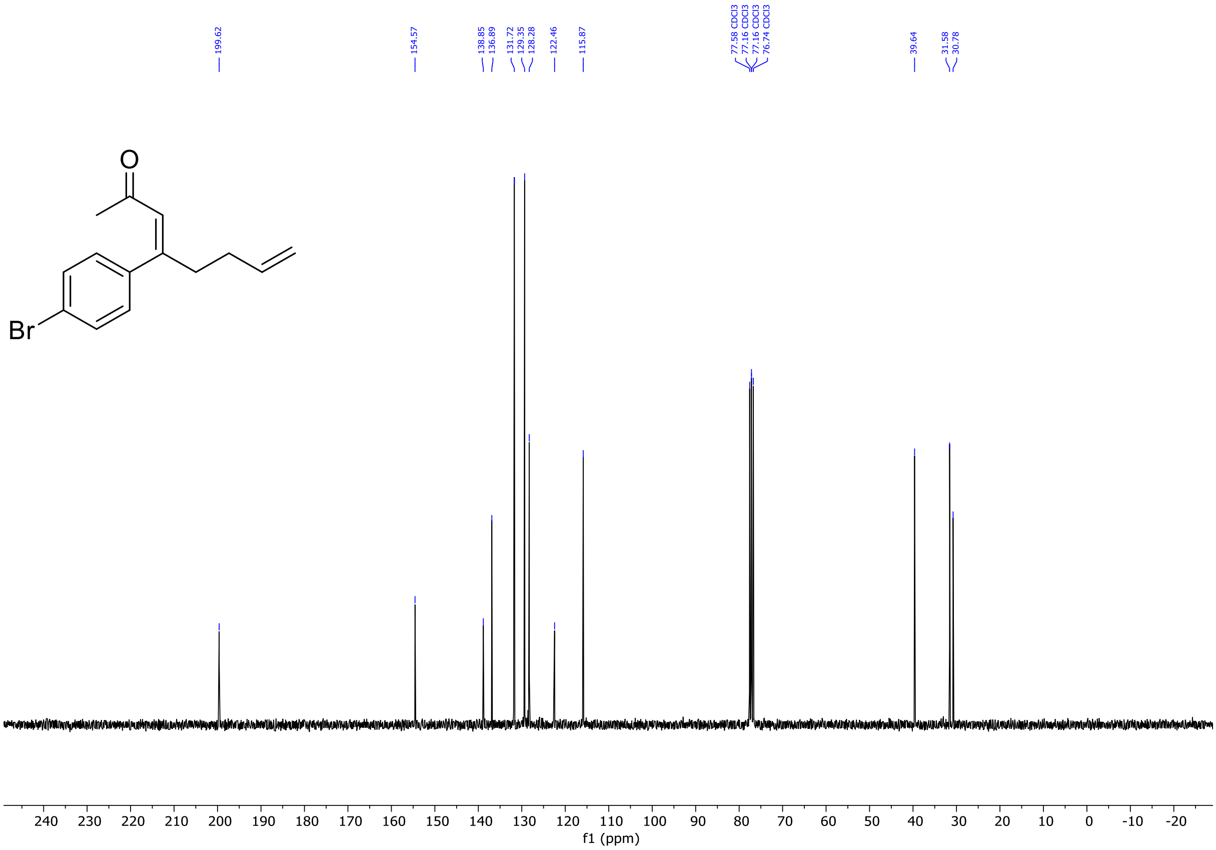

^{1}H-NMR (300 MHz, $CDCl_3$) of *(E)*- 4-(4-(trifluoromethyl)phenyl)octa-3,7-dien-2-one (**1e**)

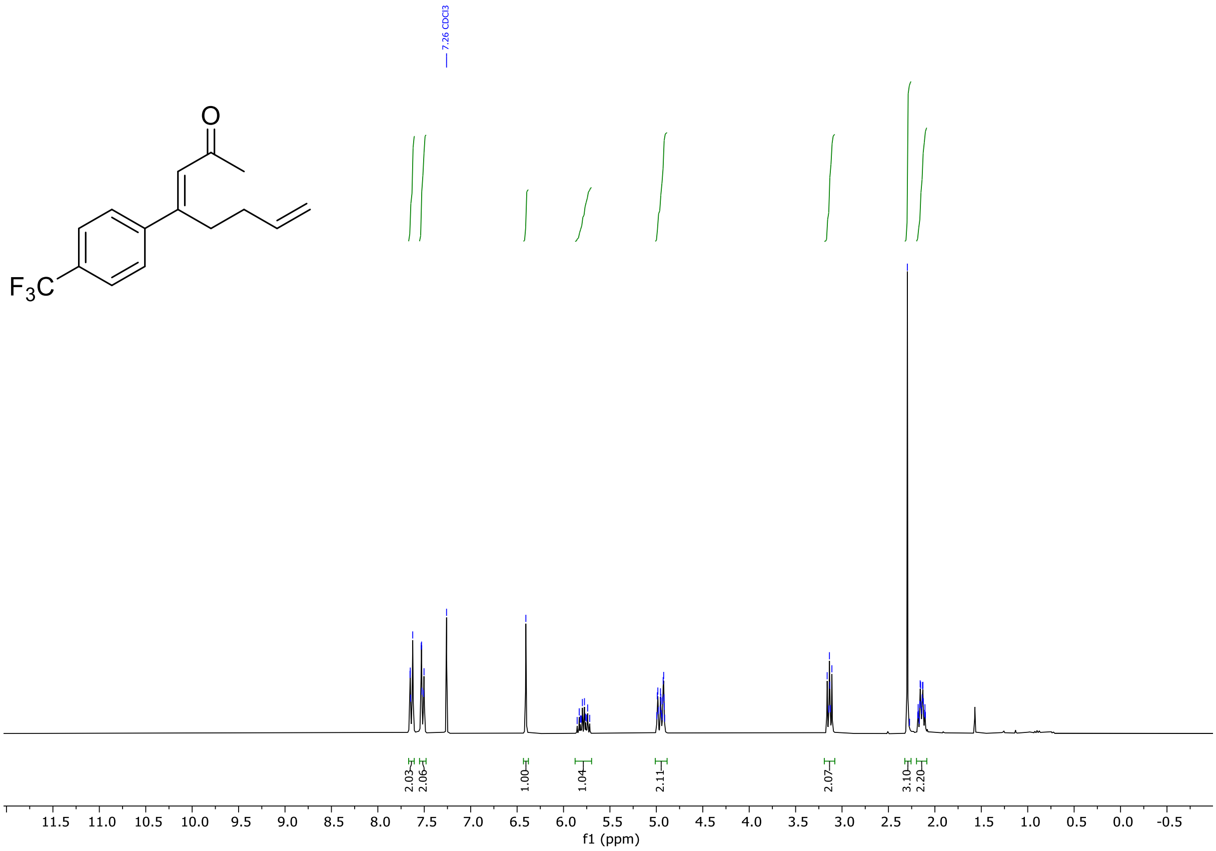

^{13}C-NMR (75 MHz, $CDCl_3$) of *(E)*- 4-(4-(trifluoromethyl)phenyl)octa-3,7-dien-2-one (**1e**)

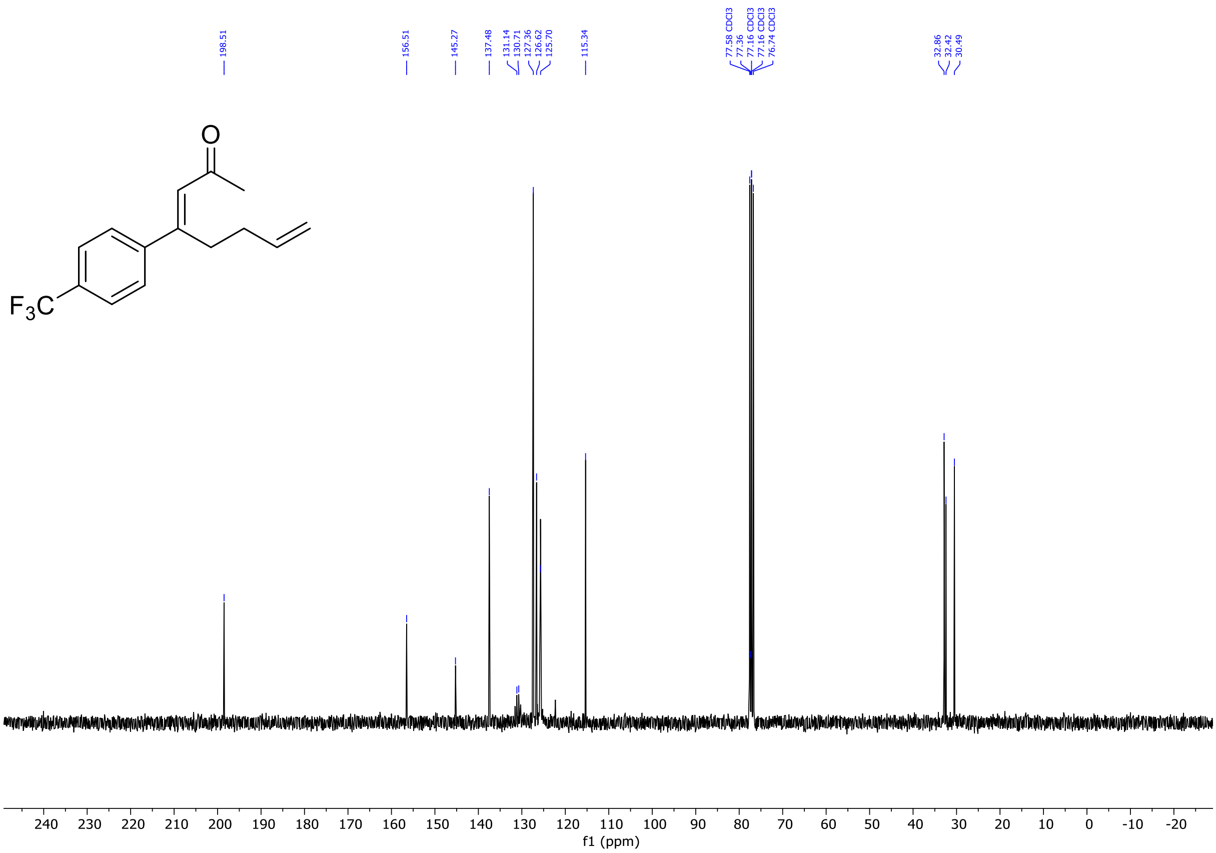

^{19}F-NMR (282 MHz, $CDCl_3$) of *(E)*- 4-(4-(trifluoromethyl)phenyl)octa-3,7-dien-2-one (**1e**)

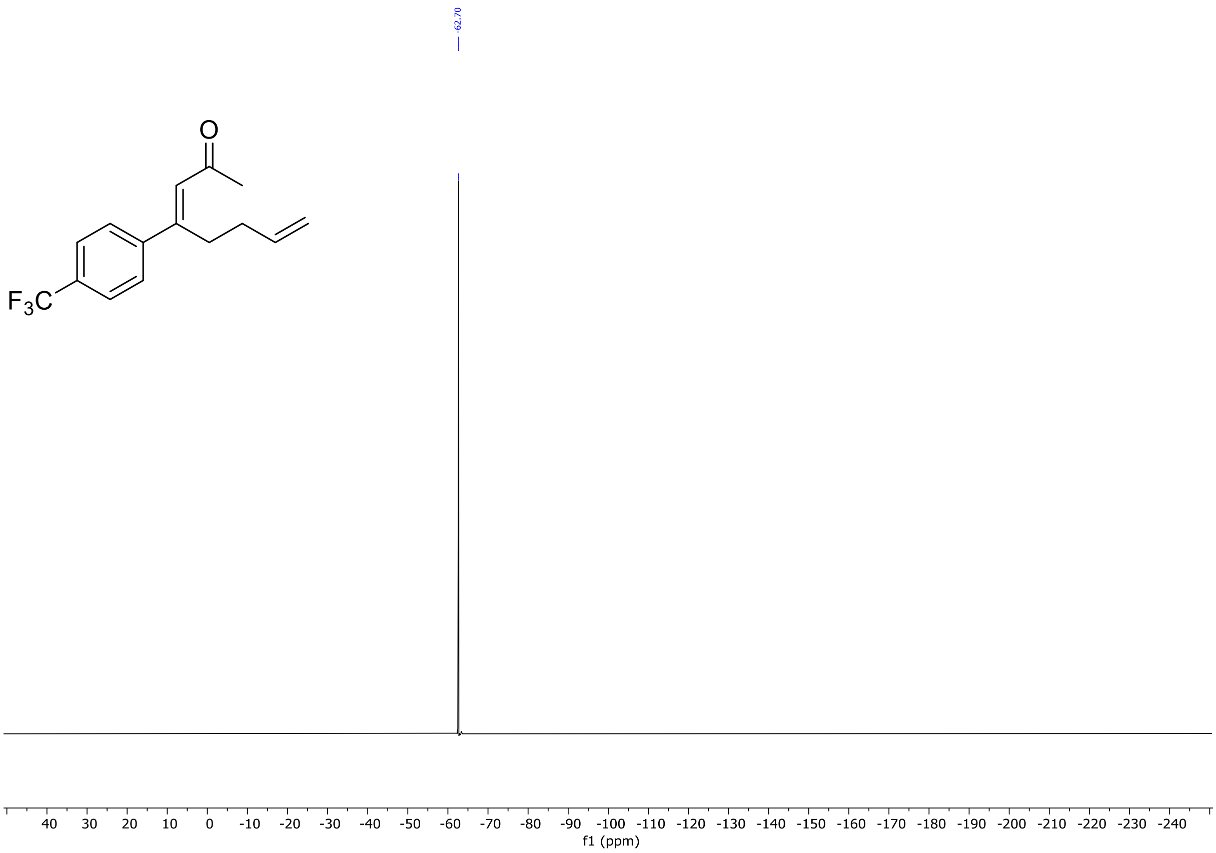

^{1}H-NMR (300 MHz, $CDCl_3$) of *(Z)*- 4-(4-(trifluoromethyl)phenyl)octa-3,7-dien-2-one (**1e**)

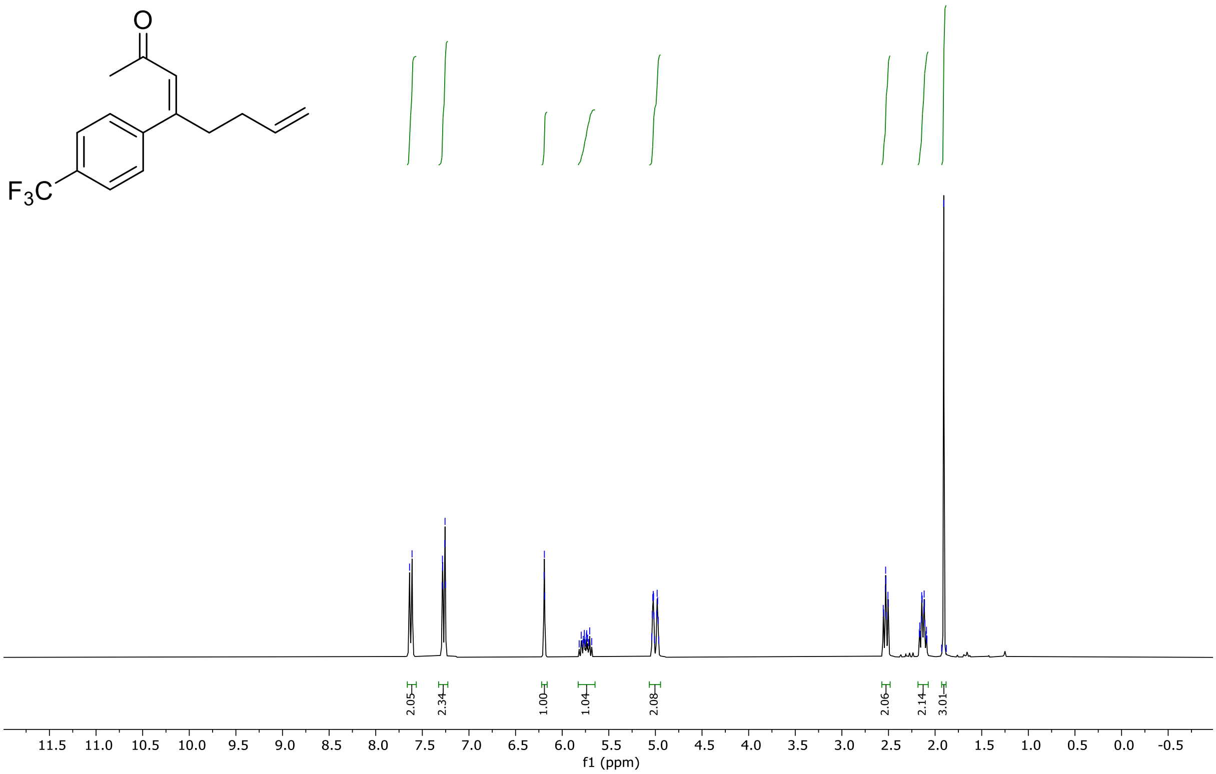

^{13}C-NMR (75 MHz, $CDCl_3$) of *(Z)*- 4-(4-(trifluoromethyl)phenyl)octa-3,7-dien-2-one (**1e**)

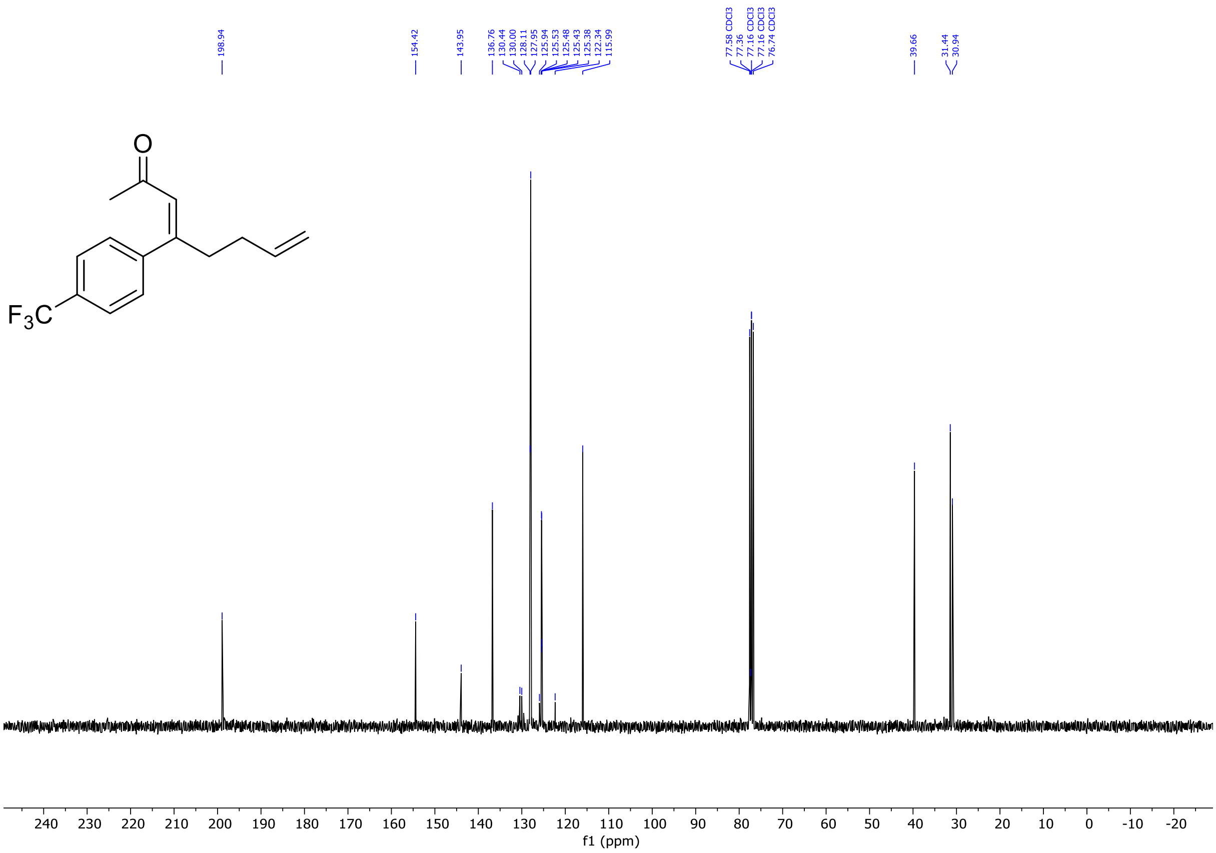

^{19}F-NMR (282 MHz, $CDCl_3$) of *(Z)*- 4-(4-(trifluoromethyl)phenyl)octa-3,7-dien-2-one (**1e**)

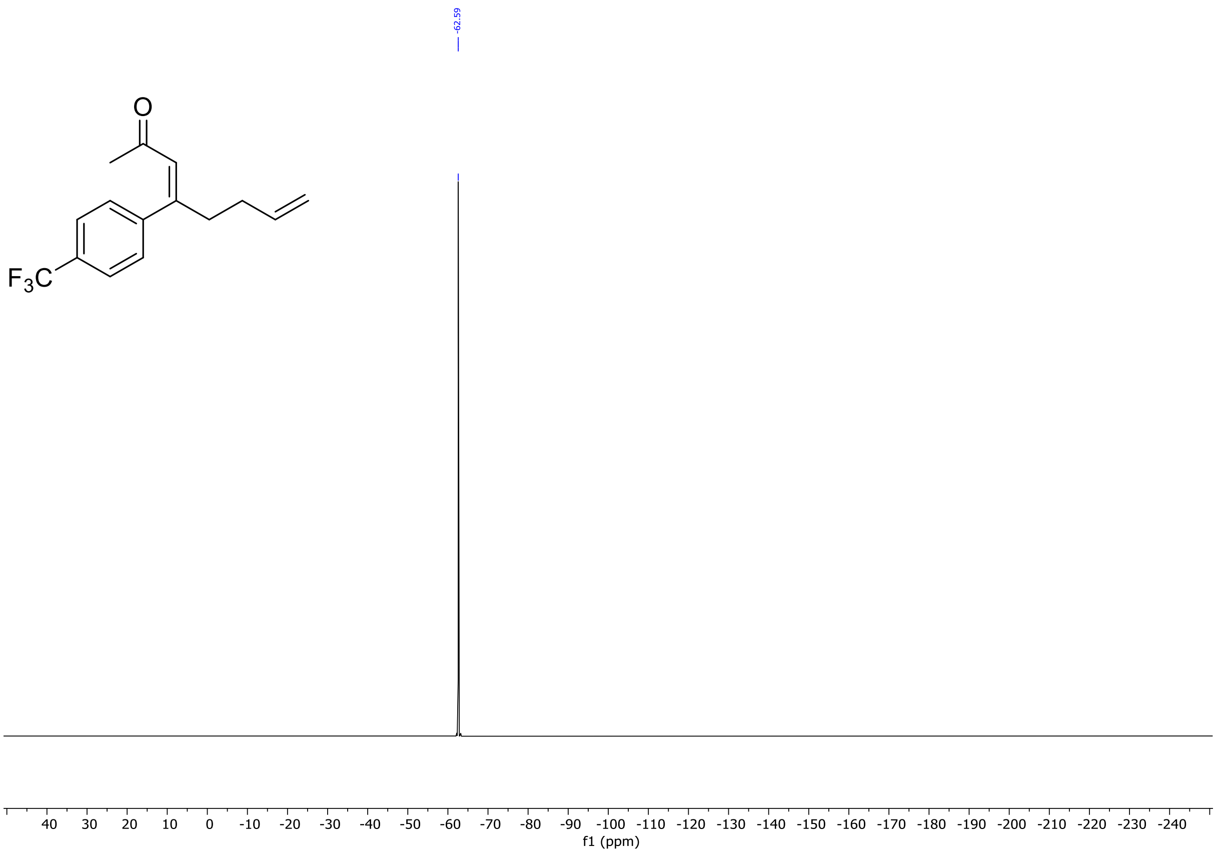

^{1}H-NMR (300 MHz, $CDCl_3$) of (*E*)-4-(*p*-tolyl)octa-3,7-dien-2-one (**1f**)

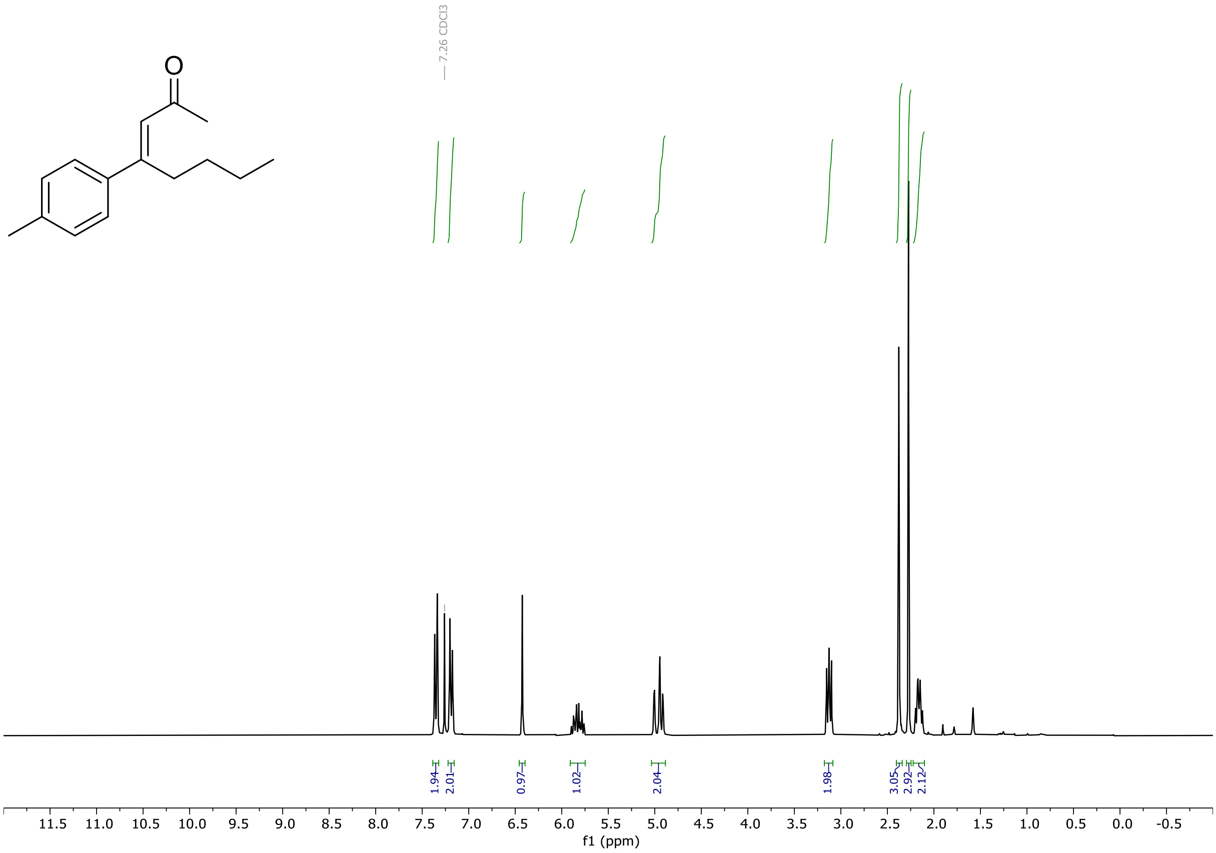

^{13}C-NMR (75 MHz, $CDCl_3$) of (*E*)-4-(*p*-tolyl)octa-3,7-dien-2-one (**1f**)

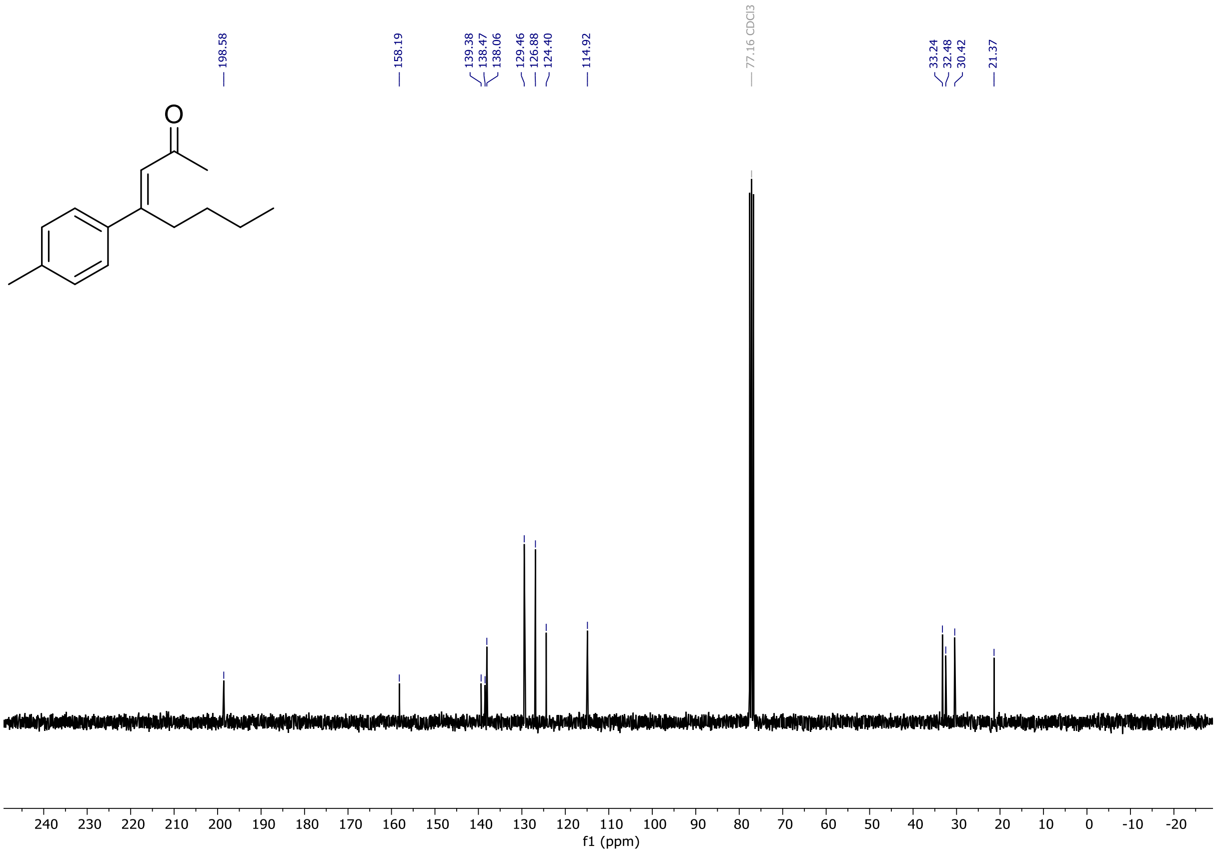

^{1}H-NMR (300 MHz, $CDCl_3$) of (*Z*)-4-(*p*-tolyl)octa-3,7-dien-2-one (**1f**)

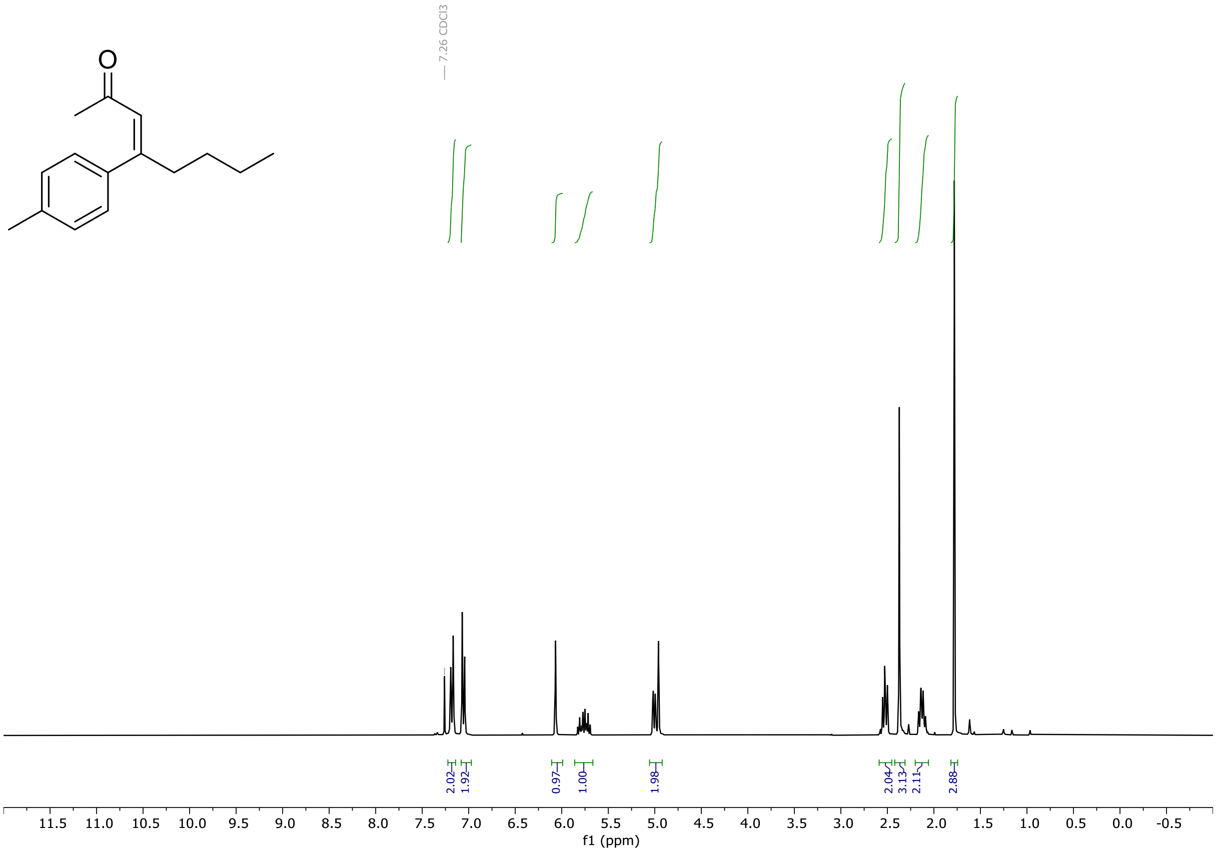

^{13}C-NMR (75 MHz, $CDCl_3$) of (*Z*)-4-(*p*-tolyl)octa-3,7-dien-2-one (**1f**)

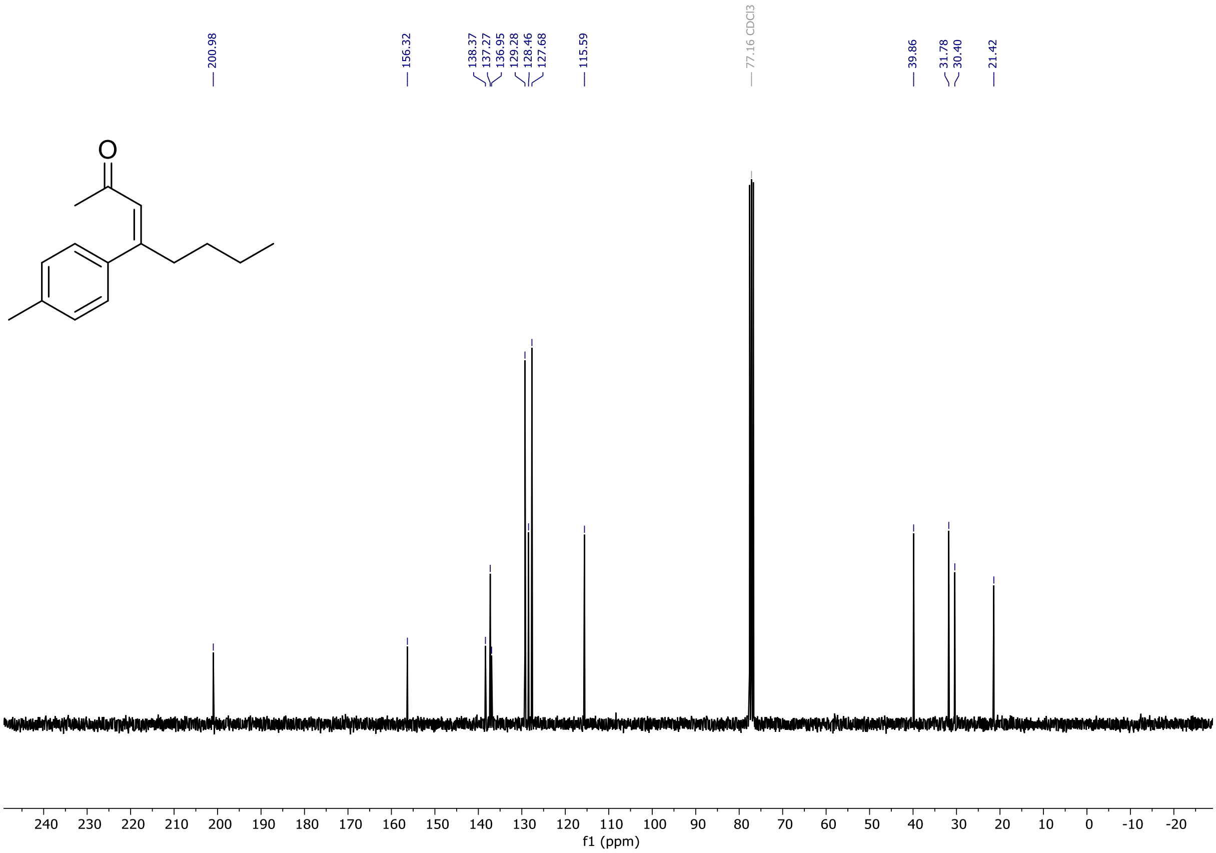

^{1}H-NMR (300 MHz, $CDCl_3$) of (*E*)- 4-(m-tolyl)octa-3,7-dien-2-one (**1g**)

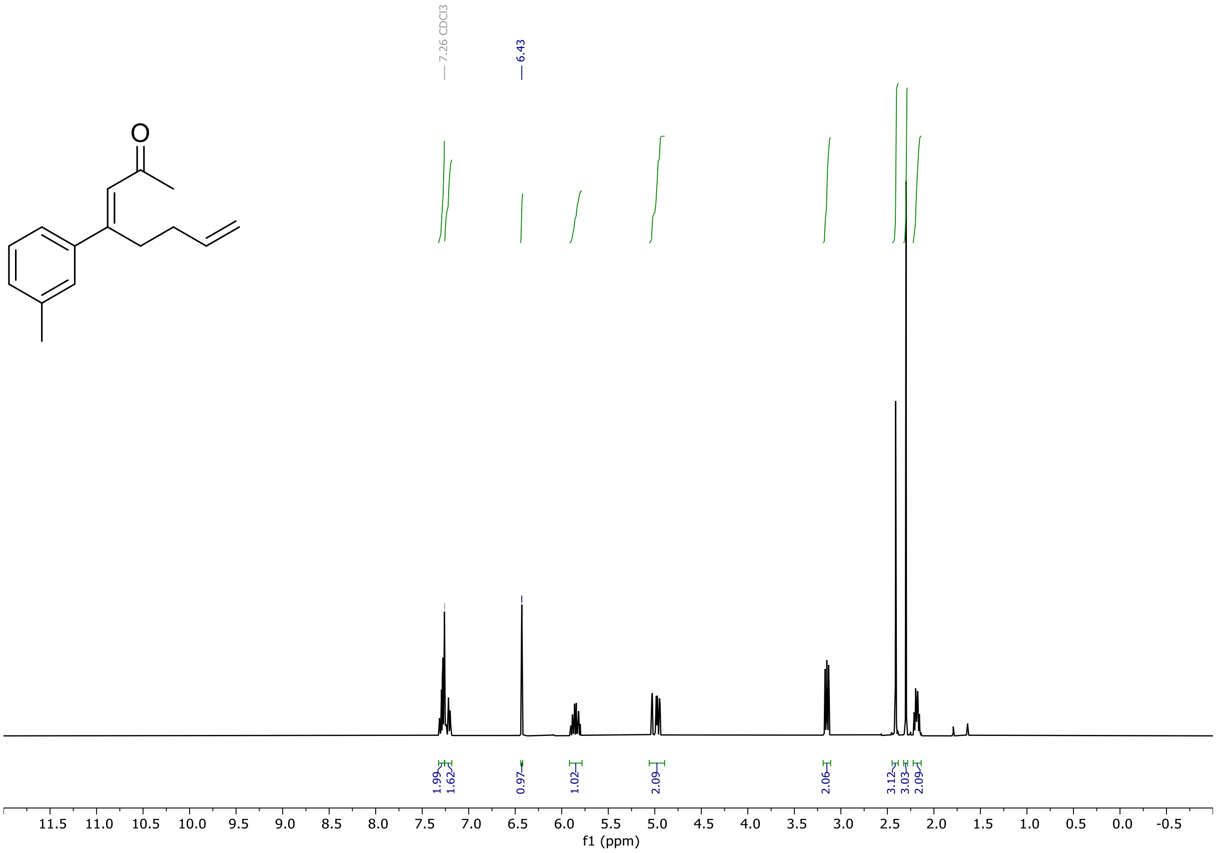

^{13}C-NMR (75 MHz, $CDCl_3$) of (*E*)- 4-(m-tolyl)octa-3,7-dien-2-one (**1g**)

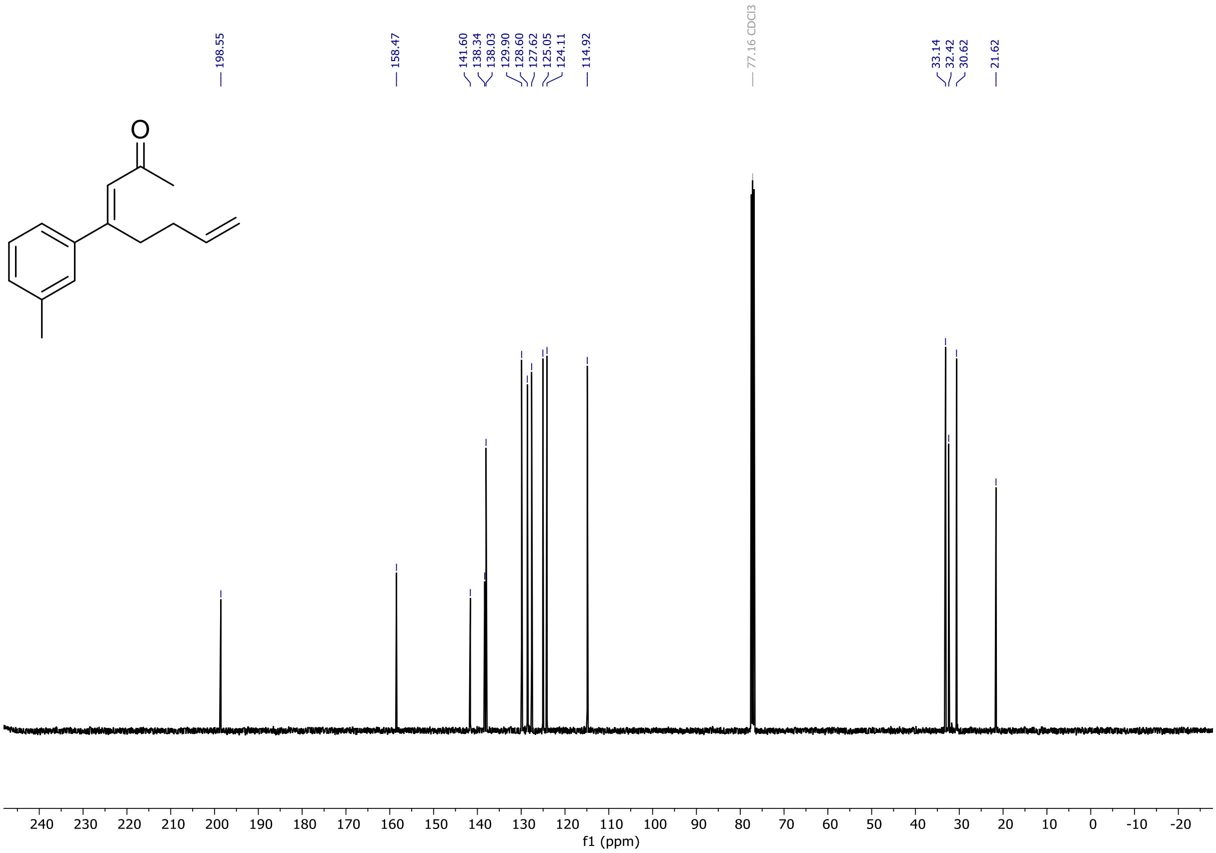

^{1}H-NMR (300 MHz, $CDCl_3$) of (*Z*)- 4-(*m*-tolyl)octa-3,7-dien-2-one (**1g**)

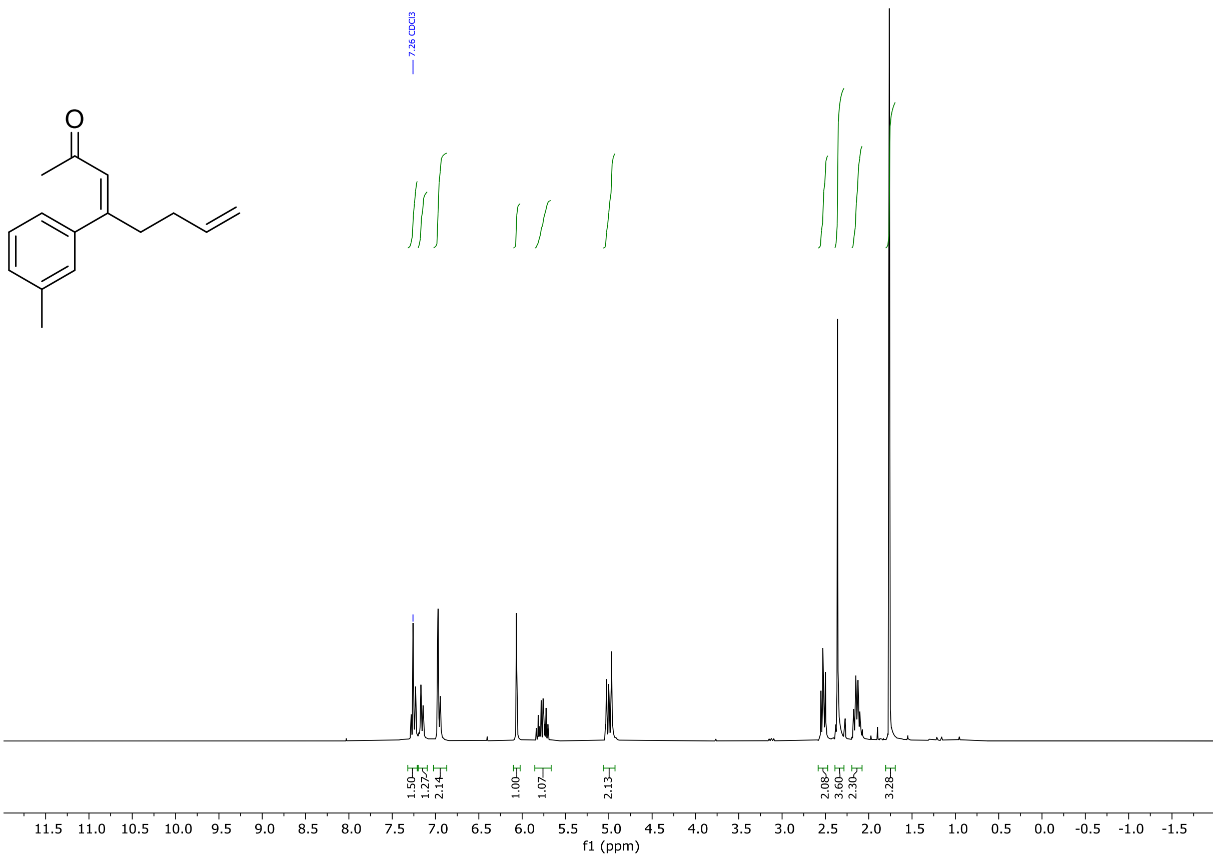

^{13}C-NMR (75 MHz, $CDCl_3$) of (*Z*)- 4-(*m*-tolyl)octa-3,7-dien-2-one (**1g**)

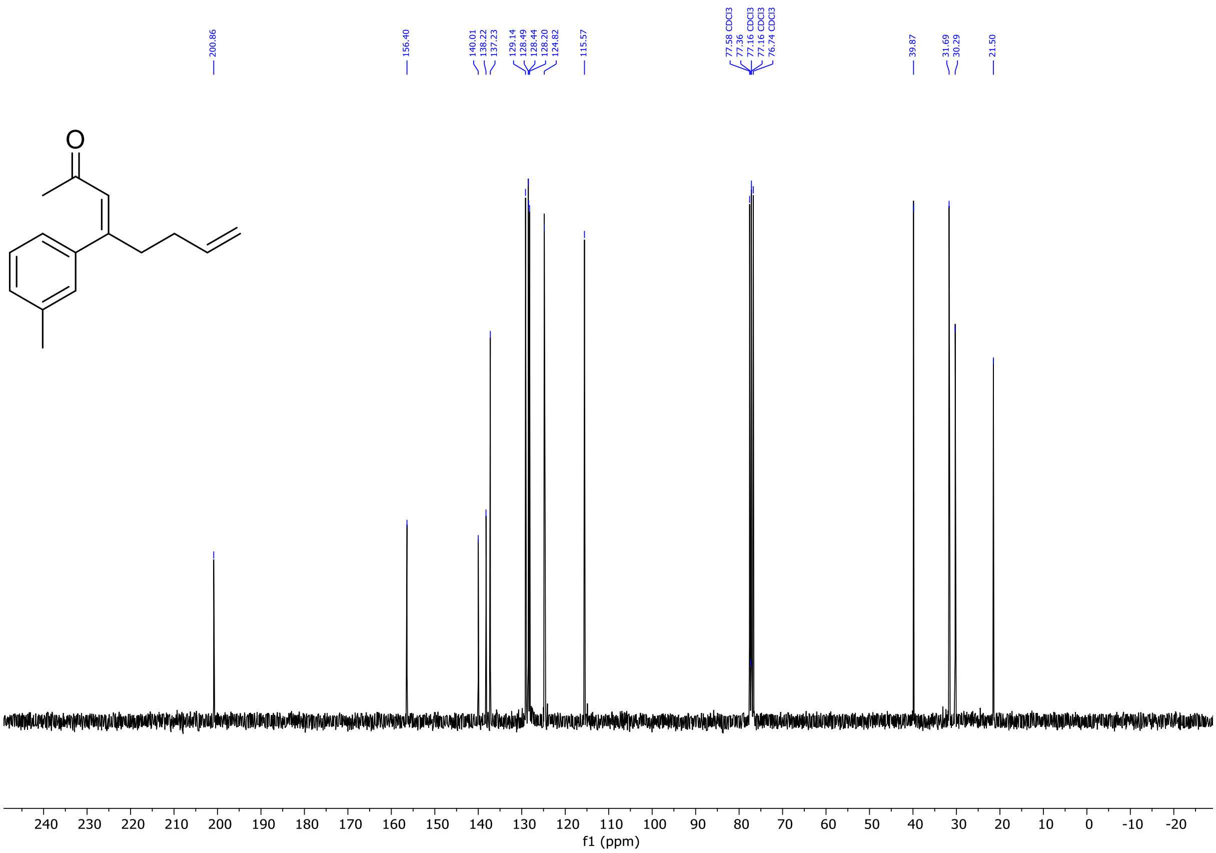

^{1}H-NMR (300 MHz, $CDCl_3$) of (*E*)-4-(naphthalen-2-yl)octa-3,7-dien-2-one (**1h**)

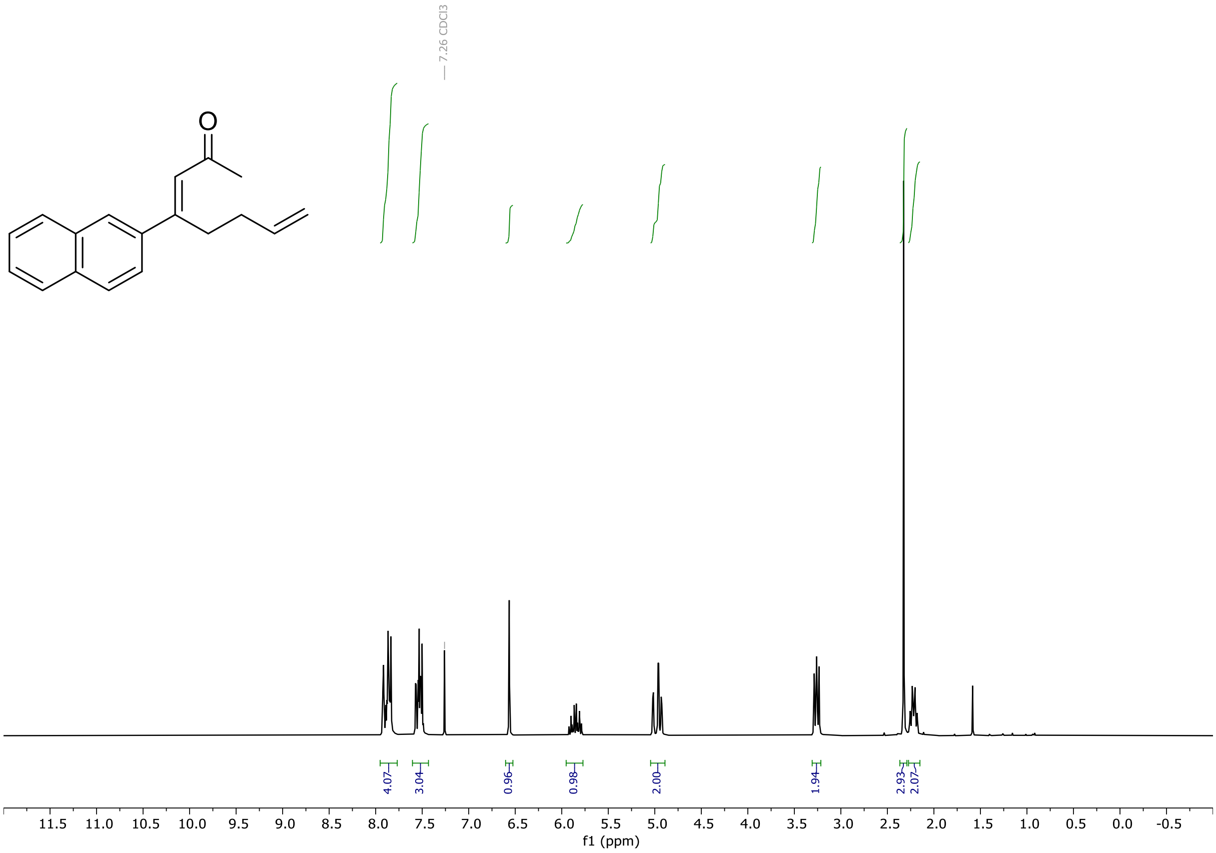

^{13}C-NMR (75 MHz, $CDCl_3$) of (*E*)-4-(naphthalen-2-yl)octa-3,7-dien-2-one (**1h**)

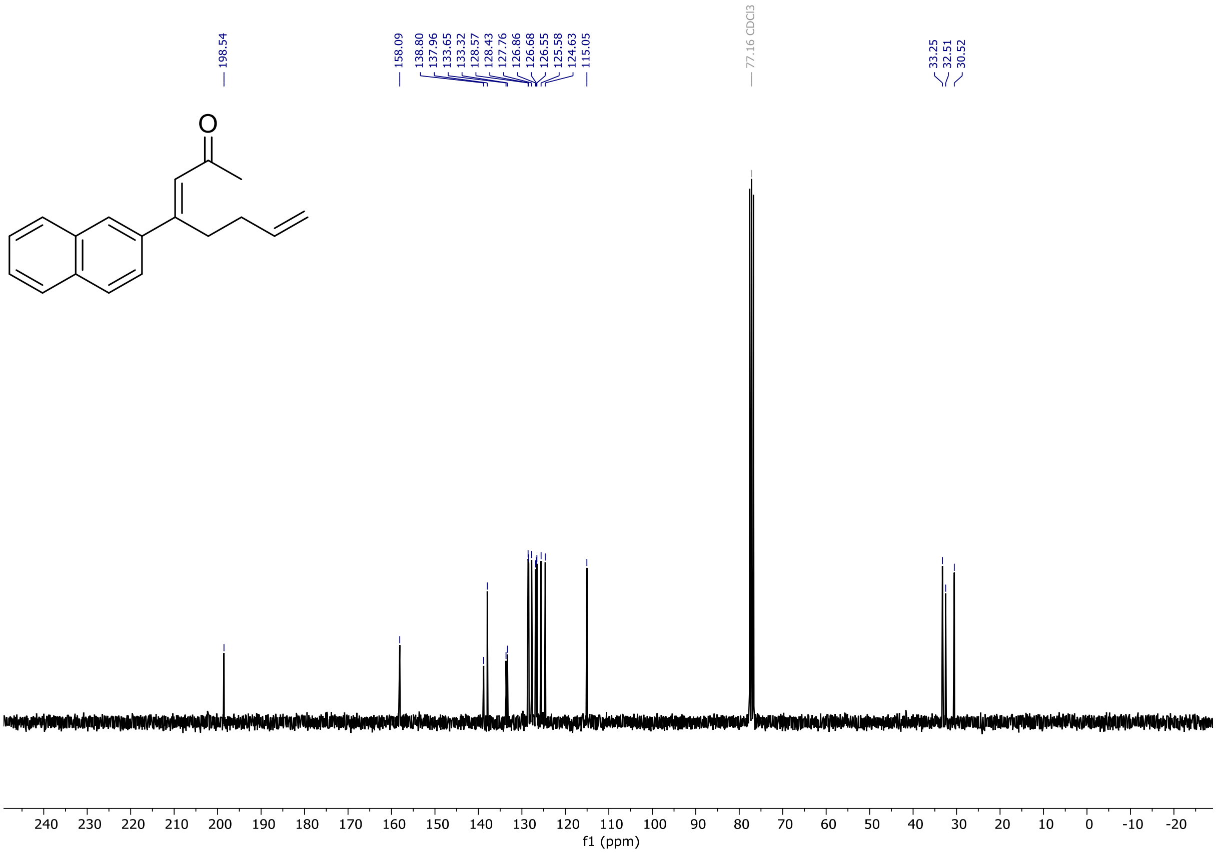

^{1}H-NMR (300 MHz, $CDCl_3$) of (*Z*)-4-(naphthalen-2-yl)octa-3,7-dien-2-one (**1h**)

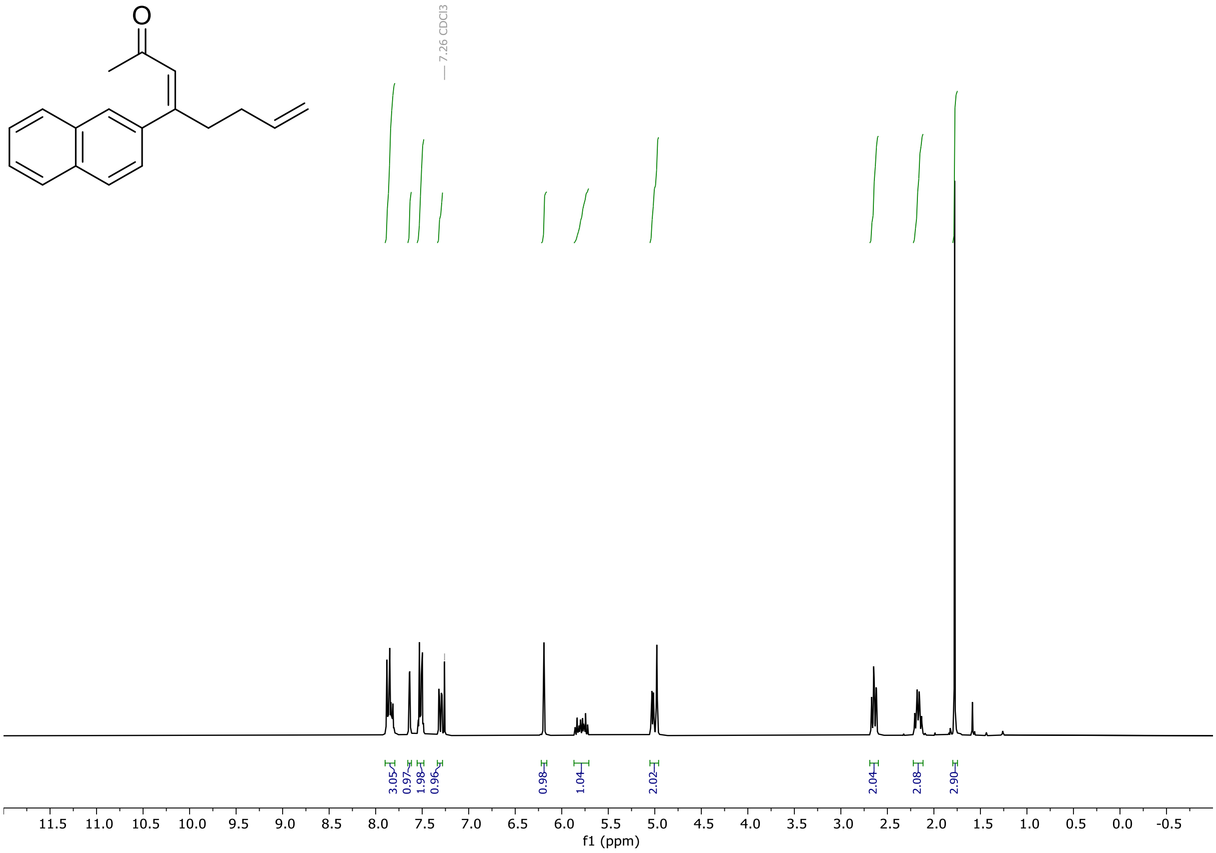

^{13}C-NMR (75 MHz, $CDCl_3$) of (*Z*)-4-(naphthalen-2-yl)octa-3,7-dien-2-one (**1h**)

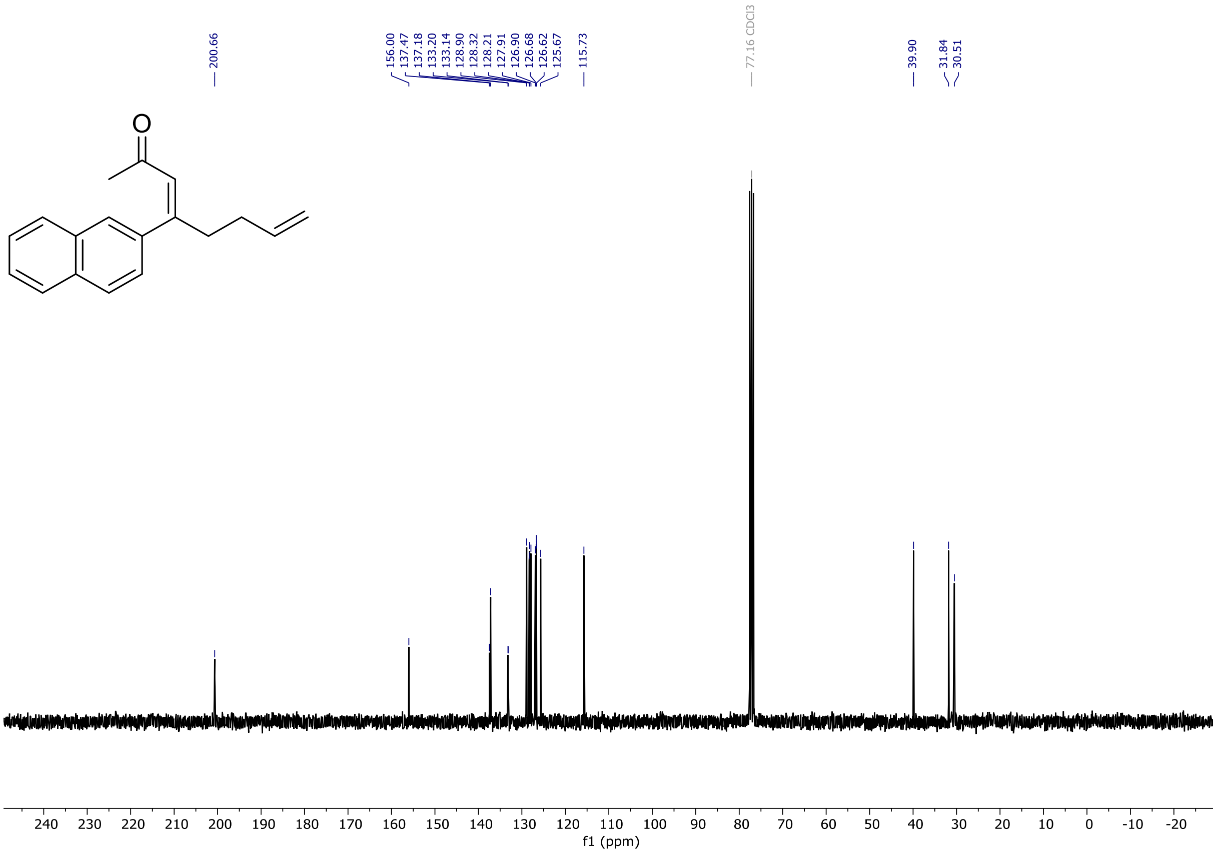

^{1}H-NMR (300 MHz, $CDCl_3$) of 1-Cyclohexyl-3-phenylhepta-2,6-dien-1-one (**1i**)

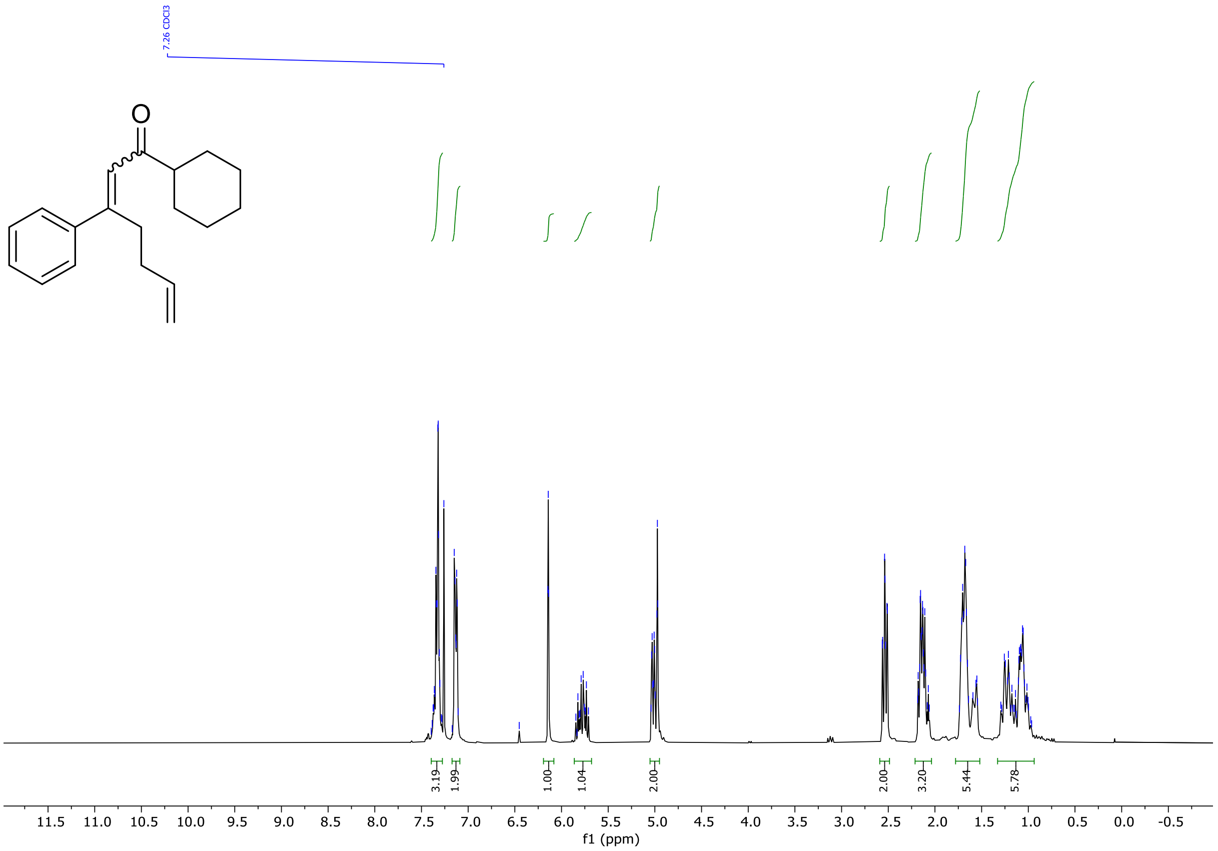

^{13}C-NMR (75 MHz, $CDCl_3$) of 1-Cyclohexyl-3-phenylhepta-2,6-dien-1-one (**1i**)

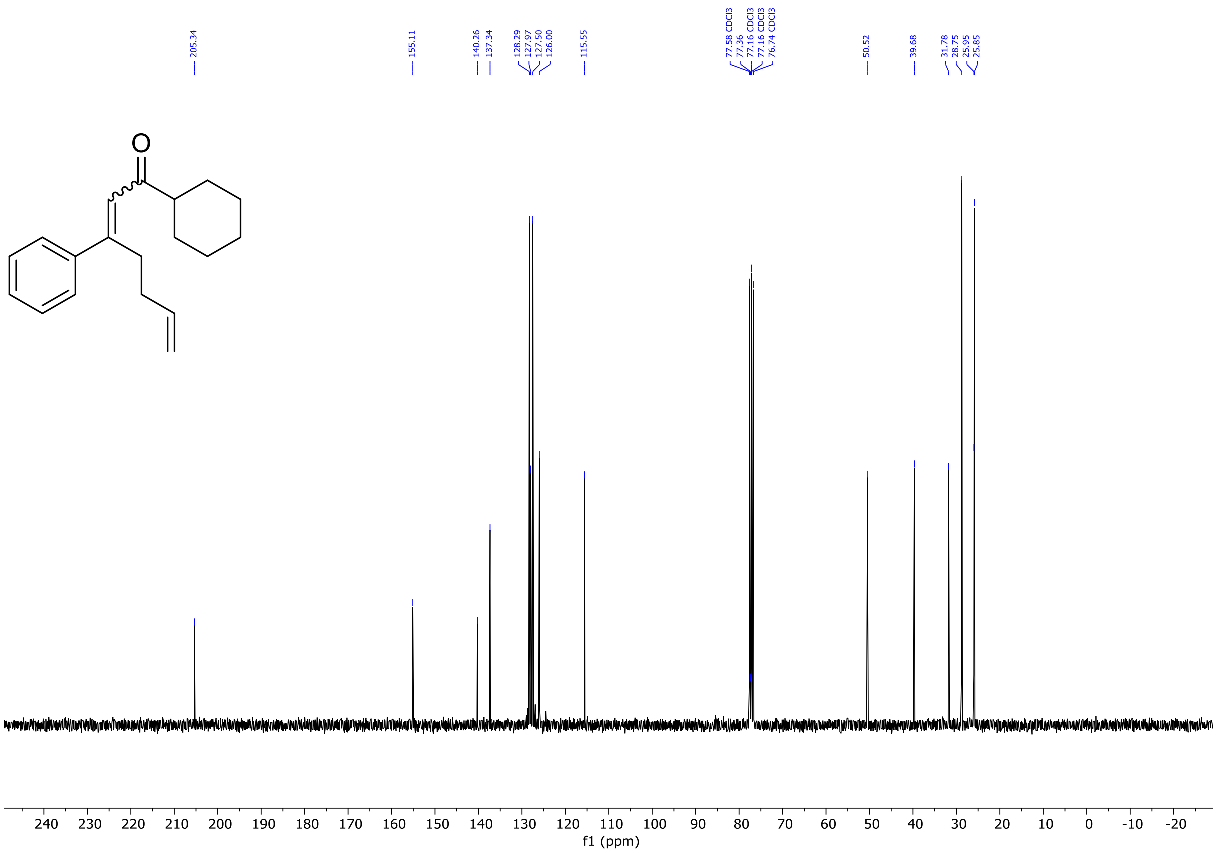

^{1}H-NMR (300 MHz, $CDCl_3$) of 1,3-diphenylhepta-2,6-dien-1-one (**1j**)

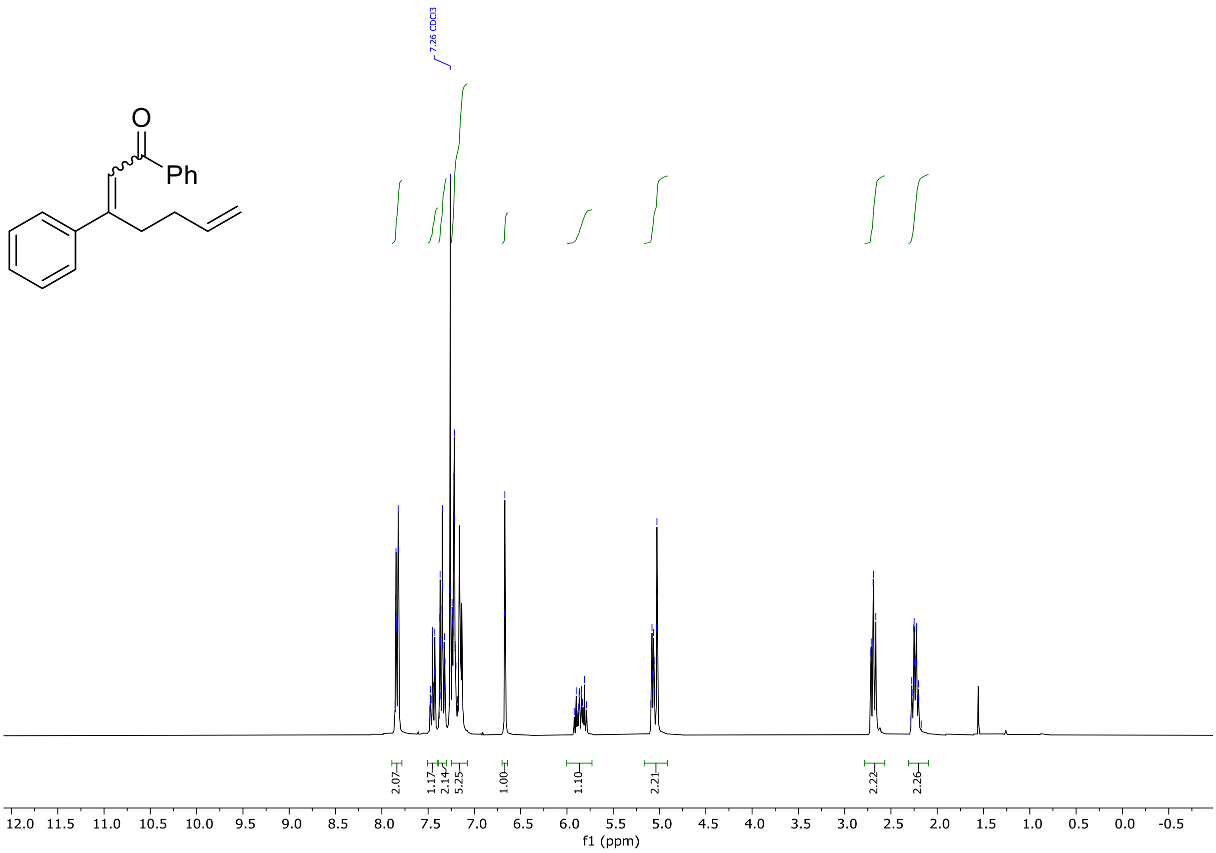

^{13}C-NMR (75 MHz, $CDCl_3$) of 1,3-diphenylhepta-2,6-dien-1-one (**1j**)

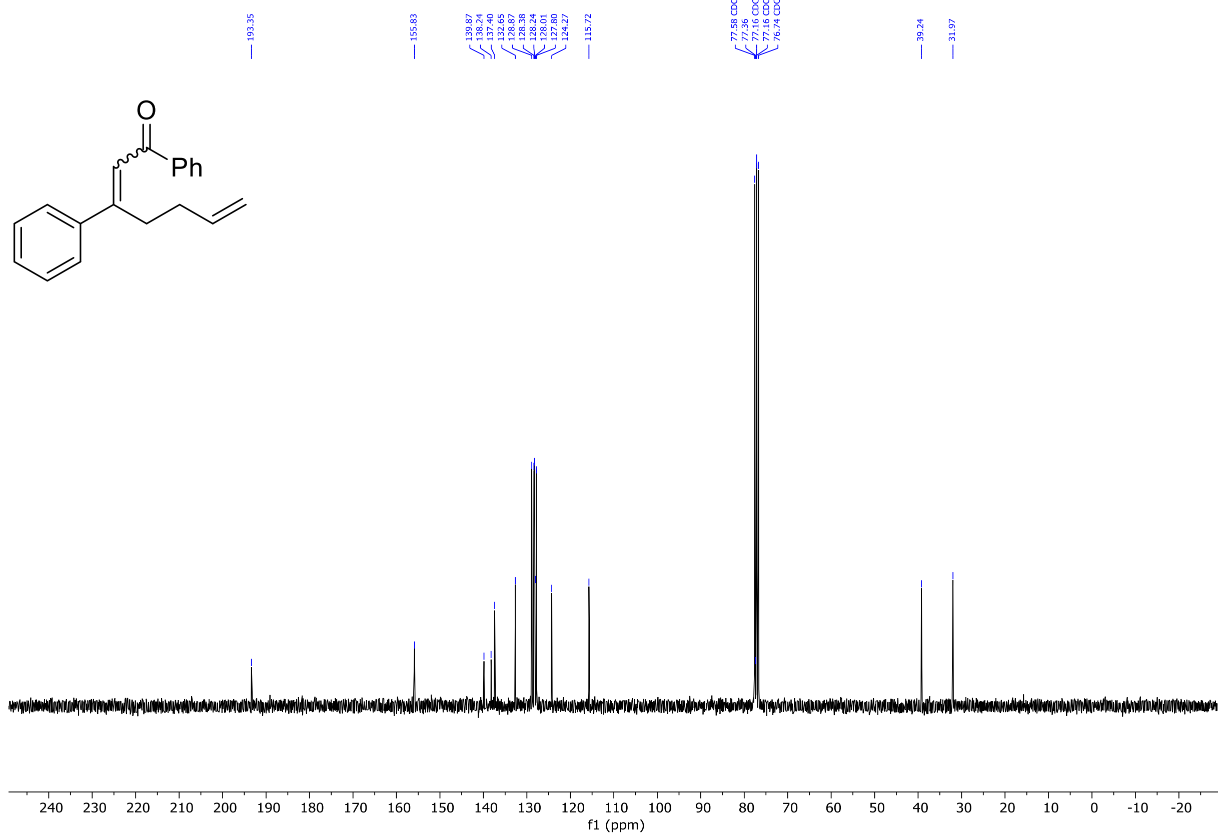

^{1}H-NMR (600 MHz, $CDCl_3$) of (*E*)-4-(2-oxoocta-3,7-dien-4-yl)benzonitrile (**1k**)

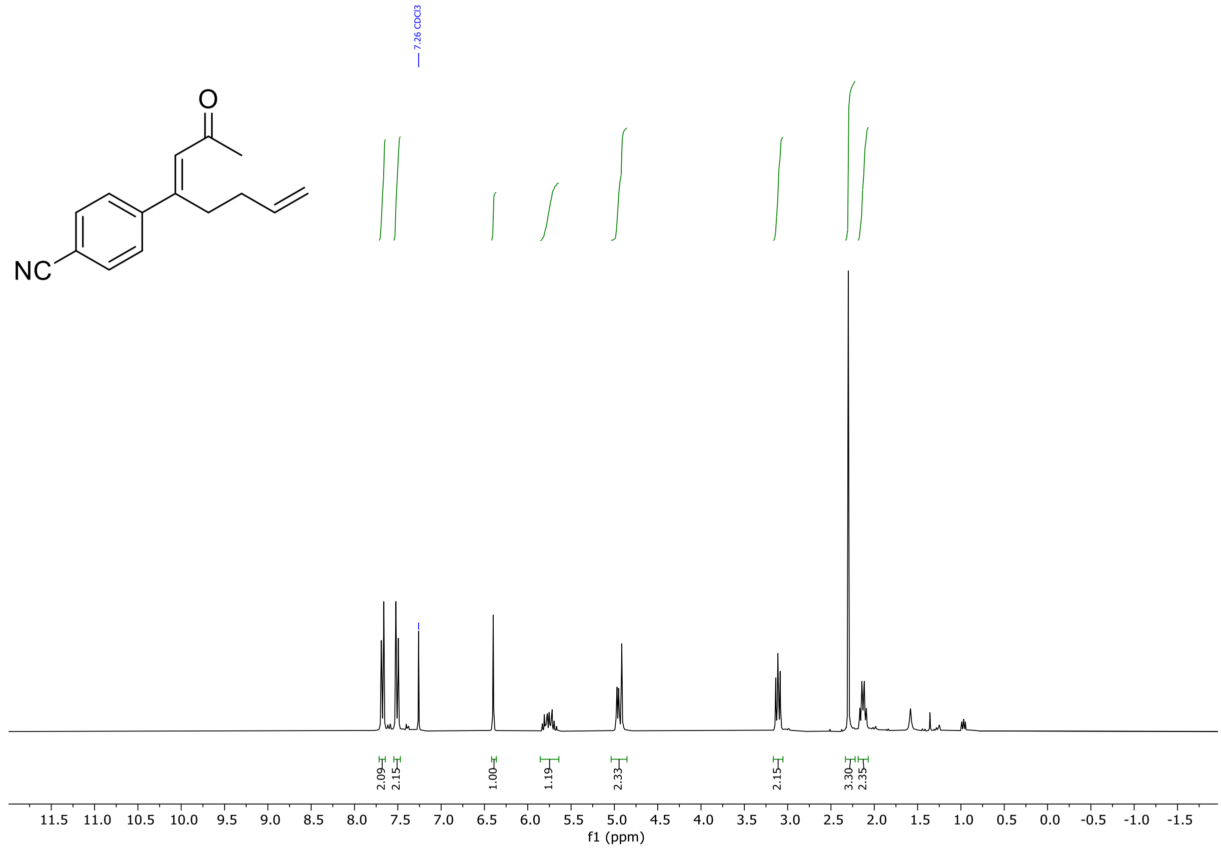

^{13}C-NMR (151 MHz, $CDCl_3$) of (*E*)-4-(2-oxoocta-3,7-dien-4-yl)benzonitrile (**1k**)

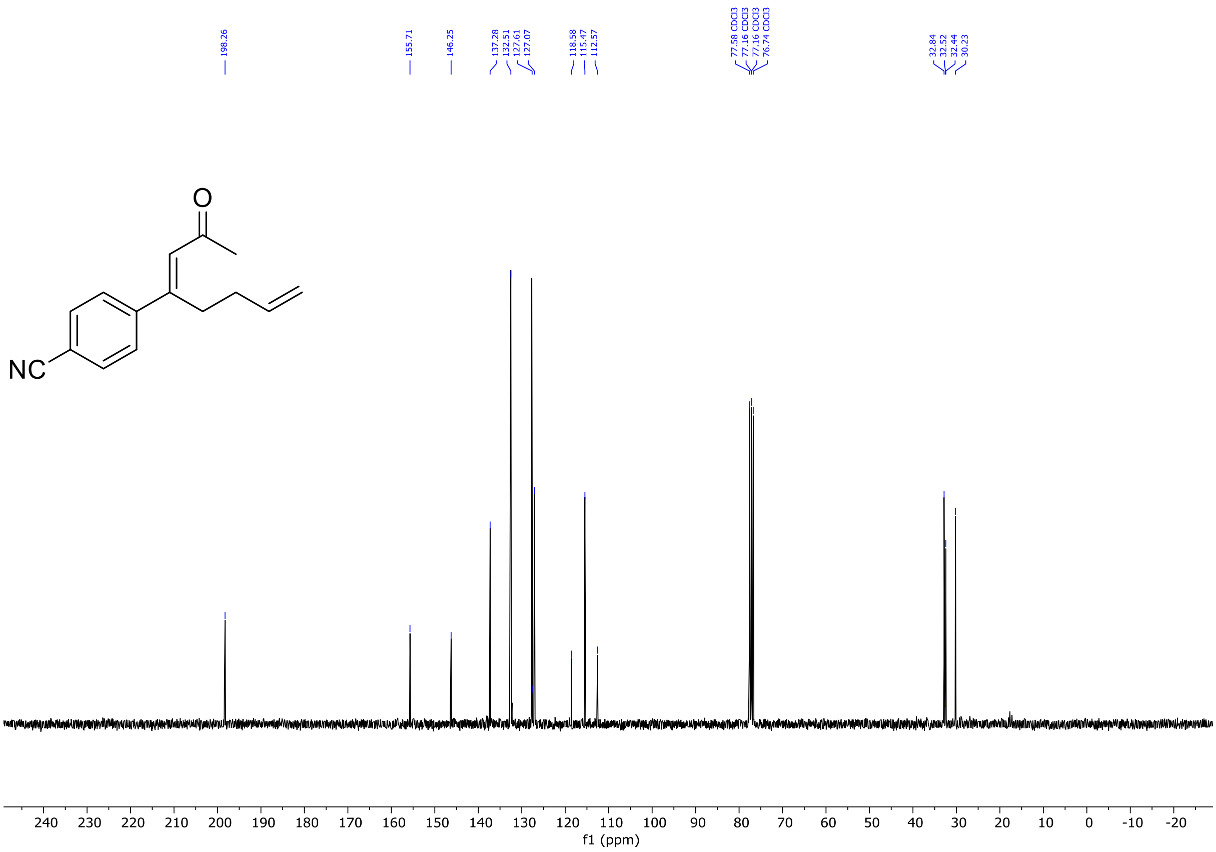

^{1}H-NMR (600 MHz, $CDCl_3$) of (*Z*)-4-(2-oxoocta-3,7-dien-4-yl)benzonitrile (**1k**)

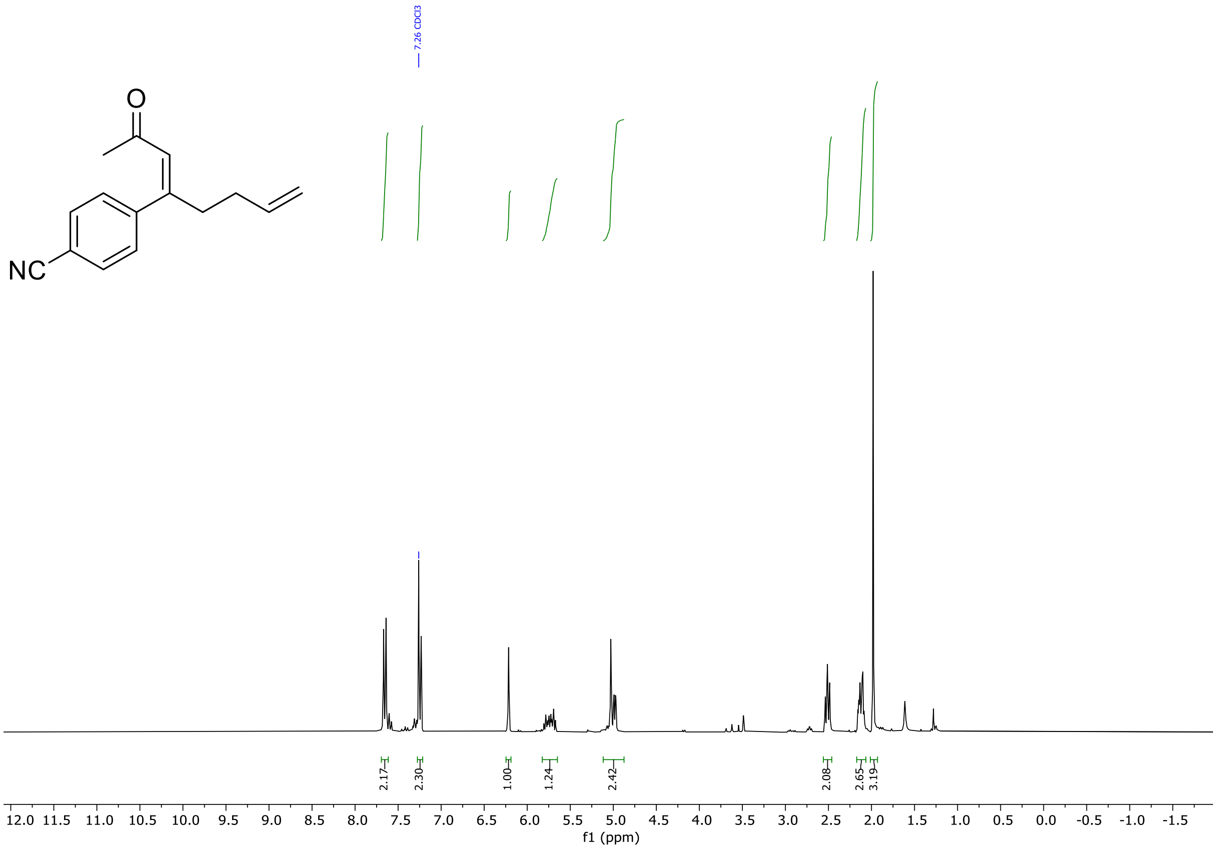

^{13}C-NMR (151 MHz, $CDCl_3$) of (*Z*)-4-(2-oxoocta-3,7-dien-4-yl)benzonitrile (**1k**)

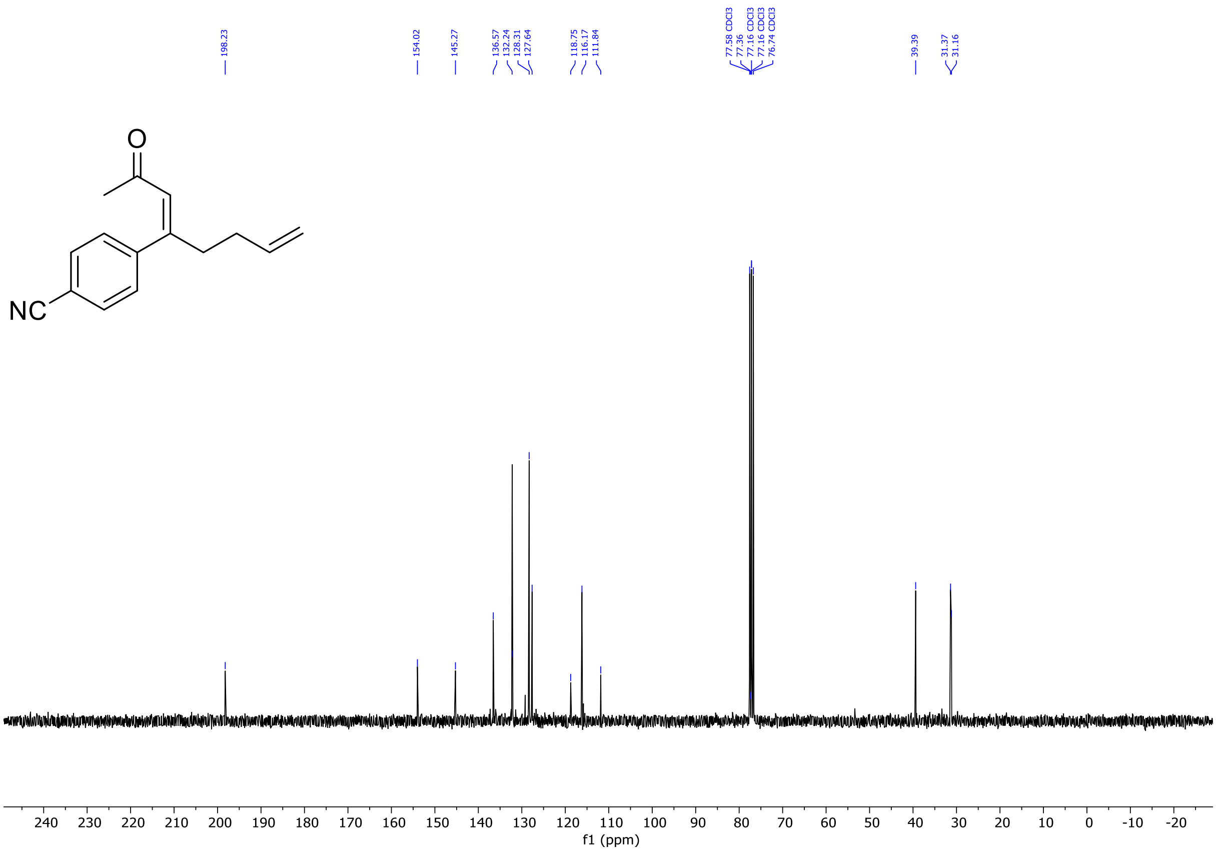

^{1}H-NMR (600 MHz, $CDCl_3$) of 1-(1-Phenylbicyclo[2.1.1]hexan-5-yl)ethan-1-one (**2a**)

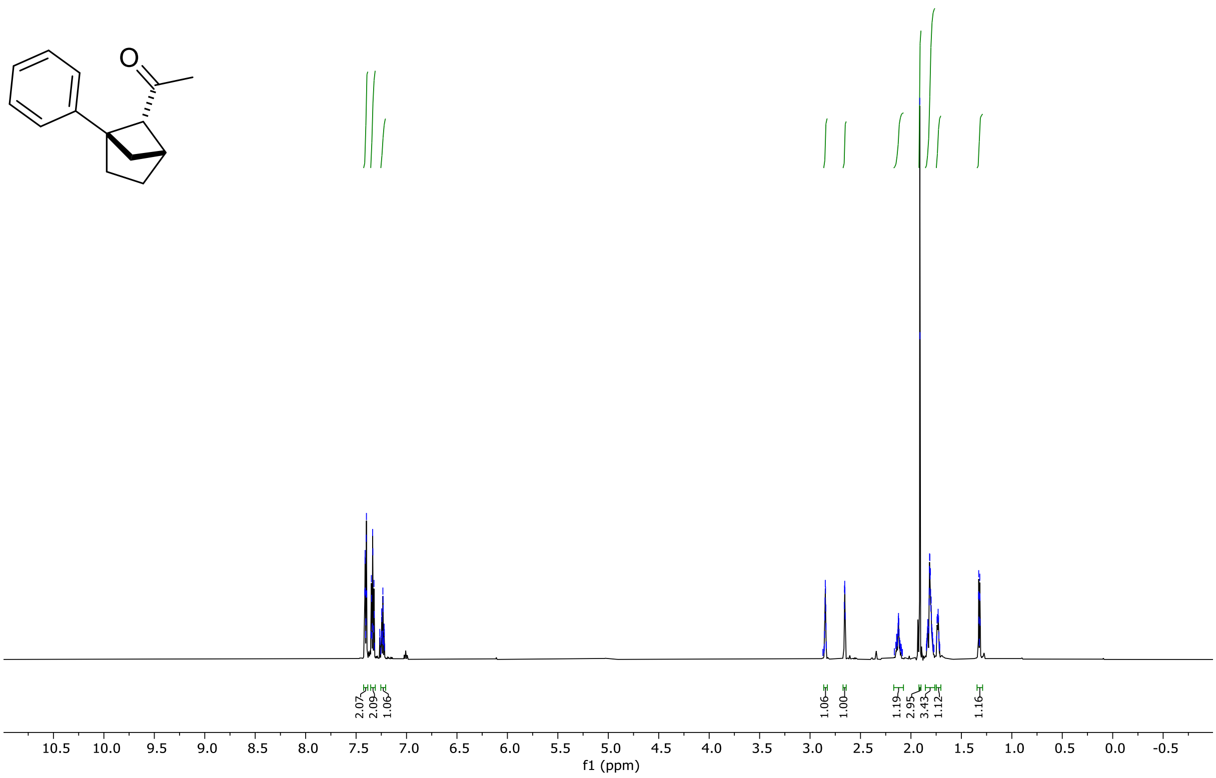

^{13}C-NMR (151 MHz, $CDCl_3$) of 1-(1-Phenylbicyclo[2.1.1]hexan-5-yl)ethan-1-one (**2a**)

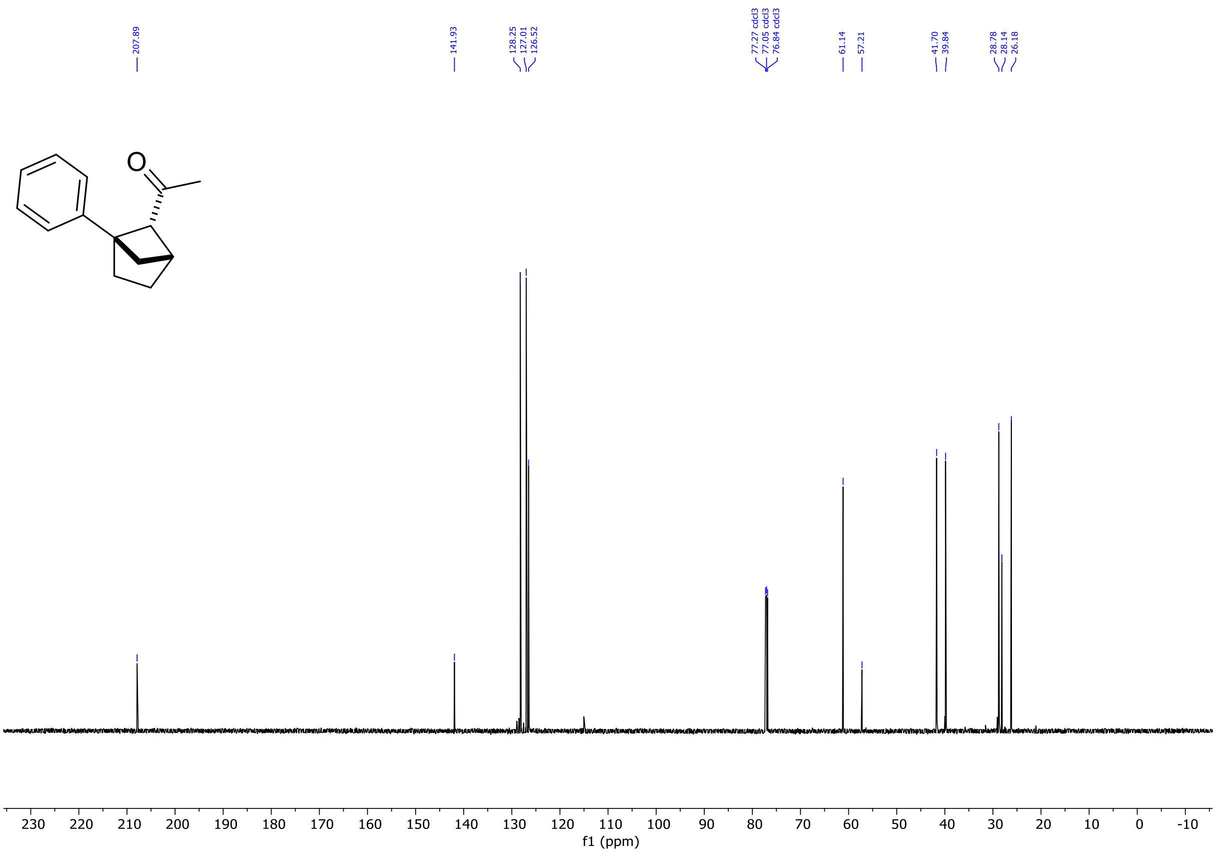

^{1}H-NMR (300 MHz, $CDCl_3$) of 1-(4-fluorophenyl)bicyclo[2.1.1]hexan-5-yl)ethan-1-one (**2b**)

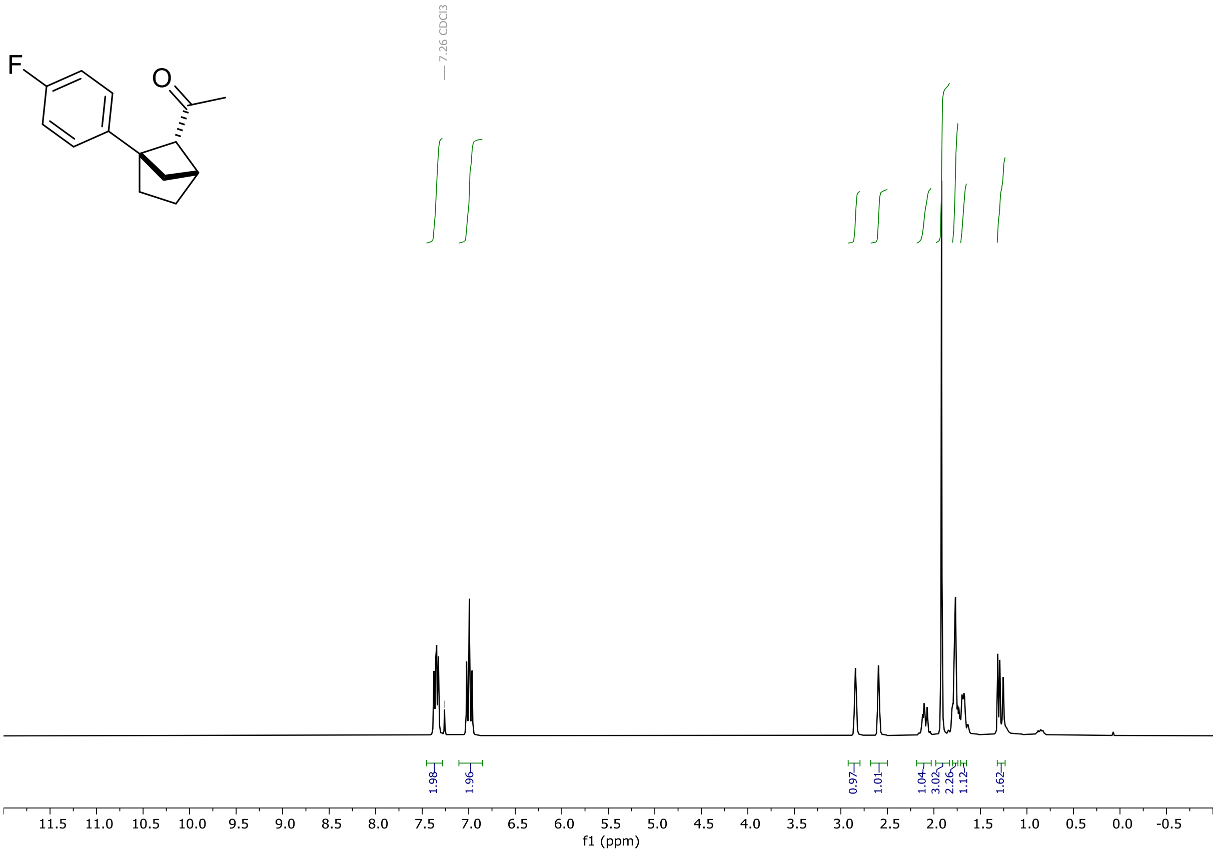

^{13}C-NMR (75 MHz, $CDCl_3$) of 1-(4-fluorophenyl)bicyclo[2.1.1]hexan-5-yl)ethan-1-one (**2b**)

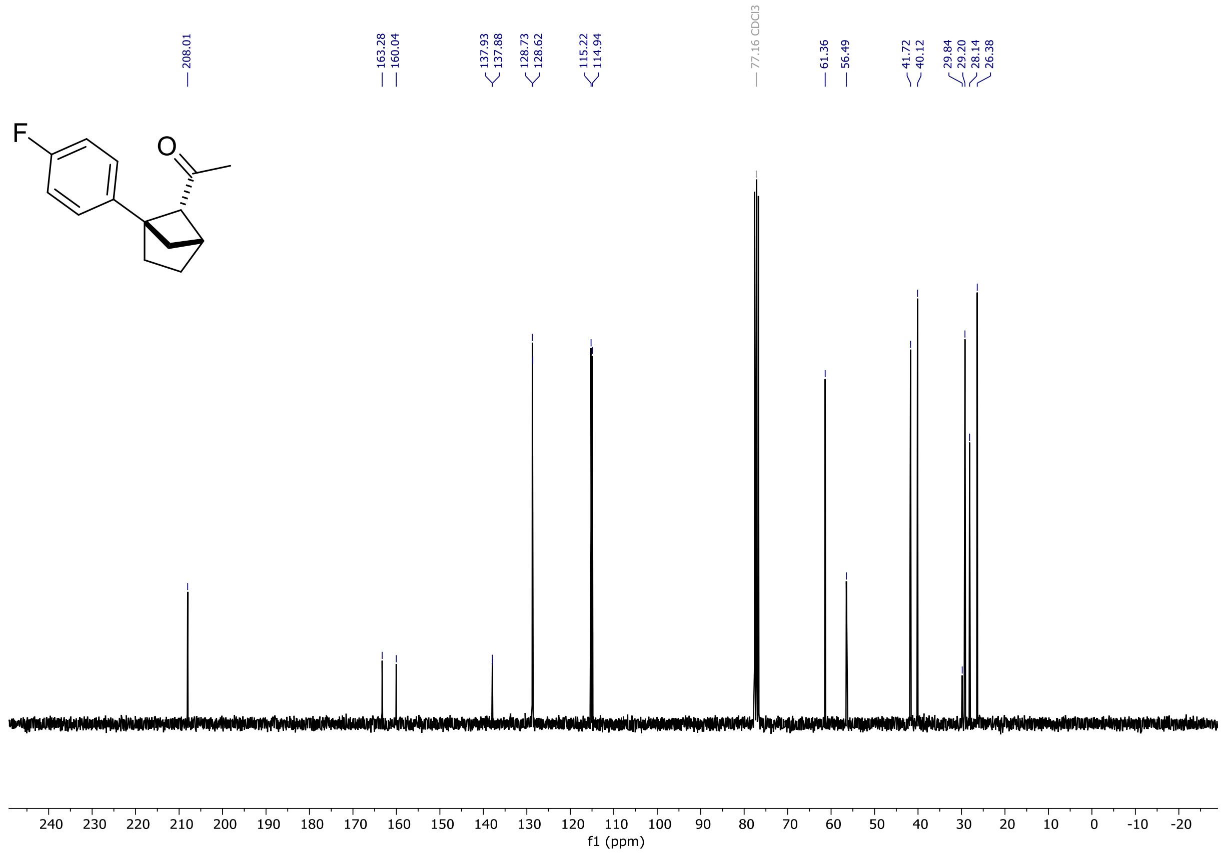

^{19}F-NMR (282 MHz, $CDCl_3$) of 1-(4-fluorophenyl)bicyclo[2.1.1]hexan-5-yl)ethan-1-one (**2b**)

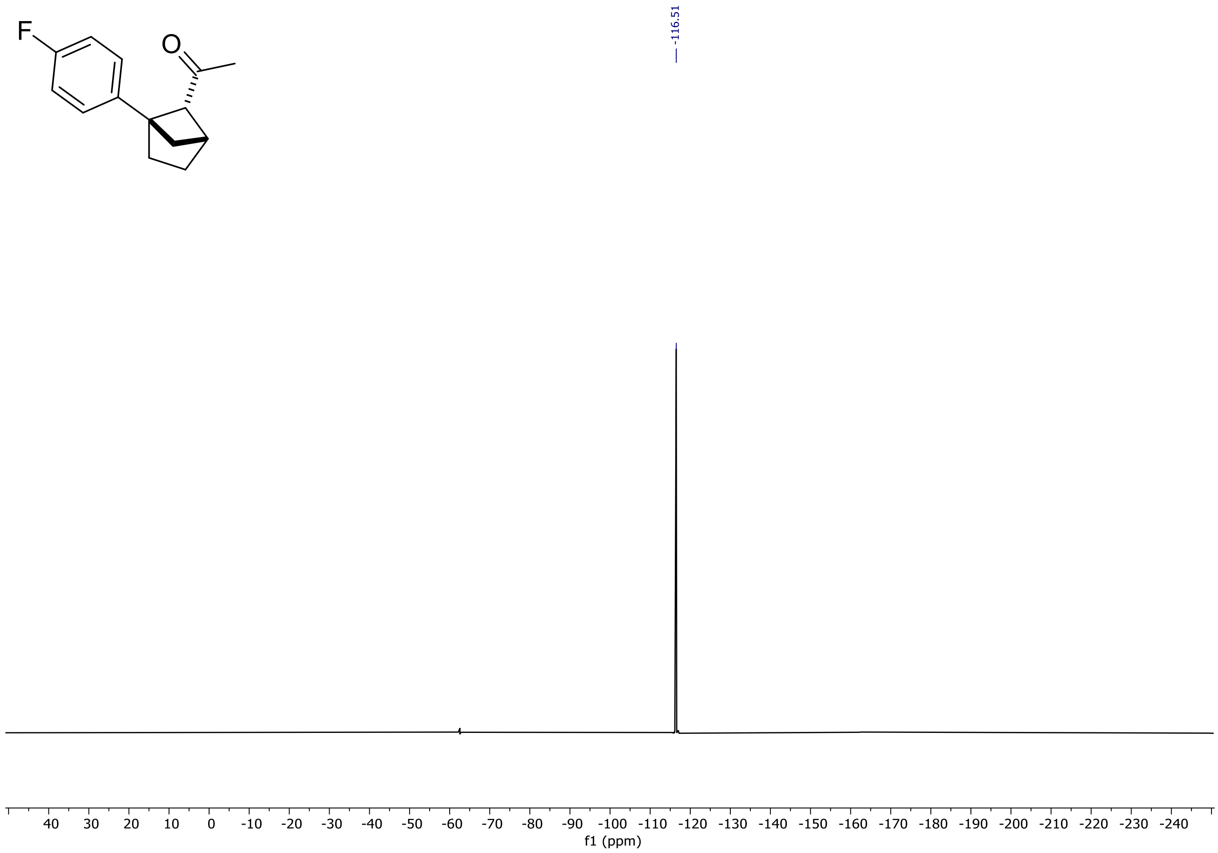

^{1}H-NMR (300 MHz, $CDCl_3$) of 1-(1-(4-chlorophenyl)bicyclo[2.1.1]hexan-5-yl)ethan-1-one (**2c**)

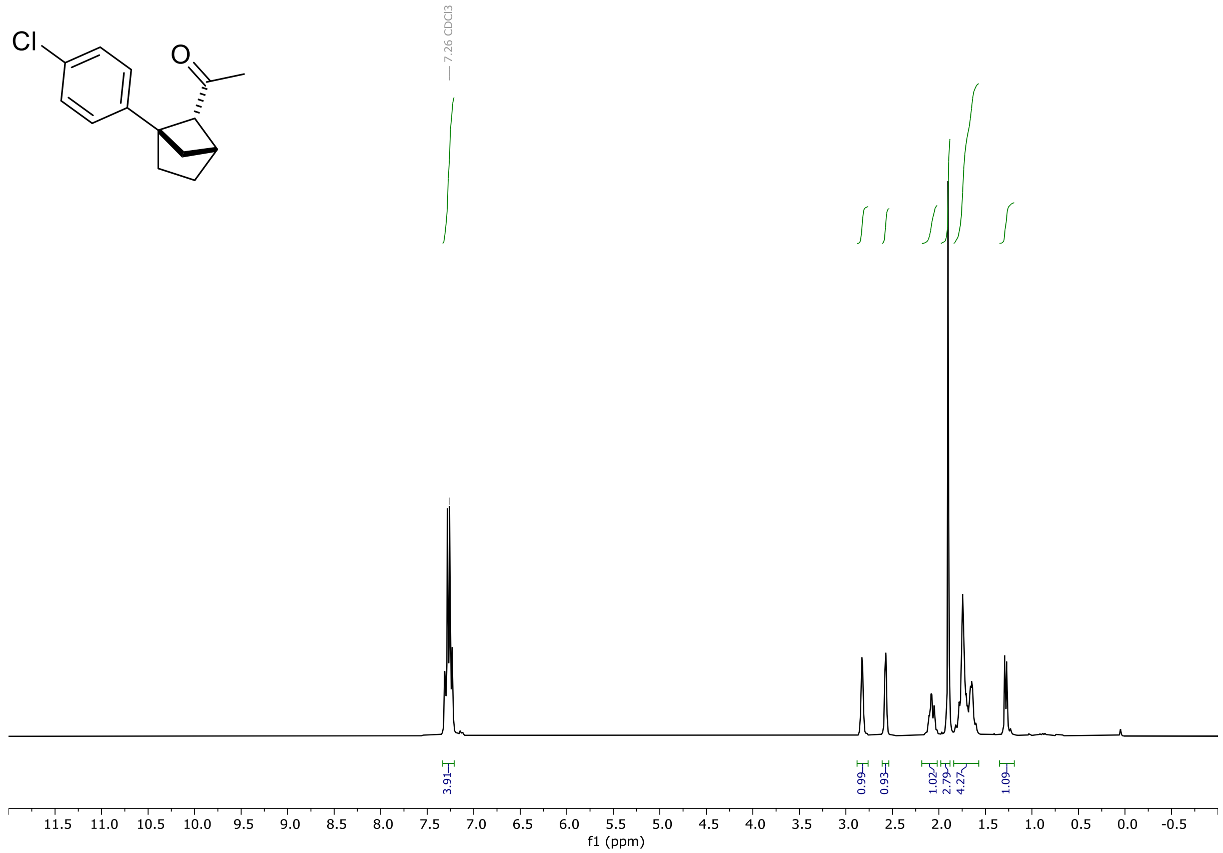

^{13}C-NMR (75 MHz, $CDCl_3$) of 1-(1-(4-chlorophenyl)bicyclo[2.1.1]hexan-5-yl)ethan-1-one (**2c**)

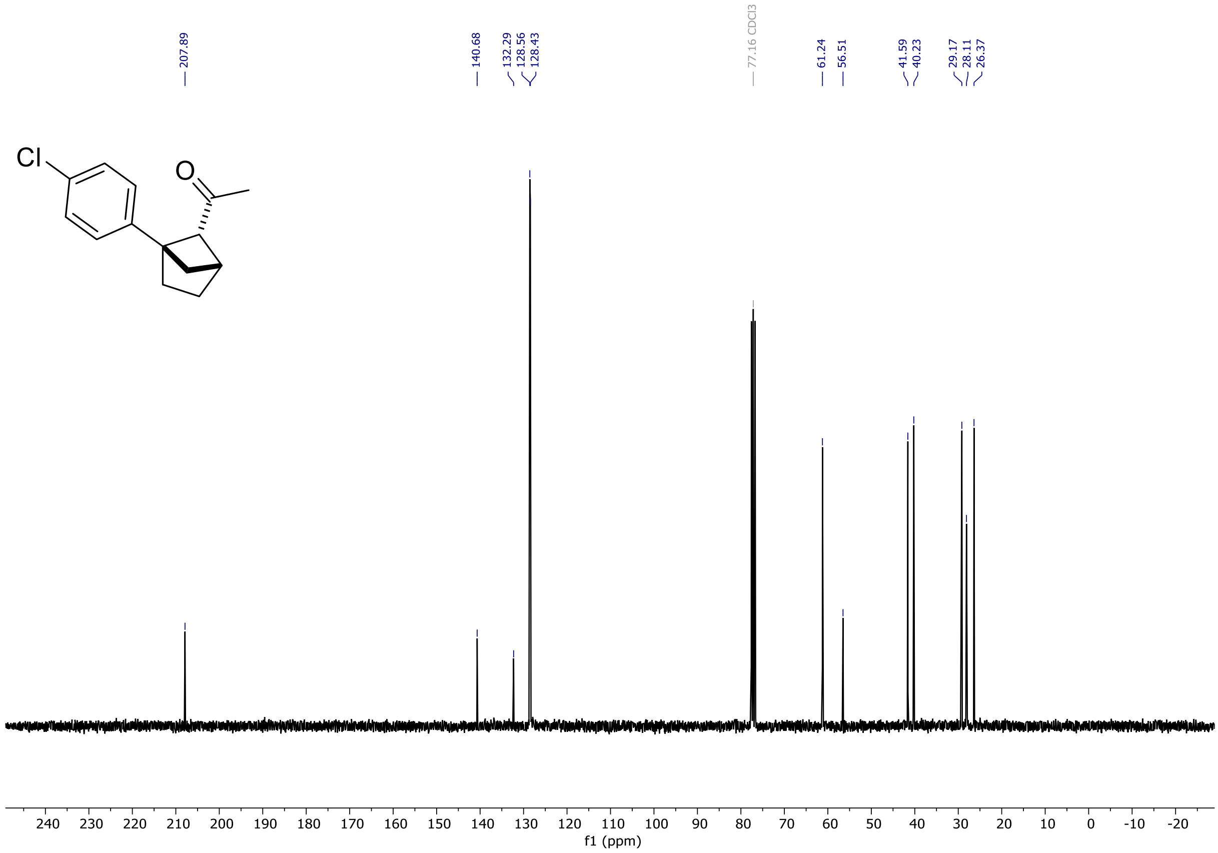

^{1}H-NMR (300 MHz, $CDCl_3$) of 1-(1-(4-bromophenyl)bicyclo[2.1.1]hexan-5-yl)ethan-1-one (**2d**)

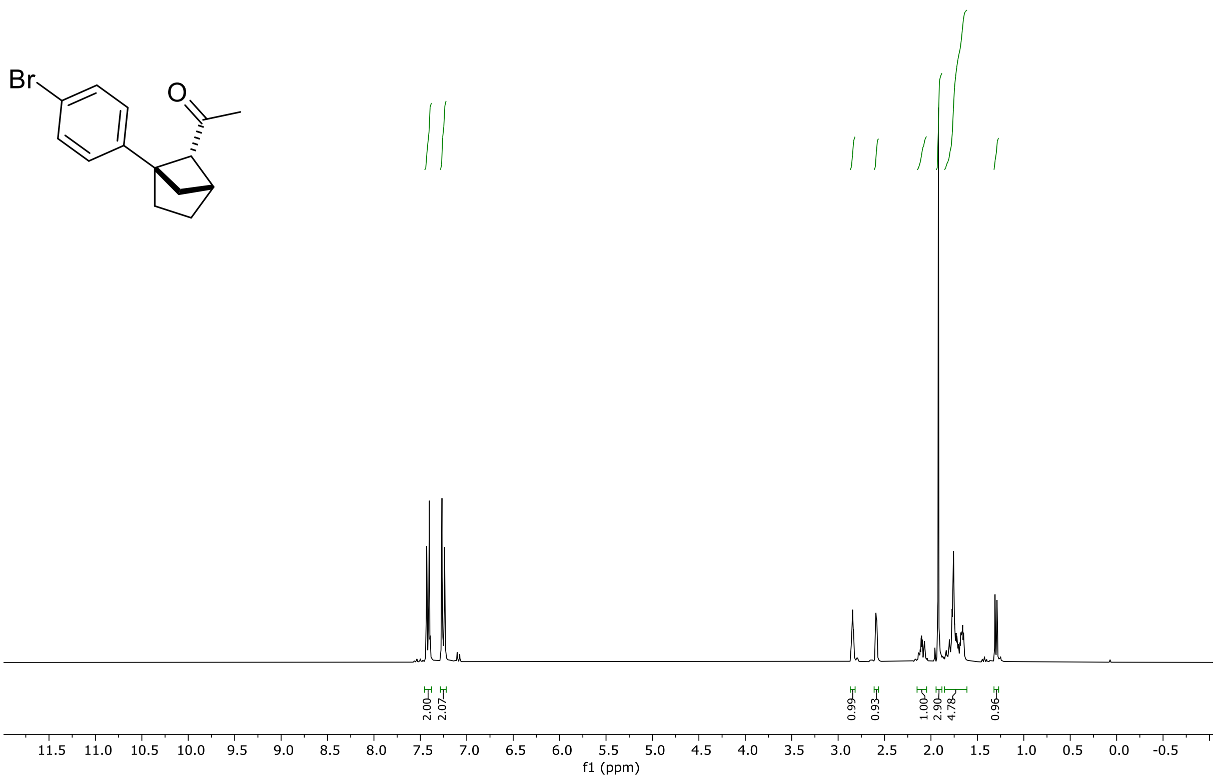

^{13}C-NMR (75 MHz, $CDCl_3$) of 1-(1-(4-bromophenyl)bicyclo[2.1.1]hexan-5-yl)ethan-1-one (**2d**)

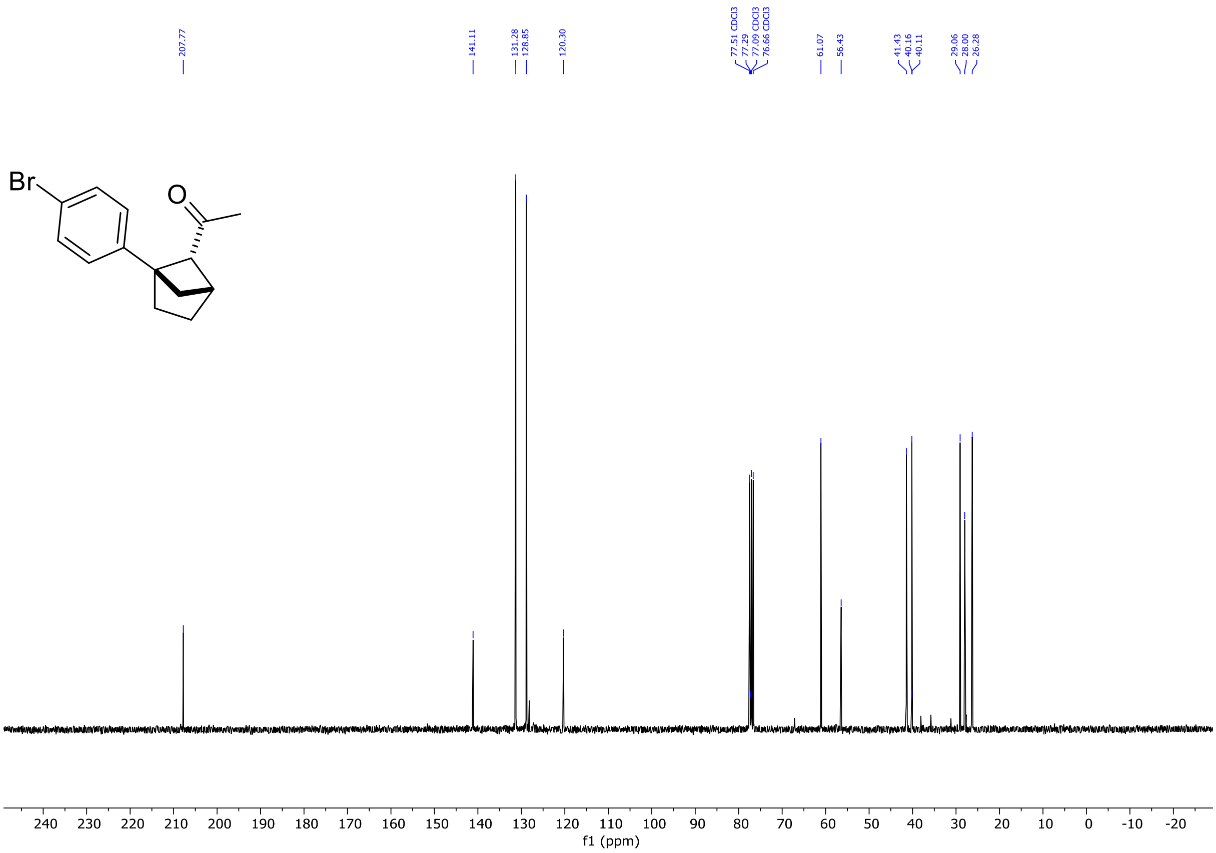

^{1}H-NMR (300 MHz, $CDCl_3$) of 1-(1-(4-(trifluoromethyl)phenyl)bicyclo[2.1.1]hexan-5-yl)ethan-1-one (**2e**)

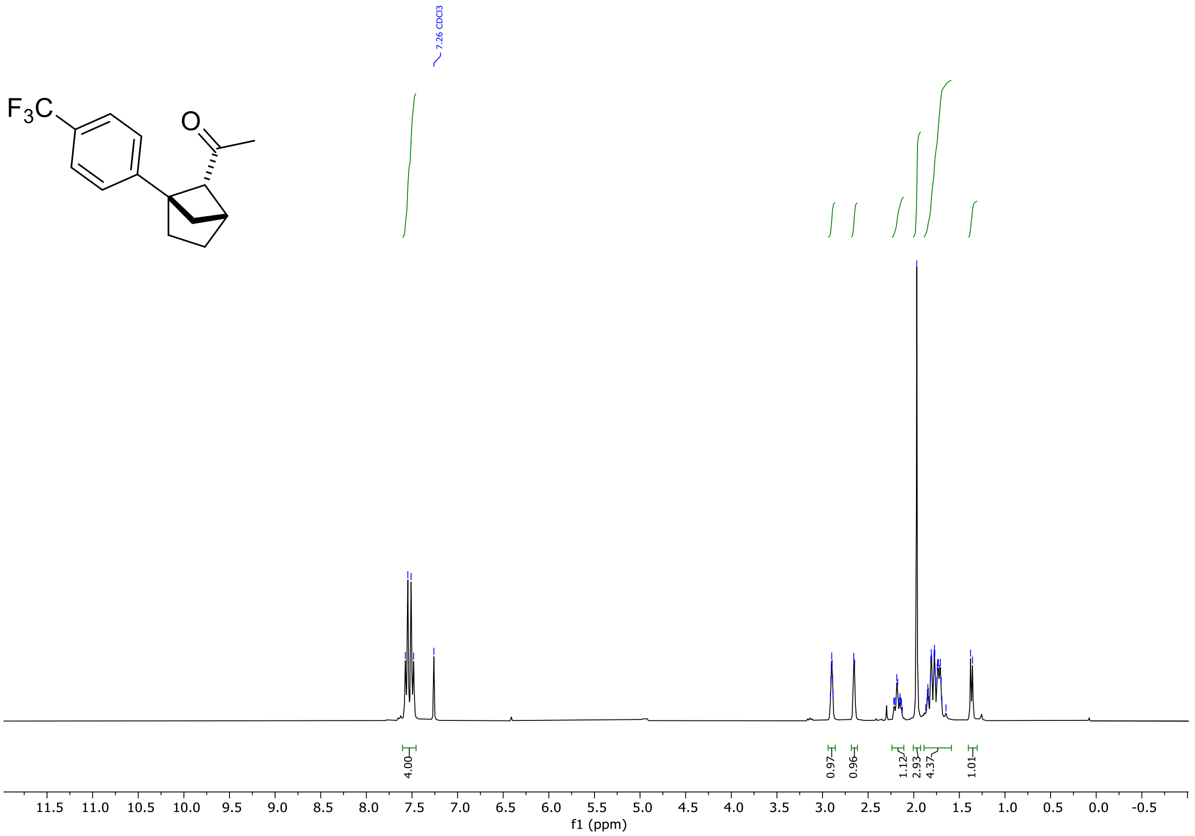

^{13}C-NMR (75 MHz, $CDCl_3$) of 1-(1-(4-(trifluoromethyl)phenyl)bicyclo[2.1.1]hexan-5-yl)ethan-1-one (**2e**)

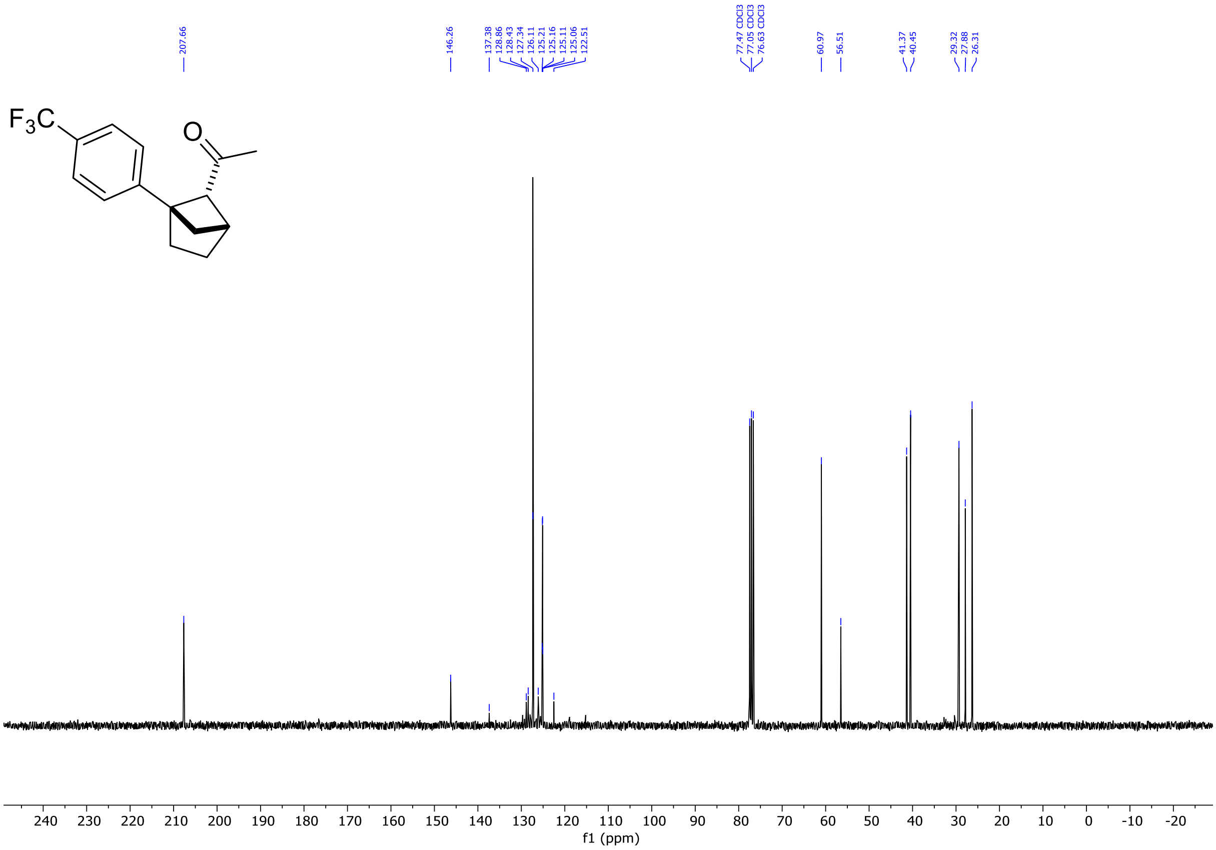

^{19}F-NMR (282 MHz, $CDCl_3$) of 1-(1-(4-(trifluoromethyl)phenyl)bicyclo[2.1.1]hexan-5-yl)ethan-1-one (**2e**)

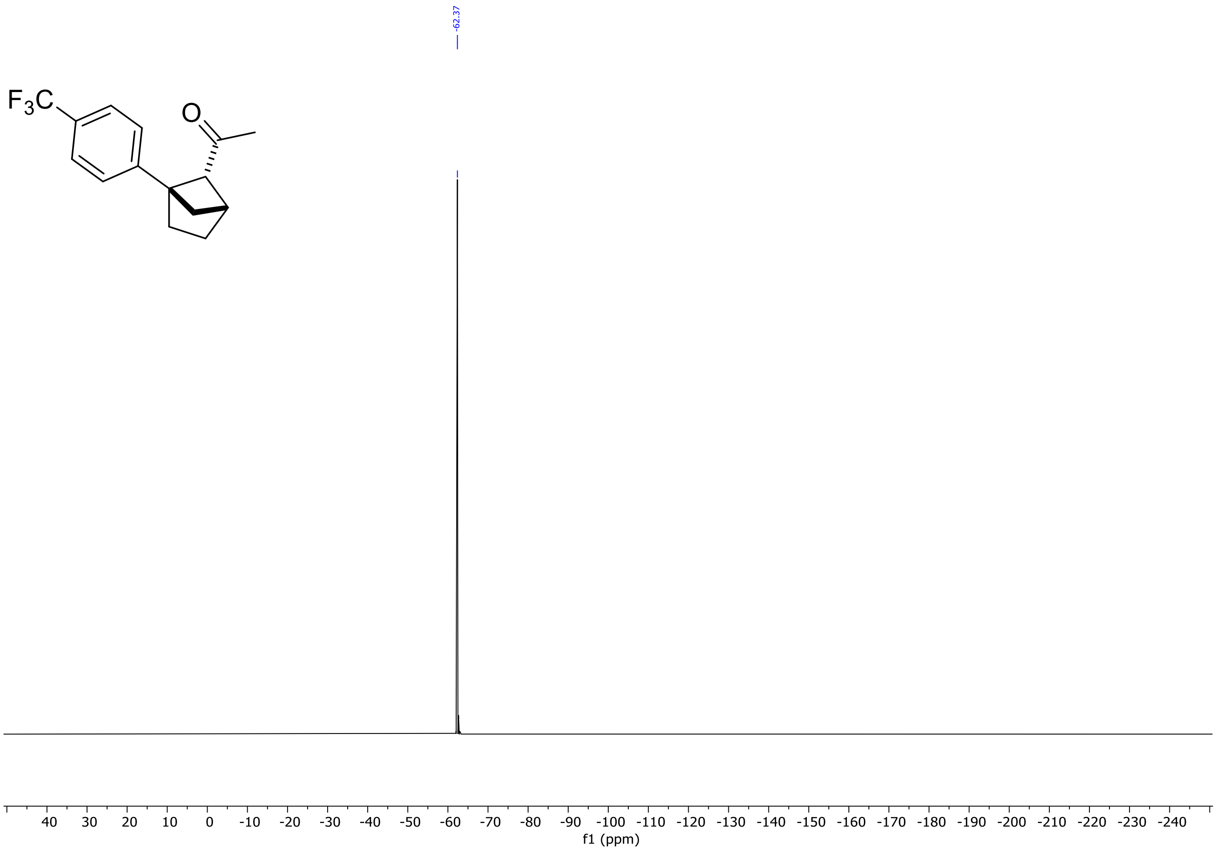

^{1}H-NMR (300 MHz, $CDCl_3$) of 1-(*p*-Tolyl)bicyclo[2.1.1]hexan-5-yl)ethan-1-one (**2f**)

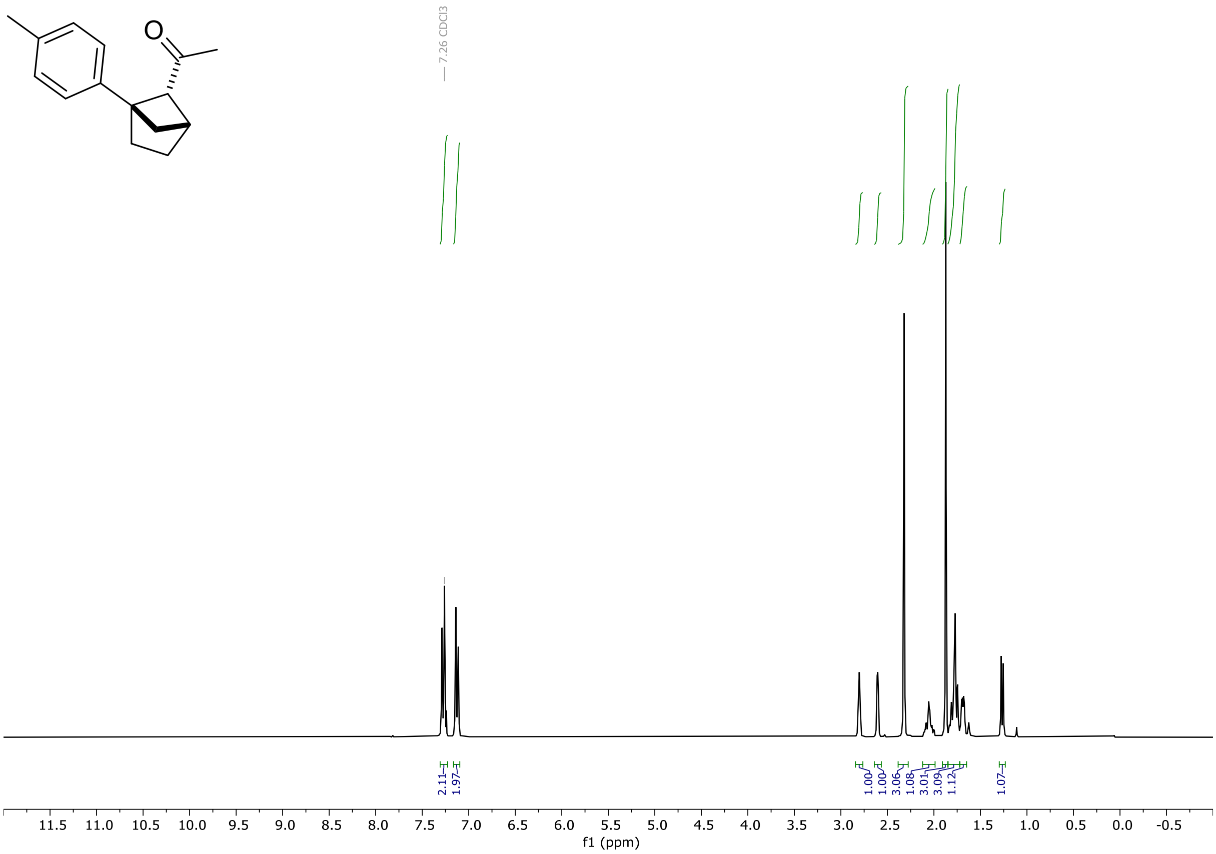

^{13}C-NMR (75 MHz, $CDCl_3$) of 1-(*p*-Tolyl)bicyclo[2.1.1]hexan-5-yl)ethan-1-one (**2f**)

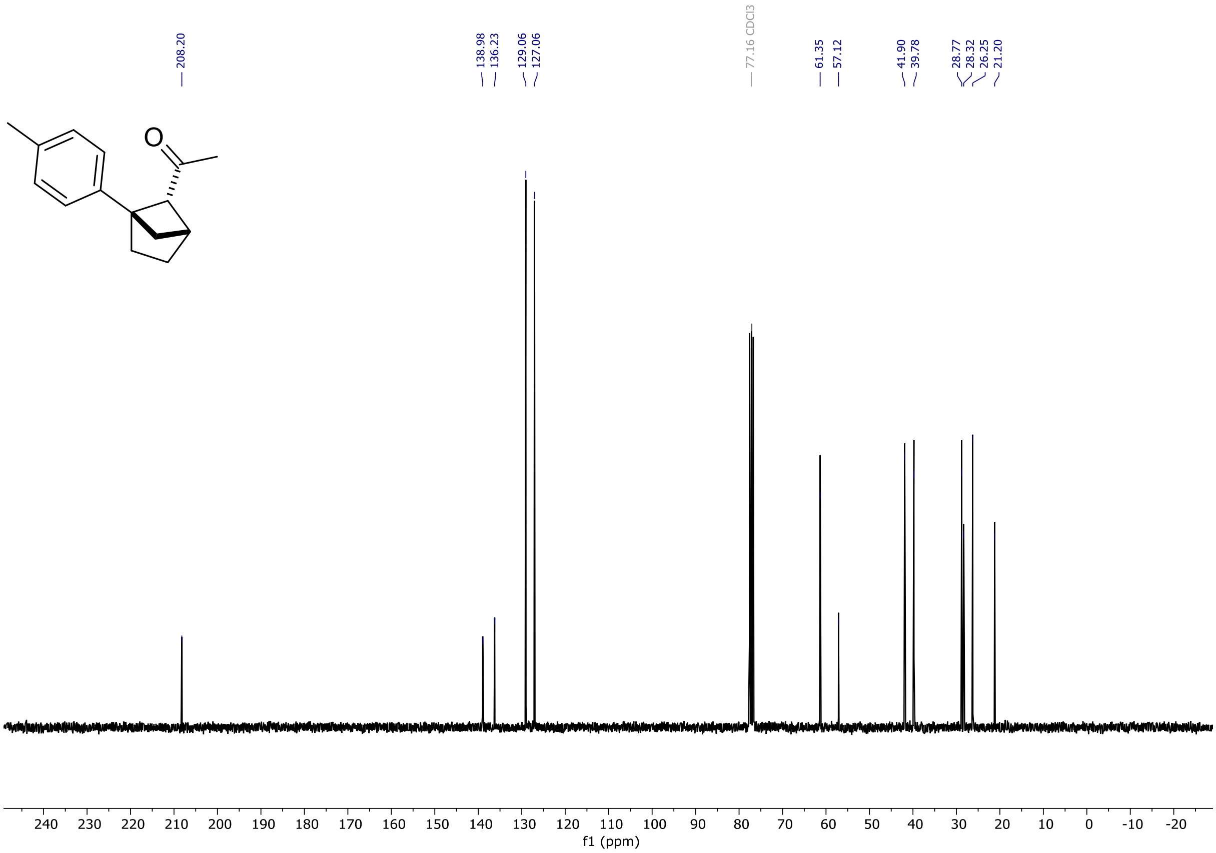

^{1}H-NMR (300 MHz, $CDCl_3$) of 1-(1-(*m*-tolyl)bicyclo[2.1.1]hexan-5-yl)ethan-1-one (**2g**)

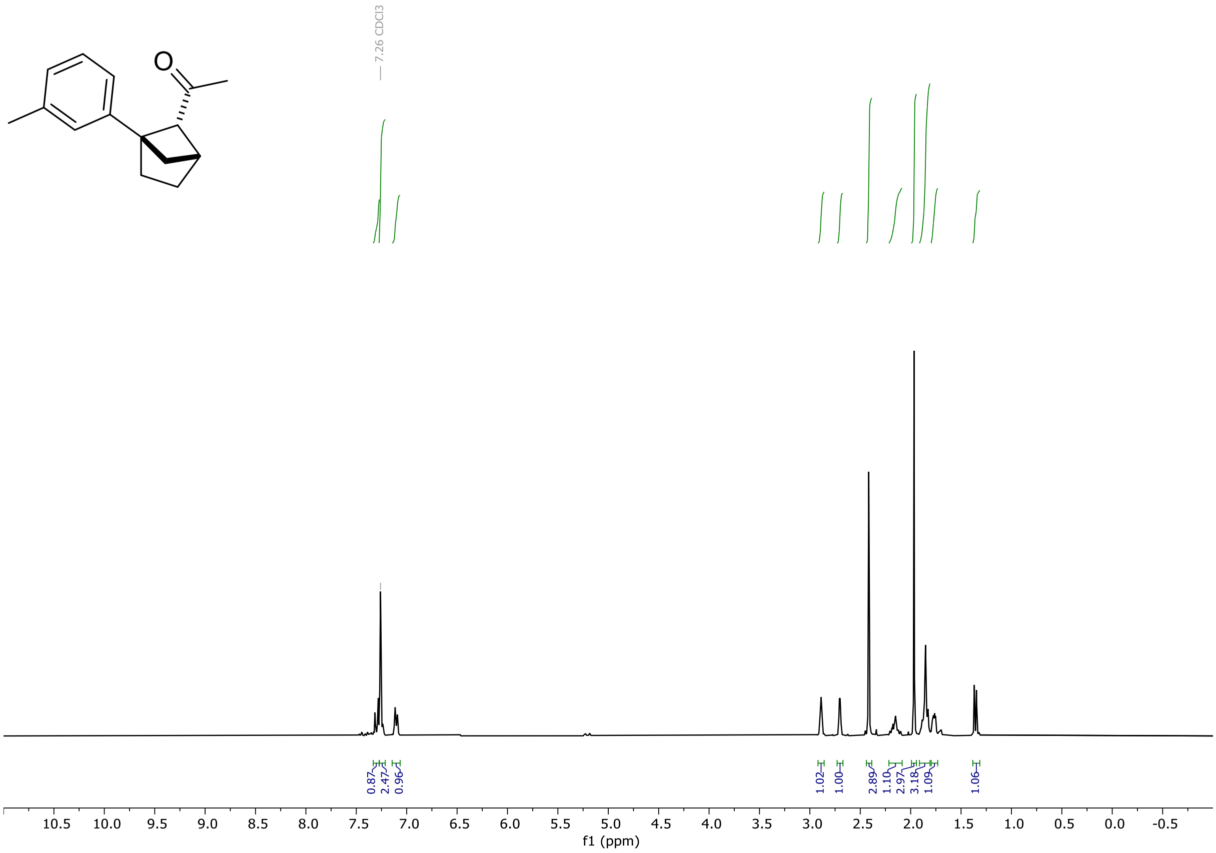

^{13}C-NMR (75 MHz, $CDCl_3$) of 1-(1-(*m*-tolyl)bicyclo[2.1.1]hexan-5-yl)ethan-1-one (**2g**)

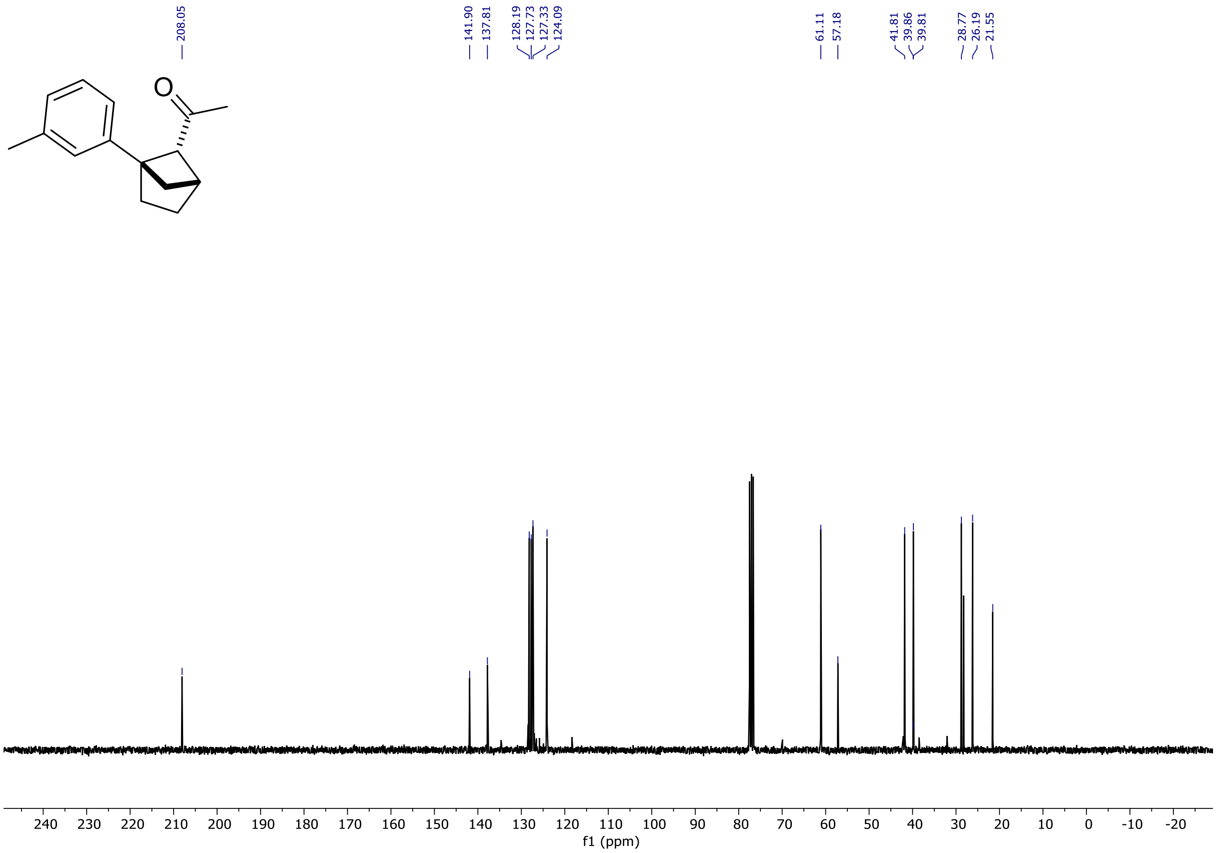

^{1}H-NMR (300 MHz, $CDCl_3$) of 1-(1-(Naphthalen-2-yl)bicyclo[2.1.1]hexan-5-yl)ethan-1-one (**2h**)

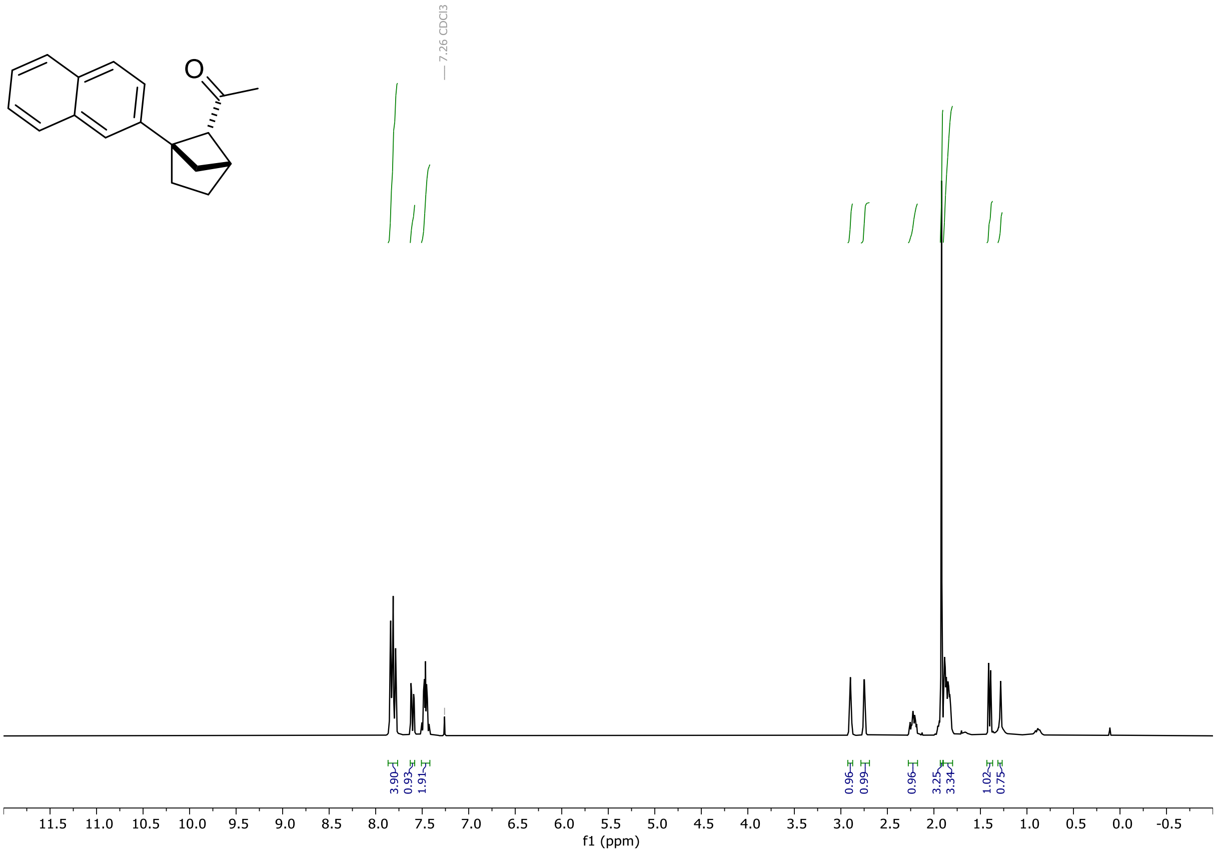

^{13}C-NMR (75 MHz, $CDCl_3$) of 1-(1-(Naphthalen-2-yl)bicyclo[2.1.1]hexan-5-yl)ethan-1-one (**2h**)

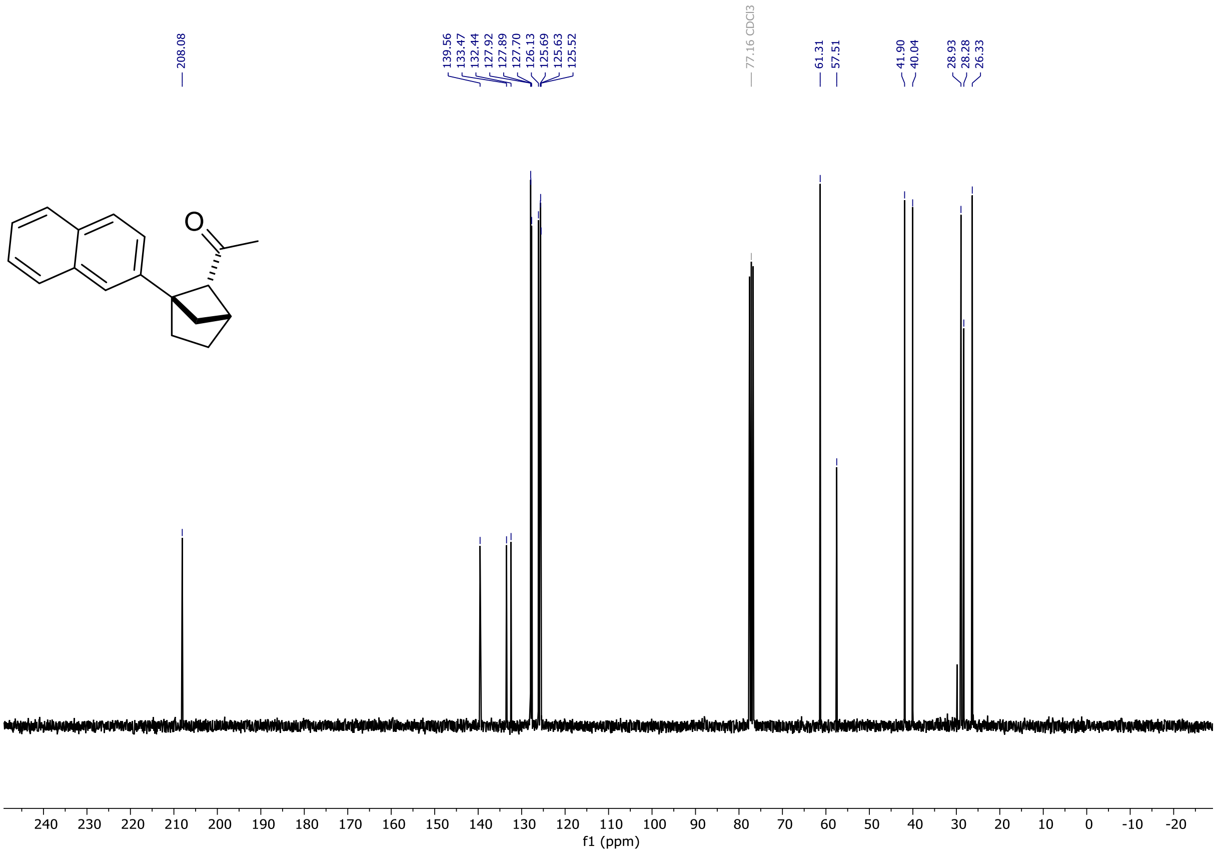

^{1}H-NMR (300 MHz, $CDCl_3$) of Cyclohexyl(1-phenylbicyclo[2.1.1]hexan-5-yl)methanone (**2i**)

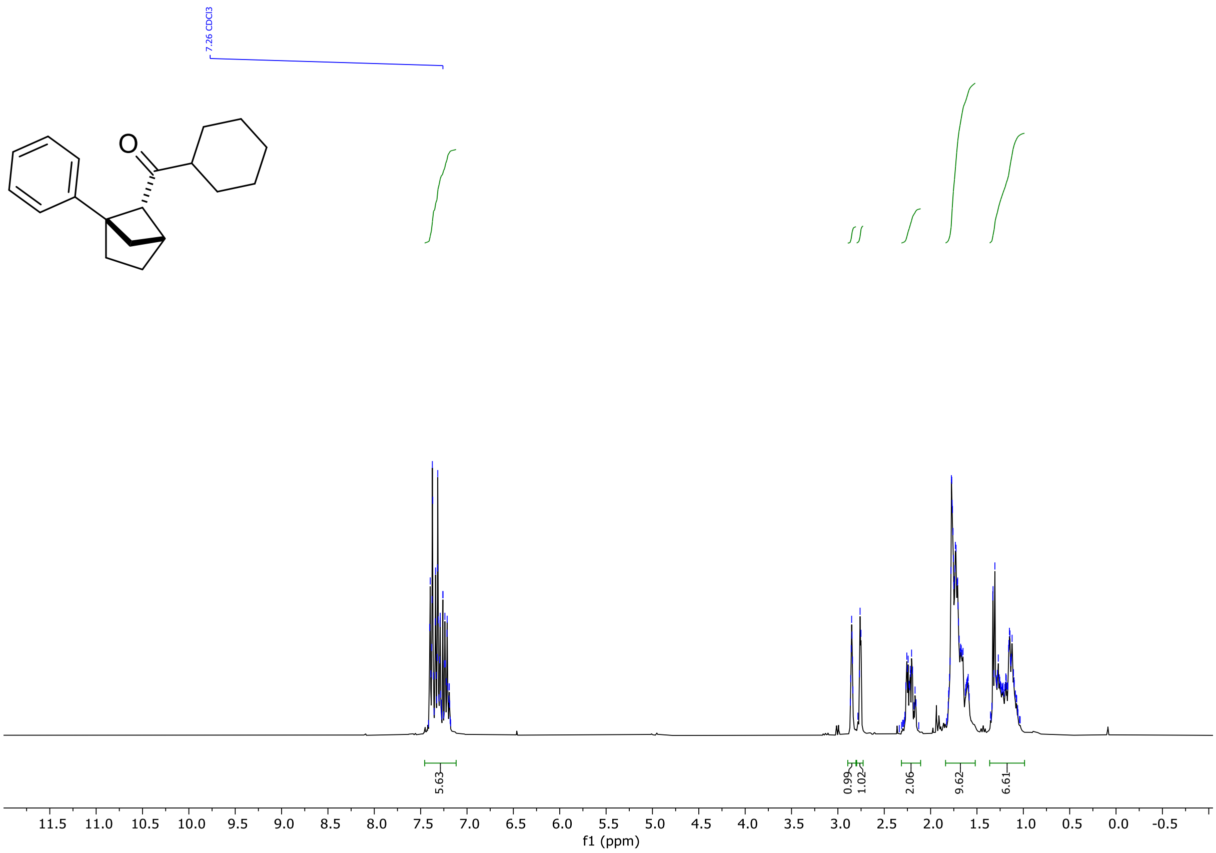

^{13}C-NMR (75 MHz, $CDCl_3$) of Cyclohexyl(1-phenylbicyclo[2.1.1]hexan-5-yl)methanone (**2i**)

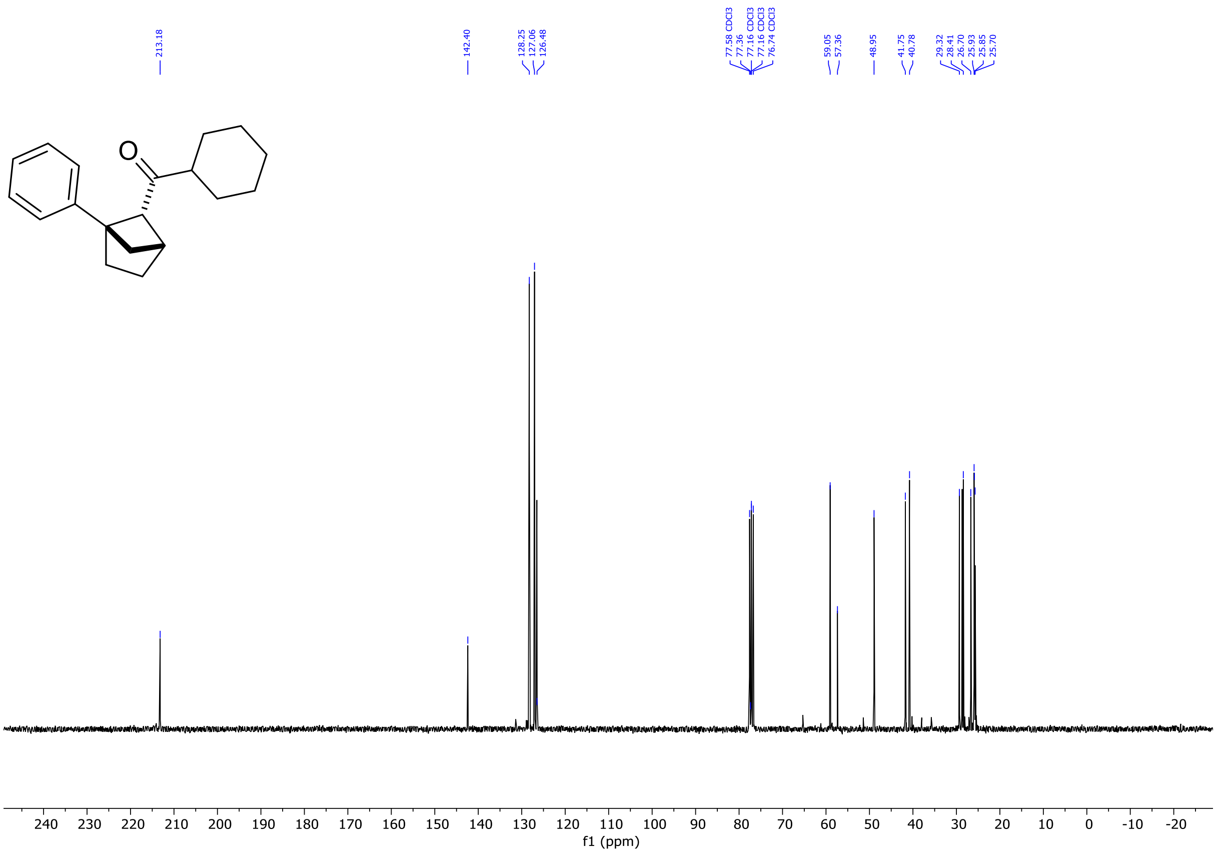

^{1}H-NMR (300 MHz, $CDCl_3$) of Phenyl-(1-phenylbicyclo[2.1.1]hexan-5-yl)methanone (**2j**)

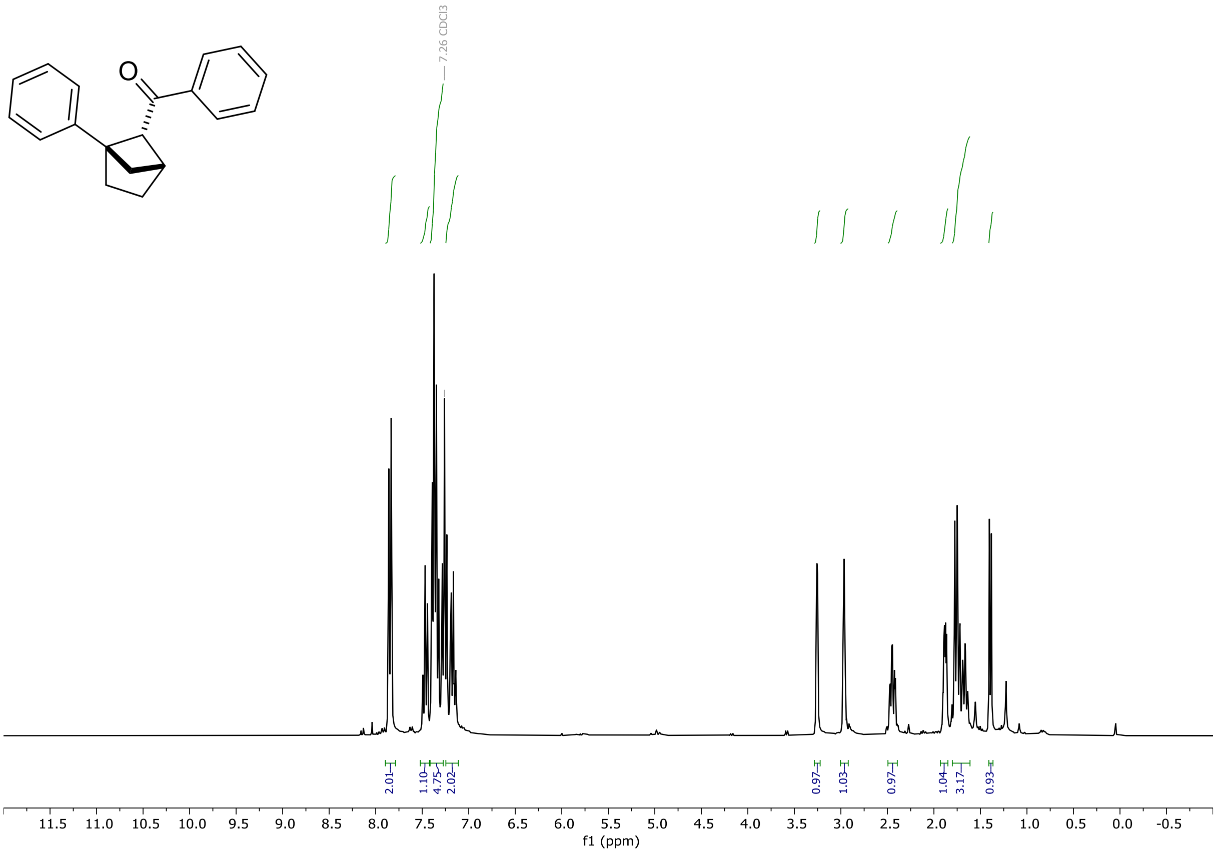

^{13}C-NMR (75 MHz, $CDCl_3$) of Phenyl-(1-phenylbicyclo[2.1.1]hexan-5-yl)methanone (**2j**)

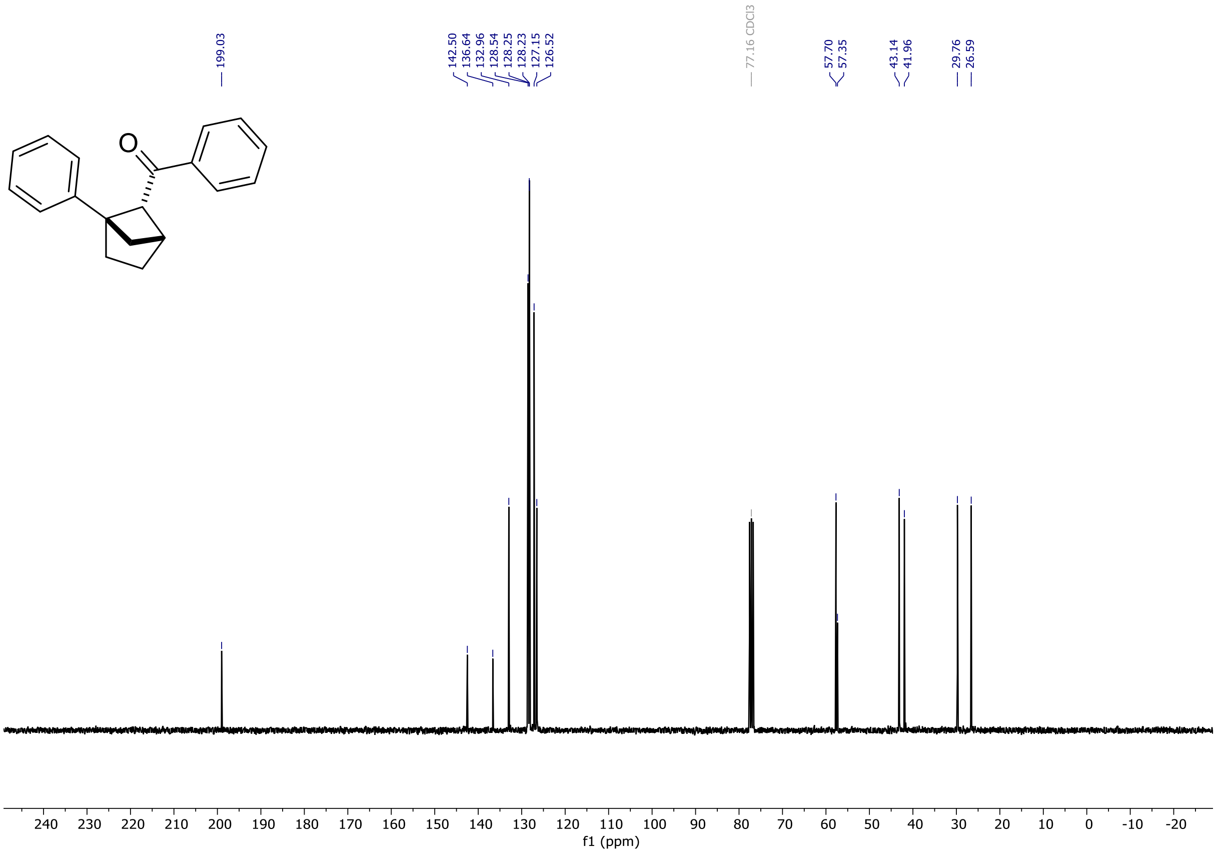

^{1}H-NMR (300 MHz, $CDCl_3$) of 4-(5-acetylbicyclo[2.1.1]hexan-1-yl)benzonitrile (**2k**)

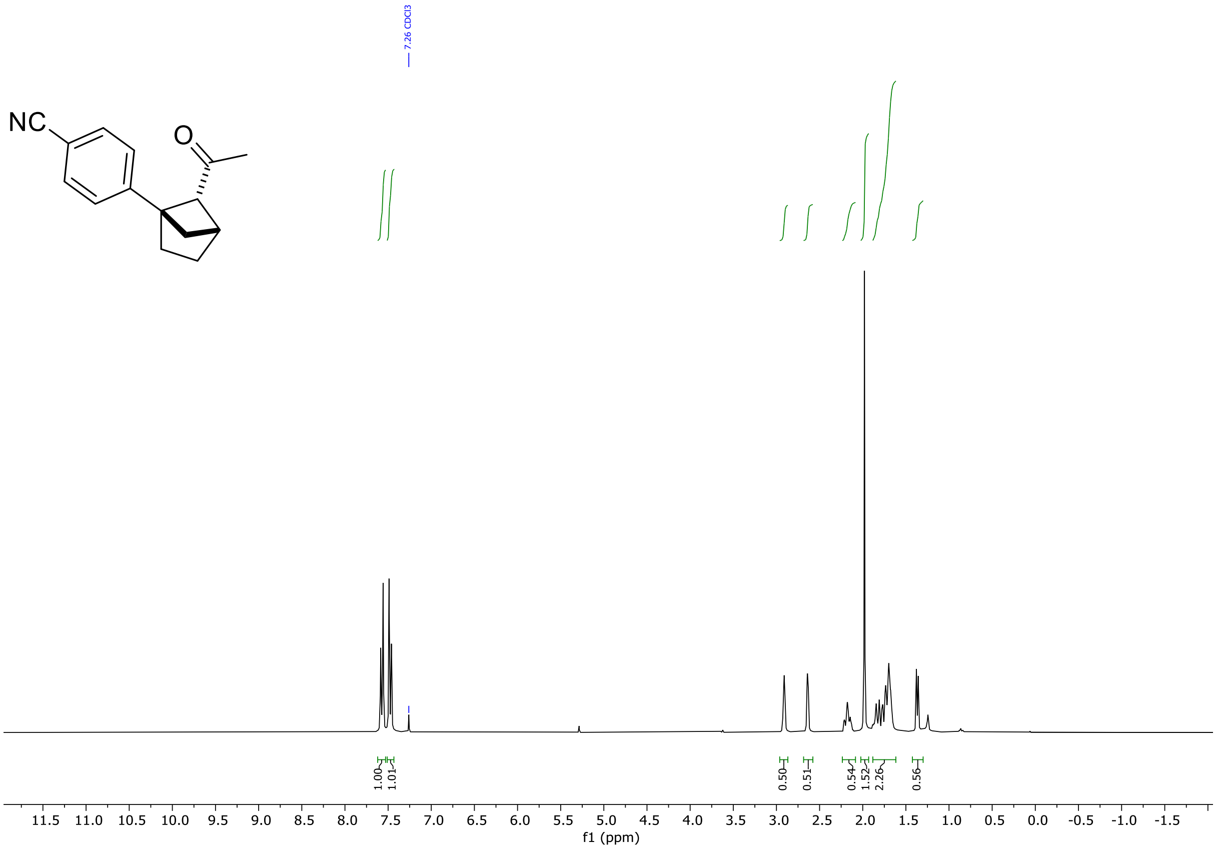

^{13}C-NMR (75 MHz, $CDCl_3$) of 4-(5-acetylbicyclo[2.1.1]hexan-1-yl)benzonitrile (**2k**)

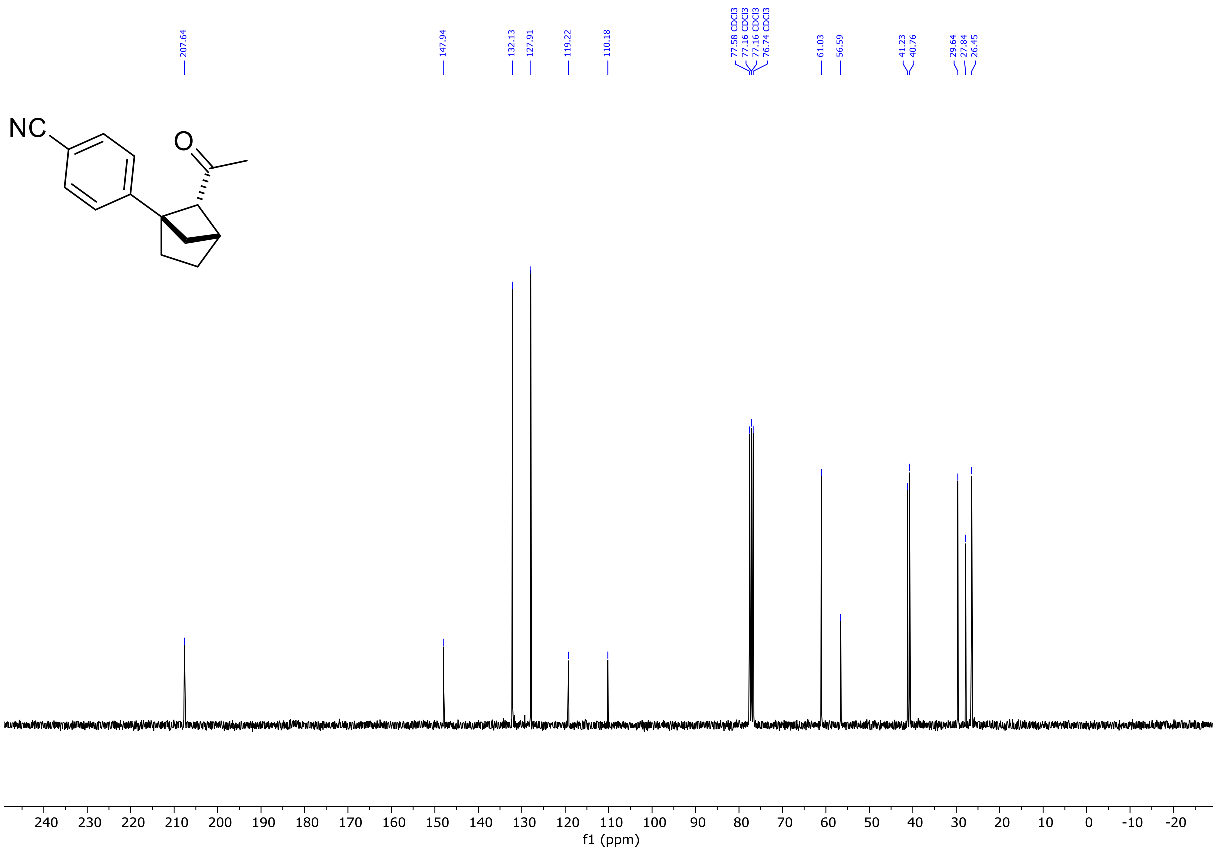

^{1}H-NMR (300 MHz, $CDCl_3$) of 1-(1-(Naphthalen-2-yl)bicyclo[2.1.1]hexan-5-yl)ethan-1-ol (**8**)

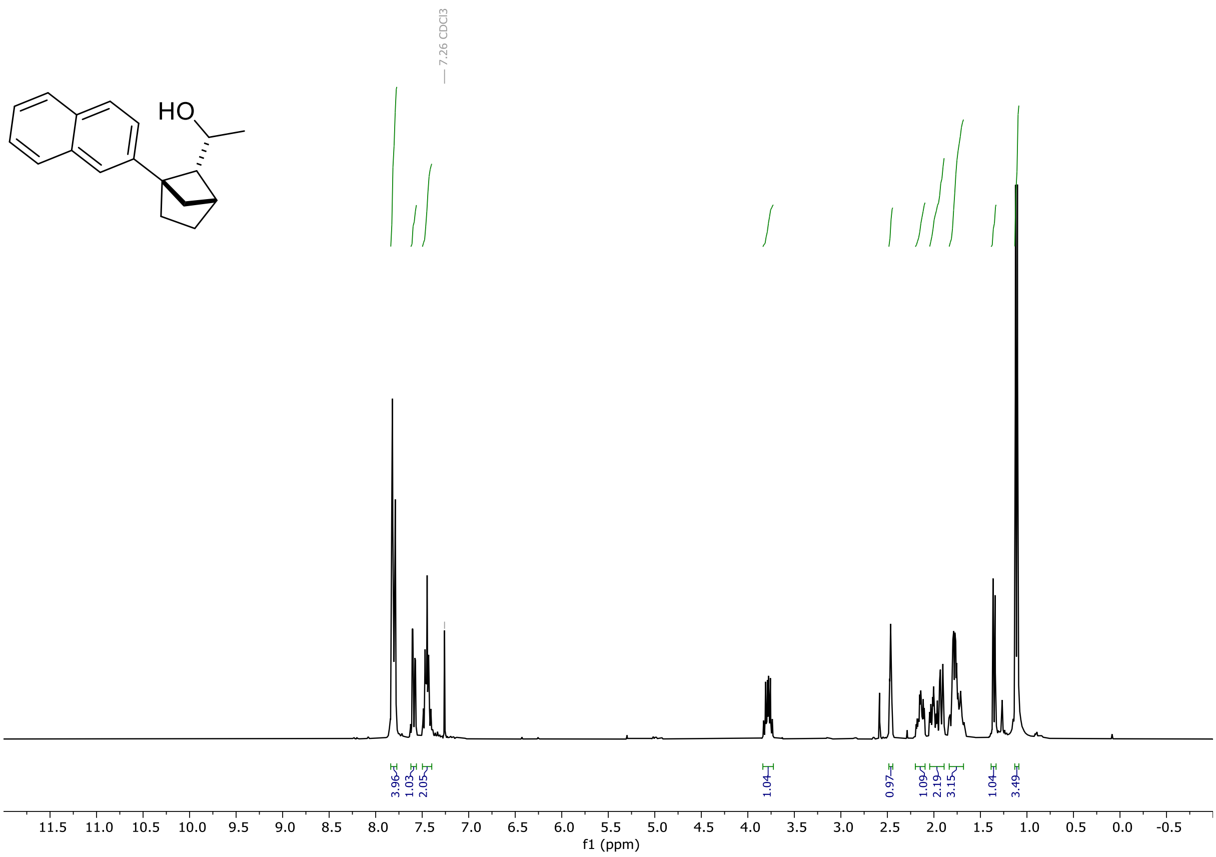

^{13}C-NMR (75 MHz, $CDCl_3$) of1-(1-(Naphthalen-2-yl)bicyclo[2.1.1]hexan-5-yl)ethan-1-ol (**8**):

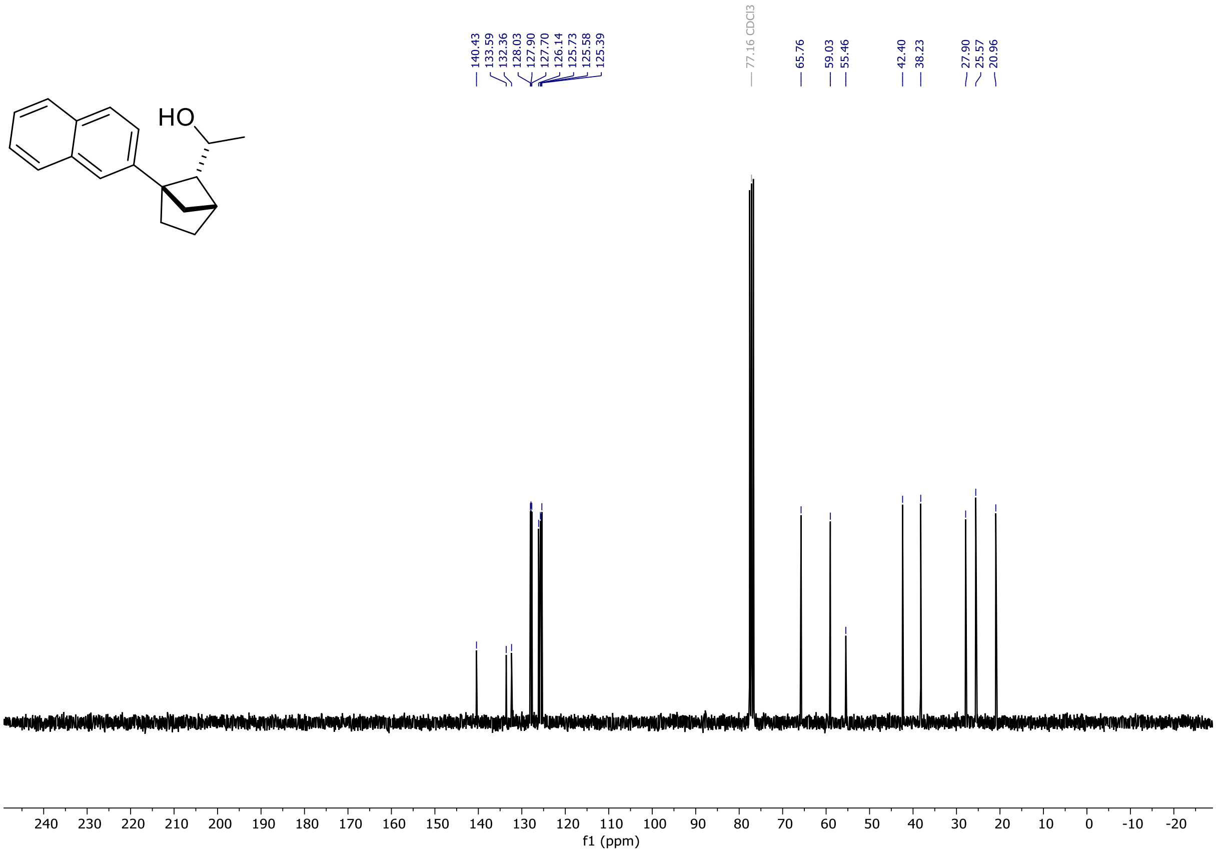

^{1}H-NMR (300 MHz, $CDCl_3$) of 1-(1-(Naphthalen-2-yl)bicyclo[2.1.1]hexan-5-yl)ethyl 4-chloro-3-nitrobenzoate (**6a**):

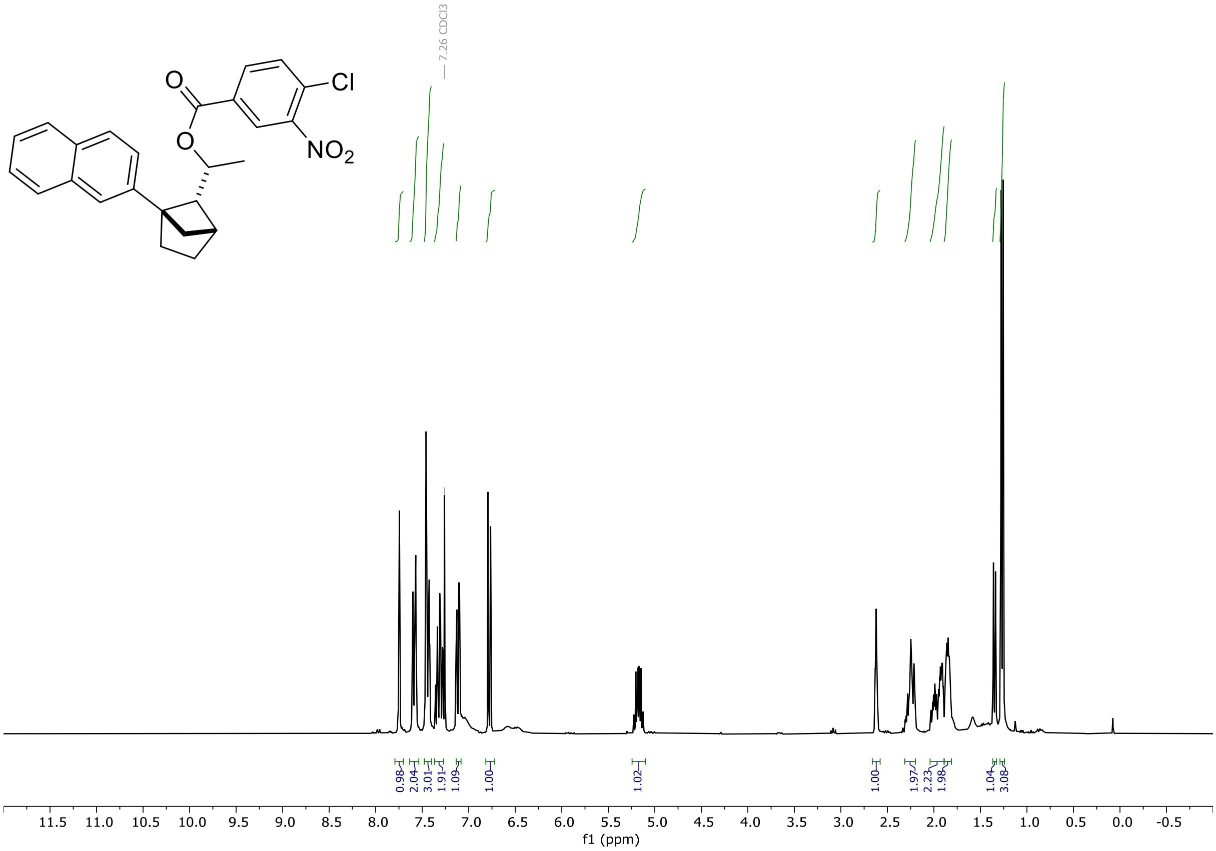

^{13}C-NMR (75 MHz, $CDCl_3$) of 1H-NMR (300 MHz, CDCl3) of 1-(1-(Naphthalen-2-yl)bicyclo[2.1.1]hexan-5-yl)ethyl 4-chloro-3-nitrobenzoate (**6a**):

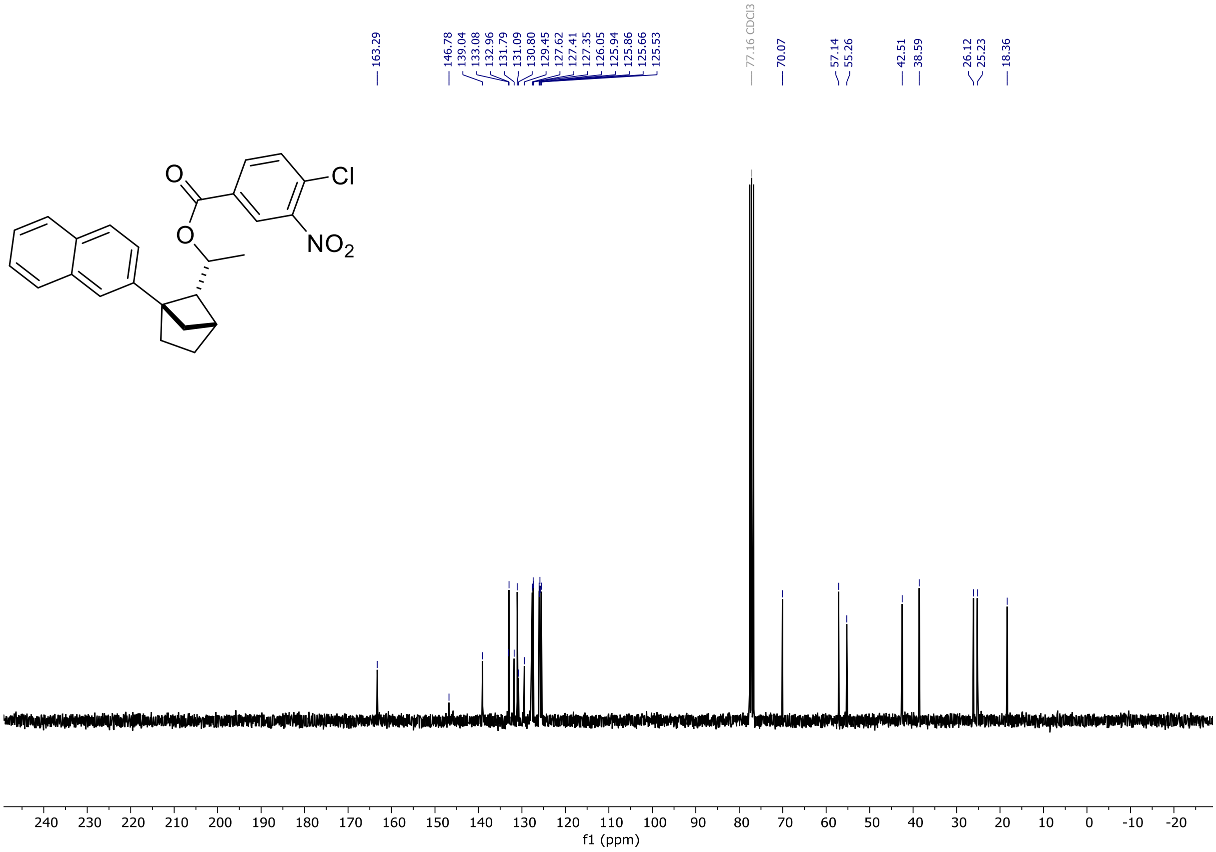

10. Supplementary References

[1] A. Denisenko, P. Garbuz, S. V. Shishkina, N. M. Voloshchuk, P. K. Mykhailiuk, *Angew. Chem. Int. Ed.,* **2020**, 59, 20515-20521.

[2] S. Nicolai, J. Waser, *Org. Lett.,* **2011**, 13, 23, 6324-6327.

[3] J. Lee, S. Wang, M. Callahan, P. Nagorny, *Org. Lett.,* **2018**, 20, 7, 2067-2070.

[4] F. Pecho, Y. Sempere, J. Gramüller, F. M. Hörmann, R. M. Gschwind, T. Bach, *J. Am. Chem. Soc.* **2021**, 143, 9350–9354.

[5] J. R. Lakowicz, Principles of fluorescence spectroscopy. Springer, New York, 3rd edn, 2006.

[6] Pavlishchuk, V. V.; Addison, A. W., *Inorganica Chim. Acta* **2000**, *298* , 97–102.

[7] Espinoza, E. M.; Clark, J. A.; Soliman, J.; Derr, J. B.; Morales, M.; Vullev, *J. Electrochem. Soc.* **2019**, *166*, H3175

[8] Glaser, F.; Kerzig, C.; Wenger, O. S., *Chem. Sci*., **2021**,12, 9922-9933.

[9] Bruker AXS (2021) APEX4 Version 2021.4-0, SAINT Version 8.40B and SADABS Bruker AXS area detector scaling and absorption correction Version 2016/2, Bruker AXS Inc., Madison, Wisconsin, USA.

[10] Sheldrick, G. M., *SHELXT – Integrated space-group and crystal-structure determination*, *Acta Cryst.,* **2015**, *A71*, 3-8.

[11] Sheldrick, G.M., *Crystal structure refinement with SHELXL*, *Acta Cryst.*, **2015**, *C71 (1)*, 3-8.

[12] Bruker AXS (**1998**) *XP – Interactive molecular graphics, Version 5.1,* Bruker AXS Inc., Madison, Wisconsin, USA.
